# Synthetic O‐Polysaccharide Backbone Units for a Single Antigen Vaccine Against Two Major Non‐Typhoidal *Salmonella* Serovars

**DOI:** 10.1002/anie.7740689

**Published:** 2026-05-07

**Authors:** Xingling Pan, Changxin Huo, Soham Maity, Herbert Kavunja, Rachel Moszyk, Cameron Talbot, Scott M. Baliban, Xuefei Huang

**Affiliations:** ^1^ Department of Chemistry Michigan State University East Lansing Michigan USA; ^2^ Institute For Quantitative Health Science & Engineering Michigan State University East Lansing Michigan USA; ^3^ Iaso Therapeutics Inc. East Lansing Michigan USA; ^4^ Center For Vaccine Development and Global Health University of Maryland School of Medicine Baltimore Maryland USA; ^5^ Department of Biomedical Engineering Michigan State University East Lansing Michigan USA

**Keywords:** broad‐spectrum vaccines, carbohydrate synthesis, o‐polysaccharide, *Salmonella* Enteritidis, *Salmonella* Typhimurium

## Abstract

*Salmonella* infections can cause life‐threatening systemic diseases in many regions of the world. With the prevalence of antimicrobial‐resistant *Salmonella*, vaccines are urgently needed to prevent and reduce infections. There are no vaccines available against any non‐typhoidal *Salmonella* (NTS) serovars, including two of the most common ones, that are, *Salmonella* Typhimurium and *Salmonella* Enteritidis. While traditional *Salmonella* vaccine approaches require a construct against each serotype, a new strategy has been developed in this work targeting the shared O‐polysaccharide backbone as a potential common vaccine antigen. Tri‐, hexa‐, and nona‐saccharides corresponding to one to three repeating units of the O‐polysaccharide backbone were synthesized stereoselectively via a modular approach. These oligosaccharides were conjugated to the bacteriophage Qβ carrier, which elicited strong IgG responses against the synthetic carbohydrate antigens upon immunization of both mice and rabbits. The trisaccharide antigen was sufficient to induce protective antibodies. The post‐immune sera from Qβ‐trisaccharide immunized rabbits recognized the native O‐polysaccharides from both *S*. Typhimurium and *S*. Enteritidis, and significantly protected mice against lethal challenges by these *Salmonella* serotypes. The ability of a single glycan antigen to protect against infections by two different NTS serovars is a significant step forward for broad‐spectrum anti‐*Salmonella* vaccine design.

## Graphical abstract

A modular strategy was developed to synthesize tri‐, hexa‐, and nona‐saccharides representing repeating units of the conserved O‐polysaccharide backbone of *Salmonella*. Conjugates of these glycans to bacteriophage Qβ induced strong IgG responses in mice and rabbits. Antibodies elicited recognized native O‐polysaccharides and conferred protection against lethal challenges by two major non‐typhoidal *Salmonella* species in mouse models.

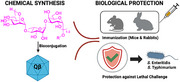

## 1 Introduction

*Salmonella* Enterica is a Gram‐negative bacterium, which can cause diseases ranging from self‐limiting gastroenteritis to severe, often life‐threatening, invasive infections, including enteric fever and invasive non‐typhoidal *Salmonella* (iNTS) diseases [1]. The non‐typhoidal *Salmonella* (NTS) infections cause approximately 94 million illnesses and 155 000 deaths annually in the world [2]. In the United States, according to the Centers for Disease Control and Prevention (CDC), *Salmonella* is one of the most common zoonotic diseases [3]. Severe *Salmonella* infections are commonly treated with antibiotics. However, many antimicrobial‐resistant *Salmonella* strains have emerged [4, 5]. For example, out of 680 *Salmonella* strains isolated in Italian clinics, nearly all (99.4%) were resistant to azithromycin [6]; 45.8% of these clinically isolated strains exhibited multidrug resistance, withmonophasic variant of *Salmonella* Typhimurium resistant to azithromycin (100.0%), tetracycline (93.0%), and ampicillin (92.4%) [6]. The alarming prevalence of such strains has prompted the World Health Organization to rate *Salmonella* as high priority on their bacterial priority pathogen list [7]. Complementary to the development of novel antibiotics, effective anti‐*Salmonella* vaccines can play important roles in controlling *Salmonella* infections.

One challenge for anti‐*Salmonella* vaccine development is that there are multiple pathogenic serovars, which are categorized as either typhoidal *Salmonella*, including *Salmonella* Typhi (*S*. Typhi), *Salmonella* Paratyphi (*S*. Paratyphi) A, B, and C, or NTS such as *Salmonella* Enteritidis (*S*. Enteritidis) and *Salmonella* Typhimurium (*S*. Typhimurium). To date, there are only vaccines approved for *S*. Typhi for human use, which are based on attenuated or killed *S*. Typhi bacteria, or Vi capsular polysaccharide present on the majority of *S*. Typhi strains [8, 9]. In the United States, the main disease‐causing serovars are NTS *S*. Enteritidis and *S*. Typhimurium. Although Vi capsular polysaccharide‐based vaccines are efficacious in preventing *S*. Typhi infections, they are not effective against NTS *S*. Enteritidis and *S*. Typhimurium due to a lack of Vi antigen expression.

In order to develop effective anti‐NTS vaccines, multiple antigens have been explored, which include cell surface proteins and carbohydrates [10]. One limitation of the current vaccine design is the low immune cross‐recognition between serogroups. In order to have a vaccine capable of providing protection against multiple pathogenic serovars, the traditional strategy is to prepare a distinct construct for each targeted serovar and administer multiple constructs together as a combination [11, 12, 13, 14, 15]. This need to develop multiple vaccine constructs complicates overall vaccine design and manufacturing.

To overcome the serovar specificity limitation, we aim to discover a protective antigen that can potentially be effective against multiple pathogenic serovars of *Salmonella*. In this work, we report the first step toward this goal, where we uncover that by targeting a conserved structure within the O‐polysaccharides of *S*. Typhimurium and *S*. Enteritidis, a single vaccine construct can be created to induce cross‐protective antibodies against these two major pathogenic NTS strains. This is the first time that the O‐polysaccharide backbone has been investigated as antigens for anti‐*Salmonella* vaccine development.

## 2 Results and Discussion

In search of a protective epitope, we explored the lipopolysaccharides of *Salmonella*. As Gram‐negative bacteria, *S*. Enteritidis and *S*. Typhimurium express lipopolysaccharides on the cell surface, which consist of an O‐polysaccharide chain linked to lipid A through a core oligosaccharide [10]. We previously examined the core of lipopolysaccharides as potential broad‐spectrum antigens. However, as the core is normally masked by the overlying O‐polysaccharide, the antibodies induced by the core oligosaccharide did not bind with bacteria unless the bacteria were pre‐treated with an inhibitor of lipopolysaccharide transport [16], which is a severe limitation.

Moving beyond the core oligosaccharide, we shifted our attention to the O‐polysaccharide, which is a polymer of repeating units consists of a conserved trisaccharide of →2)‐*α*‐D‐mannose (Man)‐(1→4)‐*α*‐L‐rhamnose (Rha)‐(1→3)‐*α*‐D‐galactose (Gal)‐α‐(1→ in the backbone (termed epitope O:12). This backbone can be variably α‐glucosylated at 6‐OH or 4‐OH of Gal, giving rise to epitopes O:1 and O:12‐2, respectively. A unique dideoxy sugar is attached to the 3‐*O* position of the Man, which is characteristic for each serovar with tyvelose for *S*. Enteritidis (epitope O:9) and abequose for *S*. Typhimurium (epitope O:4) [17, 18]. Prior studies have shown that the dideoxy sugar is the main immunodominant epitope recognized by antibodies generated against the O‐polysaccharide [10, 19, 20]. Studies of a panel of monoclonal antibodies against *S*. Typhimurium demonstrated that the antibodies specific against the abequose were more than 2500‐fold higher in protective activity than those against the backbone [21]. Antibodies can be highly specific against the dideoxy sugar, as those induced by a conjugate formed with *S*. Enteritidis O‐glycan did not bind with *S*. Paratyphi A O‐glycan and vice versa [19, 20]. These results collectively have shaped the general design of anti‐*Salmonella* vaccine to include the characteristic dideoxy sugar as antigens.

**SCHEME 1:**
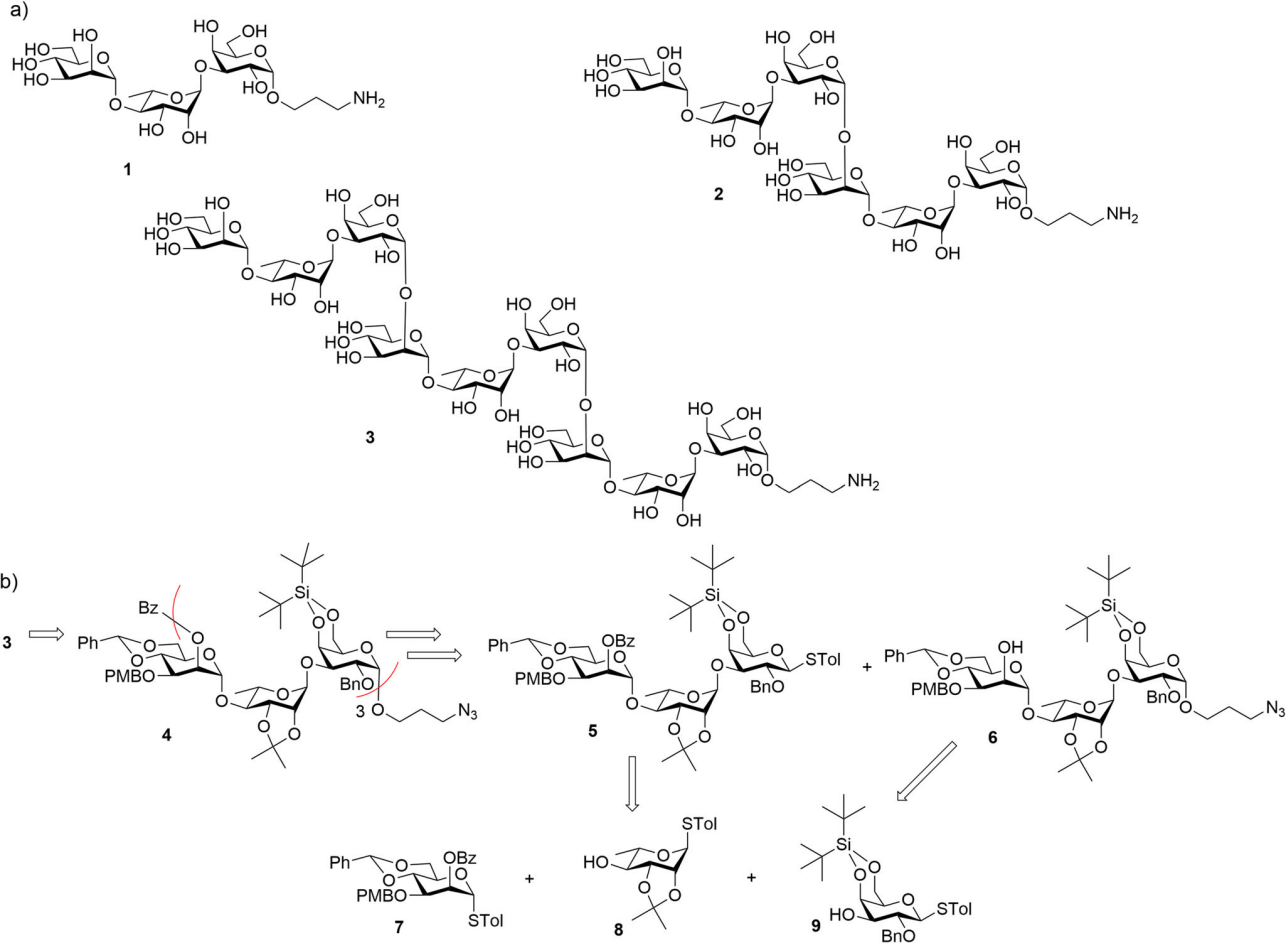
a) Structures of *Salmonella* tri‐, hexa‐, and nona‐saccharides **1**–**3** and b) retrosynthetic analysis of *Salmonella* O‐polysaccharide backbone nonasaccharide **3**.

In our journey toward the discovery of broad‐spectrum epitopes, we hypothesize that if high levels of antibodies can be elicited against the conserved O‐polysaccharide backbone through vaccination, it may provide protection against multiple *Salmonella* serovars. Vaccines specifically targeting the backbone have not been investigated to date. We decided to synthesize tri‐, hexa‐, and nona‐saccharides **1**–**3** (Scheme 1a) corresponding to one to three repeats of the O:12 backbone sequences with an amino propyl chain at the reducing end for bioconjugation. The availability of these well‐defined synthetic carbohydrates enabled us to investigate their antigenicity, and establish the structure and activity relationship.

### 2.1 Syntheses of Tri‐, Hexa‐, and Nona‐Saccharide Antigens 1–3

To prepare *Salmonella* tri‐, hexa‐, and nona‐saccharides **1**–**3** that bear all α‐glycosyl linkages, we envisioned a trisaccharide block approach (Scheme 1b). Retrosynthetically, nonasaccharide **3** can be derived from the fully protected nonasaccharide **4**, which in turn could be produced through iterative coupling of trisaccharide donor **5** with trisaccharide acceptor **6**, followed by deprotection. To access **5** and **6**, three thioglycosyl monosaccharide building blocks **7**–**9** were designed.

**SCHEME 2:**
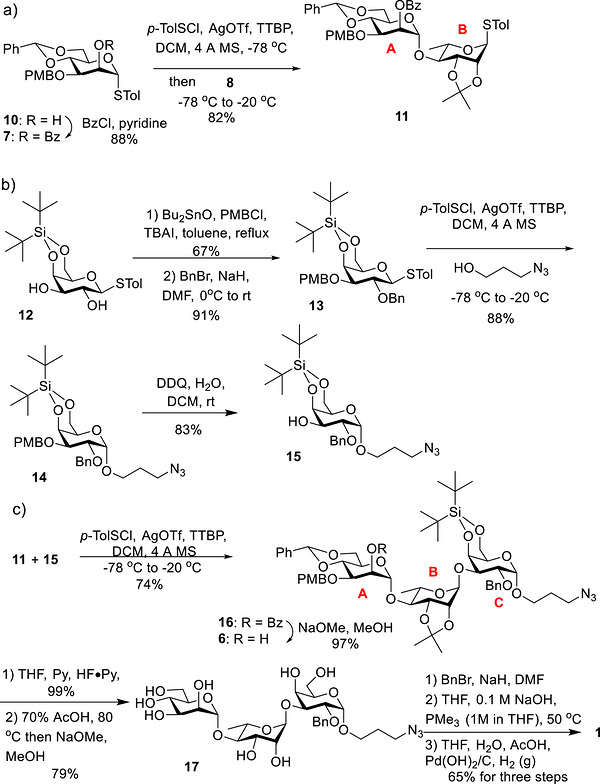
Synthesis of *Salmonella* trisaccharide **1**.

Synthesis of the O‐polysaccharide backbone started from the thiomannoside **10** [22] (Scheme 2). Protection of the free 2‐OH of **10** with benzoyl (Bz) led to mannosyl donor **7**. Pre‐activation [23] of donor **7** with the promoter system *p*‐tolylsulfenyl chloride (*p*‐TolSCl)/silver triflate (AgOTf) at −78°C followed by the addition of the bifunctional rhamnoside **8** [24] led to disaccharide **11** in 82% yield (Scheme 2a). The reducing end galactoside was synthesized from galactoside diol **12** [25] that bears a silylidene group at its 4,6‐*O* positions [26] to facilitate the formation of α‐galactosidic linkage (Scheme 2b). To differentiate the 2‐ and 3‐ OH positions of galactoside **12**, it was treated with dibutyltin oxide, followed by *p*‐methoxylbenzyl (PMB) chloride, selectively installing the PMB group on the 3‐*O* position. Subsequent protection of the 2‐OH with benzyl (Bn) produced galactoside donor **13**. Glycosylation of **13** with 3‐azido 1‐propanol promoted by *p*‐TolSCl/AgOTf led to α‐galactoside **14** in 88% yield, the PMB of which was deprotected to lead to the galactoside acceptor **15** (Scheme 2b). The anomeric configuration of **14** was confirmed to be α by nuclear magnetic resonance (NMR) analysis (^3^J_H1,H2_ = 3.5 Hz). Glycosylation of **15** by the disaccharide donor **11** led to trisaccharide **16** in 74% yield (Scheme 2c). The stereochemistry of the three anomeric centers of **16** was confirmed to be all α, as determined by one‐bond ^1^H‐^13^C coupling constants of the anomeric centers [27] (^1^J_1H‐13C_ = 174.5, 172.0, and 170.0 Hz for rings A, B, C, respectively). Reacting **16** with sodium methoxide removed its Bz group, leading to trisaccharide **6**. Treatment of **6** with HF·pyridine followed by 70% acetic acid in water deprotected the silylidene, PMB, and benzylidene groups. As some side reactions with acetic acid occurred, transforming OH groups to *O*‐acetate, an additional Zemplén *O*‐deacetylation step was performed, generating trisaccharide **17**. With the high polarity of **17**, to facilitate product purification upon azide reduction, it was per‐benzylated first. The azide group of the product was subsequently reduced under Staudinger reduction, followed by catalytic hydrogenolysis, leading to the fully deprotected *Salmonella* trisaccharide **1** (Scheme 2c). While it is possible to reduce azide and remove benzyl groups via catalytic hydrogenolysis in one pot, we opted for the two‐step procedure, as it gave higher yields based on our prior experience [28].

**SCHEME 3:**
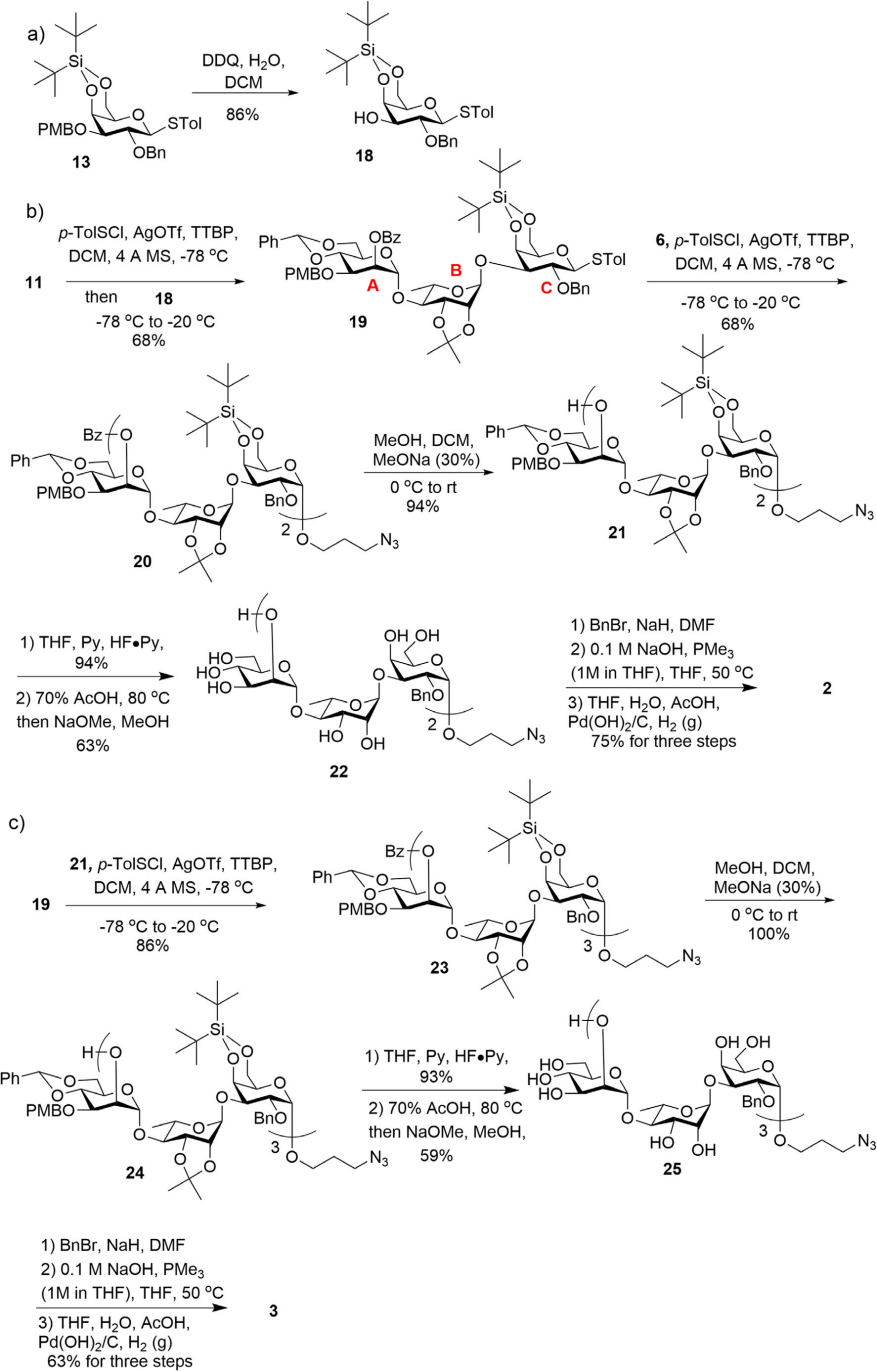
Syntheses of *Salmonella* hexa‐ and nona‐saccharides **2** and **3**.

Building on the success of trisaccharide synthesis, we next moved onto *Salmonella* hexa‐ and nona‐saccharides **2** and **3** through a modular approach. The PMB moiety in the silylidene protected galactosyl donor **13** was selectively removed by 2,3‐dichloro‐5,6‐dicyano‐1,4‐benzoquinone (DDQ) (Scheme 3a). The resulting bifunctional acceptor **18** was glycosylated by the disaccharide donor **11** under the pre‐activation condition, leading to trisaccharide donor **19** in 68% yield (Scheme 3b). The stereochemistry of the three anomeric centers of **19** was determined to be α, α, and β for rings A, B, C by NMR (^1^J_1H‐13C of C1_ = 173.0, 171.0, and 157.0 Hz for rings A, B, and C, respectively) [27]. The subsequent 3+3 glycosylation of donor **19** with acceptor **6** successfully generated the hexasaccharide **20** in 80% yield. The α‐stereochemistry of the newly formed glycosyl linkage between Gal and Man in **20** was confirmed by NMR analysis with ^1^J_1H‐13C_ of its anomeric center determined to be 173.0 Hz [27]. The Bz group in **20** was removed under the Zemplén condition leading to hexasaccharide **21** bearing a free OH group. **21** was then deprotected in a similar manner to trisaccharide **1** producing the fully deprotected hexasaccharide **2** (Scheme 3b).

To synthesize the nonasaccharide **3**, hexasaccharide acceptor **21** was glycosylated by the trisaccharide donor **19**, leading to the nonasaccharide **23** in 86% yield (Scheme 3c). The ^1^H‐^13^C one‐bond coupling constants of nine anomeric centers of **23** from the non‐reducing end to the reducing end were determined to be 173.5, 172.5, 173.5, 171.0, 172.5, 173.5, 171.0, 172.5, 168.5 Hz, thus suggesting all nine anomeric centers have α configurations [27]. Full deprotection of **23** produced the *Salmonella* nonasaccharide **3**, which is the longest *Salmonella* glycan chemically synthesized to date.

### 2.2 Conjugation of *Salmonella* Glycans With Bacteriophage Qβ

**SCHEME 4:**
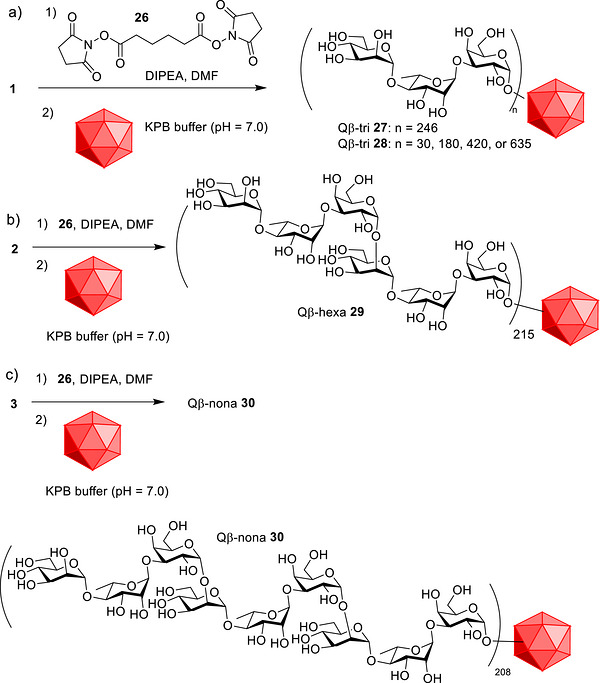
Syntheses of Qβ‐*Salmonella* glycan conjugates **27**–**30**.

It is known that carbohydrate antigens, when administered alone, generally do not produce strong antibody responses. It is critical that the antigens are covalently conjugated with an immunogenic carrier [29, 30, 31]. We have developed a bacteriophage Qβ virus‐like particle‐based carrier system [32], which has been shown to be superior to common protein carriers such as keyhole limpet hemocyanin (KLH), tetanus toxoid, and cross‐reactive material‐197 (CRM‐197) [33, 34, 35, 36]. With the structurally well‐defined *Salmonella* glycans **1**‐**3** in hand, their conjugation with Qβ was investigated. The glycans were first derivatized with a di‐*N*‐hydroxysuccinimide (NHS) ester bifunctional linker **26** (Scheme 4). The resulting NHS ester was then incubated with Qβ, which reacted with amine groups on the Qβ surface to form a covalent conjugate. A Qβ particle is composed of 180 copies of a coat protein. For trisaccharide **1**, when 12 eq of the glycan per coat protein monomer was used for conjugation, the resulting conjugate Qβ‐trisaccharide **1** (Qβ‐tri) contained an average of 1.36 copies of glycan per coat protein, corresponding to 246 of glycan per capsid as quantified by mass spectrometry (MS) (Scheme 4a). Using a similar approach, Qβ conjugates with hexasaccharide **2** (Qβ‐hexa) and nonasaccharide **3** (Qβ‐nona) were formed and characterized by MS with similar loading levels (Scheme 4b,c and Figures S1–S3). The tri‐, hexa‐, and nona‐saccharide antigens were also conjugated with bovine serum albumin (BSA) using the bifunctional linker **26** (Scheme S1 and Figures S4–S6) for analysis of levels of anti‐glycan antibodies produced through vaccination.

### 2.3 Immunization of Mice With Qβ‐Glycan Conjugates Generated Strong Anti‐Glycan IgG Antibody Response and a Threshold Antigen Density per Qβ Particle Was Observed

**FIGURE 1:**
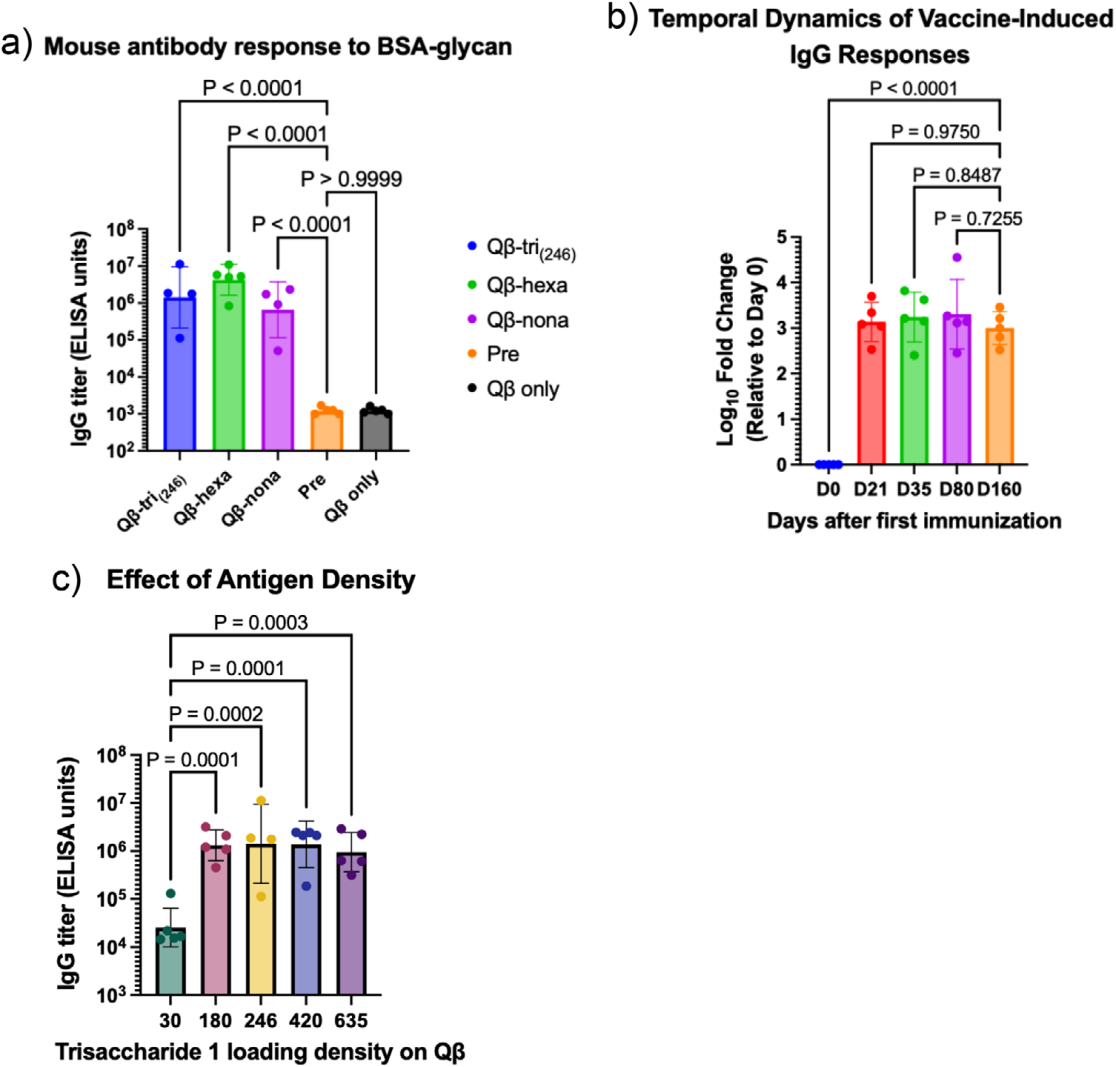
(a) Anti‐glycan IgG titers from mice immunized with Qβ only, Qβ‐trisaccharide **1** (Qβ‐tri_(246)_), Qβ‐hexasaccharide **2** (Qβ‐hexa), and Qβ‐nonasaccharide **3** (Qβ‐nona), respectively. Qβ‐glycan conjugates elicited significant levels of IgG antibodies against the immunizing glycan antigen as compared to the pre‐immunized (Pre) sera or sera from Qβ immunized mice (Qβ only). One‐way Anova showed significant differences in geometrical mean titers of anti‐glycan IgG antibodies of Qβ‐glycan immunized groups compared to pre‐immune sera or Qβ immunized mice, and no significant differences among the three Qβ‐glycan immunized groups; (b) IgG responses induced by Qβ‐tri_(246)_ were persistent. Fold change values were calculated as post‐immune IgG titers/Day 0 IgG titer and log_10_‐transformed. Significant anti‐glycan IgG titers were observed Day 21 after immunization and remained high on Day 160 compared with pre‐immune (Day 0) levels, indicating durable long‐term antibody responses were induced; (c) anti‐glycan IgG titers from mice immunized Qβ‐tri with different glycan loading. Statistical significance was determined using one‐way ANOVA followed by Dunnett's multiple comparisons test. Each symbol corresponds to an individual animal. Data are presented as geometric mean values ± standard deviation.

To evaluate the immunogenicity of our vaccine constructs, mice were immunized with Qβ‐tri_(246)_ (246 copies of glycans on average for each Qβ particle; 3 µg of trisaccharide **1** per dose per mouse) with the prime injection on Day 0, followed by booster injections on Days 14 and 28. Control mice received Qβ only at the equivalent amounts of Qβ protein as in Qβ‐tri_(246)_ immunized mice. To boost the immune response, an adjuvant was added to each injection as part of the formulation. Two adjuvants, that is, Freund's adjuvant and monophosphoryl lipid A (MPLA) isolated from *Salmonella* Minnesota (*S*. Minnesota), were tested first. Blood was drawn from each mouse on Days 0 and 35. Enzyme‐linked immunosorbent assay (ELISA) analyses of Day 35 sera using the BSA trisaccharide **1** conjugate as the coating antigen were performed. For mice immunized with Qβ‐tri_(246)_ with Freund's adjuvant, ELISA showed that significant serum IgG antibody titers were induced with the geometric mean (GMT) titer against the immunizing glycan reaching 1.4 × 10^6^ ELISA units (Figure 1a). IgM responses were also detected in these mice, although IgM levels were substantially lower with GMT values approximately 0.005% of those of IgG, suggesting IgG was the predominant antibody isotype on Day 35 (Figure S7a). We next analyzed subtypes of IgG antibodies. All major IgG subclasses, including IgG1, IgG2b, IgG2c, and IgG3, were detected, indicating balanced humoral responses (Figure S7a). In contrast to mice receiving Qβ‐tri_(246)_, control mice immunized with Qβ only gave anti‐glycan IgG GMTs below 1000 ELISA units. Interestingly, when MPLA was used as the adjuvant instead, the control group of mice receiving Qβ also gave significant anti‐glycan IgG titers. This may be because the MPLA adjuvant derived from *S*. Minnesota contained some residual lipopolysaccharide. Thus, MPLA adjuvant was not pursued further in this study.

With the ability of Qβ‐glycan conjugate to induce high antibody levels established, the persistence of the antibody response was measured. The anti‐glycan IgG titers remained high over 160 days (Figure 1b), suggesting long‐lived plasma cells were elicited through vaccination.

Another immunization parameter we investigated was the need for booster injections. We immunized a group of mice (*n* = 5) with the Qβ‐tri conjugate only once (3 µg of trisaccharide 1 per dose per mouse). Blood was collected from these mice on Day 35 after immunization and serum levels of anti‐glycan 1 IgG titers were measured. As shown in Figure S7b, one dose immunization also elicited significant levels of anti‐glycan 1 IgG antibodies, with GMT values about 10% of those for mice receiving one prime and two booster vaccinations. These results suggest booster injections significantly enhanced the IgG titers of anti‐glycan 1 antibodies produced.

To evaluate the effects of antigen loading on Qβ particles, several additional Qβ‐tri conjugates were prepared with average trisaccharide loading levels of 30, 180, 420 and 635 by varying the amounts of antigen added during conjugation (Scheme 4a). Four additional groups of mice (*n* = 5 per group) were immunized with these Qβ‐tri conjugates bearing an average of 30, 180, 420, and 635 copies of glycans, respectively. Identical total amounts of glycan 1 were administered per dose following the one prime and two boost immunization schedules. On Day 35 following the prime immunization, the levels of anti‐glycan IgG were determined by ELISA. As shown in Figure 1c, while the group of mice receiving the Qβ‐tri conjugate with an average of 30 copies of glycan per capsid gave the lowest responses, there were no significant differences among groups receiving Qβ‐tri conjugates with 180, 246, 420, and 635 copies of glycan. The average GMT titers from these groups were about 50 times higher than that from mice immunized with Qβ‐tri conjugate loaded with 30 copies of glycans, even though the total amounts of glycan used per dose were the same for all groups. This suggests that there is a threshold antigen density needed for the vaccine construct. At a low antigen density (30 copies per capsid), the conjugate may not be able to efficiently crosslink B cell receptors on cognizant B cells, resulting in weak B cell activation [37, 38, 39]. Increasing the average number of antigens to 180 or higher per capsid can significantly enhance B cell receptor crosslinking, leading to strong B cell activation and high levels of antibody production.

We next compared the length of the antigen on IgG responses. Groups of mice were immunized with Qβ‐hexa and Qβ‐nona conjugates, respectively, using the one‐prime and two‐boost immunization protocol. On Day 35 following prime immunization, the levels of anti‐glycan IgG titers were measured against the corresponding BSA‐glycan conjugates. As shown in Figure 1a, high levels of IgG antibodies were induced with IgG GMTs of 4.2 × 10^6^ and 6.6 × 10^5^ ELISA units, respectively, for mice immunized with Qβ‐hexa and Qβ‐nona.

### 2.4 Immunization of Rabbits With Qβ‐Glycan Conjugates Generated Strong Anti‐glycan IgG Antibody Responses

**FIGURE 2:**
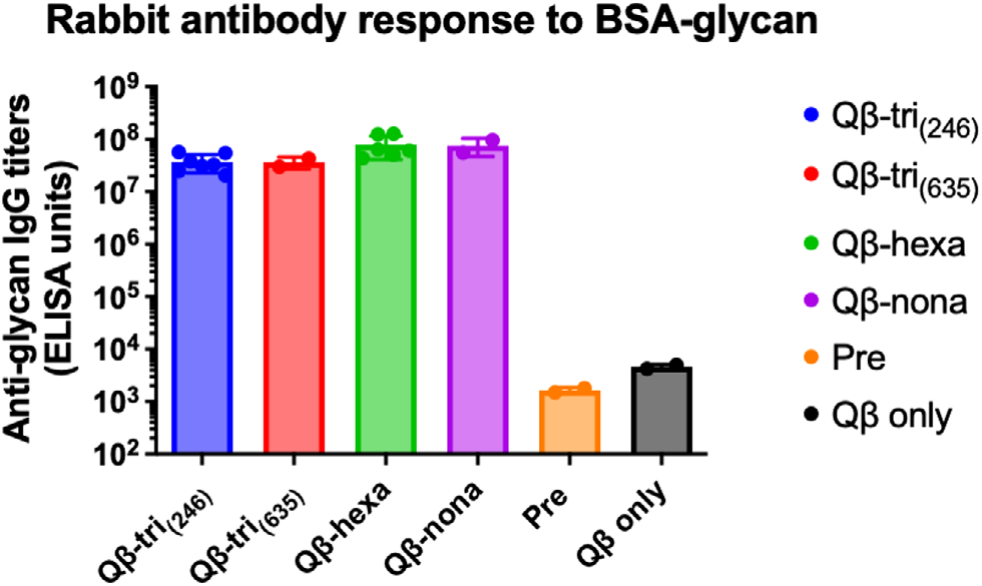
Anti‐glycan IgG titers of rabbits immunized with Qβ‐tri_(246)_, Qβ‐tri_(635)_, Qβ‐hexa, and Qβ‐nona on Day 56 post prime immunization as measured by ELISA against the corresponding BSA‐glycan conjugates. All groups of rabbits produced high levels of anti‐glycan IgG antibodies. In contrast, levels of anti‐glycan IgG in pre‐immunized rabbits (Pre) or rabbits immunized with Qβ only were below 6000 ELISA units. Each symbol corresponds to an individual animal. Data are presented as geometric mean values ± standard deviation.

To establish the translational potential of the vaccine, rabbits were immunized with Qβ‐tri, Qβ‐hexa, and Qβ‐nona, respectively. Prime immunizations were performed subcutaneously on Day 0 with complete Freund's adjuvants, which were followed by booster injections given subcutaneously on Days 14, 28, and 42 mixed with incomplete Freund's adjuvant. On Day 56, all groups of rabbits produced significant IgG titers as measured against the corresponding BSA‐glycan conjugates (Figure 2). Similar to mouse studies, no significant differences were observed in rabbits immunized with Qβ‐trisaccharide conjugates with 246 (Qβ‐tri_(246)_) or 635 (Qβ‐tri_(635)_) copies of glycans per particle (Figure 2). Likewise, high levels of IgG antibodies were also induced by Qβ‐hexa and Qβ‐nona conjugates (Figure 2).

**FIGURE 3:**
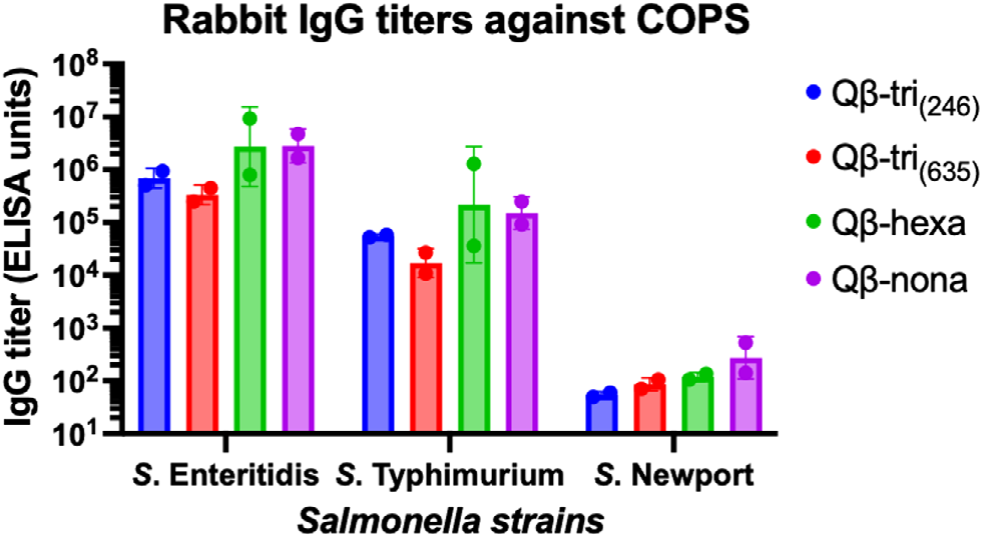
Anti‐COPS IgG ELISA titers in sera from rabbits immunized with Qβ‐tri, Qβ‐hexa, and Qβ‐nona respectively against COPS from *S*. Enteritidis, *S*. Typhimurium, and *S*. Newport. High levels of anti‐COPS IgG antibodies were induced against *S*. Enteritidis, and *S*. Typhimurium, while IgG titers against *S*. Newport with a different COPS structure were low demonstrating high selectivities of the antibodies induced. Each symbol corresponds to an individual animal. Data are presented as geometric mean values ± standard deviation.

In addition to BSA‐glycan conjugates, we analyzed IgG antibody recognition of native O‐polysaccharides. The core and O‐polysaccharides (COPS) were isolated from three serovars of *Salmonella*, that is, *S*. Enteritidis, *S*. Typhimurium, and *Salmonella* Newport (*S*. Newport). Compared to those from *S*. Enteritidis (O9,12) and *S*. Typhimurium (O4, [5],12), the COPS from *S*. Newport has a different carbohydrate sequence (O:6,8) [40], which was studied as a control to determine antibody specificities [17, 18]. The post‐vaccination sera from Qβ‐tri, Qβ‐hexa, and Qβ‐nona immunized rabbits bound with the COPS from *S*. Enteritidis and *S*. Typhimurium strongly (Figure 3). These results suggest that trisaccharide 1 with a single repeating unit is sufficient to induce antibodies recognizing the native COPS. The antibodies produced were sequence selective since their binding to COPS from *S*. Newport was only at background levels. Further studies were then performed using sera from Qβ‐tri immunized rabbits.

**FIGURE 4:**
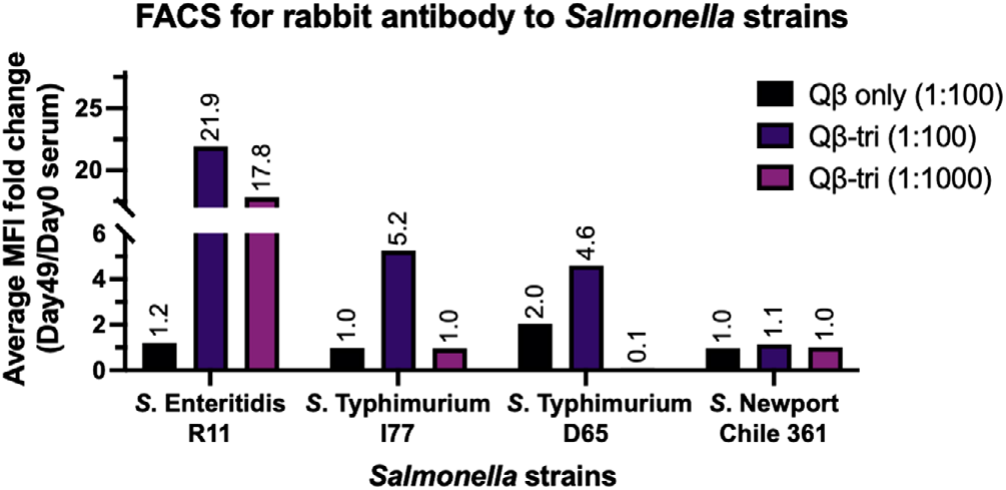
The binding of sera from rabbits immunized with Qβ‐tri**_(635)_** to *S*. Enteritidis R11, *S*. Typhimurium I77, *S*. Typhimurium D65, and *S*. Newport Chile 361 as measured by flow cytometry. The ratios of average mean fluorescence intensity (MFI) of Day 49 sera over pre‐immune sera on Day 0 are plotted. The number above each column is the average fold of change. Higher levels of anti‐COPS IgG antibodies were induced against *S*. Enteritidis, and *S*. Typhimurium, while IgG binding from all rabbits to *S*. Newport were low.

The binding of post‐vaccination sera with *Salmonella* bacteria was studied next by flow cytometry to assess whether antibodies induced could recognize intact bacteria. *S*. Enteritidis R11 [41], *S*. Typhimurium I77 [41] and D65 [42] (R11, I77, and D65 were all invasive clinical strains isolated from patients in Mali), and *S*. Newport Chile 361 [43] were analyzed. The immune sera bound well with both strains of *S*. Typhimurium as well as *S*. Enteritidis R11 (Figure 4). Negligible binding was observed by sera from control rabbits immunized with Qβ only. *S*. Newport Chile 361 was not recognized well by the immune sera. These results confirm that Qβ‐tri induced antibodies in rabbits selectively bound *S*. Typhimurium and *S*. Enteritidis, a key step for enabling anti‐bacterial activity.

**FIGURE 5:**
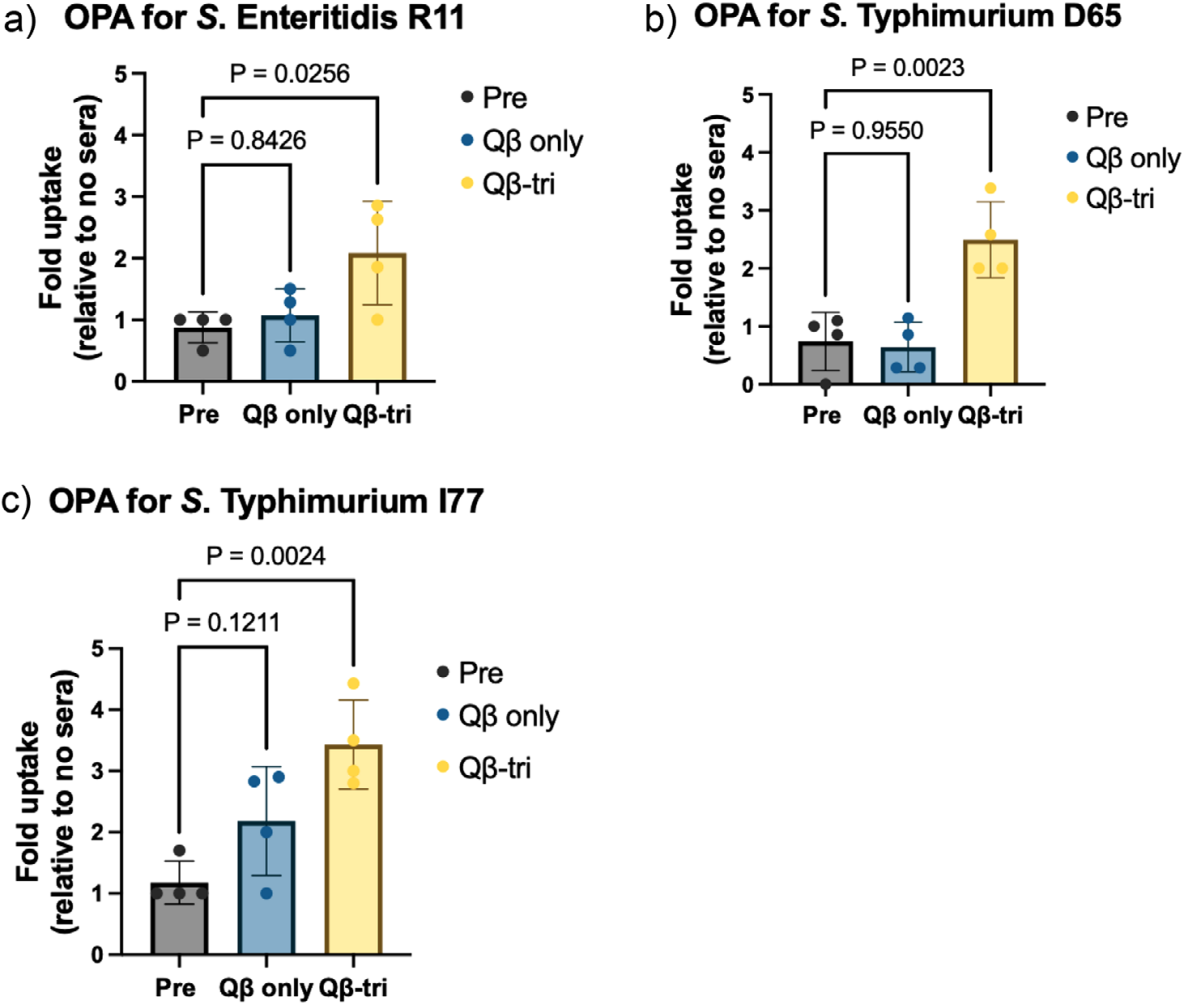
Sera from rabbits immunized with Qβ‐tri**_(635)_** exhibited significant OPA activities to (a) *S*. Enteritidis R11, (b) *S*. Typhimurium D65, and (c) *S*. Typhimurium I77 by human macrophage THP‐1 cells. Statistical significance was determined using one‐way ANOVA followed by Dunnett's multiple comparisons test. Each data point represents one technical replicate (four replicates per condition).

Opsonophagocytic activity (OPA) of antibody‐bound bacteria by phagocytes is an important functional mechanism for antibody‐mediated clearance of NTS [44]. The uptakes of *S*. Enteritidis R11, *S*. Typhimurium I77, and *S*. Typhimurium D65 by human macrophage THP‐1 cells were evaluated after incubation with sera from pre‐immune, Qβ immunized, or Qβ‐tri_(635)_ immunized rabbits (Figure 5). The sera from pre‐immune and Qβ immunized rabbit (Qβ only) did not lead to significant bacterial uptake relative to media alone. In contrast, antisera from Qβ–tri_(635)_ immunized rabbits markedly enhanced macrophage OPA of all three strains of bacteria.

**FIGURE 6:**
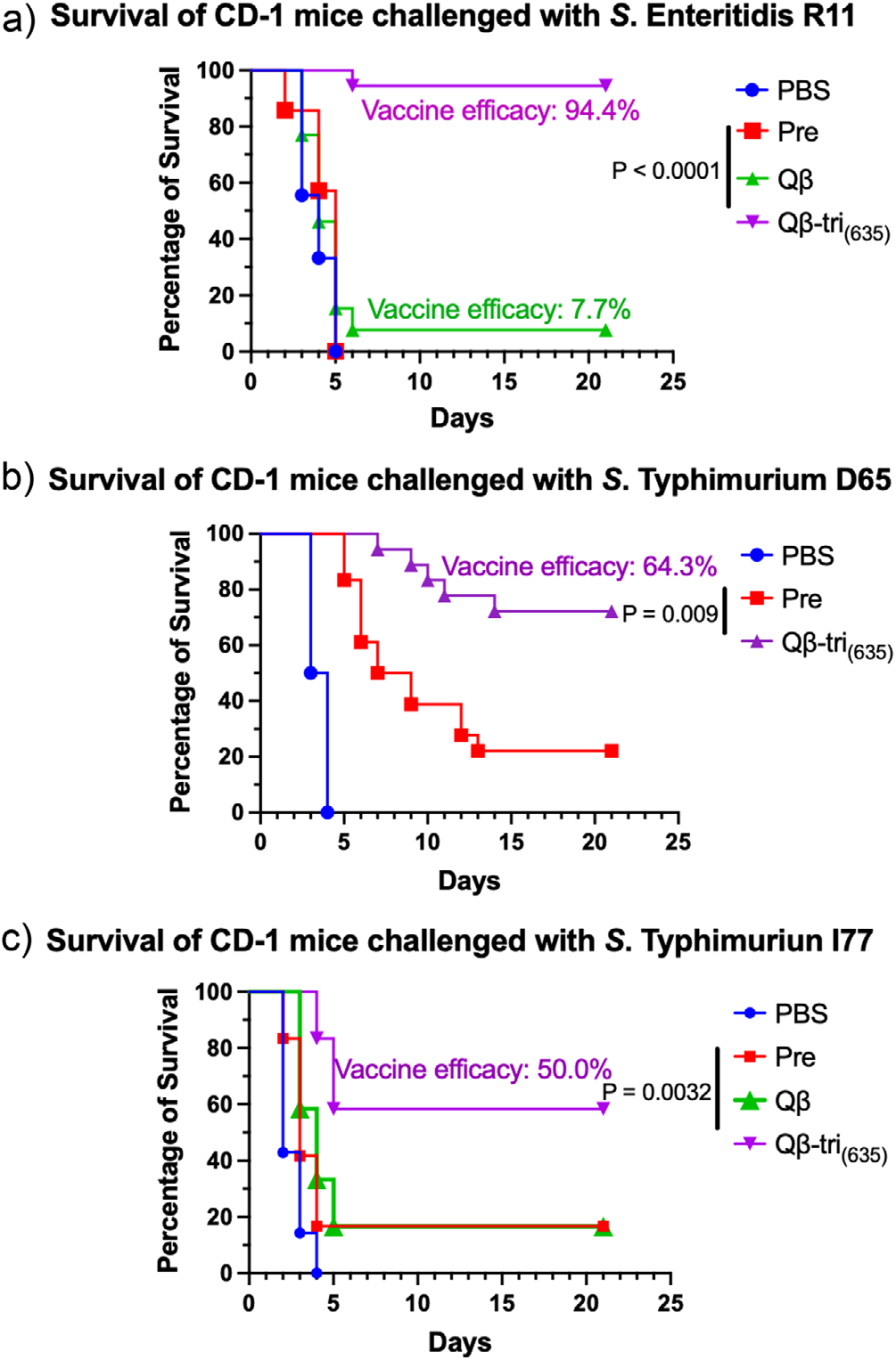
Sera from rabbits immunized with Qβ‐tri**_(635)_** exhibited significant protection of CD1 mice against lethal challenges by (a) *S*. Enteritidis R11, (b) *S*. Typhimurium D65, and (c) *S*. Typhimurium I77. Statistical analysis for survival was performed using the logrank test. Vaccine efficacy was calculated as: (1‐mortality in immune sera group/mortality in pre‐immune sera group) × 100%.

With the functional activities of the antisera established, we tested their abilities to provide protection against lethal challenges of *Salmonella*. As no rabbit models for *Salmonella* infections are available, mouse models were utilized. Naïve mice were administered with PBS (n = 10 for R11 and D65, *n* = 7 for I77), pre‐immune sera (*n* = 18 for R11 and D65, *n* = 12 for I77), sera from Qβ immunized rabbits (*n* = 12), or sera from Qβ‐tri_(635)_ immunized rabbits (*n* = 18 for R11 and D65, *n* = 12 for I77), which were followed by intraperitoneal injection with an LD100 dose of *S*. Enteritidis R11 (1.3 ×10^6^ colony forming units (CFU)), *S*. Typhimurium I77 (5 ×10^5^ CFU), or *S*. Typhimurium D65 (6.5 × 10^5^ CFU). While all mice receiving PBS died within 5 days, the Qβ‐tri_(635)_ sera provided significant protection, with vaccine efficacies of 94.4%, 64.3%, and 50.0% against *S*. Enteritidis R11, *S*. Typhimurium D65, and *S*. Typhimurium I77, respectively (Figure 6 and Table S1). For mice that survived the lethal challenge, their organs, including lungs, kidneys, and spleen, were extracted. No bacteria were detected in the examined organs, indicating successful eradication of *Salmonella* from these mice.

It is recognized that the complete Freund's adjuvant is not suitable for human vaccine formulation due to its adverse side effects. For potential future application in humans, we further examined Alum, an FDA‐approved adjuvant, for our mouse immunization study. Furthermore, rabbits were immunized with Qβ‐tri with Alum as the adjuvant following the protocol of one prime and three booster injections every two weeks. High anti‐glycan IgG antibody titers were induced in both mice and rabbits (Figure S8a,b). The rabbit anti‐sera recognized COPS from *S*. Enteritidis R11 and *S*. Typhimurium I77, but not the control strain *S*. Newport Chile 361 (Figure S8c). With the focus of the current study on the discovery of potential epitopes for broad‐spectrum protection, while further studies are needed to optimize the vaccine formulation, these results highlight its translation potential.

**FIGURE 7:**
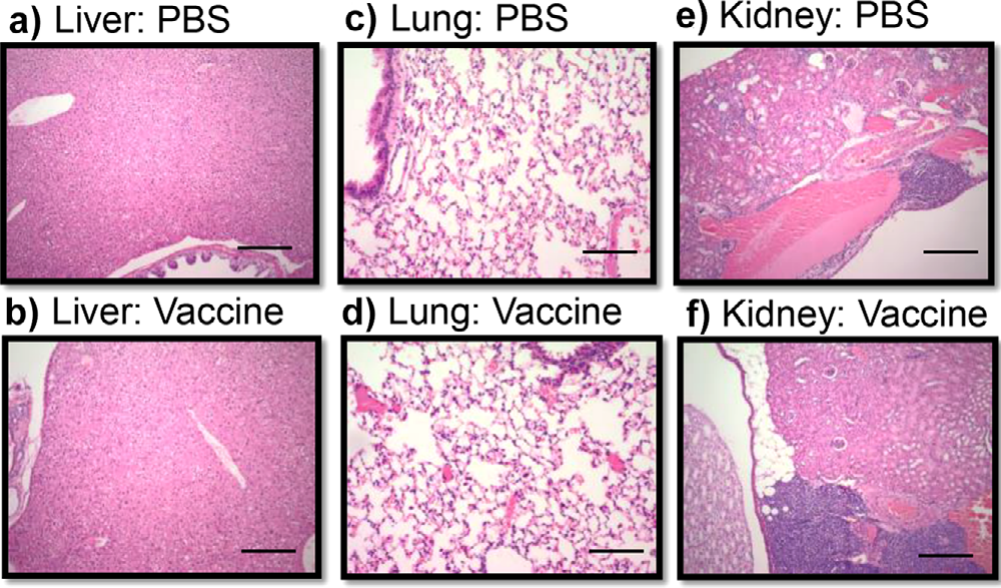
Histopathology images of extracted organs after mouse immunization. No significant differences were observed between the PBS control group (panels a, c, and e) and Qβ‐tri immunized group (panels b, d, and f). (a, b) liver tissue, (c, d), lung tissue, and (e, f) kidney tissue. The scale bar is 100 µm.

Since vaccines are generally administered prophylactically, it is important that they are biocompatible. Except for granuloma formation at injection sites, which was mainly attributed to complete Freund's adjuvant, there were no significant changes in mouse behavior or other adverse side effects observed in Qβ‐glycan conjugate vaccinated mice. On Day 35 after the prime immunization, the liver, lung, and kidney of mice immunized with the Qβ‐tri vaccine or PBS were extracted and subjected to histopathological analysis (Figure 7). No significant differences were observed between tissues from these two groups of mice, suggesting the biocompatibility of the vaccine.

Current glycoconjugate vaccine strategies against NTS have often relied on core‐ and O‐polysaccharides (COPS/O‐SP) as antigenic components, which are typically conjugated to carrier proteins such as bovine serum albumin (BSA), flagellin, tetanus toxoid, porin, or CRM197 to enhance immunogenicity [45, 46, 47, 48, 49, 50, 51]. For example, conjugates of *S*. Typhimurium COPS with homologous flagellin (FliC) or other protein carriers induce robust anti‐COPS responses but confer protection that is largely serovar‐specific, effectively protecting against *S*. Typhimurium but not heterologous strains such as *S*. Enteritidis [51].

A common feature of many of these approaches is the reliance on polysaccharides derived from bacterial cultures, either as purified native materials or as digestion products. This introduces several practical and mechanistic challenges. The purification process requires stringent control to remove endotoxins, cytoplasmic components, nucleic acids, and other contaminants [52]. In addition, these polysaccharides are inherently heterogeneous, consisting of mixtures with variable chain lengths and structural compositions. Chemical conjugation in some vaccine designs occurs randomly at hydroxyl groups along the COPS backbone, further contributing to batch‐to‐batch variability. Such structural heterogeneity complicates the precise identification of protective epitopes and limits mechanistic understanding of the elicited immune responses.

As an alternative, synthetic oligosaccharides with well‐defined structures have been explored for vaccine development. For instance, a synthetic tetrasaccharide antigen derived from *S*. Enteritidis or *S*. Paratyphi A, when conjugated to bacteriophage Qβ, conferred complete protection in a passive transfer model [19, 20]. While this approach offers improved structural definition and control over antigen composition, it remains similarly serovar‐restricted, as the synthetic antigen is designed to mimic specific O‐antigen epitopes.

Collectively, these prior studies highlight two major limitations of current glycoconjugate vaccines: (i) the reliance, in part, on extracted polysaccharides with inherent structural heterogeneity, and (ii) the strong serovar specificity of O‐antigen–directed immune responses, which limits cross‐protection. A synthetic carbohydrate antigen‐based broad‐spectrum vaccine as reported in this work can help overcome these hurdles.

## 3 Conclusion

To explore the possibility of discovering a broad‐spectrum vaccine, we studied the shared O‐polysaccharide backbone of two major NTS species, that is, *S*. Enteritidis and *S*. Typhimurium. An effective trisaccharide building block‐based approach has been carried out, which enabled the stereoselective construction of O‐polysaccharide backbone tri‐, hexa‐, and nona‐saccharide corresponding to one to three repeating units. Suitable protective group and glycosylation chemistry were designed, producing the targets containing all α glycosyl linkages successfully.

The synthetic glycan antigens were covalently linked with a powerful carrier, the bacteriophage Qβ, using a bifunctional linker through amides. Upon immunization of both mice and rabbits, the conjugates were able to induce high levels of IgG antibodies against the glycan antigens. Similar levels of antibody responses were generated against the tri‐, hexa‐, and nona‐saccharides. Interestingly, the density of glycan on Qβ is critical for antibody responses, with 180 copies or more trisaccharide per particle leading to strong anti‐glycan IgG antibody responses.

The antisera from rabbits selectively recognized the native O‐polysaccharides from *S*. Enteritidis and *S*. Typhimurium. In addition, antibodies produced in immunized rabbits were able to bind *S*. Enteritidis and *S*. Typhimurium bacteria and promote their phagocytosis by human macrophages. Passive immunization of the mice with antisera from Qβ‐trisaccharide immunized rabbits provided effective protection against lethal challenges with bloodstream isolates of *S*. Enteritidis and *S*. Typhimurium. This is the first report that a single glycan antigen derived from the O‐polysaccharide backbone can provide protection against two major NTS species. The observation that a trisaccharide unit was sufficient to induce high levels of protective antibodies highlights it as a promising direction to develop synthetic antigen‐based anti‐*Salmonella* vaccines.

## Author Contributions

**Xingling Pan**: investigation, writing – original draft, writing – review and editing, data curation. **Changxin Huo**: investigation, writing – original draft, data curation. **Soham Maity**: investigation. **Herbert Kavunja**: investigation. **Rachel Moszyk**: investigation. **Cameron Talbot**: investigation. **Scott M. Baliban**: writing – review and editing, formal analysis. **Xuefei Huang**: conceptualization, funding acquisition, writing – review and editing, supervision, project administration.

## Conflicts of Interest

S.M., H.K., and R.M. are employees of Iaso Therapeutics. X.H. is a founder of Iaso Therapeutics.

## Supporting information




**Supporting File**: Experimental procedures including synthesis, bioconjugation, immunization, in vitro analysis and bacterial challenges, as well as NMR spectra can be found at Supporting Information.

## Data Availability

The data that support the findings of this study are available in the Supporting Information of this article
