# Supplementary material for: Synthetic O‐Polysaccharide Backbone Units for a Single Antigen Vaccine Against Two Major Non‐Typhoidal Salmonella Serovars
Source: Angew Chem Int Ed Engl. 2026 May 7;65(26):e7740689. doi: 10.1002/anie.7740689 (PMC13285466; doi:10.1002/anie.7740689)
Supplement: Supplementary file 1 — Supporting File: Experimental procedures including synthesis, bioconjugation, immunization, in vitro analysis and bacterial challenges, as well as NMR spectra can be found at Supporting Information. [file ANIE-65-e7740689-s001.pdf]

Supporting Information

Synthetic O-Polysaccharide Backbone Units for a Single Antigen Vaccine against Two Major Non-typhoidal *Salmonella* Serovars

Xingling Pan,[a,b,#] Changxin Huo,[a,b,#] Soham Maity,[c] Herbert Kavunja,[c] Rachel Moszyk,[c] Cameron Talbot,[a,b] Scott M. Baliban,[d] Xuefei Huang[a,b,e*]

a. Department of Chemistry, Michigan State University; 578 South Shaw Lane, East Lansing, MI 48824, USA.

b. Institute for Quantitative Health Science & Engineering; Michigan State University; 578 South Shaw Lane, East Lansing, MI 48824, USA.

c. Iaso Therapeutics Inc., 4942 Dawn Avenue, East Lansing, MI 48823, USA.

d. Center for Vaccine Development and Global Health; University of Maryland School of Medicine; Baltimore; 685 W Baltimore St, Baltimore, MD 21201, USA.

e. Department of Biomedical Engineering; Michigan State University; East Lansing, MI 48824, USA.

[#]Equal contribution

*Email: huangxu2@msu.edu

Table of contents

Animal study ethics statement	**S4**
General experimental procedures	**S4**
Characterization of anomeric stereochemistry	**S4**
General procedure for pre-activation based single-step glycosylation:	**S4**
General procedures for protein sample preparation and analysis	**S5**
Synthesis of monosaccharide building blocks:	**S5**
Synthesis of *Salmonella* trisaccharide **1**	**S8**
Synthesis of *Salmonella* hexasaccharide **2**	**S12**
Synthesis of *Salmonella* nonasaccharide **3**	**S17**
Synthesis and characterization of Qβ-glycan conjugates	**S22**
**Scheme S1**: Synthesis and characterization of BSA-glycan conjugates	**S24**
Procedure for mouse immunization	**S24**
Procedure for rabbit immunization	**S25**
Bacterial strains and growth conditions	**S25**
Evaluation of antibody titers by ELISA	**S26**
Procedure for flow cytometry	**S26**
Procedure for opsonophagocytosis assay	**S26**
Passive immunization and lethal Salmonella challenge in mice	**S27**
Mouse tissue collection and processing for histopathology	**S28**
**Figure S1**. ESI-TOF HRMS mass spectra of representative Qβ-tri conjugates	**S28**
**Figure S2**. ESI-TOF HRMS mass spectra of representative Qβ-hexa conjugates	**S31**
**Figure S3**. ESI-TOF HRMS mass spectra of representative Qβ-nona conjugates	**S32**
**Figure S4**. MALDI-TOF mass spectra of BSA-tri conjugate	**S33**
**Figure S5**. MALDI-TOF mass spectra of BSA-hexa conjugate	**S34**
**Figure S6**. MALDI-TOF mass spectra of BSA-nona conjugate	**S35**
**Figure S7.** Antibody responses to alum-adjuvanted Qβ-tri conjugate vaccine	**S36**
**Table S1**. Calculation of vaccine efficacy	**S36**
**References**	**S37**
**NMR spectra**	
**1**	**S38**
**2**	**S41**
**3**	**S43**
**6**	**S45**
**7**	**S47**
**11**	**S49**
**13**	**S52**
**14**	**S54**
**15**	**S56**

**16**	**S58**
**17**	**S60**
**18**	**S62**
**19**	**S65**
**20**	**S67**
**21**	**S69**
**22**	**S71**
**23**	**S73**
**24**	**S75**
**25**	**S77**
**1’**	**S79**
**2’**	**S79**
**3’**	**S80**
**S1**	**S81**
**S2**	**S83**
**S3**	**S85**
**S4**	**S87**

Animal study ethics statement

All animal care procedures and experimental protocols have been approved by the Institutional Animal Care and Use Committee (IACUC) of Michigan State University (**protocol number:** 202200444) and complied with all relevant institutional and national guidelines.

General experimental procedures:

All reactions were carried out with anhydrous solvents in oven-dried glassware, unless otherwise noted. Glycosylation reactions were performed in the presence of 4 Å molecular sieves, which were flame–dried right before the reaction under high vacuum. Glycosylation solvents were dried using a solvent purification system and used directly without further drying. The chemicals used were reagent grade as supplied, except where noted. Analytical thin-layer chromatography (TLC) was performed using silica gel 60 F254 glass plates. Compounds spots were visualized by UV light (254 nm) and by staining with a yellow solution containing $Ce(NH_4)_2(NO_3)_6$ (0.5 g) and $(NH_4)_6Mo_7O_{24}\cdot 4H_2O$ (24.0 g) in 6% H_2SO_4 (500 mL). Flash column chromatography was performed on silica gel 60 (230-400 Mesh).

NMR spectra were referenced using residual $CHCl_3$ (δ ^{1}H-NMR 7.26 ppm) and $CDCl_3$ (δ ^{13}C-NMR 77.0 ppm). Peak and coupling constants assignments are based on ^{1}H-NMR, ^{1}H-^{1}H gCOSY and ^{1}H-^{13}C gHSQC and ^{1}H-^{13}C gHMBC experiments. Optical rotations were recorded on a Perkin Elemer 341 Polarimeter (λ= 589 nm, 1 dm cell).

Characterization of anomeric stereochemistry:

The stereochemistry of the newly formed glycosidic linkages in the oligosaccharide and intermediates is determined by $^3J_{(H1,H2)}$ through ^{1}H-NMR and/or $^1J_{(C1,H1)}$ through gHSQC 2-D NMR (without ^{1}H decoupling). For galactosyl building blocks, the smaller coupling constants of $^3J_{(H1,H2)}$ (around 3 Hz) indicate α linkages and larger coupling constants $^3J_{(H1,H2)}$ (7.5 Hz or larger) indicate β linkages. For all glycosyl linkages, the stereochemistry can be further confirmed via larger $^1J_{(C1,H1)}$ (around 170 Hz) suggests α linkages and smaller $^1J_{(C1,H1)}$ (around 160 Hz) for β linkages.

General procedure for pre-activation based single-step glycosylation:

A solution of donor (60 µmol) and freshly activated molecular sieve MS 4Å (200 mg) in CH_2Cl_2 (DCM) (2 mL) was stirred for 30 minutes at room temperature, and then cooled to -78 °C. A solution of AgOTf (47 mg, 180 µmol) in anhydrous Et_2O/DCM (0.8 mL/0.2 mL) was added to reaction solution without touching the wall of the flask. After 5 min., orange colored *p*-TolSCl (9.5 µL, 60 µmol) was added to the reaction mixture through a microsyringe. *p*-TolSCl should be added directly to the reaction solution to

prevent it from freezing on the flask wall. The characteristic orange color of *p*-TolSCl in the reaction solution disappeared rapidly in a few seconds indicating depletion of *p*-TolSCl. After the donor was completely activated according to TLC analysis (about 5 minutes), a solution of acceptor (60 μmol) with one equivalent of TTBP in DCM (1.2 mL) was slowly added along the wall of the flask via a syringe. The reaction was warmed up to -20 °C under stirring in 2 h. Upon reaction completion, the reaction mixture was quenched by Et_3N and filtered over Celite. The Celite was washed with DCM till no organic compounds were observed in the filtrate by TLC. After removal of the solvent, the desired oligosaccharide was purified by silica gel flash chromatography.

General procedures for protein sample preparation and analysis

All chemicals were reagent grade and were used as received from the manufacturer, unless otherwise noted. Centrifugal filter units of 10, 000 and 100, 000 molecular weight cut-off (MWCO) were purchased from EMD Millipore. ESI-TOF LC-MS analysis was performed on each purified viral nanoparticle sample (1 μL, 1 mg/mL) after denaturing (Waters Xevo G2-XS UPLC/MS/MS). MALDI-TOF MS analysis was performed on each purified BSA sample (10 μL, 1 mg/mL) after denaturing and desalting the sample using Cleanup C18 Pipette Tips (Agilent Technologies). The mixture (0.6 μL) and matrix solution (0.6 μL, 10 mg/mL sinapic acid in 50/50/0.1 CH_3CN/H_2O/TFA was spotted on a MALDI plate, air-dried, and analyzed by MALDI-TOF mass spectrometry (Applied Biosystems Voyager DE STR). Protein concentration was measured using the Coomassie Plus Protein Reagent (Bradford Assay, Pierce) with bovine serum albumin (BSA) as the standard.

Synthesis of monosaccharide building blocks:

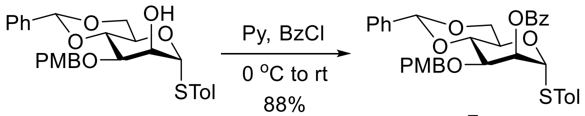

10 **7**

***p*-Tolyl 2-*O*-benzoyl-4,6-*O*-benzylidene-3-*O*-(4-methoxybenzyl)-1-thio-*α*-D-mannopyranoside (7)**: Compound **10**[3] (2.39 g, 4.83 mmol) was dissolved in pyridine (23 mL), cooled to 0 °C and BzCl (0.9 mL, 7.73 mmol) was added dropwise. After 10 h, the solution was diluted with DCM and washed with 1 M HCl, dried over Na_2SO_4, and filtered. The filtrate was concentrated *in vacuo* and the residue was purified by flash chromatography (hexanes/EtOAc, 20:1 → 4:1) to afford **7** (2.28 g, 88%) as a white foam. $[\alpha]_D^{20}$ +67.4° (*c* 1.0, DCM); ^{1}H NMR (500 MHz, $CDCl_3$) δ 8.11 – 8.06 (m, 2H, Ar-H), 7.60 – 7.56 (m, 1H, Ar-H), 7.55 – 7.51 (m, 2H, Ar-H), 7.49 – 7.43 (m, 2H, Ar-H), 7.42 – 7.34 (m, 5H, Ar-H), 7.28 – 7.25 (m, 2H, Ar-H), 7.16 – 7.11 (m, 2H, Ar-H), 6.84 – 6.78 (m, 2H, Ar-H), 5.81 (dd, *J* = 3.4, 1.5 Hz, 1H, H-2), 5.69 (s, 1H, PhCH),

5.53 (d, J = 1.5 Hz, 1H, H-1), 4.70 (d, J = 11.7 Hz, 1H, CH_2Ar), 4.64 (d, J = 11.7 Hz, 1H, CH_2Ar), 4.44 (td, J = 9.8, 4.8 Hz, 1H, H-5), 4.29 – 4.21 (m, 2H, H-4, H-6a), 4.11 (dd, J = 9.9, 3.4 Hz, 1H, H-3), 3.91 (t, J = 10.3 Hz, 1H, H-6b), 3.78 (s, 3H, OCH_3), 2.33 (s, 3H, $SPhCH_3$); ^{13}C NMR (125 MHz, $CDCl_3$) δ 165.64, 159.24, 138.39, 137.37, 133.35, 132.76, 130.00, 129.91, 129.72, 129.64, 129.44, 129.19, 128.97, 128.43, 128.19, 126.16, 113.76, 101.67, 87.56, 78.77, 73.76, 71.83, 71.78, 68.55, 65.16, 55.24, 21.16; HRMS (ESI) Anal. Calcd for $C_{35}H_{34}O_7S$ $[M+Na]^+$: 616.2363, found 616.2371.

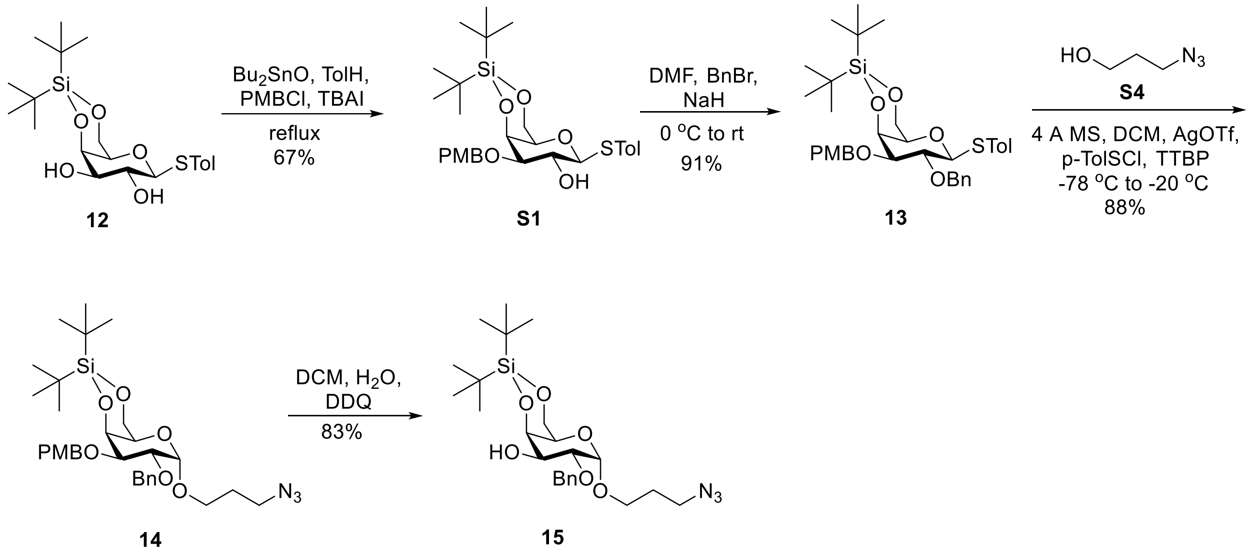

***p*-Tolyl 4,6-*O*-di-*tert*-butylsilylidene-3-*O*-(4-methoxybenzyl)-1-thio-*β*-D-galactopyranoside (S1):** nBu_2SnO (1.13 g, 4.52 mmol) was added to a solution of galactose diol **12**[1] (1.755 g, 4.11 mmol) in toluene (49 mL) and the resulting mixture was refluxed with removal of H_2O using a Dean Stark apparatus for 3 h. The reaction mixture was cooled to 25 °C and PMBCl (0.84 mL, 6.17 mmol) and TBAI (303.3 mg, 0.82 mmol) were added. The reaction mixture was refluxed again for 5 h, and then the reaction mixture was quenched by the addition of H_2O. The solvents were removed under reduced pressure and the residue was purified by flash chromatography (hexanes/EtOAc, 10:1 → 4:1) to afford **S1** (1.51 g, 67%) as a light-yellow oil. $[\alpha]_D^{20}$ -1.5° (*c* 1.0, DCM); ^1H NMR (500 MHz, $CDCl_3$) δ 7.50 – 7.44 (m, 2H, Ar-H), 7.36 – 7.30 (m, 2H, Ar-H), 7.14 – 7.07 (m, 2H, Ar-H), 6.94 – 6.85 (m, 2H, Ar-H), 4.75 (d, J = 11.3 Hz, 1H, CH_2Ar), 4.59 – 4.52 (m, 2H, H-4, CH_2Ar), 4.51 (d, J = 9.6 Hz, 1H, H-1), 4.25 (dd, J = 12.4, 1.6 Hz, 1H, H-6a), 4.21 (dd, J = 12.4, 2.3 Hz, 1H, H-6b), 3.98 (t, J = 9.6 Hz, 1H, H-2), 3.81 (s, 3H, OCH_3), 3.41 – 3.25 (m, 2H, H-3, H-5), 2.56 (brs, 1H, OH), 2.33 (s, 3H, $SPhCH_3$), 1.07 (s, 9H, $C(CH_3)_3$), 1.06 (s, 9H, $C(CH_3)_3$); ^{13}C NMR (125 MHz, $CDCl_3$) δ 159.37, 137.99, 133.34, 129.96, 129.59, 129.51, 113.96, 89.42, 81.61, 75.20, 69.93, 69.22, 68.37, 67.46, 55.30, 27.63, 27.52, 23.41, 21.15, 20.63; HRMS (ESI) Anal. Calcd for $C_{29}H_{42}O_6SSi$ $[M+H]^+$: 547.2544, found 547.2549.

***p*-Tolyl 2-*O*-benzyl-4,6-*O*-di-*tert*-butylsilylidene-3-*O*-(4-methoxybenzyl)-1-thio-*β*-D-galactopyranoside (13):** To a solution of **S1** (798 mg, 1.46 mmol) in DMF (2 mL) were added BnBr (0.35 mL, 2.92 mmol) and NaH (60% oil dispersion, 175 mg, 4.38 mmol) at 0 ºC under an atmosphere of argon. After stirring for 15 min at 0 ºC, the mixture was stirred for 15 min at room temperature, diluted with EtOAc, and quenched with ice water. The organic layer was washed with brine, dried over Na_2SO_4, and filtered. The filtrate was concentrated *in vacuo* and the residue was purified by flash chromatography (hexanes/EtOAc, 20:1 → 10:1) to afford **13** (905 mg, 91%) as a colorless oil. $[\alpha]_D^{20}$ +25.5º (*c* 1.0, DCM); ^{1}H NMR (500 MHz, $CDCl_3$) δ 7.48 – 7.41 (m, 4H, Ar-H), 7.38 – 7.29 (m, 5H, Ar-H), 7.11 – 7.05 (m, 2H, Ar-H), 6.88 – 6.82 (m, 2H, Ar-H), 4.90 (d, *J* = 10.3 Hz, 1H, CH_2Ar), 4.88 (d, *J* = 10.3 Hz, 1H, CH_2Ar), 4.71 (d, *J* = 11.6 Hz, 1H, CH_2Ar), 4.63 (d, *J* = 11.6 Hz, 1H, CH_2Ar), 4.58 (d, *J* = 9.7 Hz, 1H, H-1), 4.45 (d, *J* = 3.0 Hz, 1H, H-4), 4.20 (dd, *J* = 12.3, 1.6 Hz, 1H, H-6a), 4.16 (dd, *J* = 12.3, 2.3 Hz, 1H, H-6b), 3.81 (t, *J* = 9.7 Hz, 1H, H-2), 3.80 (s, 3H, OCH_3), 3.44 (dd, *J* = 9.7, 3.0 Hz, 1H, H-3), 3.24 – 3.23 (m, 1H, H-5), 2.32 (s, 3H, $SPhCH_3$), 1.13 (s, 9H, $C(CH_3)_3$), 1.08 (s, 9H, $C(CH_3)_3$); ^{13}C NMR (125 MHz, $CDCl_3$) δ 159.22, 138.45, 137.48, 132.82, 130.94, 130.41, 129.47, 129.39, 128.43, 128.28, 127.68, 113.81, 89.04, 82.52, 77.26, 75.88, 74.72, 70.69, 69.99, 67.39, 55.27, 27.68, 27.64, 23.42, 21.12, 20.72; HRMS (ESI) Anal. Calcd for $C_{36}H_{48}O_6SSi$ $[M+NH_4]^+$: 654.3279, found 654.3299.

3-Azidopropyl 2-*O*-benzyl-4,6-*O*-di-*tert*-butylsilylidene-3-*O*-(4-methoxybenzyl)-*α*-D-galactopyranoside (14): Compound **14** was synthesized from donor **13** and acceptor 3-azido-propanol **S4**[2] as a colorless oil in 88% yield following the general procedure of single-step glycosylation. $[\alpha]_D^{20}$ +81.1º (*c* 1.0, DCM); ^{1}H NMR (500 MHz, $CDCl_3$) δ 7.41 – 7.27 (m, 7H, Ar-H), 6.91 – 6.84 (m, 2H, Ar-H), 4.87 (d, *J* = 11.9 Hz, 1H, CH_2Ar), 4.74 – 4.59 (m, 4H, H-1, CH_2Ar), 4.49 (d, *J* = 3.0 Hz, 1H, H-4), 4.21 (dd, *J* = 12.4, 2.1 Hz, 1H, H-6a), 4.10 (dd, *J* = 12.4, 1.7 Hz, 1H, H-6b), 3.97 (dd, *J* = 10.0, 3.7 Hz, 1H, H-2), 3.81 (s, 3H, OCH_3), 3.78 (dd, *J* = 10.0, 3.0 Hz, 1H, H-3), 3.71 (ddd, *J* = 10.2, 7.3, 5.4 Hz, 1H, O*H*CHCH_2CH_2N$_3$), 3.58 – 3.57 (m, 1H, H-5), 3.47 (dt, *J* = 10.2, 5.9 Hz, 1H, OHC*H*CH_2CH_2N$_3$), 3.42 – 3.34 (m, 2H, $OCH_2CH_2C\mathit{H}_2N_3$), 1.97 – 1.76 (m, 2H, $OCH_2C\mathit{H}_2CH_2N_3$), 1.06 (s, 9H, $C(CH_3)_3$), 0.99 (s, 9H, $C(CH_3)_3$); ^{13}C NMR (125 MHz, $CDCl_3$) δ 159.07, 138.65, 131.00, 129.18, 128.34, 128.17, 127.67, 113.73, 98.26, 77.37, 74.30, 73.76, 71.10, 70.63, 67.36, 67.19, 64.62, 55.28, 48.32, 28.78, 27.67, 27.31, 23.44, 20.66; HRMS (ESI) Anal. Calcd for $C_{32}H_{47}N_3O_7Si$ $[M+NH_4]^+$: 631.3522, found 631.3538.

3-Azidopropyl 2-*O*-benzyl-4,6-*O*-di-*tert*-butylsilylidene-*α*-D-galactopyranoside (15): To a solution of **14** (606 mg, 0.99 mmol) in DCM–H_2O (22 mL, 10:1) was added DDQ (631 mg, 2.96 mmol), and the solution was stirred for 2 h at room temperature, then diluted with DCM. The solution was washed with saturated aqueous $NaHCO_3$ and brine, dried over Na_2SO_4, and filtered. The filtrate was concentrated *in*

vacuo and the residue was purified by flash chromatography (hexanes/EtOAc, 4:1 → 2:1) to afford **15** (402 mg, 83%) as a colorless oil. $[\alpha]_D^{20}$ +90.9° (*c* 1.0, DCM); ^{1}H NMR (500 MHz, $CDCl_3$) δ 7.42 – 7.27 (m, 5H, Ar-H), 4.82 (d, *J* = 12.1 Hz, 1H, CH_2Ar), 4.74 (d, *J* = 3.5 Hz, 1H, H-1), 4.68 (d, *J* = 12.1 Hz, 1H, CH_2Ar), 4.44 (dd, *J* = 3.5, 1.3 Hz, 1H, H-4), 4.26 (dd, *J* = 12.6, 2.1 Hz, 1H, H-6a), 4.12 (dd, *J* = 12.6, 1.8 Hz, 1H, H-6b), 3.94 (dd, *J* = 9.8, 3.4 Hz, 1H, H-3), 3.74 – 3.64 (m, 3H, H-2, H-5, O*H*CHCH$_2$CH$_2$N$_3$), 3.44 – 3.36 (m, 3H, OHC*H*CH$_2$C*H*$_2$N$_3$), 2.49 (brs, 1H, OH), 1.92 – 1.78 (m, 2H, OCH$_2$C*H*$_2$CH$_2$N$_3$), 1.04 (s, 9H, $C(CH_3)_3$), 0.96 (s, 9H, $C(CH_3)_3$); ^{13}C NMR (125 MHz, $CDCl_3$) δ 138.15, 128.46, 128.22, 127.91, 97.74, 75.79, 73.56, 73.12, 69.79, 67.05, 66.86, 64.62, 48.20, 28.78, 27.54, 27.18, 23.34, 20.62; HRMS (ESI) Anal. Calcd for $C_{24}H_{39}N_3O_6Si$ $[M+Na]^+$: 516.2500, found 516.2512.

Synthesis of *Salmonella* trisaccharide 1

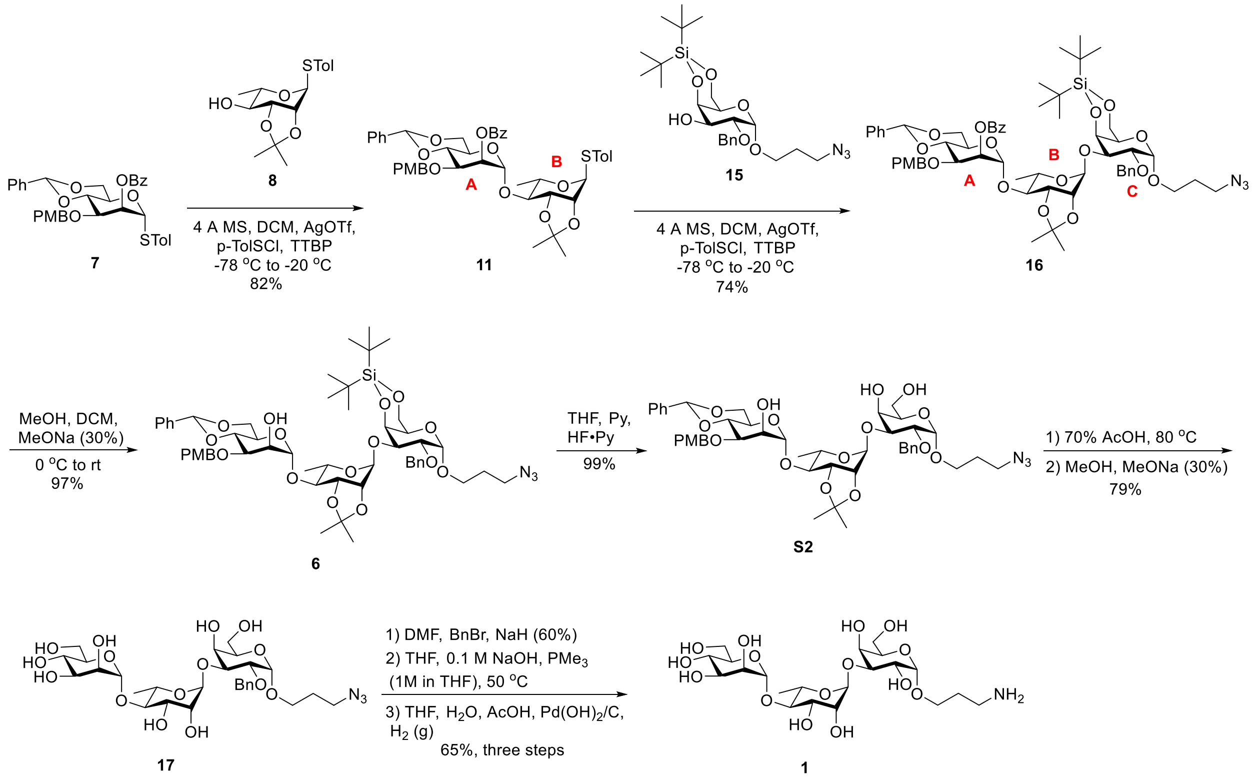

***p*-Tolyl 2-*O*-benzoyl-4,6-*O*-benzylidene-3-*O*-(4-methoxybenzyl)-*α*-D-mannopyranosyl-(1→4)-2,3-*O*-isopropylidene-*α*-L-rhamnopyranoside (11):** Compound **11** was synthesized from donor **7** and acceptor **8**[4] as a white foam in 82% yield following the general procedure of single-step glycosylation. $[\alpha]_D^{20}$ -118.7° (*c* 1.0, DCM); ^{1}H NMR (500 MHz, $CDCl_3$) δ 8.14 – 8.08 (m, 2H, Ar-H), 7.62 – 7.57 (m, 1H, Ar-H), 7.57 – 7.54 (m, 2H, Ar-H), 7.47 (m, 2H, Ar-H), 7.44 – 7.35 (m, 5H, Ar-H), 7.29 – 7.25 (m, 2H, Ar-H), 7.15 (m, 2H, Ar-H), 6.83 – 6.78 (m, 2H, Ar-H), 5.70 (s, 1H, PhCH), 5.68 (s, 1H, H-1B), 5.54 (dd, *J* = 3.5, 1.6 Hz,

1H, H-2A), 5.03 (d, *J* = 1.6 Hz, 1H, H-1A), 4.70 (d, *J* = 11.9 Hz, 1H, CH_2Ar), 4.66 (d, *J* = 11.9 Hz, 1H, CH_2Ar), 4.34 (d, *J* = 5.4 Hz, 1H, H-2B), 4.33 – 4.29 (m, 1H, H-5A), 4.24 – 4.16 (m, 4H, H-4A, H-5B, H-6A), 4.15 – 4.10 (m, 1H, H-3A), 3.91 – 3.84 (m, 1H, H-3B), 3.78 (s, 3H, OCH_3), 3.45 (dd, *J* = 10.1, 7.5 Hz, 1H, H-4B), 2.35 (s, 3H, $SPhCH_3$), 1.52 (s, 3H, $C(CH_3)_2$), 1.36 (s, 3H, $C(CH_3)_2$), 1.27 (d, *J* = 6.3 Hz, 3H, H-6B); ^{13}C NMR (125 MHz, $CDCl_3$) δ 165.92, 159.14, 138.00, 137.52, 133.36, 132.52, 130.08, 129.95, 129.89, 129.62, 129.39, 129.22, 128.90, 128.42, 128.17, 126.18, 113.71, 109.42, 101.60, 99.32, 84.07, 81.43, 78.60, 76.61, 76.44, 73.52, 71.73, 70.38, 68.83, 66.20, 63.95, 55.24, 28.16, 26.56, 21.16, 17.28; HRMS (ESI) Anal. Calcd for $C_{44}H_{48}O_{11}S$ $[M+H]^+$: 785.2990, found 785.3004.

3-Azidopropyl 2-*O*-benzoyl-4,6-*O*-benzylidene-3-*O*-(4-methoxybenzyl)-*α*-D-mannopyranosyl-(1→4)-2,3-*O*-isopropylidene-*α*-L-rhamnopyranosyl-(1→3)-2-*O*-benzyl-4,6-*O*-di-*tert*-butylsilylidene-*α*-D-galactopyranoside (16): Compound **16** was synthesized from donor **11** and acceptor **15** as a white foam in 74% yield following the general procedure of single-step glycosylation. $[\alpha]_D^{20}$ +44.1° (*c* 1.0, DCM); ^1H NMR (500 MHz, $CDCl_3$) δ 8.15 – 8.08 (m, 2H, Ar-H), 7.63 – 7.57 (m, 1H, Ar-H), 7.57 – 7.52 (m, 2H, Ar-H), 7.48 (m, 2H, Ar-H), 7.43 – 7.28 (m, 8H, Ar-H), 7.26 – 7.23 (m, 2H, Ar-H), 6.82 – 6.76 (m, 2H, Ar-H), 5.70 (s, 1H, PhCH), 5.50 (dd, *J* = 3.5, 1.6 Hz, 1H, H-2A), 5.25 (s, 1H, H-1B), 5.04 (d, *J* = 1.5 Hz, 1H, H-1A), 4.74 (d, *J* = 11.9 Hz, 1H, CH_2Ar), 4.72 (s, 1H, H-1C), 4.68 (d, *J* = 12.0 Hz, 1H, CH_2Ar), 4.63 – 4.60 (m, 2H, CH_2Ar), 4.42 (s, 1H, H-4C), 4.32 (dd, *J* = 10.0, 3.8 Hz, 1H, H-5A), 4.25 (dd, *J* = 12.6, 2.0 Hz, 1H, H-6aC), 4.21 – 4.16 (m, 4H, H-6A, H-2B, H-3B), 4.12 (dd, *J* = 12.5, 2.0 Hz, 1H H-6bC), 4.10 – 4.07 (m, 1H, H-3A), 4.04 – 3.99 (m, 1H, H-5B), 3.94 – 3.93 (m, 2H, H-2C, H-3C), 3.91 – 3.84 (m, 1H, H-4A), 3.77 (s, 3H, OCH_3), 3.76 – 3.71 (m, 1H, $O\mathit{H}CHCH_2CH_2N_3$), 3.69 – 3.67 (m, 1H, H-5C), 3.51 – 3.34 (m, 4H, H-4B, $OHC\mathit{H}CH_2C\mathit{H}_2N_3$), 1.93 – 1.80 (m, 2H, $OCH_2C\mathit{H}_2CH_2N_3$), 1.51 (s, 3H, $C(CH_3)_2$), 1.33 (s, 3H, $C(CH_3)_2$), 1.31 (d, *J* = 6.3 Hz, 3H, H-6B), 1.07 (s, 9H, $C(CH_3)_3$), 1.02 (s, 9H, $C(CH_3)_3$); ^{13}C NMR (125 MHz, $CDCl_3$) δ 165.95, 159.12, 138.20, 137.57, 133.37, 130.03, 129.94, 129.63, 129.12, 128.89, 128.45, 128.43, 128.15, 128.07, 127.91, 126.19, 113.70, 109.18, 101.60, 99.35, 99.25, 97.80, 81.58, 78.59, 77.92, 76.51, 75.94, 74.36, 73.98, 73.42, 71.50, 70.30, 68.85, 67.25, 66.99, 64.91, 64.70, 63.89, 55.19, 48.23, 28.79, 28.17, 27.48, 27.39, 26.50, 23.41, 20.74, 17.80; HRMS (ESI) Anal. Calcd for $C_{61}H_{79}N_3O_{17}Si$ $[M+NH_4]^+$: 1171.5517, found 1171.5518.

3-Azidopropyl 4,6-*O*-benzylidene-3-*O*-(4-methoxybenzyl)-*α*-D-mannopyranosyl-(1→4)-2,3-*O*-isopropylidene-*α*-L-rhamnopyranosyl-(1→3)-2-*O*-benzyl-4,6-*O*-di-*tert*-butylsilylidene-*α*-D-galactopyranoside (6): Compound **16** (105 mg, 0.091 mol) was dissolved in MeOH/DCM (6 mL, 2:1). The solution was cooled to 0 °C and NaOMe (cat.) was added. After stirring overnight at room temperature, the mixture was neutralized with DOWEX-H^+ ion exchange resins, filtered and concentrated. The residue

was purified by flash chromatography (hexanes/EtOAc, 2:1 → 3:2) to afford **6** (93 mg, 97%) as a colorless oil. $[\alpha]_D^{20}$ +88.4° (*c* 1.0, DCM); ^{1}H NMR (500 MHz, $CDCl_3$) δ 7.56 – 7.51 (m, 2H, Ar-H), 7.41 – 7.27 (m, 10H, Ar-H), 6.90 – 6.84 (m, 2H, Ar-H), 5.63 (s, 1H, PhCH), 5.24 (s, 1H, H-1B), 4.94 (d, *J* = 1.3 Hz, 1H, H-1A), 4.79 (d, *J* = 11.5 Hz, 1H, CH_2Ar), 4.74 (d, *J* = 11.9 Hz, 1H, CH_2Ar), 4.71 (s, 1H, H-1C), 4.67 (d, *J* = 11.5 Hz, 1H, CH_2Ar), 4.61 (d, *J* = 11.9 Hz, 1H, CH_2Ar), 4.41 – 4.39 (m, 1H, H-4C), 4.33 – 4.28 (m, 1H, H-5A), 4.23 (dd, *J* = 12.5, 1.9 Hz, 1H, H-6aC), 4.19 (d, *J* = 5.4 Hz, 1H, H-2B), 4.16 – 4.09 (m, 4H, H-3B, H-4A, H-6aA, H-6bC), 4.03 (dd, *J* = 3.6, 1.4 Hz, 1H, H-2A), 4.01 – 3.93 (m, 1H, H-5B), 3.94 – 3.91 (m, 2H, H-2C, H-3C), 3.89 (dd, *J* = 9.0, 3.6 Hz, 1H, H-3A), 3.85 – 3.83 (m, 1H, H-6bA), 3.81 (s, 3H, OCH_3), 3.76 – 3.71 (m, 1H, O*H*$CHCH_2CH_2N_3$), 3.67 (s, 1H, H-5C), 3.50 – 3.34 (m, 4H, H-4B, OHC*H*CH_2C*H*$_2N_3$), 2.70 (brs, 1H, OH), 1.93 – 1.80 (m, 2H, OCH_2C*H*$_2CH_2N_3$), 1.52 (s, 3H, $C(CH_3)_2$), 1.33 (s, 3H, $C(CH_3)_2$), 1.24 (d, *J* = 6.3 Hz, 3H, H-6B), 1.04 (s, 9H, $C(CH_3)_3$), 1.02 (s, 9H, $C(CH_3)_3$); ^{13}C NMR (125 MHz, $CDCl_3$) δ 159.37, 138.20, 137.68, 130.03, 129.43, 128.88, 128.45, 128.20, 128.07, 127.91, 126.11, 113.87, 109.14, 101.53, 100.62, 99.40, 97.82, 81.01, 78.83, 77.92, 76.61, 75.94, 75.04, 74.29, 74.00, 73.42, 72.55, 69.97, 68.91, 67.23, 67.00, 64.97, 64.70, 63.25, 55.24, 48.22, 28.78, 28.17, 27.49, 27.39, 26.54, 23.39, 20.74, 17.90; HRMS (ESI) Anal. Calcd for $C_{54}H_{75}N_3O_{16}Si$ $[M+NH_4]^+$: 1067.5255, found 1067.5251.

3-Azidopropyl 4,6-*O*-benzylidene-3-*O*-(4-methoxybenzyl)-*α*-D-mannopyranosyl-(1→4)-2,3-*O*-isopropylidene-*α*-L-rhamnopyranosyl-(1→3)-2-*O*-benzyl-*α*-D-galactopyranoside (S2): To a solution of **6** (22 mg, 0.021 mmol) in dry THF (2 mL) were added pyridine (17.5 mg, 0.23 mmol) and HF·pyridine (70% HF in pyridine, 8.4 mg, 0.29 mmol), and the solution was stirred for 1 h at room temperature. The reaction mixture was diluted with DCM, washed with saturated aqueous $NaHCO_3$, dried over Na_2SO_4, and filtered. The filtrate was concentrated *in vacuo* and the residue was purified by flash chromatography (DCM/MeOH, 40:1) to afford **S2** (19 mg, 99%) as a colorless oil. $[\alpha]_D^{20}$ +74.0° (*c* 1.0, DCM); ^{1}H NMR (500 MHz, $CDCl_3$) δ 7.55-7.49 (d, Ar-H), 7.42 – 7.29 (m, 9H, Ar-H), 7.27 – 7.25 (m, 1H, Ar-H), 6.87 (m, 2H, Ar-H), 5.62 (s, 1H, PhCH), 5.34 (s, 1H, H-1B), 4.92 (s, 1H, H-1A), 4.86 (d, *J* = 3.6 Hz, 1H, H-1C), 4.80 (d, *J* = 11.5 Hz, 1H, CH_2Ar), 4.71 – 4.56 (m, 3H, CH_2Ar), 4.28 (dd, *J* = 10.1, 3.5 Hz, 1H, H-5A), 4.19 – 4.00 (m, 8H, H-2B, H-3B, H-2A, H-4A), 3.92 – 3.72 (m, 10H, H-2C, H-5B, H-6A, OCH_3, O*H*$CHCH_2CH_2N_3$), 3.51 – 3.32 (m, 4H, H-4B, OHC*H*CH_2C*H*$_2N_3$), 2.78 (s, 1H, OH), 2.71 (s, 1H, OH), 2.34 (brs, 1H, OH), 1.94 – 1.81 (m, 2H, OCH_2C*H*$_2CH_2N_3$), 1.51 (s, 3H, $C(CH_3)_2$), 1.33 (s, 3H, $C(CH_3)_2$), 1.25 (d, *J* = 6.2 Hz, 3H, H-6B); ^{13}C NMR (125 MHz, $CDCl_3$) δ 159.37, 138.02, 137.61, 130.04, 129.50, 128.88, 128.46, 128.21, 127.95, 127.85, 126.08, 113.86, 109.16, 101.50, 100.73, 99.02, 97.33, 80.36, 78.75, 76.48, 76.13, 75.93, 75.51, 75.14, 72.97, 72.68, 71.23, 69.91, 69.03, 68.86, 65.69, 64.83, 63.35, 63.16, 55.28, 48.20, 28.76, 28.05, 26.43, 17.52; HRMS (ESI) Anal. Calcd for $C_{46}H_{59}N_3O_{16}$ $[M+NH_4]^+$: 927.4234, found 927.4246.

3-Azidopropyl *α*-D-mannopyranosyl-(1→4)-*α*-L-rhamnopyranosyl-(1→3)-2-*O*-benzyl-*α*-D-galactopyranoside (17): A solution of **S2** (68 mg, 0.075 mmol) in acetic acid (70% aq., 11 mL) was stirred at 80 ºC for 4 h. The solvent was evaporated followed by co-evaporation with toluene. The residue was dissolved in MeOH (6 mL) and NaOMe (cat.) was added. After stirring 1 h at room temperature, the mixture was neutralized with DOWEX-H^+ ion exchange resins, filtered and concentrated. The residue was purified by flash chromatography (DCM/MeOH, 6:1 → 4:1) to afford **17** (39 mg, 79%) as a colorless oil. $[\boldsymbol{\alpha}]_{\mathbf{D}}^{\mathbf{20}}$ +51.8º (*c* 1.0, MeOH); ^{1}H NMR (500 MHz, D_2O) δ 7.34 – 7.24 (m, 5H, Ar-H), 4.85 (s, 1H), 4.82 (s, 1H), 4.77 (d6, *J* = 3.7 Hz, 1H, H-1C), 4.57 (d, *J* = 11.7 Hz, 1H, CH_2Ar), 4.50 (d, *J* = 11.6 Hz, 1H, CH_2Ar), 3.89 – 3.84 (m, 2H), 3.82 – 3.78 (m, 3H), 3.75 – 3.54 (m, 11H, H-2C, H-5B, O*H*CHCH$_2$CH$_2$N$_3$), 3.43 – 3.32 (m, 2H, OHC*H*CH$_2$CH$_2$N$_3$), 3.31 – 3.23 (m, 2H, OCH$_2$CH$_2$C*H*$_2$N$_3$), 1.80 – 1.67 (m, 2H, OCH$_2$C*H*$_2$CH$_2$N$_3$), 1.14 (d, *J* = 6.2 Hz, 3H, H-6B); ^{13}C NMR (125 MHz, D_2O) δ 137.26, 128.68, 128.64, 128.42, 101.57, 101.30, 96.36, 81.19, 75.88, 75.39, 73.08, 72.97, 70.66, 70.35, 70.28, 70.13, 68.96, 68.83, 67.94, 66.36, 64.81, 60.91, 60.52, 48.01, 27.77, 16.74; HRMS (ESI) Anal. Calcd for $C_{28}H_{43}N_3O_{15}$ $[M+NH_4]^+$: 684.2586, found 684.2610.

3-Aminopropyl *α*-D-mannopyranosyl-(1→4)-*α*-L-rhamnopyranosyl-(1→3)-*α*-D-galactopyranoside (1): To a solution of **17** (15 mg, 0.023 mmol) in DMF (2 mL) were added BnBr (0.043 mL, 0.36 mmol) and NaH (60% oil dispersion, 21.8 mg, 0.54 mmol). After stirring for 30 min at room temperature, the mixture was diluted with EtOAc, and quenched with ice water. The organic layer was washed with brine, dried over Na_2SO_4, and filtered. The filtrate was concentrated *in vacuo* and the residue was purified by flash chromatography (hexanes/EtOAc, 4:1 → 1:1) to afford perbenzylated trisaccharide (30 mg, 96%) as a colorless oil. The product was dissolved in dry THF (3.5 mL). To the solution were added 0.1 M NaOH (0.5 mL) and a 1 M solution of PMe_3 in THF (0.5 mL). The flask was then equipped with a reflux condenser and heated at 50 °C for 3 hours. The mixture was concentrated to dryness. The resulting residue was dissolved in DCM, washed with H_2O, dried over Na_2SO_4, and filtered. The filtrate was concentrated *in vacuo* and the residue was purified by flash chromatography (DCM/MeOH, 15:1 → 10:1) to afford amine (20 mg, 68%) as a colorless oil. The product and $Pd(OH)_2$/C (10%-20%, 20 mg) in THF/H_2O/AcOH (3 mL, 1:1:1) was stirred at room temperature under atmospheric pressure H_2 overnight. The mixture was filtered through Celite, concentrated, and extracted with DCM and EtOAc. The water layer was then lyophilized to afford **1** (8 mg, 100%) as an acetate salt as white foam. $[\boldsymbol{\alpha}]_{\mathbf{D}}^{\mathbf{20}}$ +35.3º (*c* 0.17, H_2O); ^{1}H NMR (500 MHz, D_2O) δ 4.86 (s, 1H), 4.84 – 4.81 (m, 2H), 3.94 – 3.51 (m, 16H, H-1A, H-1B, H-1C, H-5B, O*H*CHCH$_2$CH$_2$NH$_2$), 3.49 – 3.36 (m, 2H, OHC*H*CH$_2$CH$_2$NH$_2$), 3.08 – 2.93 (m, 2H, OCH$_2$CH$_2$C*H*$_2$NH$_2$), 1.92 – 1.80 (m, 2H, OCH$_2$C*H*$_2$CH$_2$NH$_2$), 1.75 (s, 3H, CH_3CO), 1.16 (d, *J* = 6.1 Hz, 3H, H-6B); ^{13}C NMR (125 MHz, D_2O) δ 101.90, 101.27, 98.33, 81.13, 77.35, 72.98, 70.98, 70.27, 70.24, 70.13, 68.96, 68.81,

67.98, 67.23, 66.36, 65.57, 61.03, 60.53, 37.57, 26.44, 16.76; HRMS (ESI) Anal. Calcd for $C_{21}H_{39}NO_{15}$ $[M+H]^+$: 546.2392, found 546.2391.

Synthesis of *Salmonella* hexasaccharide 2

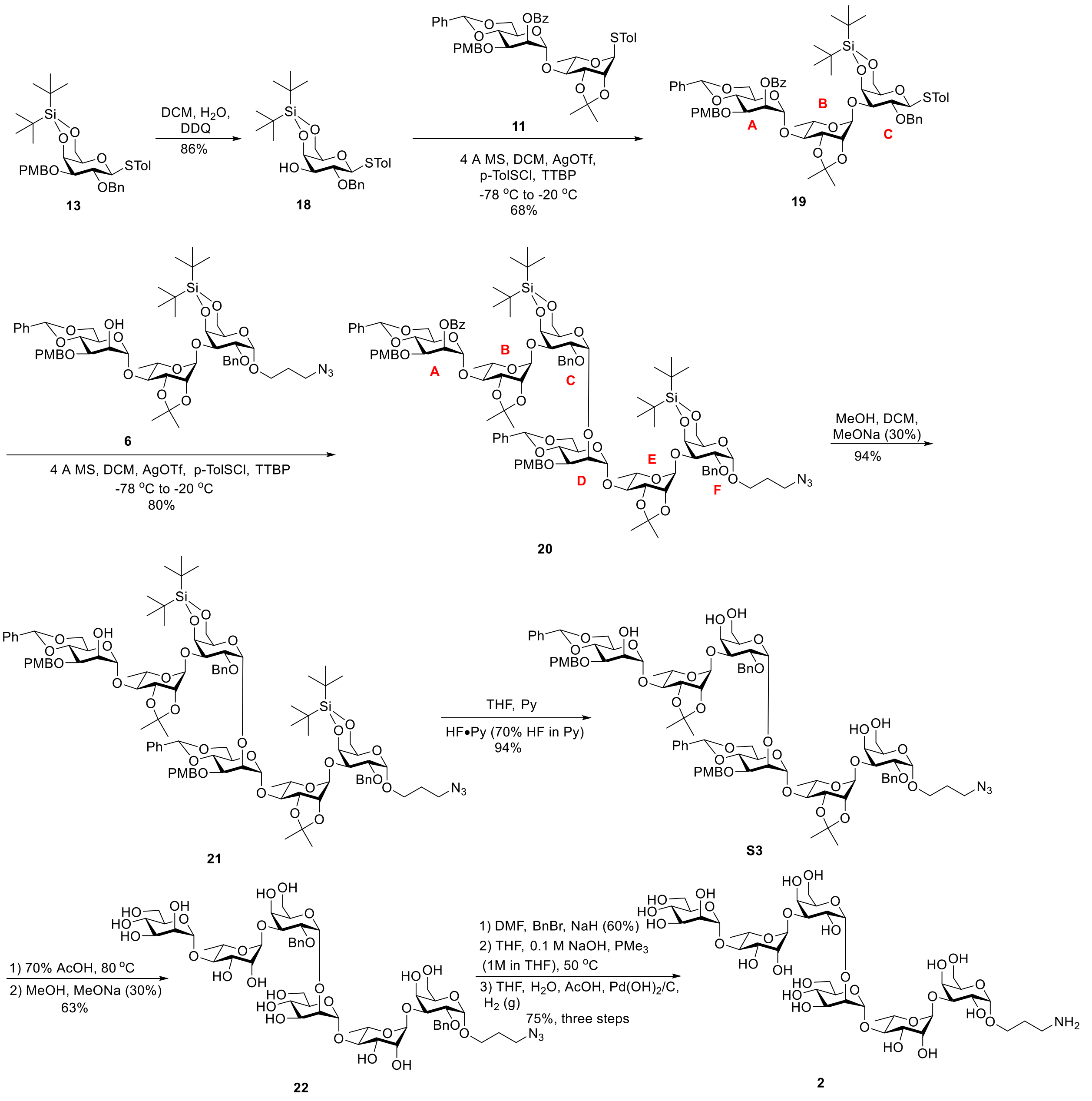

***p*-Tolyl 2-*O*-benzyl-4,6-*O*-di-*tert*-butylsilylidene-1-thio-*β*-D-galactopyranoside (18):** To a solution of 13 (98 mg, 0.15 mmol) in DCM–H_2O (5.5 mL, 10:1) was added DDQ (98 mg, 0.46 mmol), and the solution

was stirred for 2 h at room temperature, then diluted with DCM. The solution was washed with saturated aqueous $NaHCO_3$ and brine, dried over Na_2SO_4, and filtered. The filtrate was concentrated *in vacuo* and the residue was purified by flash chromatography (hexanes/EtOAc, 10:1 → 4:1) to afford **18** (69 mg, 86%) as a colorless oil. $[\alpha]_D^{20}$ -18.6° (*c* 1.0, DCM); ^{1}H NMR (500 MHz, $CDCl_3$) δ 7.52 – 7.48 (m, 2H, Ar-H), 7.47 – 7.44 (m, 2H, Ar-H), 7.39 – 7.34 (m, 2H, Ar-H), 7.33 – 7.29 (m, 1H, Ar-H), 7.13-7.07 (m, 2H, Ar-H), 4.94 (s, 2H, CH_2Ar), 4.57 (d, *J* = 9.7 Hz, 1H, H-1), 4.41 (dd, *J* = 3.5, 1.0 Hz, 1H, H-4), 4.22 (d, *J* = 1.9 Hz, 2H, H-6), 3.64 (dd, *J* = 8.7, 3.4 Hz, 1H, H-3), 3.56 (dd, *J* = 9.7, 8.8 Hz, 1H, H-2), 3.40 – 3.32 (m, 1H, H-5), 2.74 (brs, 1H, OH), 2.34 (s, 3H, $SPhCH_3$), 1.10 (s, 9H, $C(CH_3)_3$), 1.06 (s, 9H, $C(CH_3)_3$); ^{13}C NMR (125 MHz, $CDCl_3$) δ 138.09, 137.59, 132.92, 130.38, 129.42, 128.32, 128.24, 127.69, 88.29, 78.96, 75.49, 75.39, 74.62, 72.87, 66.96, 27.45, 27.43, 23.25, 21.05, 20.62; HRMS (ESI) Anal. Calcd for $C_{28}H_{40}O_5SSi$ $[M+Na]^+$: 539.2258, found 539.2255.

***p*-Tolyl 2-*O*-benzoyl-4,6-*O*-benzylidene-3-*O*-(4-methoxybenzyl)-*α*-D-mannopyranosyl-(1→4)-2,3-*O*-isopropylidene-*α*-L-rhamnopyranosyl-(1→3)-2-*O*-benzyl-4,6-*O*-di-*tert*-butylsilylidene-*β*-D-galactopyranoside (19):** Compound **19** was synthesized from donor **11** and acceptor **18** as a white foam in 68% yield following the general procedure of single-step glycosylation. $[\alpha]_D^{20}$ +17.1° (*c* 1.0, DCM); ^{1}H NMR (500 MHz, $CDCl_3$) δ 8.14 – 8.08 (m, 2H, Ar-H), 7.62 – 7.57 (m, 1H, Ar-H), 7.56 – 7.52 (m, 2H, Ar-H), 7.50 – 7.43 (m, 4H, Ar-H), 7.43 – 7.27 (m, 9H, Ar-H), 7.26-7.22 (m, 1H, Ar-H), 7.13-7.07 (m, 2H, Ar-H), 6.82 – 6.75 (m, 2H, Ar-H), 5.69 (s, 1H, PhCH), 5.49 (dd, *J* = 3.5, 1.6 Hz, 1H, H-2A), 5.23 (s, 1H, H-1B), 5.04 (d, *J* = 1.6 Hz, 1H, H-1A), 5.01 (d, *J* = 10.2 Hz, 1H, CH_2Ar), 4.73 (d, *J* = 10.2 Hz, 1H, CH_2Ar), 4.67 (d, *J* = 12.0 Hz, 1H, CH_2Ar), 4.64 – 4.58 (m, 2H, H-1C, CH_2Ar), 4.39 (d, *J* = 3.1 Hz, 1H, H-4C), 4.31 (dd, *J* = 10.1, 3.9 Hz, 1H, H-6aA), 4.25 – 4.14 (m, 6H, H-4A, H-2B, H-3B, H-6bA, H-6C), 4.08 (dd, *J* = 9.4, 3.6 Hz, 1H, H-3A), 4.04 – 3.97 (m, 1H, H-5B), 3.87 (t, *J* = 9.6 Hz, 1H, H-5A), 3.80 (t, *J* = 9.5 Hz, 1H, H-2C), 3.76 (s, 3H, OCH_3), 3.59 (dd, *J* = 9.3, 3.1 Hz, 1H, H-3C), 3.42 – 3.33 (m, 2H, H-4B, H-5C), 2.33 (s, 3H, $SPhCH_3$), 1.51 (s, 3H, $C(CH_3)_2$), 1.31 (s, 3H, $C(CH_3)_2$), 1.29 (d, *J* = 6.3 Hz, 3H, H-6B), 1.15 (s, 9H, $C(CH_3)_3$), 1.08 (s, 9H, $C(CH_3)_3$); ^{13}C NMR (126 MHz, $CDCl_3$) δ 165.95, 159.12, 137.88, 137.65, 137.55, 133.37, 132.72, 130.79, 130.00, 129.93, 129.61, 129.57, 129.12, 128.90, 128.47, 128.43, 128.23, 128.15, 127.89, 126.18, 113.70, 109.22, 101.60, 99.72, 99.23, 89.22, 83.14, 81.47, 78.57, 77.37, 76.50, 76.09, 75.93, 74.65, 73.36, 73.30, 71.51, 70.32, 68.83, 67.15, 65.02, 63.91, 55.18, 28.13, 27.76, 27.44, 26.44, 23.39, 21.14, 20.77, 17.75; HRMS (ESI) Anal. Calcd for $C_{65}H_{80}O_{16}SSi$ $[M+Na]^+$: 1199.4829, found 1199.4835.

3-Azidopropyl 2-*O*-benzoyl-4,6-*O*-benzylidene-3-*O*-(4-methoxybenzyl)-*α*-D-mannopyranosyl-(1→4)-2,3-*O*-isopropylidene-*α*-L-rhamnopyranosyl-(1→3)-2-*O*-benzyl-4,6-*O*-di-*tert*-butylsilylidene-*α*-D-galactopyranosyl-(1→2)-4,6-*O*-benzylidene-3-*O*-(4-methoxybenzyl)-*α*-D-mannopyranosyl-(1→4)-

2,3-*O*-isopropylidene-*α*-L-rhamnopyranosyl-(1→3)-2-*O*-benzyl-4,6-*O*-di-*tert*-butylsilylidene-*α*-D-galactopyranoside (20): Compound **20** was synthesized from donor **19** and acceptor **6** as a white foam in 80% yield following the general procedure of single-step glycosylation. $[\alpha]_D^{20}$ +62.2° (*c* 1.0, DCM); ^{1}H NMR (500 MHz, $CDCl_3$) δ 8.16 – 8.10 (m, 2H, Ar-H), 7.63 – 7.58 (m, 1H, Ar-H), 7.58 – 7.46 (m, 6H, Ar-H), 7.45 – 7.27 (m, 13H, Ar-H), 7.26 – 7.15 (m, 7H, Ar-H), 6.85 – 6.76 (m, 4H, Ar-H), 5.71 (s, 1H, PhCH), 5.54 (d, *J* = 3.7 Hz, 1H, H-1C), 5.52 (dd, *J* = 3.5, 1.5 Hz, 1H, H-2A), 5.48 (s, 1H, PhCH), 5.29 – 5.26 (m, 2H, H-1B, H-1E), 5.06 (d, *J* = 1.5 Hz, 1H, H-1A), 4.83 (d, *J* = 11.3 Hz, 1H, CH_2Ar), 4.80 (s, 1H, H-1D), 4.77 – 4.60 (m, 8H, CH_2Ar, H-1F), 4.43 (d, *J* = 3.1 Hz, 1H), 4.41 (s, 1H), 4.36 – 4.30 (m, 2H), 4.29 – 3.66 (m, 32H, H-2C, H-3A, H-2B, H-2E, H-2D, H-2F, H-5B, H-5E, OCH_3, O*H*CHCH$_2$CH$_2$N$_3$), 3.53 – 3.31 (m, 5H, OHC*H*CH$_2$C*H*$_2$N$_3$), 1.95 – 1.80 (m, 2H, OHCHC*H*$_2$CH$_2$N$_3$), 1.52 (s, 6H, $C(CH_3)_2$), 1.37 – 1.29 (m, 9H, $C(CH_3)_2$), 1.19 (d, *J* = 6.3 Hz, 3H), 1.11 – 1.06 (m, 18H, $C(CH_3)_3$), 1.04 (s, 9H, $C(CH_3)_3$), 1.03 (s, 9H, $C(CH_3)_3$); ^{13}C NMR (125 MHz, $CDCl_3$) δ 165.96, 159.17, 159.13, 138.55, 138.18, 137.90, 137.60, 133.37, 130.58, 130.05, 129.96, 129.65, 129.44, 129.14, 128.89, 128.79, 128.46, 128.44, 128.17, 128.15, 128.08, 128.07, 127.92, 127.89, 127.38, 126.21, 126.12, 113.71, 109.14, 109.08, 101.60, 101.24, 100.85, 99.39, 99.37, 99.24, 98.01, 97.78, 81.63, 81.15, 79.16, 78.60, 77.76, 76.53, 76.50, 76.14, 75.98, 75.91, 74.44, 74.30, 73.98, 73.92, 73.74, 73.41, 73.37, 73.34, 71.51, 71.31, 70.33, 68.86, 68.67, 67.62, 67.30, 67.02, 66.91, 64.97, 64.86, 64.71, 64.48, 63.89, 55.21, 55.19, 48.23, 28.80, 28.21, 28.17, 27.56, 27.51, 27.40, 27.35, 26.49, 26.46, 23.44, 23.42, 20.74, 20.71, 17.74, 17.48; HRMS (ESI) Anal. Calcd for $C_{112}H_{147}N_3O_{32}Si_2$ $[M+NH_4]^+$: 2119.9844, found 2119.9927.

3-Azidopropyl 4,6-*O*-benzylidene-3-*O*-(4-methoxybenzyl)-*α*-D-mannopyranosyl-(1→4)-2,3-*O*-isopropylidene-*α*-L-rhamnopyranosyl-(1→3)-2-*O*-benzyl-4,6-*O*-di-*tert*-butylsilylidene-*α*-D-galactopyranosyl-(1→2)-4,6-*O*-benzylidene-3-*O*-(4-methoxybenzyl)-*α*-D-mannopyranosyl-(1→4)-2,3-*O*-isopropylidene-*α*-L-rhamnopyranosyl-(1→3)-2-*O*-benzyl-4,6-*O*-di-*tert*-butylsilylidene-*α*-D-galactopyranoside (21): Compound **20** (131 mg, 0.062 mol) was dissolved in MeOH/DCM (3 mL, 2:1). The solution was cooled to 0 °C and NaOMe (cat.) was added. After stirring 3 h at room temperature, the mixture was neutralized with DOWEX-H$^+$ ion exchange resins, filtered and concentrated. The residue was purified by flash chromatography (hexanes/EtOAc, 2:1 → 3:2) to afford **21** (116 mg, 94%) as a white foam. $[\alpha]_D^{20}$ +80.3° (*c* 1.0, DCM); ^{1}H NMR (500 MHz, $CDCl_3$) δ 7.55 – 7.48 (m, 4H, Ar-H), 7.45 – 7.26 (m, 14H, Ar-H), 7.26 – 7.12 (m, 6H, Ar-H), 6.90 – 6.84 (m, 2H, Ar-H), 6.83 – 6.78 (m, 2H, Ar-H), 5.62 (s, 1H, PhCH), 5.52 (d, *J* = 3.7 Hz, 1H, H-1C), 5.47 (s, 1H, PhCH), 5.27 – 5.23 (m, 2H, H-1B, H-1E), 4.94 (s, 1H, H-1A), 4.84 – 4.76 (m, 3H, CH_2Ar, H-1D), 4.75 – 4.58 (m, 6H, CH_2Ar, H-1F), 4.41 – 4.37 (m, 2H), 4.34 – 4.00 (m, 17H, H-2B, H-2E, H-2A, H-2D), 3.98 – 3.66 (m, 19H, H-2C, H-2F, H-5B, H-5E, OCH_3, O*H*CHCH$_2$CH$_2$N$_3$), 3.51 – 3.29 (m, 5H, OHC*H*CH$_2$C*H*$_2$N$_3$), 2.70 (brs, 1H, OH), 1.95 – 1.78 (m, 2H,

OHCHCH_2CH_2N_3), 1.52 (s, 3H, $C(CH_3)_2$), 1.50 (s, 3H, $C(CH_3)_2$), 1.34 – 1.30 (m, 6H, $C(CH_3)_2$), 1.23 (d, J = 6.3 Hz, 3H), 1.17 (d, J = 6.3 Hz, 3H), 1.06 (s, 9H, $C(CH_3)_3$), 1.03 (s, 9H, $C(CH_3)_3$), 1.02 (s, 18H, $C(CH_3)_3$); ^{13}C NMR (126 MHz, $CDCl_3$) δ 159.36, 159.16, 138.52, 138.16, 137.88, 137.69, 130.56, 130.04, 129.42, 128.87, 128.78, 128.44, 128.19, 128.16, 128.06, 128.06, 127.91, 127.87, 127.36, 126.11, 113.86, 113.70, 109.13, 109.02, 101.52, 101.23, 100.85, 100.59, 99.41, 99.38, 98.01, 97.76, 81.15, 81.04, 79.14, 78.82, 77.73, 76.62, 76.48, 76.13, 75.97, 75.90, 75.05, 74.42, 74.30, 73.97, 73.83, 73.74, 73.35, 73.33, 72.55, 71.30, 69.97, 68.91, 68.65, 67.58, 67.29, 67.01, 66.90, 64.95, 64.90, 64.70, 64.48, 63.23, 55.24, 55.20, 48.22, 28.78, 28.19, 28.15, 27.54, 27.50, 27.38, 27.34, 26.48, 23.43, 23.38, 20.73, 20.70, 17.81, 17.46; HRMS (ESI) Anal. Calcd for $C_{105}H_{143}N_3O_{31}Si_2$ $[M+NH_4]^+$: 2015.9582, found 2015.9696.

3-Azidopropyl 4,6-*O*-benzylidene-3-*O*-(4-methoxybenzyl)-*α*-D-mannopyranosyl-(1→4)-2,3-*O*-isopropylidene-*α*-L-rhamnopyranosyl-(1→3)-2-*O*-benzyl-*α*-D-galactopyranosyl-(1→2)-4,6-*O*-benzylidene-3-*O*-(4-methoxybenzyl)-*α*-D-mannopyranosyl-(1→4)-2,3-*O*-isopropylidene-*α*-L-rhamnopyranosyl-(1→3)-2-*O*-benzyl-*α*-D-galactopyranoside (S3): To a solution of **21** (133 mg, 0.067 mmol) in dry THF (6.7 mL) were added pyridine (55.5 mg, 0.73 mmol) and HF·pyridine (70% HF in pyridine, 26.6 mg, 0.93 mmol), and the solution was stirred for 1 h at room temperature. The reaction mixture was diluted with DCM, washed with saturated aqueous $NaHCO_3$, dried over Na_2SO_4, and filtered. The filtrate was concentrated *in vacuo* and the residue was purified by flash chromatography (DCM/MeOH, 40:1 → 20:1) to afford **S3** (108 mg, 94%) as a white foam. $[\alpha]_D^{20}$ +68.2° (c 1.0, DCM); ^{1}H NMR (500 MHz, $CDCl_3$) δ 7.57 – 7.50 (m, 4H, Ar-H), 7.47 – 7.28 (m, 13H, Ar-H), 7.26 – 7.25 (m, 2H, Ar-H), 7.20 – 7.10 (m, 5H, Ar-H), 6.88 (d, J = 8.5 Hz, 2H, Ar-H), 6.82 (d, J = 8.5 Hz, 2H, Ar-H), 5.67 (d, J = 3.8 Hz, 1H, H-1C), 5.63 (s, 1H, PhCH), 5.47 (s, 1H, PhCH), 5.41 (s, 1H), 5.35 (s, 1H), 4.97 – 4.84 (m, 4H, CH_2Ar, H-1A, H-1D, H-1F), 4.80 (d, J = 11.5 Hz, 1H, CH_2Ar), 4.73 – 4.58 (m, 6H, CH_2Ar), 4.37 – 3.98 (m, 20H, H-2B, H-2E), 3.97 – 3.67 (m, 17H, H-2C, H-5B, H-5E, OCH_3, O*H*CHCH_2CH_2N_3), 3.52 – 3.32 (m, 5H, OHC*H*CH_2C*H_2*N_3), 2.82 (s, 1H, OH), 2.79 (s, 1H, OH), 2.60 (s, 1H, OH), 2.53 – 2.42 (m, 2H, OH), 1.96 – 1.83 (m, 2H, OHCHC*H_2*CH_2N_3), 1.56 – 1.48 (m, 6H, $C(CH_3)_2$), 1.37 – 1.32 (m, 6H, $C(CH_3)_2$), 1.29 – 1.22 (m, 6H, H-6B, H-6E); ^{13}C NMR (125 MHz, $CDCl_3$) δ 159.37, 159.22, 138.17, 138.03, 137.74, 137.61, 130.47, 130.04, 129.57, 129.50, 128.89, 128.83, 128.47, 128.22, 128.21, 128.11, 127.96, 127.93, 127.86, 127.51, 126.09, 126.04, 113.86, 113.75, 109.20, 109.07, 101.51, 101.24, 100.72, 100.51, 99.05, 98.93, 97.37, 97.35, 80.47, 79.93, 79.22, 78.77, 76.49, 76.47, 76.28, 76.18, 75.89, 75.82, 75.53, 75.44, 75.14, 74.69, 74.36, 73.84, 72.98, 72.68, 71.14, 71.12, 71.11, 69.92, 69.52, 69.04, 68.86, 68.64, 65.66, 65.54, 64.84, 64.59, 63.34, 63.16, 63.05, 55.28, 55.24, 48.21, 28.76, 28.06, 28.01, 26.42, 26.35, 17.57, 17.53; HRMS (ESI) Anal. Calcd for $C_{89}H_{111}N_3O_{31}$ $[M+NH_4]^+$: 1735.7540, found 1735.7568.

3-Azidopropyl *α*-D-mannopyranosyl-(1→4)-*α*-L-rhamnopyranosyl-(1→3)-2-*O*-benzyl-*α*-D-galactopyranosyl-(1→2)-*α*-D-mannopyranosyl-(1→4)-*α*-L-rhamnopyranosyl-(1→3)-2-*O*-benzyl-*α*-D-galactopyranoside (22): A solution of **S3** (108 mg, 0.063 mmol) in acetic acid (70% aq., 9.1 mL) was stirred at 80 ºC for 12 h. The solvent was evaporated followed by co-evaporation with toluene. The residue was dissolved in MeOH (5 mL) and NaOMe (cat.) was added. After stirring 1 h at room temperature, the mixture was neutralized with DOWEX-H^+ ion exchange resins, filtered and concentrated. The residue was purified by flash chromatography (DCM/MeOH, 3:1 → 2:1) to afford **22** (48 mg, 63%) as a white foam. $[\alpha]_D^{20}$ +56.4º (*c* 1.0, MeOH); ^1H NMR (500 MHz, D_2O) δ 7.36 – 7.23 (m, 10H, Ar-H), 5.10 (s, 1H, H-1A), 5.02 (d, *J* = 3.7 Hz, 1H, H-C), 4.87 – 4.81 (m, 3H, H-1B, H-1D, H-1E), 4.77 (d, *J* = 3.6 Hz, 1H, H-1F), 4.61 – 4.47 (m, 4H, CH_2Ar), 3.98 – 3.48 (m, 30H, H-2A, H-2B, H-2C, H-2D, H-2E, H-2F, H-5B, H-5E, O*H*CHCH$_2$CH$_2$N$_3$), 3.44 – 3.22 (m, 6H, OHC*H*CH$_2$C*H*$_2$N$_3$), 1.80 – 1.66 (m, 2H, OHCHC*H*$_2$CH$_2$N$_3$), 1.20 – 1.09 (m, 6H, H-6B, H-6E); ^{13}C NMR (125 MHz, D_2O) δ 137.29, 136.86, 128.81, 128.76, 128.70, 128.64, 128.58, 128.43, 101.57, 101.51, 101.31, 99.52, 99.12, 96.38, 81.28, 81.21, 79.44, 75.95, 75.63, 75.60, 75.40, 73.28, 73.09, 72.99, 72.96, 71.14, 70.67, 70.42, 70.39, 70.37, 70.30, 70.15, 68.98, 68.92, 68.88, 68.84, 67.99, 67.90, 66.74, 66.38, 64.82, 60.93, 60.84, 60.55, 60.45, 48.03, 27.79, 16.91, 16.78; HRMS (ESI) Anal. Calcd for $C_{53}H_{79}N_3O_{29}$ $[M+NH_4]^+$: 1239.5137, found 1239.5093.

3-Aminopropyl *α*-D-mannopyranosyl-(1→4)-*α*-L-rhamnopyranosyl-(1→3)-*α*-D-galactopyranosyl-(1→2)-*α*-D-mannopyranosyl-(1→4)-*α*-L-rhamnopyranosyl-(1→3)-*α*-D-galactopyranoside (2): To a solution of **22** (48 mg, 0.039 mmol) in DMF (2 mL) were added BnBr (0.14 mL, 1.18 mmol) and NaH (60% oil dispersion, 70.9 mg, 1.77 mmol). After stirring for 30 min at room temperature, the mixture was diluted with EtOAc, and quenched with ice water. The organic layer was washed with brine, dried over Na_2SO_4, and filtered. The filtrate was concentrated *in vacuo* and the residue was purified by flash chromatography (hexanes/EtOAc, 3:1 → 2:1) to afford perbenzylated hexasaccharide (83 mg, 82%) as a colorless oil. The product was dissolved in dry THF (7 mL). To the solution were added 0.1 M NaOH (1 mL) and a 1 M solution of PMe_3 in THF (1 mL). The flask was then equipped with a reflux condenser and heated at 50 °C for 3 hours. The mixture was concentrated to dryness. The resulting residue was dissolved in DCM, washed with H_2O, dried over Na_2SO_4, and filtered. The filtrate was concentrated *in vacuo* and the residue was purified by flash chromatography (DCM/MeOH, 15:1 → 10:1) to afford amine (76 mg, 92%) as a colorless oil. The product and $Pd(OH)_2/C$ (10%-20%, 76 mg) in THF/H_2O/AcOH (4.8 mL, 1:1:1) were stirred at room temperature under atmospheric pressure H_2 overnight. The mixture was filtered through Celite, concentrated, and extracted with DCM and EtOAc. The water layer was then lyophilized to afford **2** (30 mg, 100%) as an acetate salt as a white foam. $[\alpha]_D^{20}$ +74.1º (*c* 0.39, H_2O); ^1H NMR (500 MHz, D_2O) δ 5.17 (s, 1H, H-1A), 5.02 (d, *J* = 3.3 Hz, 1H, H-1C), 4.91 – 4.80 (m, 4H, H-1B, H-1D, H-1E, H-1F), 4.04 – 3.34

(m, 34H, H-2A, H-2B, H-2C, H-2D, H-2E, H-2F, H-5B, H-5E, OC*H*$_2$CH$_2$CH$_2$NH$_2$), 3.08 – 2.92 (m, 2H, OCH$_2$CH$_2$C*H*$_2$NH$_2$), 1.92 – 1.79 (m, 2H, OHCHC*H*$_2$CH$_2$NH$_2$), 1.75 (s, 3H, CH$_3$CO), 1.24 – 1.08 (m, 6H, H-6B, H-6E); ^{13}C NMR (125 MHz, D$_2$O) δ 101.89, 101.81, 101.28, 101.21, 99.47, 98.33, 81.19, 81.13, 79.78, 77.38, 76.84, 72.97, 72.90, 71.31, 70.98, 70.28, 70.24, 70.15, 68.98, 68.96, 68.79, 67.99, 67.96, 67.24, 66.59, 66.37, 65.59, 61.05, 60.88, 60.53, 60.41, 37.59, 26.45, 16.91, 16.79; HRMS (ESI) Anal. Calcd for C$_{39}$H$_{69}$NO$_{29}$ [M+H]$^+$: 1016.4028, found 1016.3987.

Synthesis of *Salmonella* nonasaccharide 3

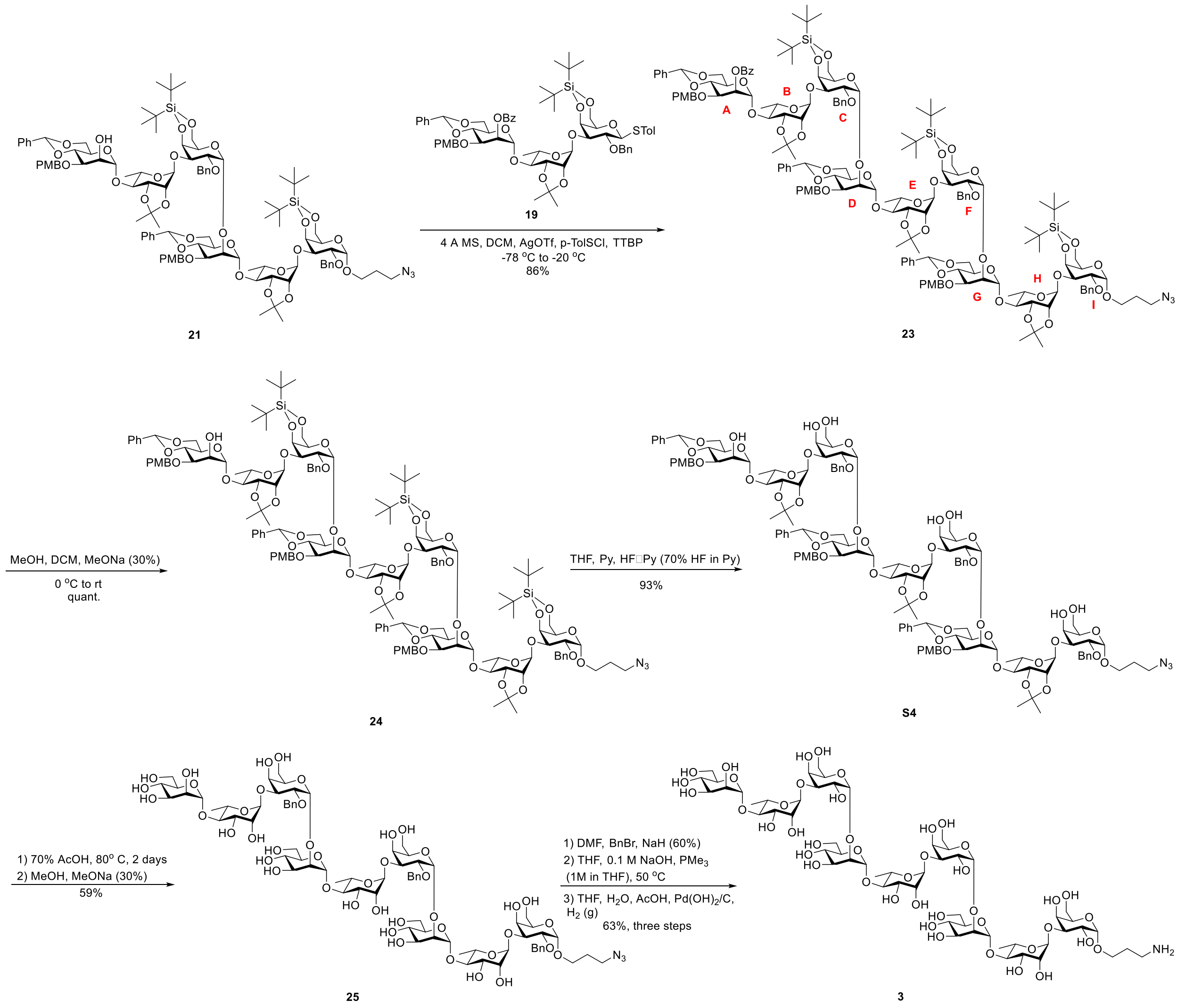

3-Azidopropyl 2-*O*-benzoyl-4,6-*O*-benzylidene-3-*O*-(4-methoxybenzyl)-*α*-D-mannopyranosyl-(1→4)-2,3-*O*-isopropylidene-*α*-L-rhamnopyranosyl-(1→3)-2-*O*-benzyl-4,6-*O*-di-*tert*-butylsilylidene-*α*-D-galactopyranosyl-(1→2)-4,6-*O*-benzylidene-3-*O*-(4-methoxybenzyl)-*α*-D-mannopyranosyl-(1→4)-2,3-*O*-isopropylidene-*α*-L-rhamnopyranosyl-(1→3)-2-*O*-benzyl-4,6-*O*-di-*tert*-butylsilylidene-*α*-D-

galactopyranosyl-(1→2)-4,6-*O*-benzylidene-3-*O*-(4-methoxybenzyl)-*α*-D-mannopyranosyl-(1→4)-2,3-*O*-isopropylidene-*α*-L-rhamnopyranosyl-(1→3)-2-*O*-benzyl-4,6-*O*-di-*tert*-butylsilylidene-*α*-D-galactopyranoside (23): Compound **23** was synthesized from donor **19** and acceptor **21** as a white foam in 86% yield following the general procedure of single-step glycosylation. $[\alpha]_D^{20}$ +64.6° (*c* 1.0, DCM); ^{1}H NMR (500 MHz, $CDCl_3$) δ 8.15 – 8.09 (m, 2H, Ar-H), 7.63 – 7.58 (m, 1H, Ar-H), 7.58 – 7.45 (m, 9H, Ar-H), 7.45 – 7.27 (m, 16H, Ar-H), 7.27 – 7.13 (m, 13H, Ar-H), 6.84 – 6.76 (m, 6H, Ar-H), 5.70 (s, 1H, PhCH), 5.55 – 5.52 (m, 2H, H-1C, H-1F), 5.51 (dd, *J* = 3.5, 1.5 Hz, 1H, H-2A), 5.48 – 5.45 (m, 2H, PhCH), 5.30 – 5.24 (m, 3H, H-1B, H-1E, H-1H), 5.05 (d, *J* = 1.5 Hz, 1H, H-1A), 4.86 – 4.77 (m, 4H, H-1D, H-1G, CH_2Ar), 4.76 – 4.59 (m, 10H, H-1I, CH_2Ar), 4.45 – 4.38 (m, 3H), 4.36 – 3.66 (m, 52H, H-2B, H-2C, H-2D, H-2E, H-2F, H-2G, H-2H, H-2I, H-3A, H-5B, H-5E, H-5H, OCH_3, O*H*CHC$H_2CH_2N_3$), 3.52 – 3.29 (m, 6H, OHC*H*CH_2C*H_2*N_3), 1.96 – 1.80 (m, 2H, OCH_2C*H_2*CH_2N_3), 1.54 – 1.47 (m, 9H, $C(CH_3)_2$), 1.36 – 1.29 (m, 12H, $C(CH_3)_2$), 1.21 – 1.15 (m, 6H), 1.11 – 1.06 (m, 27H, $C(CH_3)_3$), 1.05 – 0.99 (m, 27H, $C(CH_3)_3$); ^{13}C NMR (125 MHz, $CDCl_3$) δ 165.95, 159.17, 159.12, 138.55, 138.51, 138.17, 137.91, 137.88, 137.60, 133.37, 130.59, 130.55, 130.04, 129.96, 129.65, 129.44, 129.14, 128.89, 128.79, 128.78, 128.46, 128.43, 128.17, 128.16, 128.14, 128.07, 127.92, 127.89, 127.88, 127.36, 126.21, 126.12, 113.71, 109.14, 109.07, 109.01, 101.59, 101.24, 100.86, 100.84, 99.42, 99.39, 99.35, 99.24, 97.97, 97.77, 81.63, 81.16, 79.16, 78.59, 77.75, 77.15, 76.52, 76.49, 76.14, 75.98, 75.94, 75.90, 74.43, 74.27, 74.25, 73.98, 73.90, 73.73, 73.41, 73.37, 73.35, 71.50, 71.29, 71.24, 70.32, 68.86, 68.66, 67.65, 67.61, 67.30, 67.02, 66.92, 66.90, 64.96, 64.89, 64.85, 64.71, 64.49, 64.45, 63.88, 55.21, 55.19, 48.23, 28.80, 28.21, 28.19, 28.17, 27.57, 27.56, 27.51, 27.39, 27.35, 27.33, 26.48, 26.45, 26.44, 23.44, 23.41, 20.74, 20.70, 17.73, 17.47, 17.36; HRMS (ESI) Anal. Calcd for $C_{163}H_{215}N_3O_{47}Si_3$ $[M+2Na]^{2+}$: 1548.1809, found 1548.1771.

3-Azidopropyl 4,6-*O*-benzylidene-3-*O*-(4-methoxybenzyl)-*α*-D-mannopyranosyl-(1→4)-2,3-*O*-isopropylidene-*α*-L-rhamnopyranosyl-(1→3)-2-*O*-benzyl-4,6-*O*-di-*tert*-butylsilylidene-*α*-D-galactopyranosyl-(1→2)-4,6-*O*-benzylidene-3-*O*-(4-methoxybenzyl)-*α*-D-mannopyranosyl-(1→4)-2,3-*O*-isopropylidene-*α*-L-rhamnopyranosyl-(1→3)-2-*O*-benzyl-4,6-*O*-di-*tert*-butylsilylidene-*α*-D-galactopyranosyl-(1→2)-4,6-*O*-benzylidene-3-*O*-(4-methoxybenzyl)-*α*-D-mannopyranosyl-(1→4)-2,3-*O*-isopropylidene-*α*-L-rhamnopyranosyl-(1→3)-2-*O*-benzyl-4,6-*O*-di-*tert*-butylsilylidene-*α*-D-galactopyranoside (24): Compound **23** (129 mg, 0.042 mol) was dissolved in MeOH/DCM (3 mL, 2:1). The solution was cooled to 0 °C and NaOMe (cat.) was added. After stirring 3 h at room temperature, the mixture was neutralized with DOWEX-H^+ ion exchange resins, filtered and concentrated. The residue was purified by flash chromatography (hexanes/EtOAc, 3:2 → 1:1) to afford **24** (125 mg, 100%) as a white foam. $[\alpha]_D^{20}$ +83.1° (*c* 1.0, DCM); ^{1}H NMR (500 MHz, $CDCl_3$) δ 7.55 – 7.48 (m, 6H, Ar-H), 7.44 – 7.28 (m, 16H, Ar-H), 7.25 – 7.12 (m, 14H, Ar-H), 6.89 – 6.83 (m, 2H, Ar-H), 6.82 – 6.78 (m, 4H, Ar-H), 5.62

(s, 1H, PhCH), 5.55 – 5.49 (m, 2H, H-1C, H-1F), 5.46 (s, 1H, PhCH), 5.45 (s, 1H, PhCH), 5.30 – 5.21 (m, 3H, H-1B, H-1E, H-1H), 4.93 (s, 1H, H-1A), 4.84 – 4.57 (m, 14H, H-1D, H-1G, H-1I, CH_2Ar), 4.41 – 4.37 (m, 3H), 4.33 – 3.66 (m, 53H, H-2A, H-2B, H-2C, H-2D, H-2E, H-2F, H-2G, H-2H, H-2I, CH_2Ar, H-5B, H-5E, H-5H, OCH_3, O*H*$CHCH_2CH_2N_3$), 3.50 – 3.27 (m, 6H, OHC*H*CH_2C*H*$_2N_3$), 2.67 (brs, 1H, OH), 1.95 – 1.79 (m, 2H, OCH_2C*H*$_2CH_2N_3$), 1.53 – 1.46 (m, 9H, $C(CH_3)_2$), 1.36 – 1.28 (m, 9H, $C(CH_3)_2$), 1.23 (d, *J* = 6.3 Hz, 3H), 1.20 – 1.12 (m, 6H), 1.09 – 0.97 (m, 54H, $C(CH_3)_3$); ^{13}C NMR (125 MHz, $CDCl_3$) δ 159.37, 159.18, 159.17, 138.53, 138.49, 138.17, 137.90, 137.87, 137.70, 130.58, 130.54, 130.05, 129.44, 128.88, 128.80, 128.78, 128.46, 128.20, 128.17, 128.07, 127.93, 127.89, 127.37, 126.12, 113.87, 113.72, 113.71, 109.15, 109.03, 109.01, 101.54, 101.25, 100.86, 100.60, 99.40, 97.99, 97.97, 97.77, 81.17, 81.05, 79.15, 78.83, 77.74, 77.12, 76.63, 76.49, 76.14, 75.98, 75.95, 75.91, 75.07, 74.44, 74.26, 73.98, 73.83, 73.76, 73.72, 73.36, 72.56, 71.30, 71.26, 69.98, 68.92, 68.66, 67.66, 67.59, 67.30, 67.02, 66.92, 64.96, 64.90, 64.72, 64.50, 64.46, 63.24, 55.25, 55.21, 48.24, 28.80, 28.21, 28.19, 28.16, 27.57, 27.56, 27.52, 27.39, 27.34, 27.34, 26.49, 26.44, 23.44, 23.43, 23.40, 20.74, 20.71, 20.70, 17.82, 17.47, 17.36; HRMS (ESI) Anal. Calcd for $C_{156}H_{211}N_3O_{46}Si_3$ $[M+2NH_4]^{2+}$: 1491.2124, found 1491.2079.

3-Azidopropyl 4,6-*O*-benzylidene-3-*O*-(4-methoxybenzyl)-*α*-D-mannopyranosyl-(1→4)-2,3-*O*-isopropylidene-*α*-L-rhamnopyranosyl-(1→3)-2-*O*-benzyl-*α*-D-galactopyranosyl-(1→2)-4,6-*O*-benzylidene-3-*O*-(4-methoxybenzyl)-*α*-D-mannopyranosyl-(1→4)-2,3-*O*-isopropylidene-*α*-L-rhamnopyranosyl-(1→3)-2-*O*-benzyl-*α*-D-galactopyranosyl-(1→2)-4,6-*O*-benzylidene-3-*O*-(4-methoxybenzyl)-*α*-D-mannopyranosyl-(1→4)-2,3-*O*-isopropylidene-*α*-L-rhamnopyranosyl-(1→3)-2-*O*-benzyl-*α*-D-galactopyranoside (S5): To a solution of **24** (125 mg, 0.042 mmol) in dry THF (4.2 mL) were added pyridine (107.8 mg, 1.40 mmol) and HF·pyridine (70% HF in pyridine, 50.8 mg, 1.78 mmol), and the solution was stirred for 1 h at room temperature. The reaction mixture was diluted with DCM, washed with saturated aqueous $NaHCO_3$, dried over Na_2SO_4, and filtered. The filtrate was concentrated *in vacuo* and the residue was purified by flash chromatography (DCM/MeOH, 30:1 → 20:1) to afford **S5** (100 mg, 93%) as a white foam. $[\alpha]_D^{20}$ +63.2° (*c* 1.0, DCM); ^1H NMR (500 MHz, $CDCl_3$) δ 7.57 – 7.50 (m, 6H, Ar-H), 7.46 – 7.27 (m, 18H, Ar-H), 7.26 – 7.23 (m, 2H, Ar-H), 7.20 – 7.10 (m, 10H, Ar-H), 6.89 – 6.85 (m, 2H, Ar-H), 6.84 – 6.78 (m, 4H, Ar-H), 5.68 – 5.64 (m, 2H, H-1C, H-1F), 5.62 (s, 1H, PhCH), 5.49 – 5.45 (m, 2H, PhCH), 5.43 –5.35 (m, 3H, H-1B, H-1E, H-1H), 4.95 – 4.83 (m, 6H, H-1A, H-1D, H-1G, H-1I, CH_2Ar), 4.80 (d, *J* = 11.5 Hz, 1H, CH_2Ar), 4.74 – 4.57 (m, 8H, CH_2Ar), 4.32 – 3.97 (m, 29H, H-2A, H-2B, H-2D, H-2E, H-2G, H-2H, CH_2Ar), 3.96 – 3.69 (m, 27H, H-2C, H-2F, H-2I, H-5B, H-5E, H-5H, OCH_3, O*H*$CHCH_2CH_2N_3$), 3.52 – 3.33 (m, 6H, OHC*H*CH_2C*H*$_2N_3$), 2.84 (brs, 1H, OH), 2.80 (brs, 1H, OH), 2.67 (brs, 1H, OH), 2.61 (brs, 1H, OH), 2.58 – 2.42 (m, 3H, OH), 1.93 – 1.86 (m, 2H, OCH_2C*H*$_2CH_2N_3$), 1.55 – 1.48 (m, 9H, $C(CH_3)_2$), 1.37 – 1.31 (m, 9H, $C(CH_3)_2$), 1.30 – 1.21 (m, 9H, H-6B, H-6E, H-6H); ^{13}C NMR

(125 MHz, $CDCl_3$) δ 159.37, 159.22, 138.19, 138.05, 137.75, 137.62, 130.47, 130.05, 129.58, 129.51, 128.89, 128.84, 128.47, 128.21, 128.11, 127.96, 127.93, 127.86, 127.51, 126.09, 126.04, 113.87, 113.75, 109.20, 109.10, 109.07, 101.51, 101.25, 100.73, 100.50, 99.06, 98.93, 97.36, 80.48, 80.10, 79.95, 79.23, 78.77, 76.50, 76.47, 76.28, 76.20, 75.89, 75.83, 75.80, 75.55, 75.43, 75.15, 74.74, 74.70, 74.35, 73.84, 72.98, 72.68, 71.14, 71.11, 71.03, 69.92, 69.56, 69.50, 69.06, 68.87, 68.65, 65.66, 65.54, 65.51, 64.84, 64.59, 63.35, 63.18, 63.07, 63.04, 55.29, 55.24, 48.22, 28.77, 28.06, 28.02, 26.42, 26.36, 26.33, 17.57, 17.54; HRMS (ESI) Anal. Calcd for $C_{132}H_{163}N_3O_{46}$ $[M+2NH_4]^{2+}$: 1281.0592, found 1281.0562.

3-Azidopropyl *α*-D-mannopyranosyl-(1→4)-*α*-L-rhamnopyranosyl-(1→3)-2-*O*-benzyl-*α*-D-galactopyranosyl-(1→2)-*α*-D-mannopyranosyl-(1→4)-*α*-L-rhamnopyranosyl-(1→3)-2-*O*-benzyl-*α*-D-galactopyranosyl-(1→2)-*α*-D-mannopyranosyl-(1→4)-*α*-L-rhamnopyranosyl-(1→3)-2-*O*-benzyl-*α*-D-galactopyranoside (25): A solution of **S5** (80 mg, 0.032 mmol) in acetic acid (70% aq., 4.7 mL) was stirred at 80 °C for 48 h. The solvent was evaporated followed by co-evaporation with toluene. The residue was dissolved in MeOH (5 mL) and NaOMe (cat.) was added. After stirring 1 h at room temperature, the mixture was neutralized with DOWEX-H^+ ion exchange resins, filtered and concentrated. The residue was purified by flash chromatography (DCM/MeOH, 2:1 → 1:1) to afford **25** (33 mg, 59%) as a colorless oil. $[\alpha]_D^{20}$ +58.6° (*c* 1.0, MeOH); ^{1}H NMR (500 MHz, D_2O) δ 7.35 – 7.24 (m, 15H, Ar-H), 5.13 – 5.09 (m, 2H, H-1A, H-1D), 5.04 – 5.01 (m, 2H, H-1C, H-1F), 4.86 – 4.81 (m, 4H, H-1B, H-1E, H-1G, H-1H), 4.77 (d, J = 3.7 Hz, 1H, H-1I), 4.62 – 4.46 (m, 6H, CH_2Ar), 4.00 – 3.49 (m, 46H, H-2A, H-2B, H-2C, H-2D, H-2E, H-2F, H-2G, H-2H, H-2I, H-5B, H-5E, H-5H, O*H*$CHCH_2CH_2N_3$), 3.43 – 3.24 (m, 6H, OHC*H*CH_2C*H*$_2N_3$), 1.75 – 1.68 (m, 2H, OCH_2C*H*$_2CH_2N_3$), 1.18 – 1.08 (m, 9H, H-6B, H-6E, H-6H); ^{13}C NMR (125 MHz, D_2O) δ 137.29, 136.86, 128.81, 128.76, 128.70, 128.65, 128.58, 128.43, 101.58, 101.52, 101.31, 99.53, 99.12, 96.39, 81.28, 81.21, 79.44, 75.95, 75.67, 75.63, 75.60, 75.40, 73.29, 73.09, 72.99, 72.97, 71.14, 70.68, 70.42, 70.39, 70.29, 70.15, 68.98, 68.92, 68.87, 68.84, 67.99, 67.94, 67.90, 66.74, 66.38, 64.82, 60.93, 60.85, 60.55, 60.45, 48.03, 27.79, 16.93, 16.91, 16.78; HRMS (ESI) Anal. Calcd for $C_{78}H_{115}N_3O_{43}$ $[M+Na]^+$: 1804.6797, found 1804.6729.

3-Aminopropyl *α*-D-mannopyranosyl-(1→4)-*α*-L-rhamnopyranosyl-(1→3)-*α*-D-galactopyranosyl-(1→2)-*α*-D-mannopyranosyl-(1→4)-*α*-L-rhamnopyranosyl-(1→3)-*α*-D-galactopyranosyl-(1→2)-*α*-D-mannopyranosyl-(1→4)-*α*-L-rhamnopyranosyl-(1→3)-*α*-D-galactopyranoside (3): To a solution of **25** (33 mg, 0.019 mmol) in DMF (2 mL) were added BnBr (0.097 mL, 0.81 mmol) and NaH (60% oil dispersion, 49 mg, 1.22 mmol). After stirring for 30 min at room temperature, the mixture was diluted with EtOAc, and quenched with ice water. The organic layer was washed with brine, dried over Na_2SO_4, and filtered. The filtrate was concentrated *in vacuo* and the residue was purified by flash chromatography

(hexanes/EtOAc, 3:1 → 2:1) to afford the perbenzylated nonasaccharide (48 mg, 69%) as a colorless oil. The product was dissolved in dry THF (3.9 mL). To the solution were added 0.1 M NaOH (0.6 mL) and a 1 M solution of PMe_3 in THF (0.6 mL). The flask was then equipped with a reflux condenser and heated at 50 °C for 3 hours. The mixture was concentrated to dryness. The resulting residue was dissolved in DCM, washed with H_2O, dried over Na_2SO_4, and filtered. The filtrate was concentrated *in vacuo* and the residue was purified by flash chromatography (DCM/MeOH, 20:1 → 10:1) to afford amine (44 mg, 92%) as a colorless oil. The product and $Pd(OH)_2/C$ (10%-20%, 44 mg) in THF/H_2O/AcOH (3 mL, 1:1:1) was stirred at room temperature under atmospheric pressure H_2 overnight. The mixture was filtered through Celite, concentrated, and extracted with DCM and EtOAc. The water layer was then lyophilized to afford **3** (17 mg, 100%) in acetate form as a white foam. $[\alpha]_D^{20}$ +20.0º (*c* 0.21, H_2O); ^{1}H NMR (500 MHz, D_2O) δ 5.21 – 5.13 (m, 2H, H-1A, H-1D), 5.05 – 4.98 (m, 2H, H-1C, H-1F), 4.91 – 4.78 (m, 5H, H-1B, H-1E, H-1G, H-1H, H-1I), 4.00 – 3.34 (m, 50H, H-2A, H-2B, H-2C, H-2D, H-2E, H-2F, H-2G, H-2H, H-2I, H-5B, H-5E, H-5H, $OCH_2CH_2CH_2NH_2$), 3.06 – 2.93 (m, 2H, $OCH_2CH_2CH_2NH_2$), 1.92 – 1.81 (m, 2H, $OCH_2CH_2CH_2NH_2$), 1.75 (s, 3H, CH_3CO), 1.22 – 1.09 (m, 9H, H-6B, H-6E, H-6H); ^{13}C NMR (125 MHz, D_2O) δ 101.90, 101.82, 101.28, 101.21, 99.47, 98.34, 81.19, 81.14, 79.79, 79.78, 77.39, 76.89, 76.85, 72.97, 72.90, 71.32, 70.99, 70.28, 70.24, 70.15, 68.97, 68.80, 67.97, 67.24, 66.59, 66.38, 65.59, 61.04, 60.88, 60.88, 60.53, 60.40, 37.58, 26.45, 16.91, 16.78; HRMS (ESI) Anal. Calcd for $C_{57}H_{99}NO_{43}$ $[M+H]^+$: 1486.5664, found 1486.5616.

Synthesis and characterization of Qβ-glycan conjugates

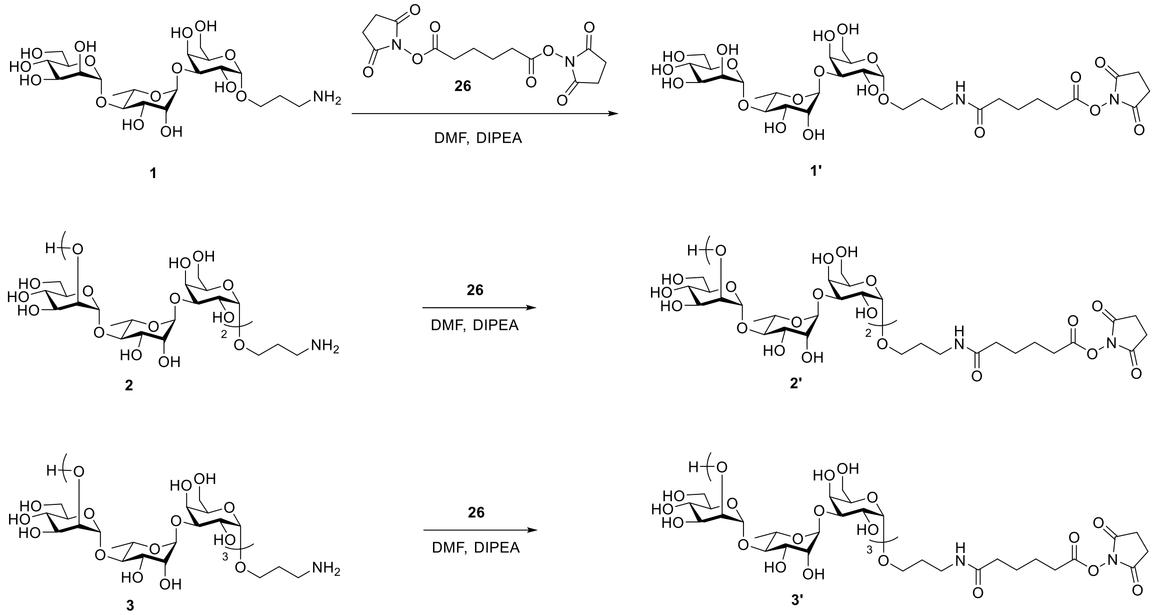

A solution of adipate bisNHS ester **26** (16 mg in 0.5 mL DMF, 0.045 mmol) was added into a solution of trisaccharide **1** (5 mg in 0.5 mL DMF, 0.009 mmol), which was followed by addition of di-isopropylethylamine (DIPEA, 2 µL, 0.009 mmol). The reaction mixture was stirred at room temperature for 4 h. After completion of the reaction, the solvent was removed under reduced pressure. The resulting residue was suspended in DCM and centrifuged. Under these conditions, product **1'** precipitated, whereas excess bis-NHS ester **26** remained soluble in DCM. The supernatant was carefully decanted, and the solid was washed with DCM three times in the same manner to remove residual **26**. The resulting solid was dried to give pure compound **1'** (5 mg, 71%). White solid; ^{1}H NMR (500 MHz, CD_3OD): δ 8.03 (t, J = 5.0 Hz, 1H, NH), 5.05 (d, J = 1.2 Hz, 1H), 4.82 (d, J = 3.9 Hz, 1H), 4.02 – 3.78 (m, 11H), 3.74 – 3.59 (m, 5H), 3.52 – 3.48 (m, 2H), 3.41 – 3.38 (m, 1H), 3.30 – 3.22 (m, 1H), 2.84 (s, 4H, CH_2 in NHS), 2.68 (t, J = 7.0 Hz, 2H, $COCH_2$), 2.25 (t, J = 6.9 Hz, 2H, $COCH_2$), 1.90 – 1.67 (m, 6H), 1.28 (d, J = 6.3 Hz, 3H, CH_3).

Compounds **2'** and **3'** were synthesized following the same procedure as that described for compound **1'**. ^{1}H NMR of **2'** (500 MHz, CD_3OD): δ 8.03 (t, J = 5.5 Hz, 1H, NH), 5.28 (s, 1H), 5.04 – 5.01 (m, 3H), 4.82 (d, J = 3.8 Hz, 1H), 4.14 – 3.58 (m, 31H), 3.55 – 3.48 (m, 3H), 3.41 – 3.38 (m, 1H), 3.30 – 3.24 (m, 1H), 2.84 (s, 4H, CH_2 in NHS), 2.68 (t, J = 6.9 Hz, 2H, $COCH_2$), 2.25 (t, J = 6.8 Hz, 2H, $COCH_2$), 1.89 – 1.69 (m, 6H), 1.29 (t, J = 6.0 Hz, 6H, $2CH_3$). ^{1}H NMR of **3'** (500 MHz, CD_3OD): δ 5.28 (s, 2H), 5.02 (dd, J = 9.2, 4.8 Hz, 4H), 4.81 (d, J = 3.9 Hz, 1H), 4.07 – 3.44 (m, 50H), 3.43 – 3.35 (m, 1H), 3.28 – 3.24 (m, 1H), 2.83 (s, 4H, CH_2 in NHS), 2.68 – 2.65 (m, 2H, $COCH_2$), 2.24 (t, J = 6.8 Hz, 2H, $COCH_2$), 1.86 – 1.67 (m, 6H), 1.30 – 1.27 (m, 9H, $3CH_3$).

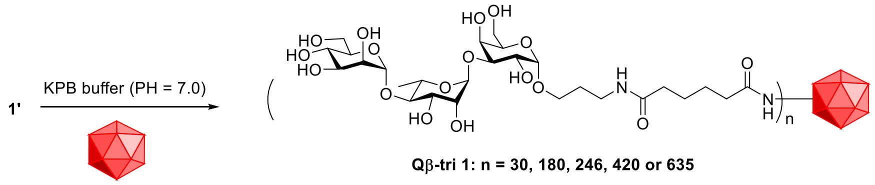

Qβ-tri 1: n = 30, 180, 246, 420 or 635

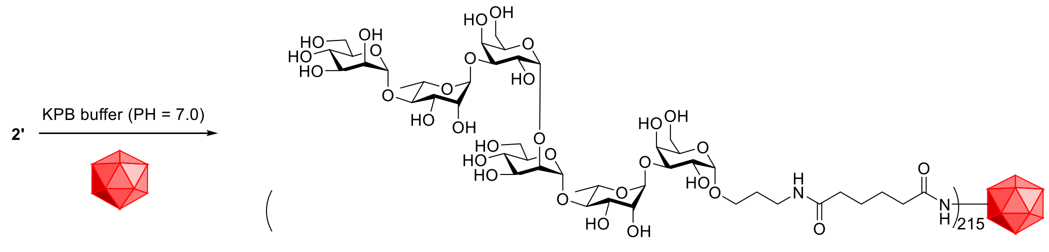

Qβ-hexa 2

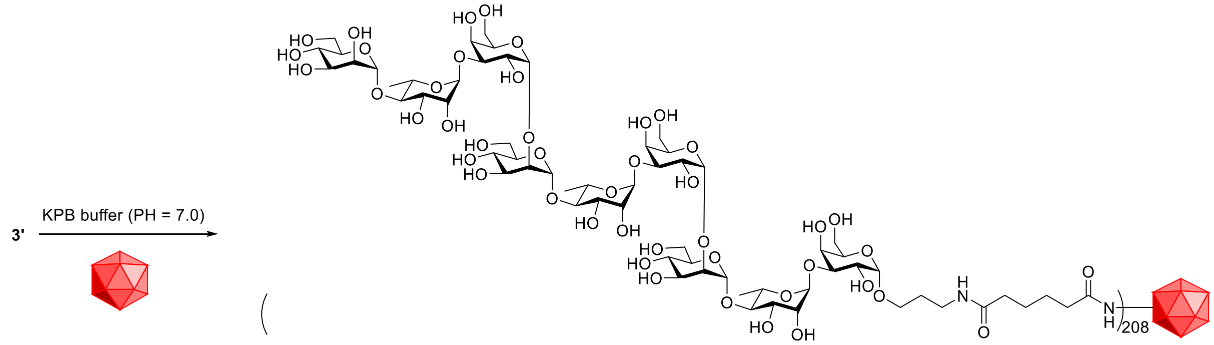

Qβ-nona 3

To a solution of bacteriophage Qβ (5 mg, expressed and purified as previously described)[1] in potassium phosphate buffer (0.1 M, pH = 7, 2.5 mL) was added a solution of **1'** (20 mg/mL in DMSO). The reaction mixture was stirred overnight at room temperature on a tube rotator. The resulting conjugate, designated **Qβ-tri**, was purified by repeated filtration using an Amicon Ultra 100 kDa MW cut-off device against PBS buffer. Total protein content was quantified by Bradford assay against BSA standards. An average loading of trisaccharide **1** per Qβ particle was determined by ESI-TOF LC-MS analysis.

By varying the amount of **1'** added, different degrees of trisaccharide loading were achieved. Addition of 0.10 mL (0.002 mmol) of **1'** resulted in an average loading of 30 trisaccharide units per Qβ particle (**Figure S1a**). When 0.20 mL (0.004 mmol), 0.25 mL (0.006 mmol), 0.50 mL (0.012 mmol), and 0.75 mL (0.019 mmol) of **1'** were used, average loadings of 180, 246, 460, and 635 trisaccharide units per Qβ particle were obtained, respectively (**Figures S1b-e**).

Qβ-glycan conjugate **Qβ-hexa** and **Qβ-nona** were prepared from **2** and **3**, respectively, by following a procedure analogous to that used for **Qβ-tri**. The average loadings of hexasaccharide **2** and nonasaccharide **3** per Qβ particle were determined by ESI-TOF LC-MS analysis to be 215 and 208, respectively (**Figures S2** and **S3**).

Scheme S1: Synthesis and characterization of BSA-glycan conjugates

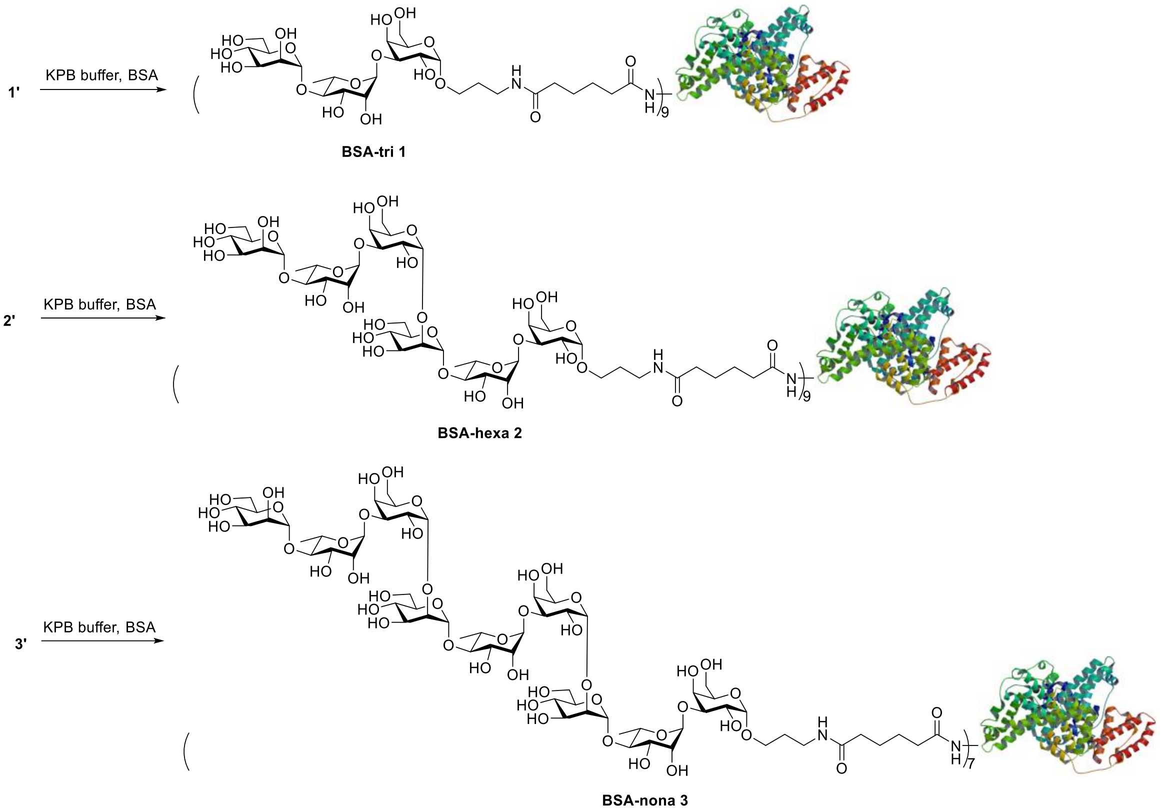

A solution of **1'** (20 mg/mL in DMSO, 0.1 mL, 0.003 mmol) was added to a solution of BSA (2 mg) in potassium phosphate buffer (0.1 M, pH = 7, 1 mL). The reaction mixture was stirred overnight at room temperature on a tube rotator. The conjugate was purified by repeated filtration using an Amicon Ultra 10 kDa MW cut-off device against PBS buffer. Total protein content was quantified by Bradford assay against BSA standards. A loading of 9 trisaccharides per BSA was determined using MALDI-TOF MS analysis (**Figure S4**).

BSA-glycan conjugates **BSA-hexa** and **BSA-nona** were prepared from compounds **2'** and **3'**, respectively, following a procedure analogous to that used for **BSA-tri**. The average loadings of hexasaccharide and nonasaccharide per BSA molecule were 9 and 7, respectively, as determined by MALDI-TOF MS analysis (**Figures S5** and **S6**).

Procedure for mouse immunization.

Pathogen-free C57BL6 female mice aged 6-10 weeks were purchased from Charles River and maintained in the University Laboratory Animal Resources facility of Michigan State University. All animal experiments were performed in accordance with the guidelines of the Institutional Animal Care and Use Committee (IACUC) of Michigan State University. C57BL6 mice were injected subcutaneously under

the scruff on day 0 with 0.1 mL Qβ-glycan constructs (3 μg glycan for trisaccharide based conjugates and 6 μg glycan for hexasaccharide and nonasaccharide based conjugates) as emulsions in Complete Freund's Adjuvant according to manufacturer's instructions. Boosters were given subcutaneously under the scruff on days 14 and 28 mixed with Incomplete Freund's Adjuvant. Serum samples were collected on days 0 (before immunization), 7 and 35. For single-dose immunization, mice received only the primary injection on day 0 without subsequent boosters, and serum samples were collected on day 35 for analysis.

In a separate mouse immunization study, the same immunization schedule and antigen doses for trisaccharide were used, except that 20 μg Alum (CAS# 21645-51-2, Alhydrogel® adjuvant 2%, InvivoGen) was used as the adjuvant per injection. For these experiments, Qβ-glycan constructs were formulated with Alum following the manufacturer's recommendations and administered subcutaneously on day 0, with booster injections given on days 14, 28 using the same Alum formulation. Sera samples were collected on days 0 (pre-immune), 7, and 35.

Procedure for rabbit immunization.

Rabbit immunization studies were performed by ProSci Inc (Poway, CA). Two New Zealand rabbits were injected subcutaneously on day 0 with 0.1 mL Qβ-glycan constructs (at 6 μg for trisaccharide based conjugates and 12 μg glycan for hexasaccharide and nonasaccharide based conjugates) as emulsions in Complete Freund's Adjuvant according to manufacturer's instructions. Boosters were given subcutaneously on days 14, 28 and 42 mixed with Incomplete Freund's Adjuvant. Serum samples were collected on days 0 (before immunization), 35, 49 and 56.

In a separate rabbit immunization study, the same immunization schedule and antigen doses for trisaccharide were used, except that 125 μg Alum (CAS# 21645-51-2, Alhydrogel® adjuvant 2%, InvivoGen) was used as the adjuvant per injection. For these experiments, Qβ-glycan constructs were formulated with Alum following the manufacturer's recommendations and administered subcutaneously on day 0, with booster injections given on days 14, 28, and 42 using the same Alum formulation. Serum samples were collected on days 0 (pre-immune), 35, 49, and 56.

Bacterial strains and growth conditions

The bacterial strains used in this study included wild-type *S*. Enteritidis R11, *S*. Typhimurium I77, *S*. Typhimurium D65, and *S*. Newport Chile 361, as well as genetically engineered COPS-producing strains CVD 1925 (wzzB mutant, *S*. Typhimurium), CVD 1943 (*S*. Enteritidis), and CVD 1966 (*S*. Newport). All the subtypes of *Salmonella* have been described previously[2, 3] and all strains were maintained in Hi-Soy (HS) bacteriological media (5 g/L sodium chloride, 10 g/L soytone [Teknova, CA], 5 g/L Hyyest [Sigma Aldrich, MO]) at 37 °C as described.[4]

Salmonella OPS used as an ELISA antigen were purified from fermentation cultures as described[5] previously by extraction with acetic acid and purification by sequential tangential flow filtration, anion exchange chromatography, and ammonium sulfate precipitation.

Evaluation of antibody titers by ELISA.

A 96-well Nunc microtiter plate was first coated with a solution of BSA-glycan conjugate or COPS (10 μg mL^{-1}, 100 μL/well) in $NaHCO_3/Na_2CO_3$ buffer (pH = 9.5) and incubated at 4 °C overnight. The plate was washed with PBST (4 × 200 μL), blocked with 1% BSA/PBS (200 μL/well) for 1 h at room temperature, washed with PBST (4 × 200 μL), and incubated with serial dilutions of anti-sera from immunized mice or rabbits in 0.1% BSA/PBS (100 μL/well, 4 wells for each dilution). The plate was incubated for 2 h at 37 °C and then washed with PBST (4 × 200 μL). A 1:2000 dilution of HRP-conjugated goat anti-mouse IgG (Cat. No. 115-035-003), IgM (Cat. No. 111-035-020), IgG1 (Cat. No. 111-035-205), IgG2b (Cat. No. 111-035-207), IgG2c (Cat. No. 111-035-208), and IgG3 (Cat. No. 111-035-209) or goat anti-rabbit IgG (Cat. No. 111-035-003) (all from Jackson ImmunoResearch Laboratory) in 0.1% BSA/PBS (100 μL) was added to the wells respectively to determine the levels of antibodies generated. The plate was incubated for 1 h at 37 °C. A solution of enzymatic substrate was prepared by dissolving TMB (5 mg) in a mixture of DMSO (2 mL) and citric acid buffer (18 mL) in a 50 mL centrifuge tube covered with aluminum foil. H_2O_2 (20 μL) was added and the mixture was homogenized by vortexing. The plate was washed with PBST (4 × 200 μL) and a solution of enzymatic substrate was added (200 μL). Color was allowed to develop for 15 min and 0.5 M H_2SO_4 (50 μL) was added to quench the reaction. The absorbance was measured at 450 nm using a microplate reader. The titer was determined by regression analysis with log10 dilution plotted with optical density. The titer was reported as the highest fold of dilution (ELISA unit) that gives OD = 0.3.

Procedure for flow cytometry

A single colony of *S.* Enteritidis R11 was grown in HS broth overnight at 37°C with shaking at 160 rpm. The following day, bacteria were adjusted to an OD600 of 0.4 and placed on ice. 250 μL of bacteria were washed once with flow buffer (1% heat-inactivated FBS in PBS) and incubated with different dilution of pooled rabbit sera (heat-inactivated for 30 min at 56 °C, pre-immune sera or post-immune (D49) sera) for 1 h at 4°C. Bacteria were then washed two times with flow buffer, followed by incubation with 5 μg/mL of FITC-conjugated donkey anti-rabbit IgG (Cat# 406003, Biolegend) diluted in flow buffer for 1 h at 4 °C. Bacteria were washed twice with PBS, fixed with 2% formaldehyde, and read using BD Accuri C6 with 1 × 10^4 events recorded. As a negative control, bacteria were incubated with secondary antibody alone. Flow cytometry studies on other strains were conducted in similar manners.

Procedure for opsonophagocytosis assay

THP-1 (human acute monocytic leukemia cell line, ATCC TIB-202) were maintained in Roswell Park Memorial Institute (RPMI) 1640 (Thermo Fisher, #11875093) with L-glutamine and 10% v/v heat-inactivated FBS (Cat# P711S500, Lonza), hereafter referred to as R10. For routine subculture, R10 was supplemented with 100 U/mL penicillin/100 μg/mL streptomycin (Cat# 15140122, Gibco) to prevent contamination. Cells were maintained at a density between 2×10^5 and 1×10^6 cells/mL. For differentiation, THP-1 cells were harvested, counted, and seeded into 24-well plates at 1×10^6 cells/well. Phorbol 12-myristate 13-acetate (PMA; CAS# 16561-29-8, MedChemExpress) was added to a final concentration of 100 nM. Plates were gently mixed and incubated for 24 h at 37 °C in a humidified atmosphere containing 5% CO_2. Following differentiation, partially confluent THP-1 monolayers were washed three times with R10 to remove PMA-containing medium. The media was then replaced with 500 μL of fresh complete RPMI-1640 without PMA, and cells were incubated for an additional 24-48 h. Once a stable monolayer had formed, the culture medium was replaced with R10. Bacteria were prepared in the following manner: a single colony of *S*. Enteritidis R11 was used to inoculate an overnight HS broth culture (37 °C, 220 rpm). The following day, log-phase cultures were obtained by diluting the overnight culture into fresh HS broth and incubating under the same conditions. Bacterial cultures were adjusted to an OD600 of 0.1 in PBS, corresponding to approximately 8×10^4 CFU/μL. For opsonization, 22.5 μL of the diluted bacteria, 20 μL of pooled rabbit sera, and 55.5 μL of PBS were combined in microcentrifuge tubes and incubated for 20 min at room temperature with periodic gentle mixing. The sera for this experiment were collected from Qβ-tri immunized rabbits (pre-immunization and day 49 post-immunization) and Qβ-immunized rabbits (day 49 post-immunization). Subsequently, 6 μL of the opsonized bacteria (1×10^5 CFU) were added to duplicate wells of the THP-1 monolayers in 24-well plate. Plates were centrifuged at 500 × g for 2 min to facilitate bacterial contact with cells, followed by incubation at 37 °C / 5% CO_2 for 50 min. After incubation, wells were washed once with sterile R10, and 500 μL of R10 supplemented with 100 μg/mL of gentamicin (Cat# 15750060, Gibco) was added to each well. Plates were returned to the 37 °C /5% CO_2 incubator for 1 h. After gentamicin treatment, wells were washed three times with sterile R10, and cells were lysed with 500 μL of sterile water for 10 min with shaking at 250 rpm. Wells were then scraped with a pipette tip to ensure complete lysis. Lysates were then serially diluted in PBS and plated on HS agar plates to determine viable counts.

S. Typhimurium I77 and *S*. Typhimurium D65 were prepared and tested using the same experimental procedure.

Passive immunization and lethal *Salmonella* challenge in mice

Pre-immunization and day 49 sera from Qβ-tri immunized were pooled, heat-inactivated at 56 °C for 30 min and diluted in PBS. Naïve 8-week-old female CD-1 mice (Charles River Laboratories, USA)

were injected intraperitoneally (i.p.) with diluted rabbit sera or PBS as a control. Five to six hours later, mice were challenged i.p. with the indicated *Salmonella* strains. For all challenge experiments, bacteria were grown to log phase, washed, and diluted in sterile PBS immediately prior to injection. For *S*. Enteritidis R11, naïve CD-1 mice received pooled pre-immune or day 49 post-immunization rabbit sera diluted 1:100 in PBS and were challenged 5-6 h later with 1.3×10^6 CFU of *S*. Enteritidis R11. For *S*. Typhimurium D65, the same protocol was followed, except that mice were challenged with 6.5×10^5 CFU. For *S*. Typhimurium I77, pooled rabbit sera were diluted 1:200 in PBS, and mice were challenged with 5×10^5 CFU. Following challenges, mice were monitored three times daily for up to 21 days. Animals that became moribund and/or exhibited sustained body weight loss of ≥20% were euthanized and recorded as having succumbed to infection. At 21 days post-challenge, all surviving mice were euthanized, and their kidneys and spleens were aseptically collected and homogenized. Bacterial burdens were determined by serial dilution and plating on HS agar plates to confirm clearance of the challenge bacteria.

Mouse tissue collection and processing for histopathology

All mice were euthanized by carbon dioxide inhalation followed by cervical dislocation. A full postmortem examination was completed including body weight determination, gross examination of all organs, and collection of critical organs in 10% neutral buffered formalin (including lung, kidney, and liver). Tissues were processed using standard histologic processes, embedded in paraffin, sectioned at 5 μm, and stained with hematoxylin and eosin. The slides were examined at 2 - 40 × magnification using a standard bright field microscope. Lesions were recorded and collated. Images were taken using an Idea® digital camera, model 28.2 (5MP).

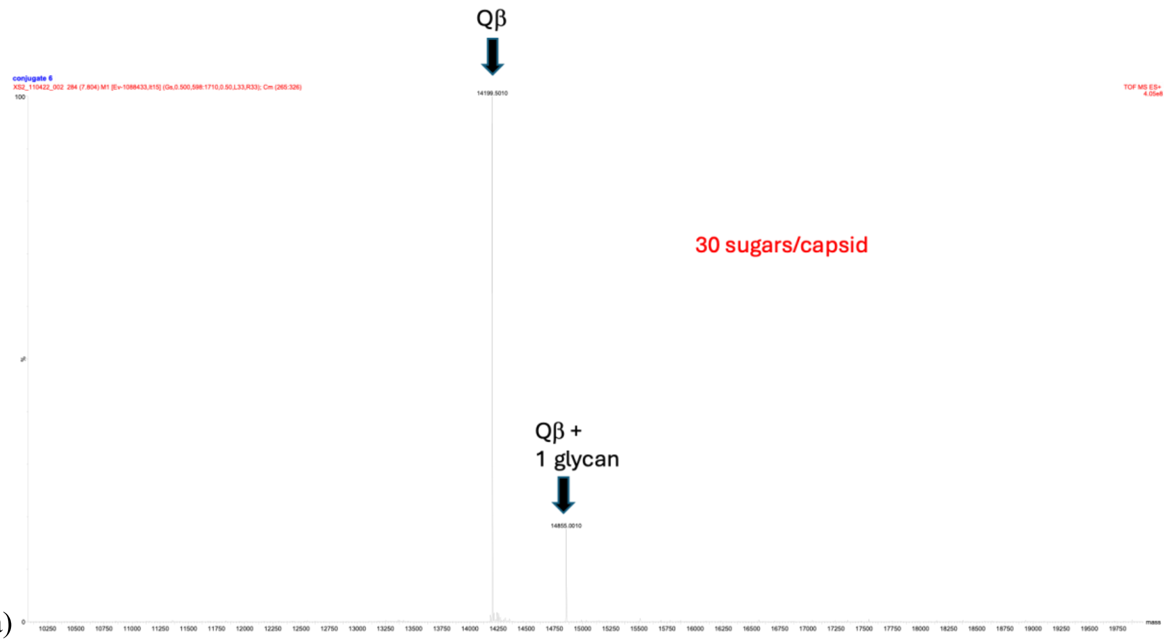
Qβ
conjugate 6
XS2_110422_002 284 (7.804) M1 [Ev-1088433,It15] (Gs,0.500,598:1710,0.50,L33,R33); Cm (265:326)
14199.5010
TOF MS ES+
30 sugars/capsid
Qβ +
1 glycan
14855.0010
mass
a)

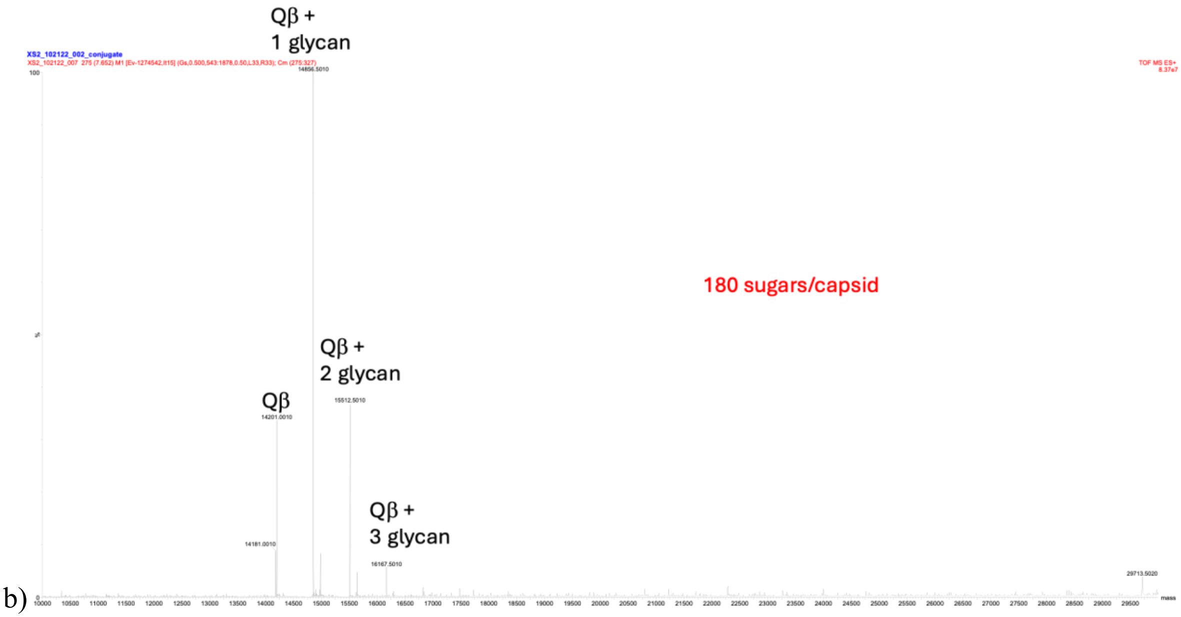
Qβ +
1 glycan
XS2_102122_002_conjugate
XS2_102122_007 275 (7.652) M1 [Ev-1274542,It15] (Gs,0.500,543:1878,0.50,L33,R33); Cm (275:327)
14856.5010
TOF MS ES+
180 sugars/capsid
Qβ +
2 glycan
15512.5010
Qβ
14201.0010
Qβ +
3 glycan
14181.0010
16167.5010
29713.5020
mass
b)

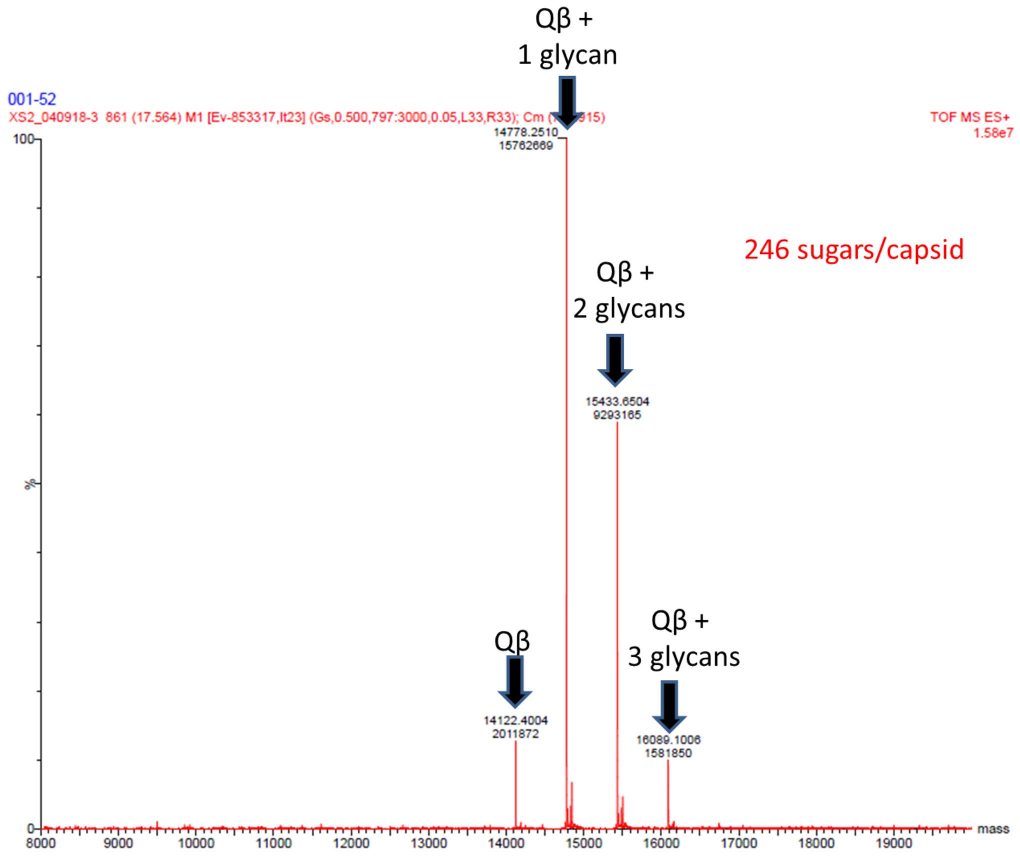
Qβ +
1 glycan
001-52
TOF MS ES+
1.58e7
14778.2510
15762669
246 sugars/capsid
Qβ +
2 glycans
15433.6504
9293165
Qβ
14122.4004
2011872
Qβ +
3 glycans
16089.1006
1581850
%
mass
8000
9000
10000
11000
12000
13000
14000
15000
16000
17000
18000
19000

c)

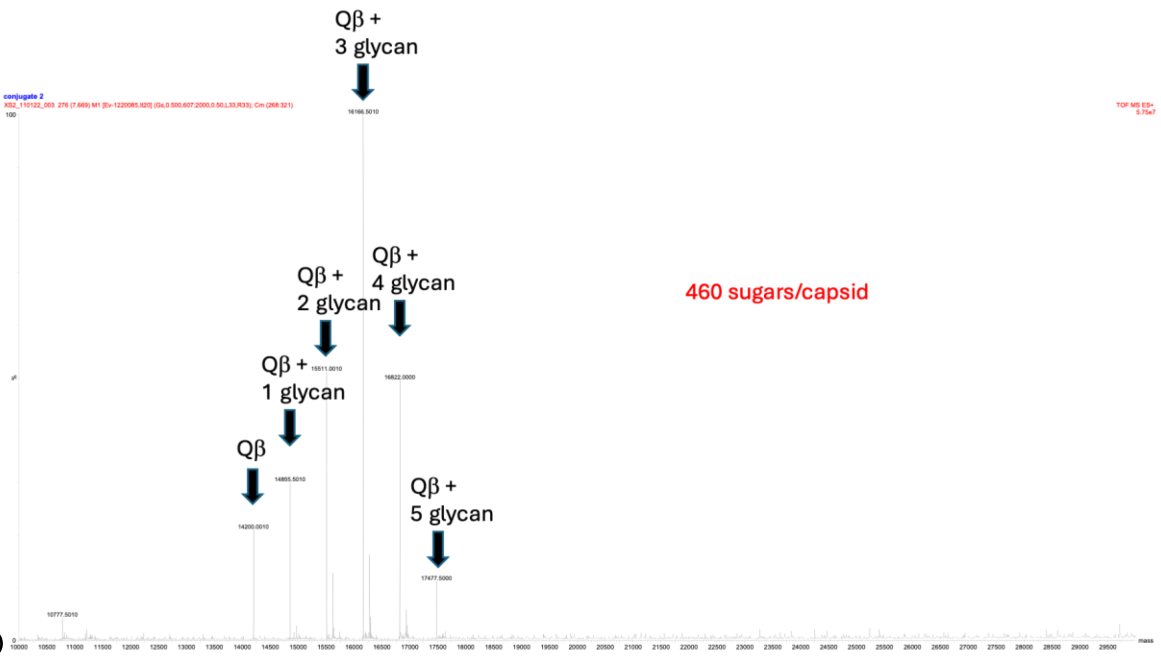
Qβ +
3 glycan
Qβ +
4 glycan
Qβ +
2 glycan
460 sugars/capsid
Qβ +
1 glycan
Qβ
Qβ +
5 glycan
conjugate 2
mass

d)

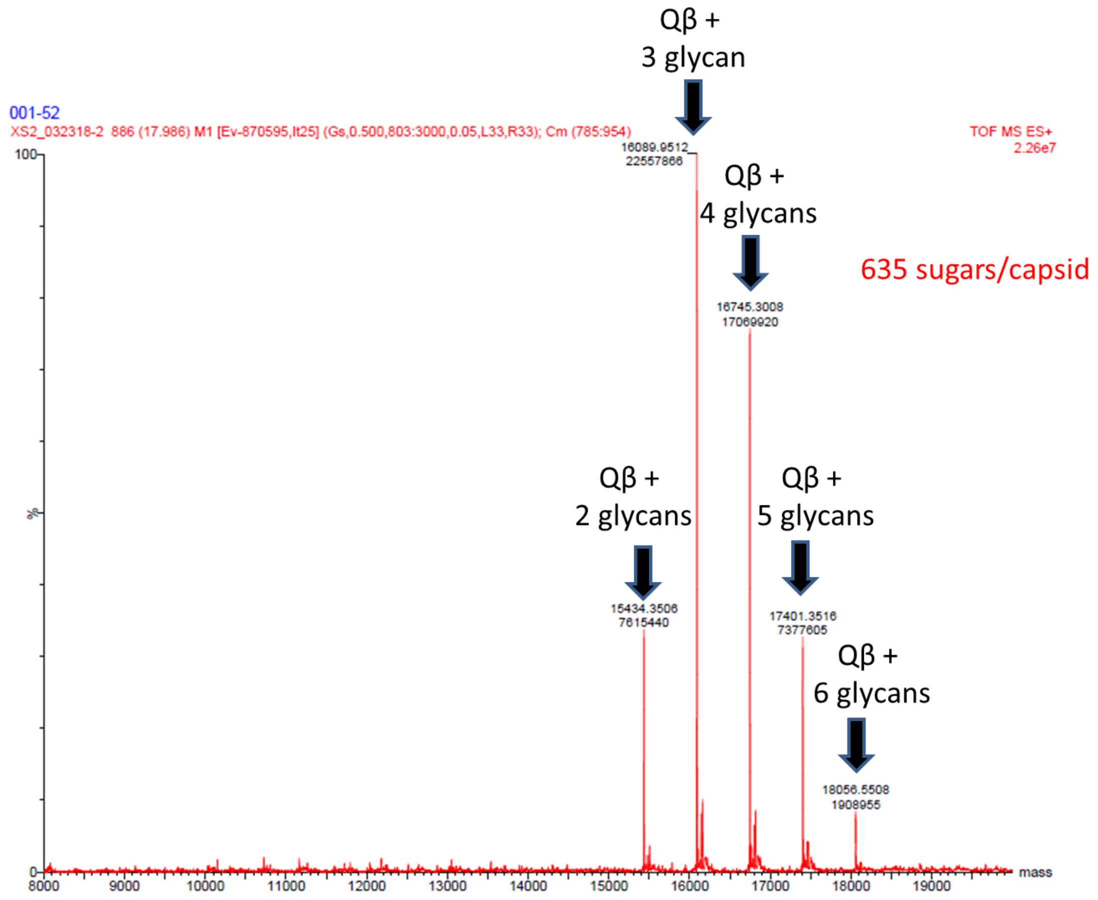

e)

Figure S1. ESI-TOF LC-MS of Qβ-glycan conjugate Qβ-tri **1** with peaks from Qβ monomer and the respective Qβ-glycan conjugates marked. Assuming Qβ monomer and Qβ-glycan conjugates have the same ionizing efficiencies, the ion intensities of the peaks were used to estimate the relative quantities of each species. a) On average, there were 0.17 glycan **1** per Qβ monomer, which corresponds to 30 copies of glycan **1** per Qβ particle; b) On average, there were 1 glycan **1** per Qβ monomer, which corresponds to 180 copies of glycan **1** per Qβ particle; c) On average, there were 1.37 glycan **1** per Qβ monomer, which corresponds to 246 copies of glycan **1** per Qβ particle; d) On average, there were 2.56 glycan **1** per Qβ monomer, which corresponds to 460 copies of glycan **1** per Qβ particle; e) On average, there were 3.53 glycan **1** per Qβ monomer, which corresponds to 635 copies of glycan **1** per Qβ particle.

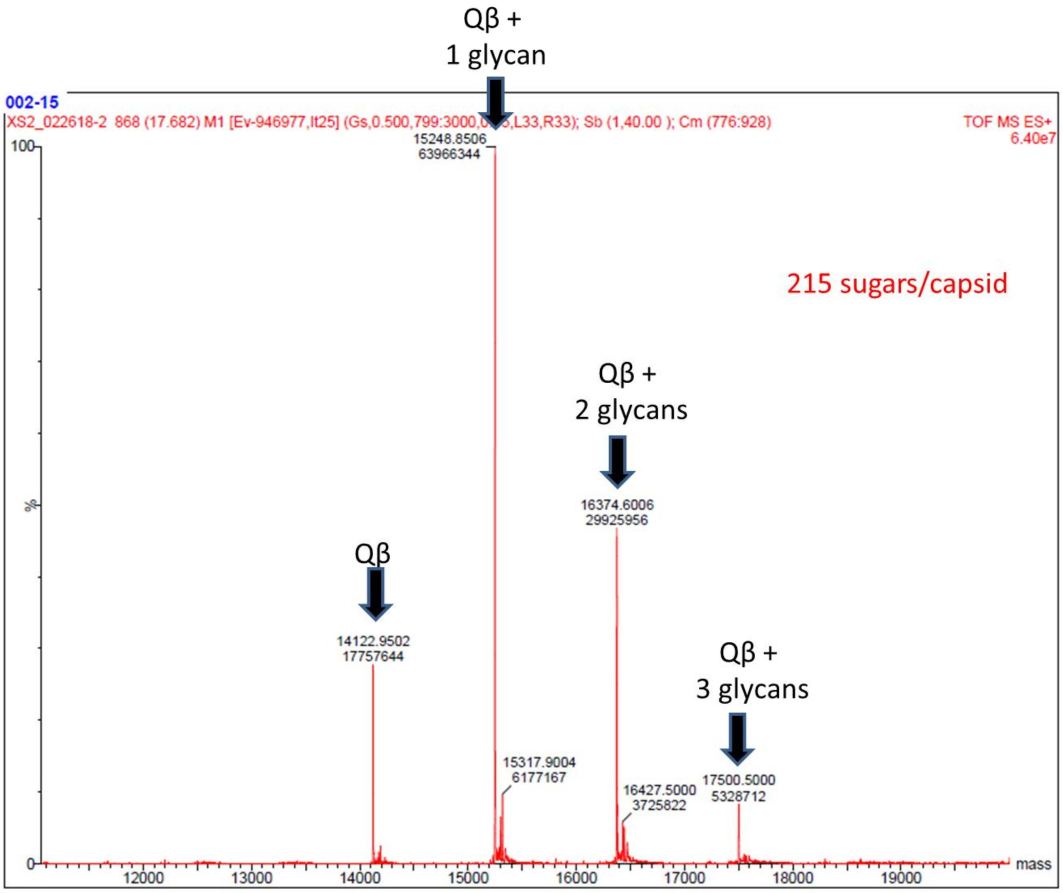

Figure S2. ESI-TOF LC-MS of Qβ-glycan conjugate **Qβ-hexa 2** with peaks from Qβ monomer and the respective Qβ-glycan conjugates marked. Assuming Qβ monomer and Qβ-glycan conjugates have the same ionizing efficiencies, the ion intensities of the peaks were used to estimate the relative quantities of each species. On average, there were 1.19 glycan **2** per Qβ monomer, which corresponds to 215 copies of glycan **2** per Qβ particle.

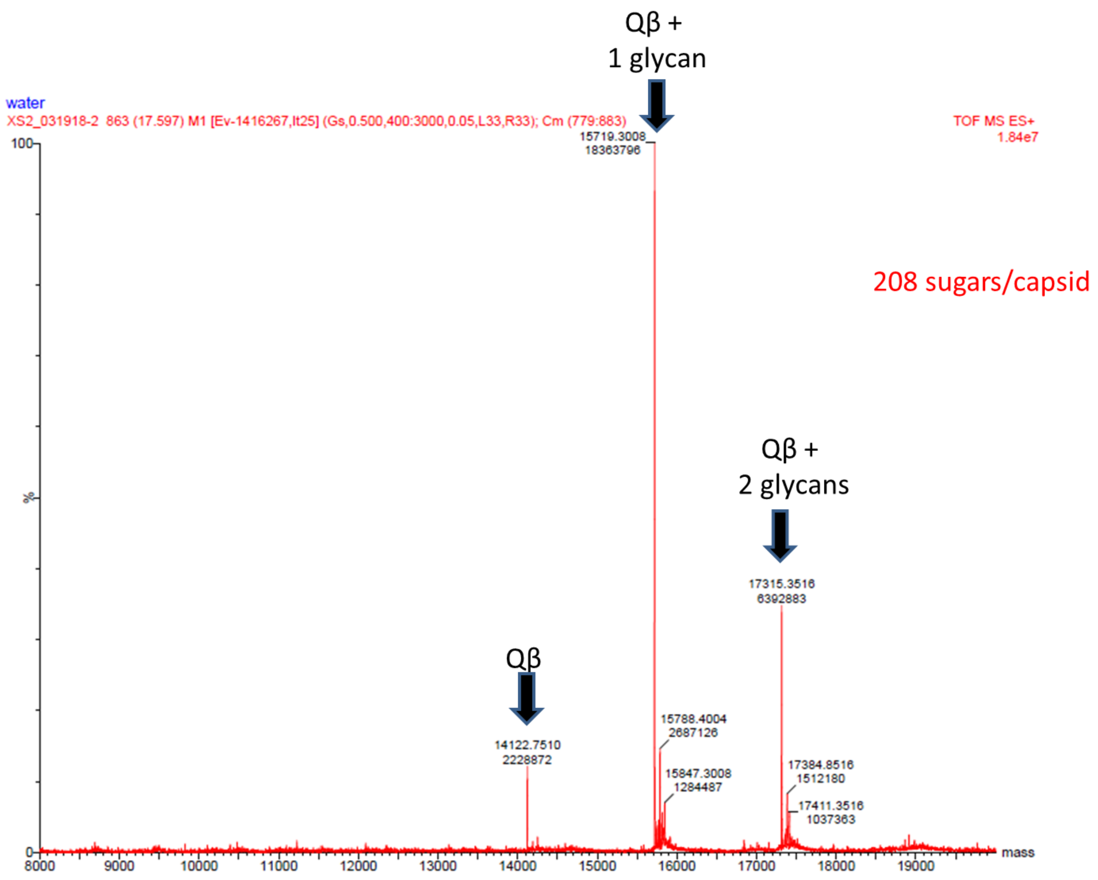

Figure S3. ESI-TOF LC-MS of Qβ-glycan conjugate **Qβ-nona 3** with peaks from Qβ monomer and the respective Qβ–glycan conjugates marked. Assuming Qβ monomer and Qβ-glycan conjugates have the same ionizing efficiencies, the ion intensities of the peaks were used to estimate the relative quantities of each species. On average, there were 1.16 glycan **3** per Qβ monomer, which corresponds to 208 copies of glycan **3** per Qβ particle.

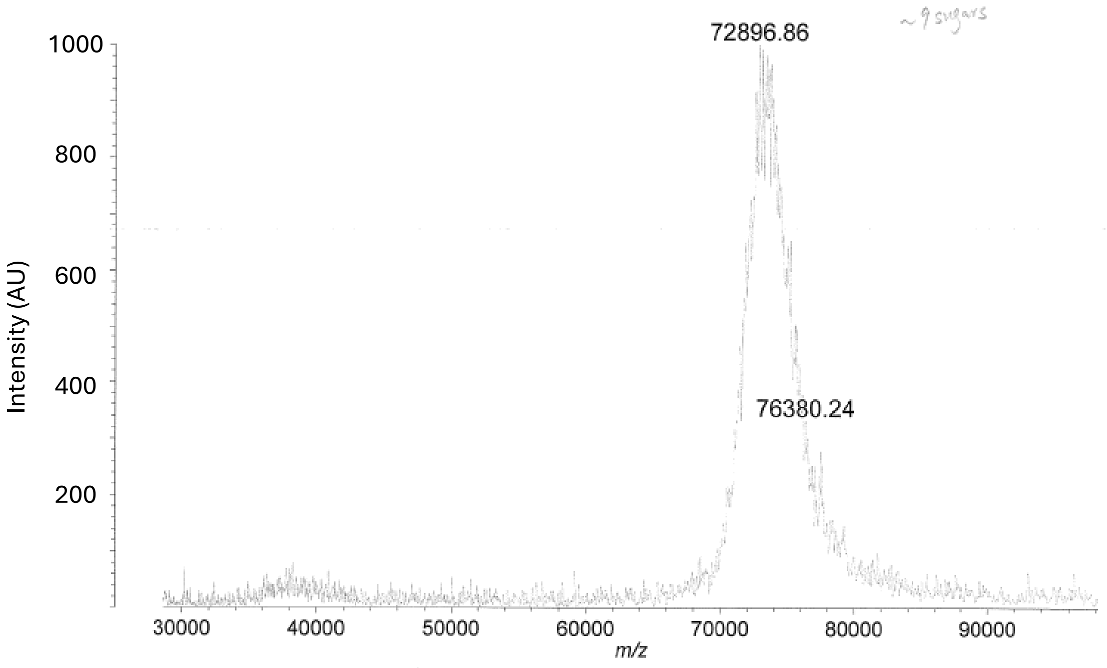

Figure S4. MALDI-TOF MS of **BSA-tri** conjugate**.** Based on the molecular weight difference between the **BSA-tri** conjugate and unmodified BSA, the average number of glycan **1** per BSA was calculated to be 9.

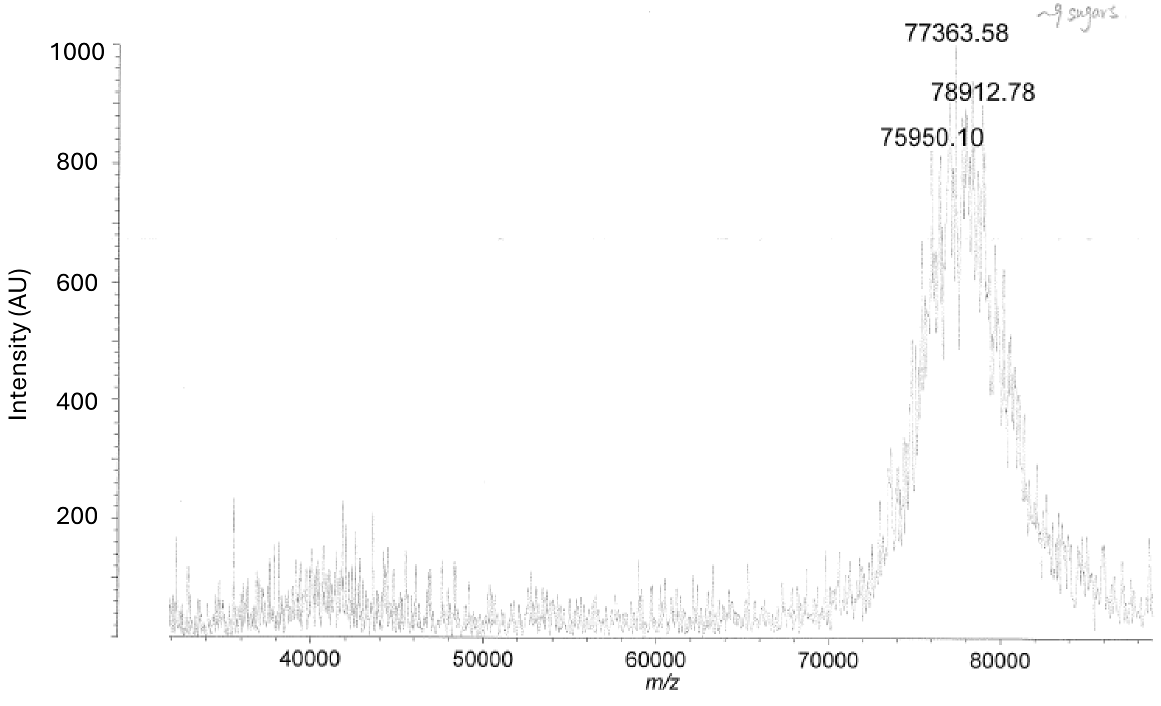

Figure S5. MALDI-TOF MS of **BSA-hexa** conjugate**.** Based on the molecular weight difference between the **BSA-hexa** conjugate and unmodified BSA, the average number of glycan **2** per BSA was calculated to be 9.

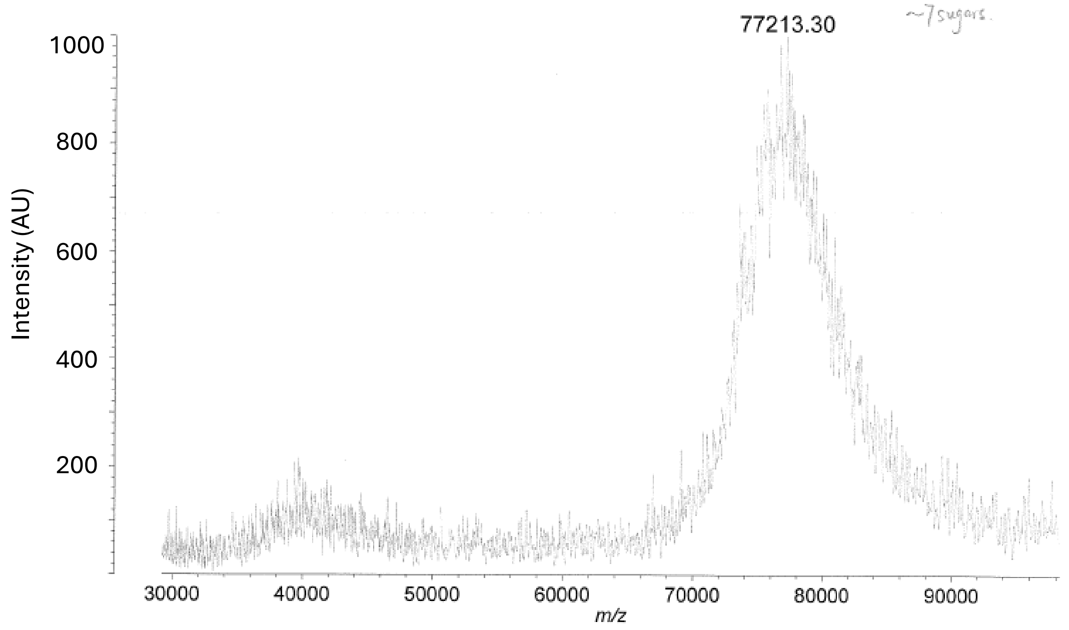

Figure S6. MALDI-TOF MS of **BSA-nona** conjugate**.** Based on the molecular weight difference between the **BSA-nona** conjugate and unmodified BSA, the number of glycan **3** per BSA was calculated to be 7.

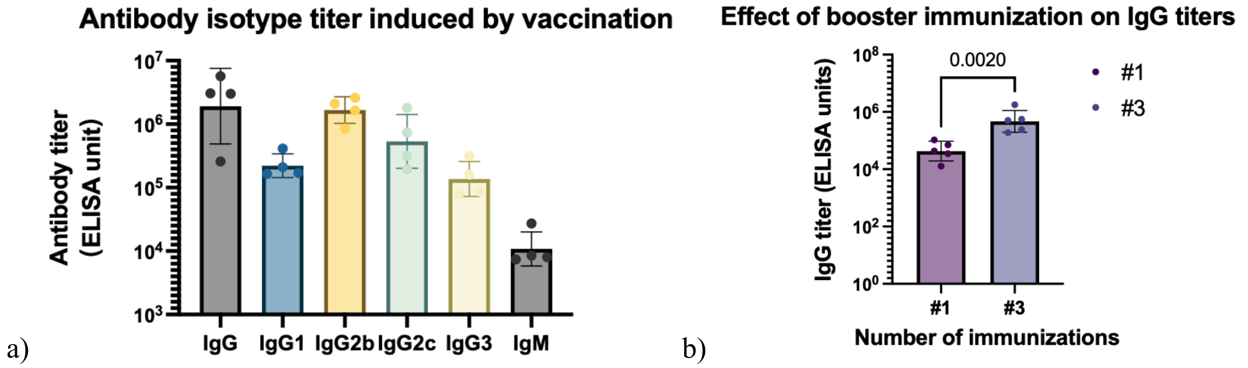

Figure S7: (a) Geometric mean titers of anti-glycan **1** IgG subtype and IgM from mice vaccinated with Qβ-tri$_{(246)}$; b) ELISA analysis of day-35 sera of mice immunized with one (#1) or three (#3) doses of Qβ-tri$_{(246)}$. While Immunization with one dose produced significant amounts of anti-glycan **1** IgG antibodies on day 35, three dose immunization elicited significantly higher IgG titers compared to one dose, suggesting booster can significantly enhance the antibody immune response. Statistical analysis was performed via a student t test. P = 0.0020.

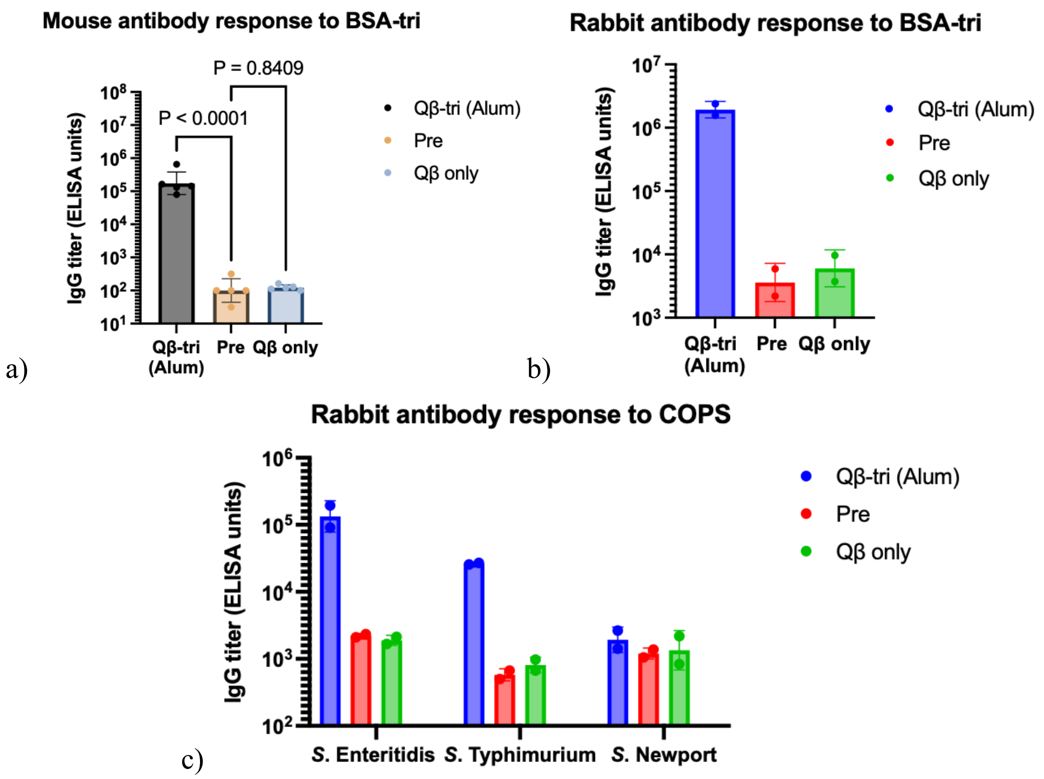

Figure S8. a) Anti-glycan IgG titers from mice immunized Qβ only, Qβ-trisaccharide **1** formulated with alum, including pre-immune sera; b) Anti-glycan IgG titers from rabbit immunized with Qβ only, Qβ-trisaccharide **1** formulated with alum, including pre-immune sera; Qβ–glycan conjugates elicited significantly higher levels of IgG antibodies specific to the immunizing glycan compared with pre-immune sera or sera from Qβ-immunized animals. b) Anti-COPS IgG ELISA titers in sera from rabbits immunized with Qβ-trisaccharide **1** against COPS from *S.* Enteritidis R11, *S.* Typhimurium, and *S.* Newport Chile 361. Statistical significance was determined using one-way ANOVA followed by Dunnett's multiple comparisons test. Each symbol corresponds to an individual animal. Data are presented as geometric mean values ± standard deviation.

Table S1. Calculation of vaccine efficacy. Vaccine efficacy (%) = (1 – mortality rate of immune sera group/mortality rate of the group receiving pre-immune sera) ×100%.

	***S.* Enteritidis R11**	***S.* Typhimurium D65**	***S.* Typhimurium I77**
**PBS (Mortality)**	100%	100%	100%
**Pre (Mortality)**	100%	77.8%	83.3%
**Qβ (Mortality)**	92.3%	Not performed	83.3%
**Vaccine efficacy of Qβ**	7.7%	Not performed	0%
**Qβ-tri (Mortality)**	5.6%	27.8%	41.6%
**Vaccine efficacy of Qβ-tri**	94.4%	64.3%	50.0%

References

1. Yin, Z.; Chowdhury, S.; McKay, C.; Baniel, C.; Wright, W. S.; Bentley, P.; Kaczanowska, K.; Gildersleeve, J. C.; Finn, M. G.; BenMohamed, L.; Huang, X., Significant Impact of Immunogen Design on the Diversity of Antibodies Generated by Carbohydrate-Based Anticancer Vaccine. *ACS Chem. Biol.* **2015,** *10* (10), 2364-2372.
2. Tennant, S. M.; Wang, J. Y.; Galen, J. E.; Simon, R.; Pasetti, M. F.; Gat, O.; Levine, M. M., Engineering and preclinical evaluation of attenuated nontyphoidal Salmonella strains serving as live oral vaccines and as reagent strains. *Infect. Immun.* **2011,** *79* (10), 4175-4185.
3. Nasrin, S.; Fuche, F. J.; Sears, K. T.; Jones, J. A.; Levine, M. M.; Simon, R.; Tennant, S. M., Refinement of a Live Attenuated Salmonella enterica Serovar Newport Vaccine with Improved Safety. *Vaccines (Basel)* **2021,** *9* (1).
4. Darton, T. C.; Baliban, S. M.; Curtis, B.; Toema, D.; Tennant, S. M.; Levine, M. M.; Pasetti, M. F.; Simon, R., Immunogenicity and efficacy following sequential parenterally-administered doses of Salmonella Enteritidis COPS:FliC glycoconjugates in infant and adult mice. *PLoS Negl. Trop. Dis.* **2018,** *12* (5), e0006522.
5. Baliban, S. M.; Yang, M.; Ramachandran, G.; Curtis, B.; Shridhar, S.; Laufer, R. S.; Wang, J. Y.; Van Druff, J.; Higginson, E. E.; Hegerle, N.; Varney, K. M.; Galen, J. E.; Tennant, S. M.; Lees, A.; MacKerell, A. D., Jr.; Levine, M. M.; Simon, R., Development of a glycoconjugate vaccine to prevent invasive Salmonella Typhimurium infections in sub-Saharan Africa. *PLoS Negl Trop Dis* **2017,** *11* (4), e0005493.

NMR spectra

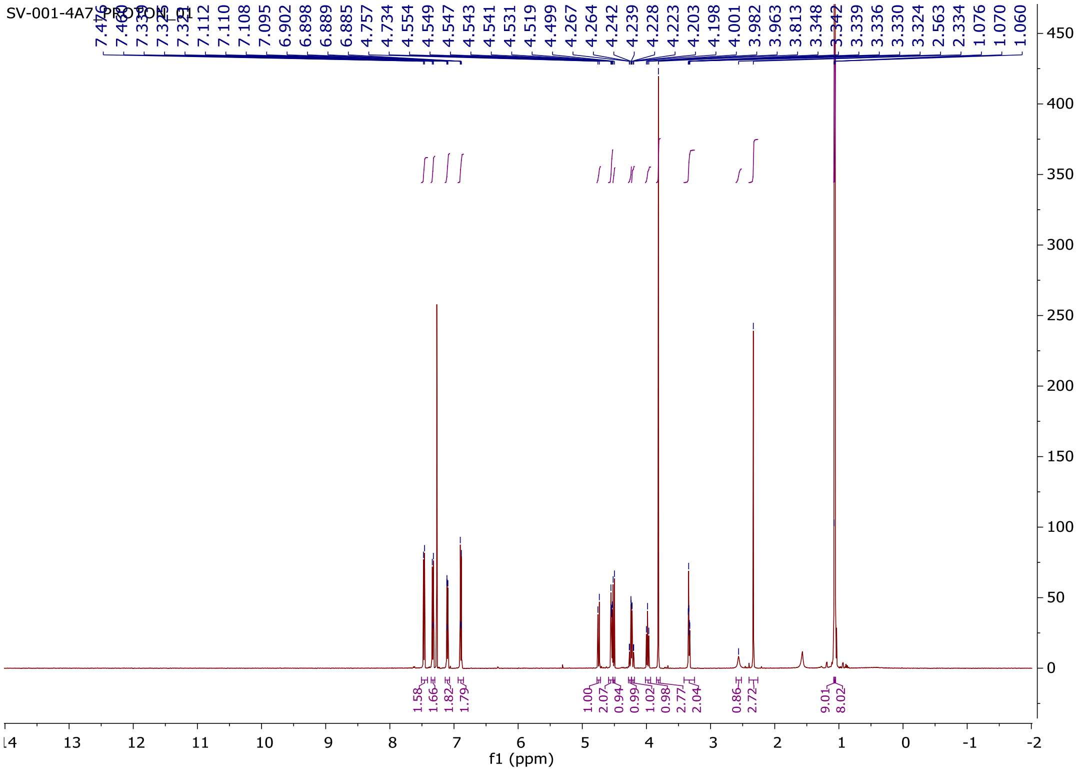

^{1}H NMR spectrum of compound **1**

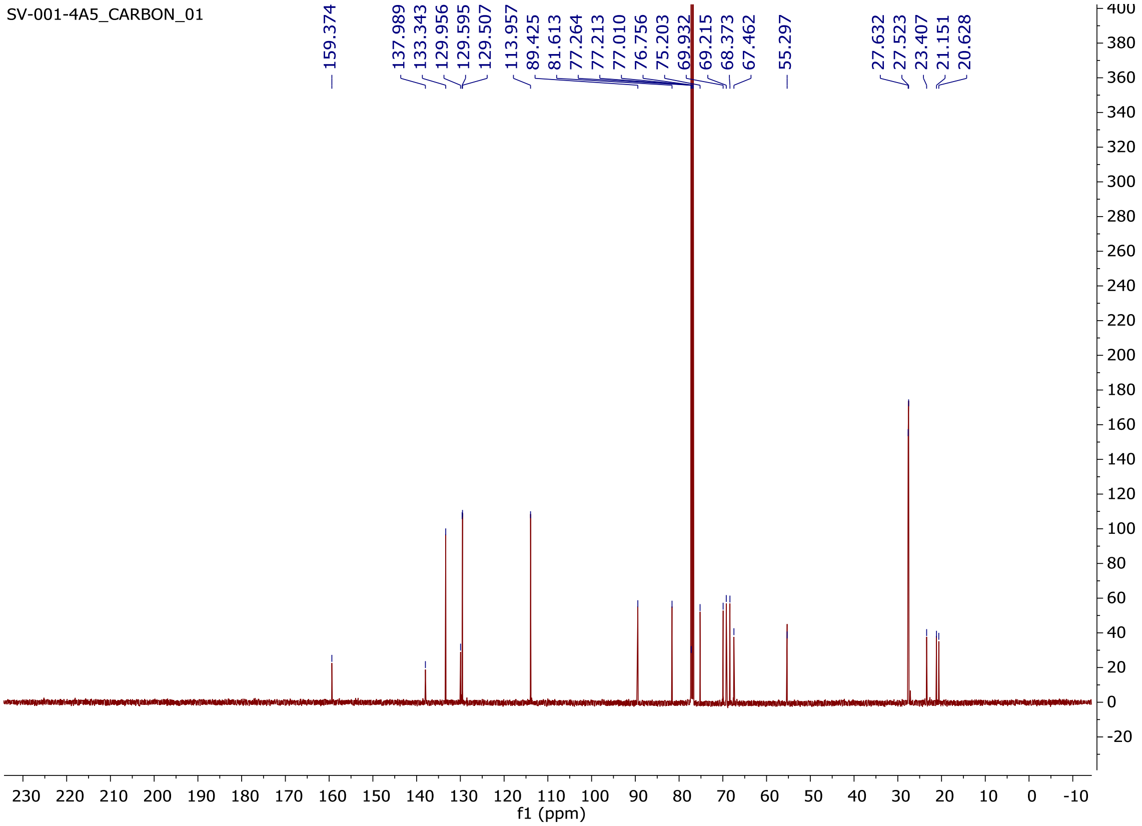

^{13}C NMR spectrum of compound **1**

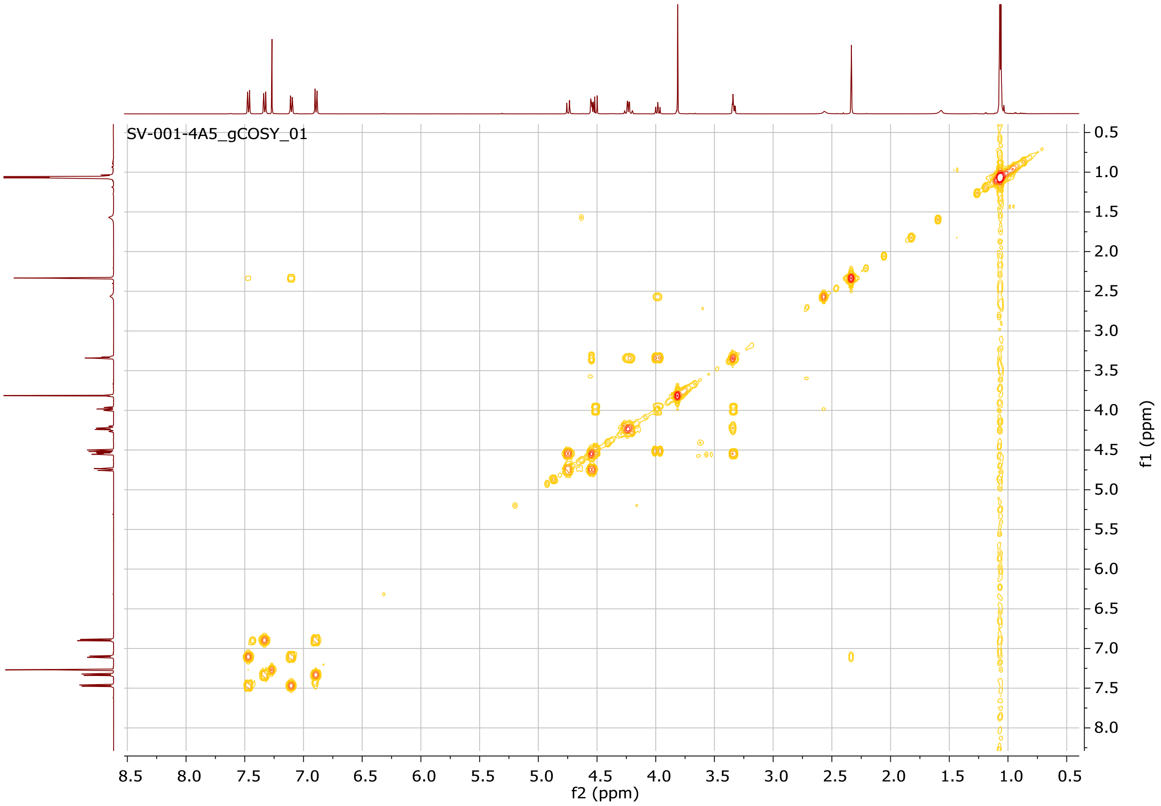

gCOSY NMR spectrum of compound **1**

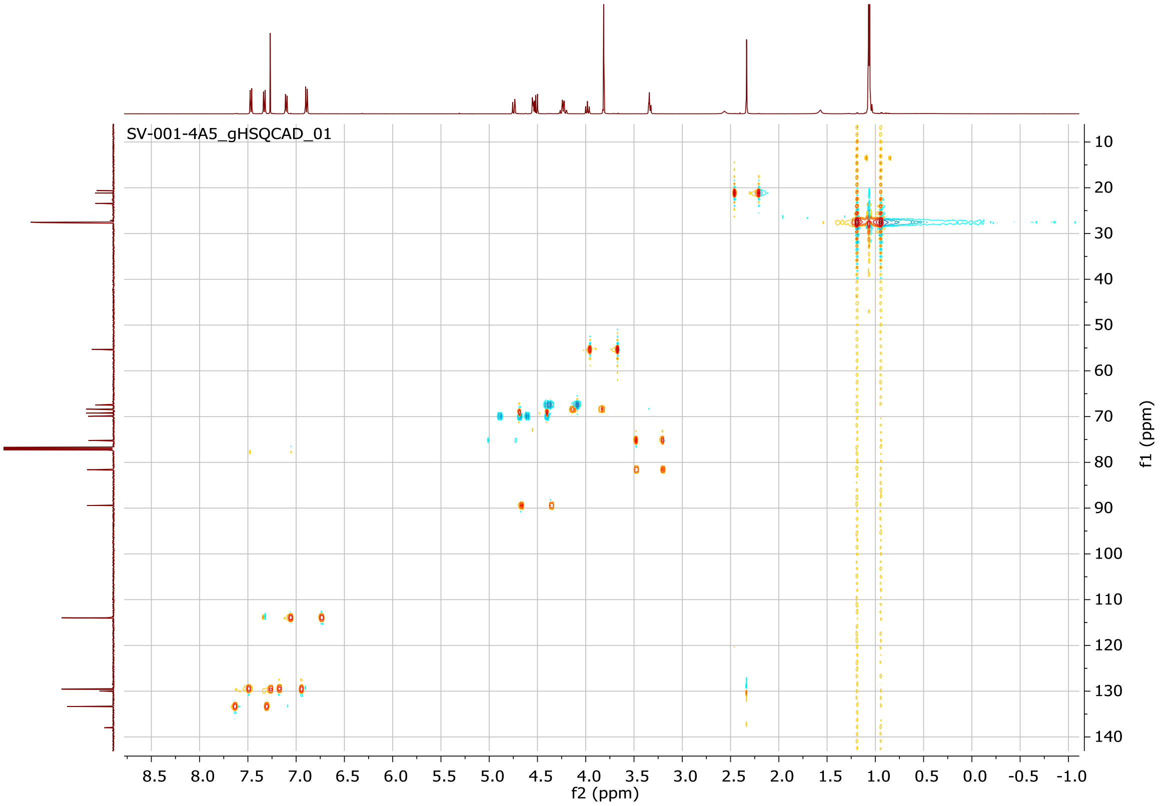

^{1}H-Coupled gHSQC NMR spectrum of compound **1**

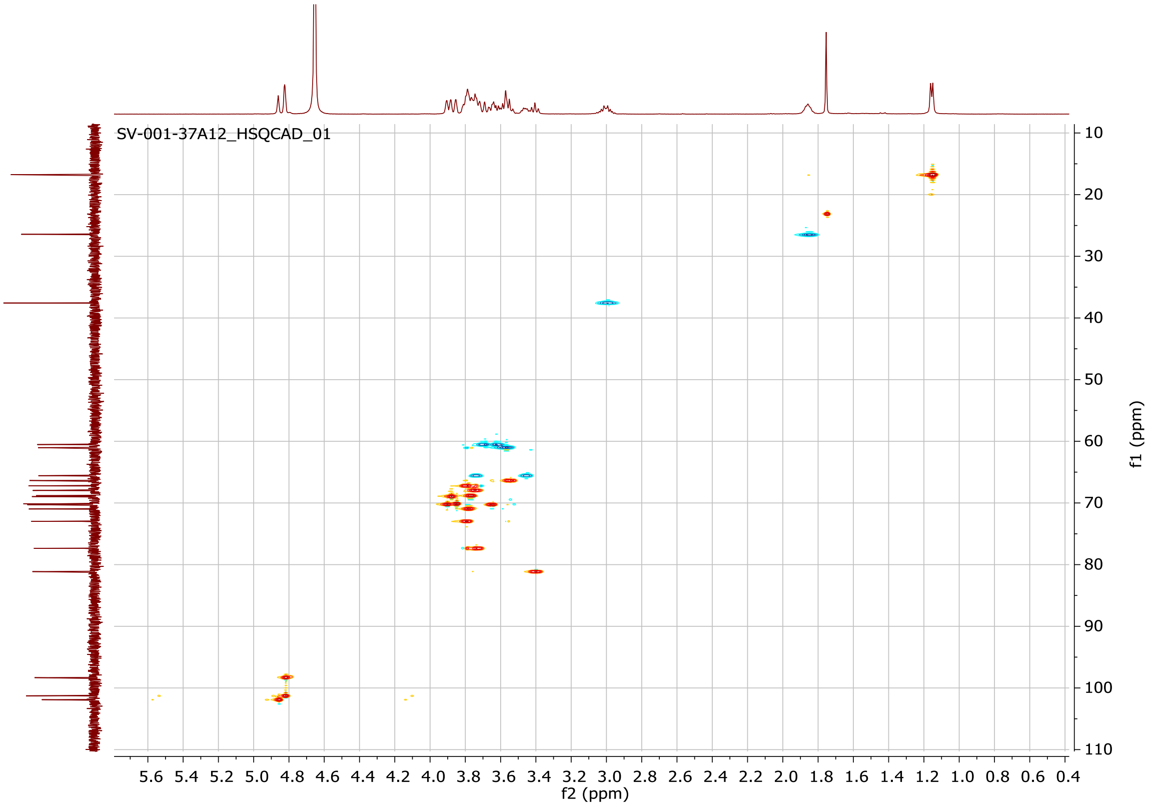

gHSQC NMR spectrum of compound **1**

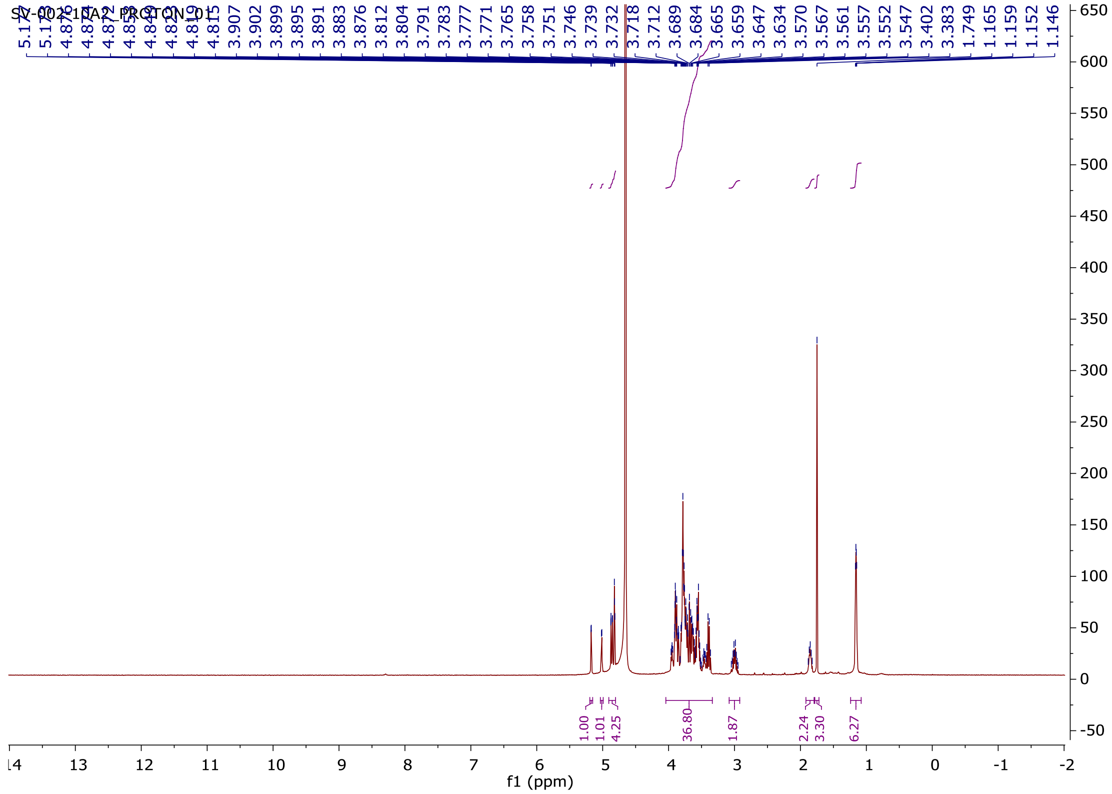

^{1}H NMR spectrum of compound **2**

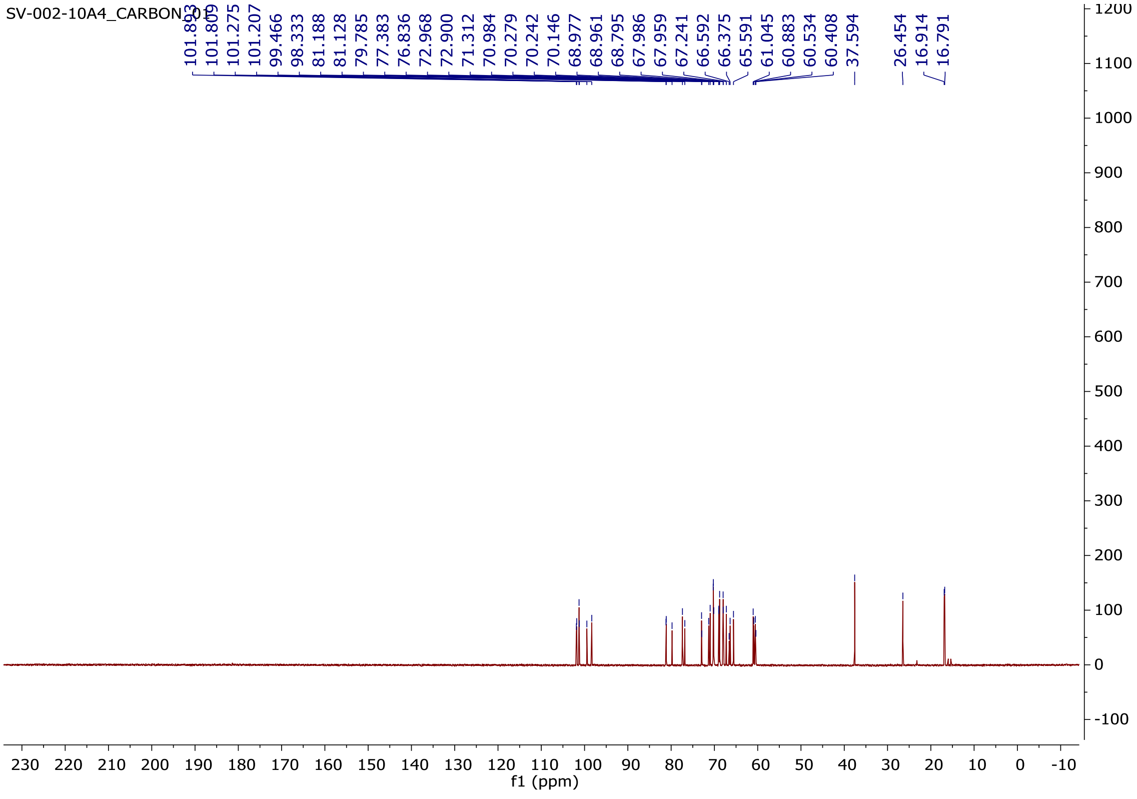

^{13}C NMR spectrum of compound **2**

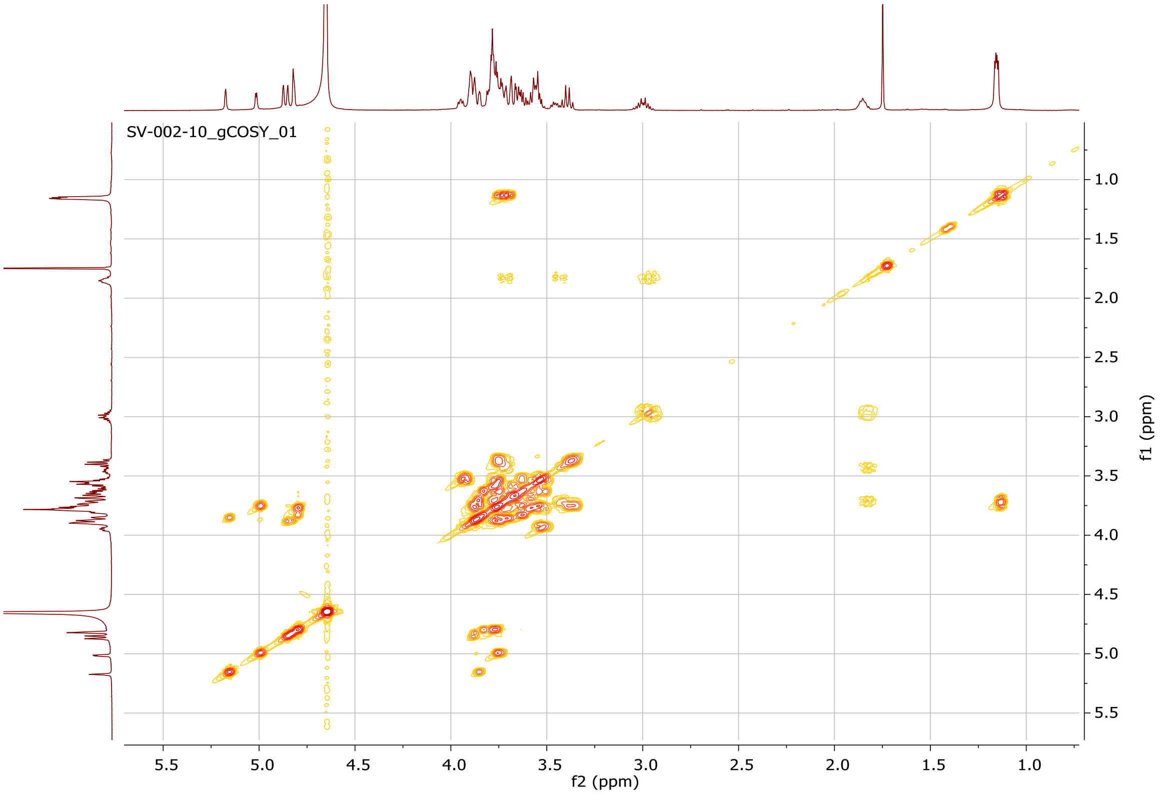

gCOSY NMR spectrum of compound **2**

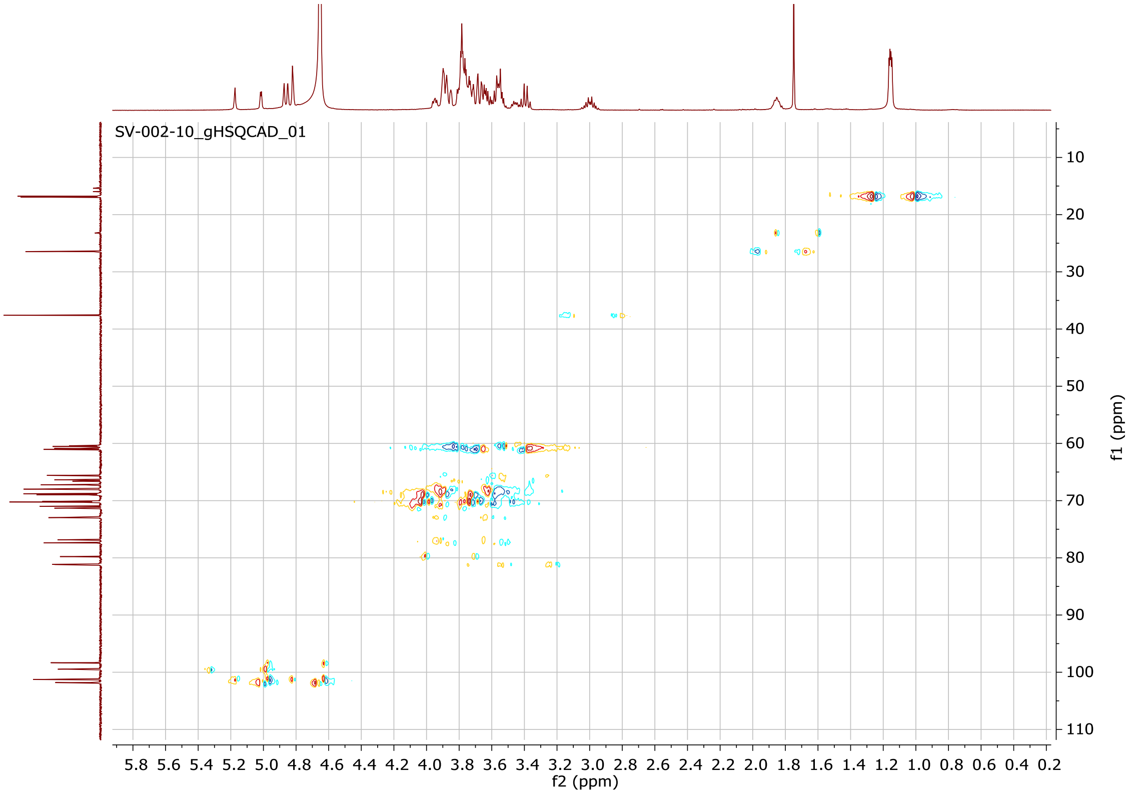

^{1}H-Coupled gHSQC NMR spectrum of compound **2**

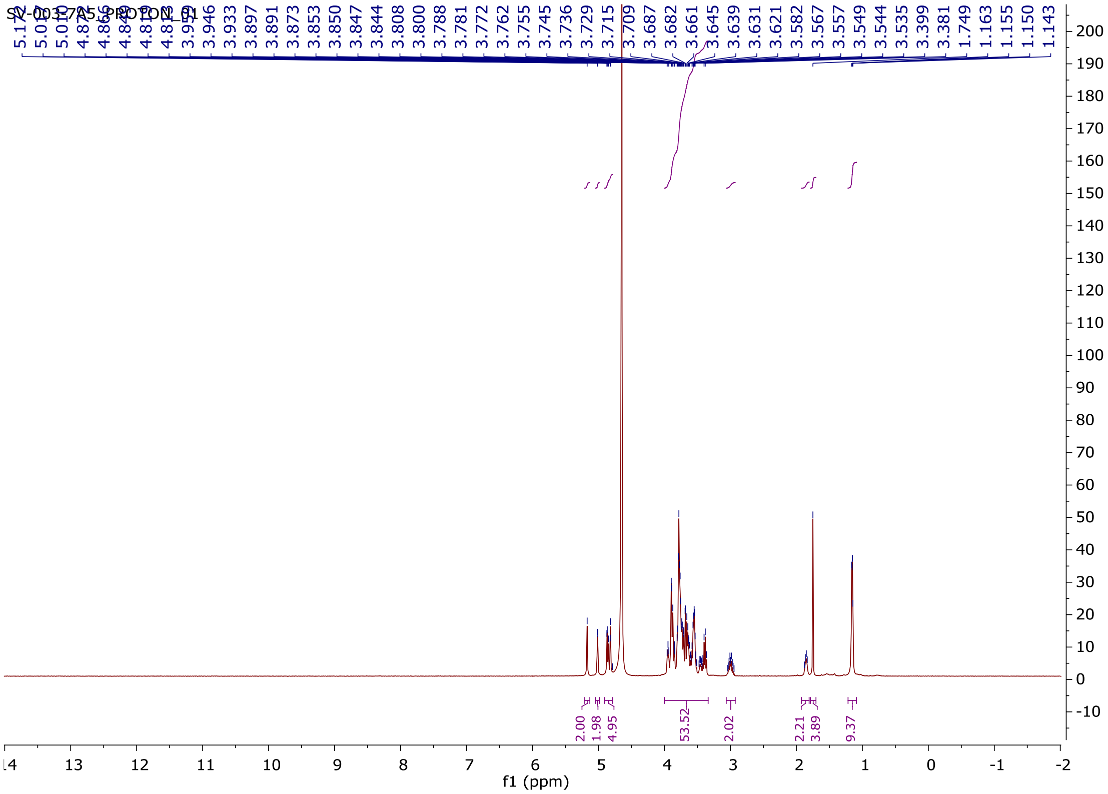

^{1}H NMR spectrum of compound **3**

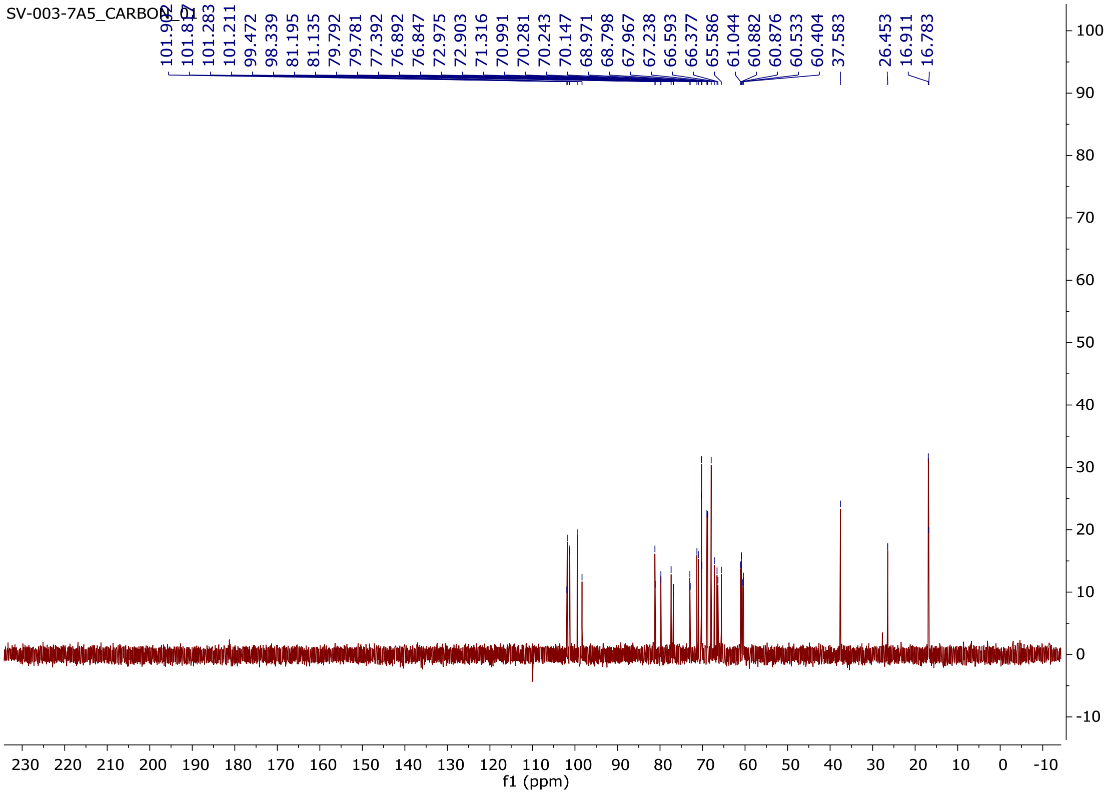

^{13}C NMR spectrum of compound **3**

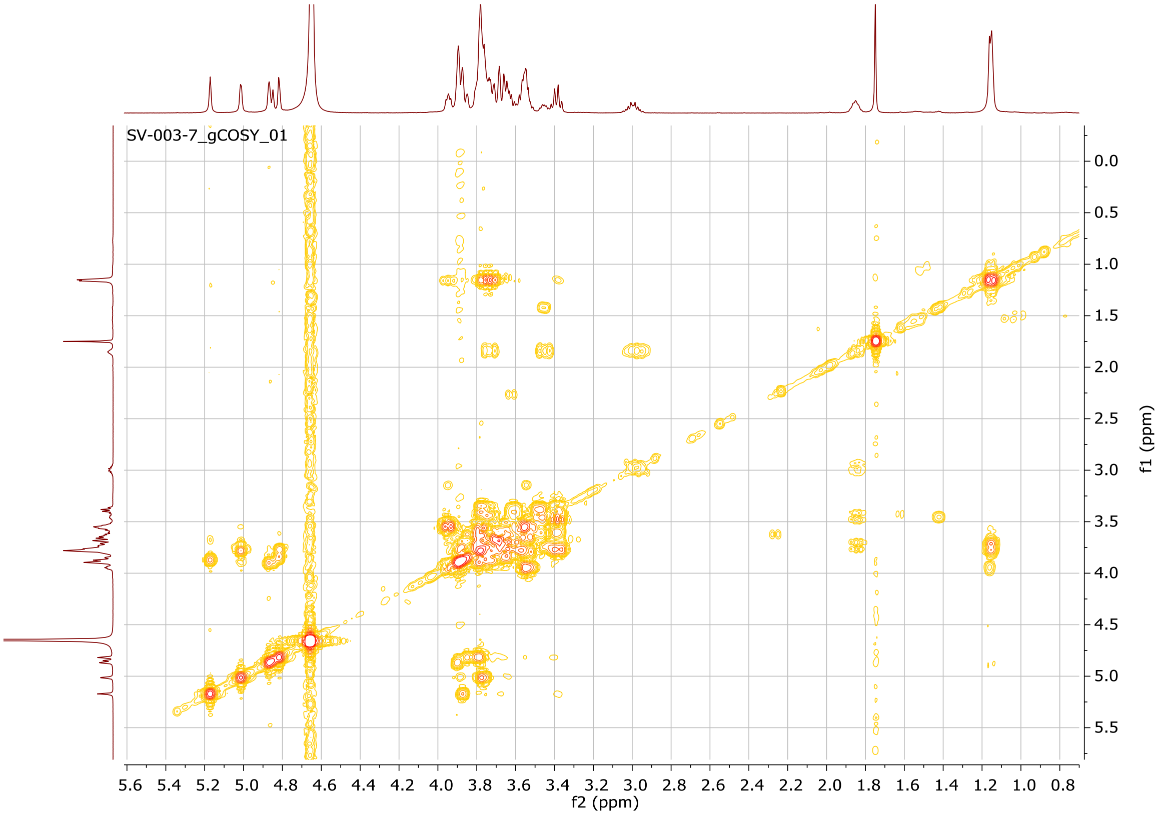

gCOSY NMR spectrum of compound **3**

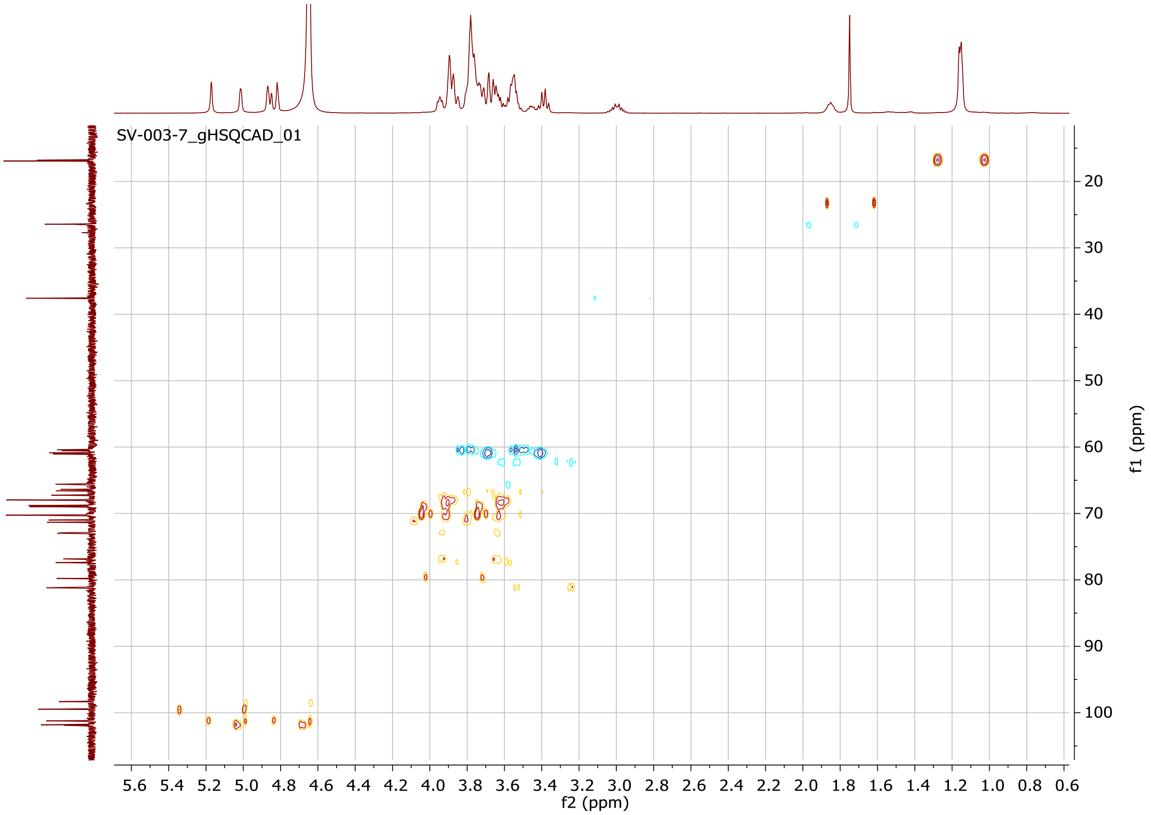

^{1}H-Coupled gHSQC NMR spectrum of compound **3**

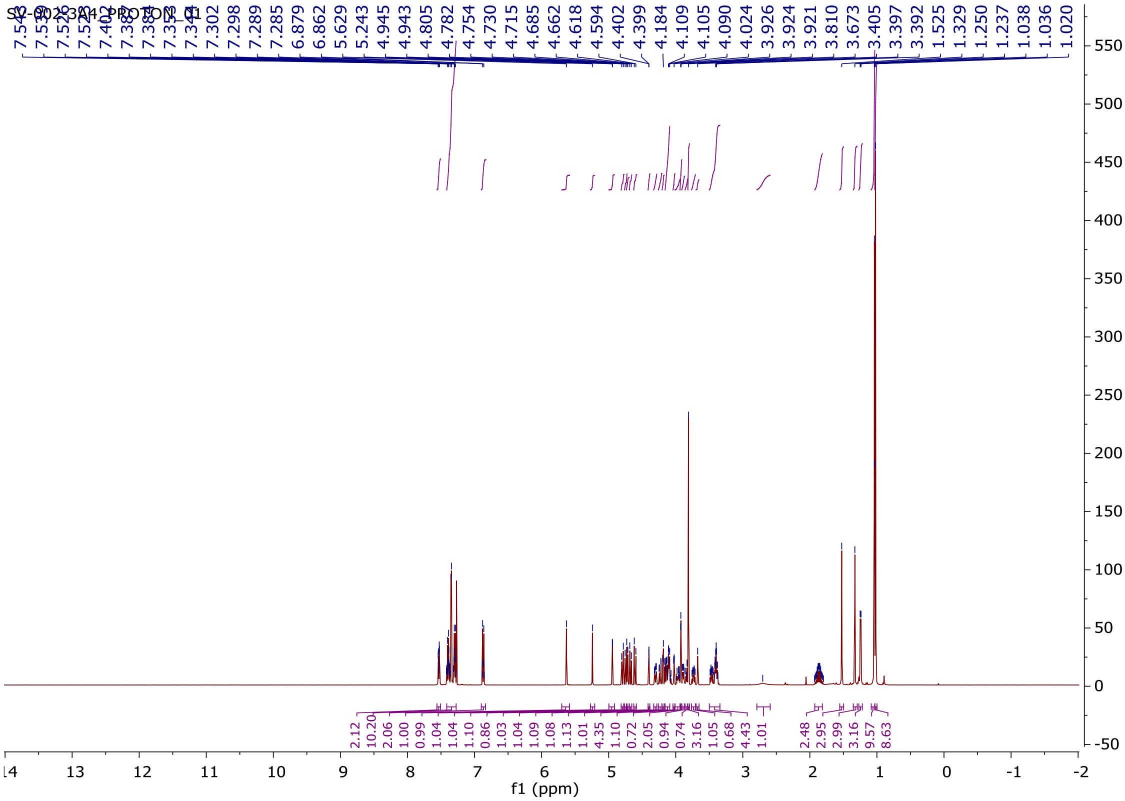

^{1}H NMR spectrum of compound **6**

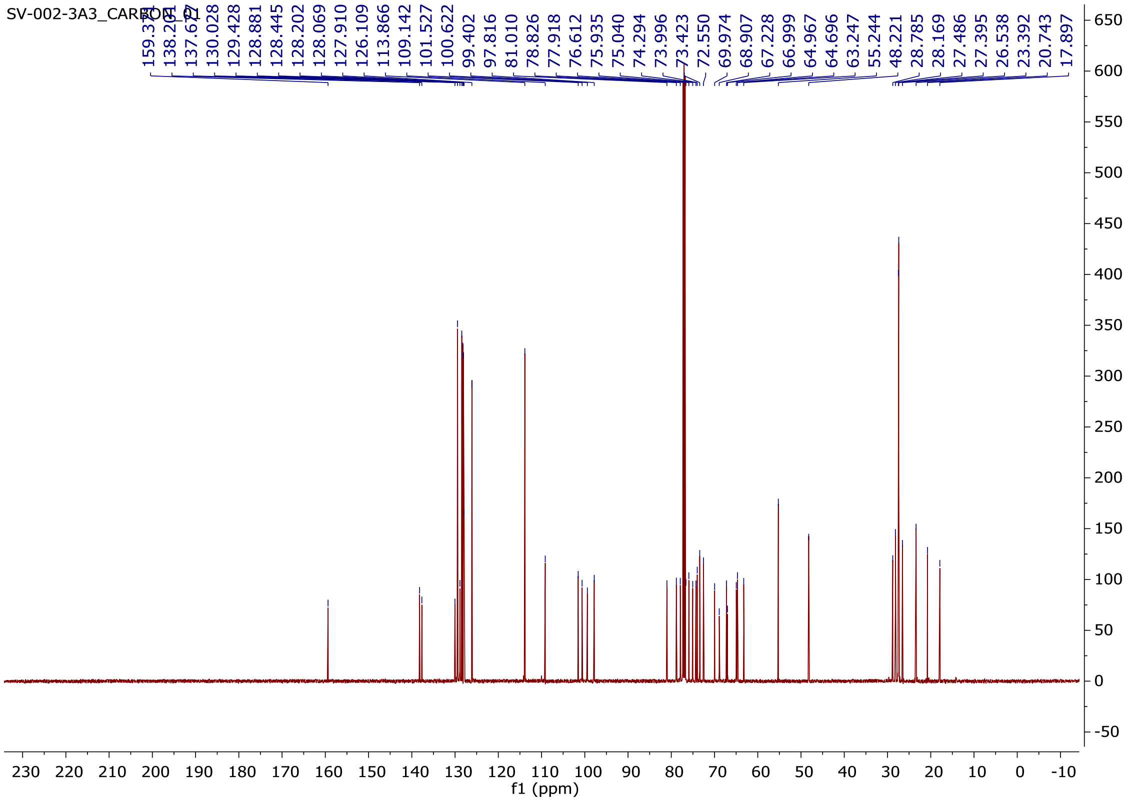

^{13}C NMR spectrum of compound **6**

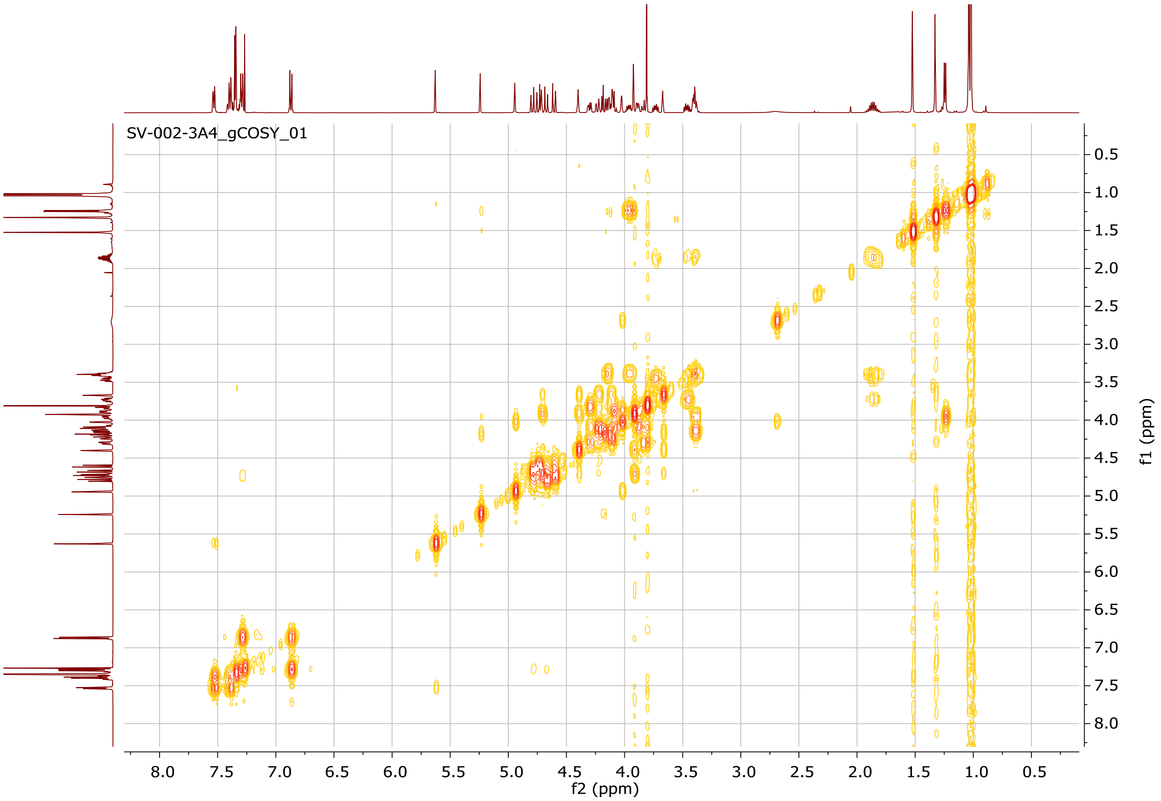

gCOSY NMR spectrum of compound **6**

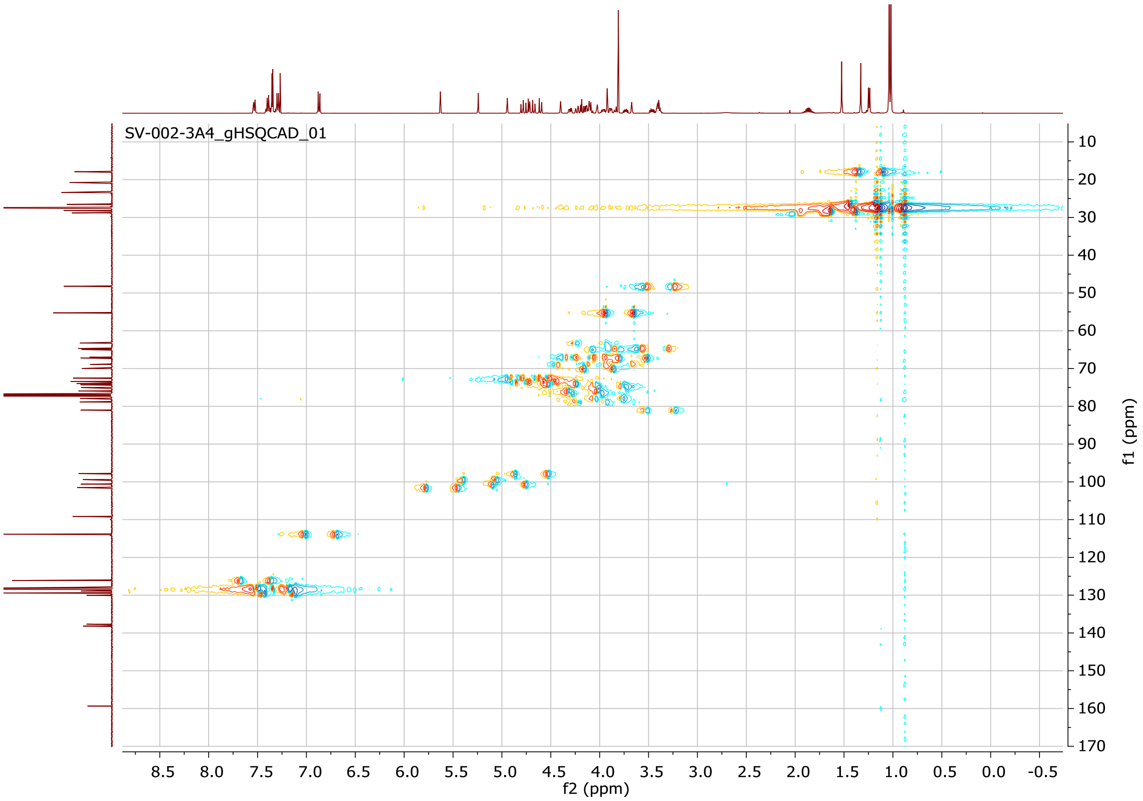

^{1}H-Coupled gHSQC NMR spectrum of compound **6**

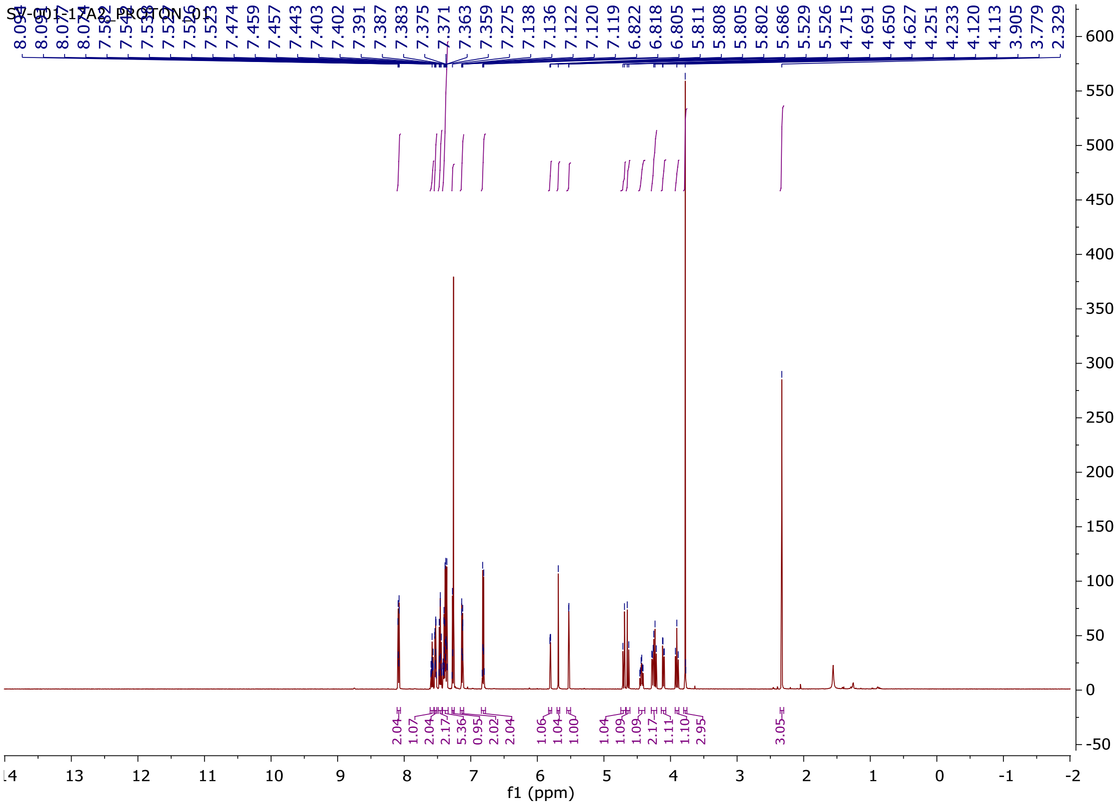

^{1}H NMR spectrum of compound **7**

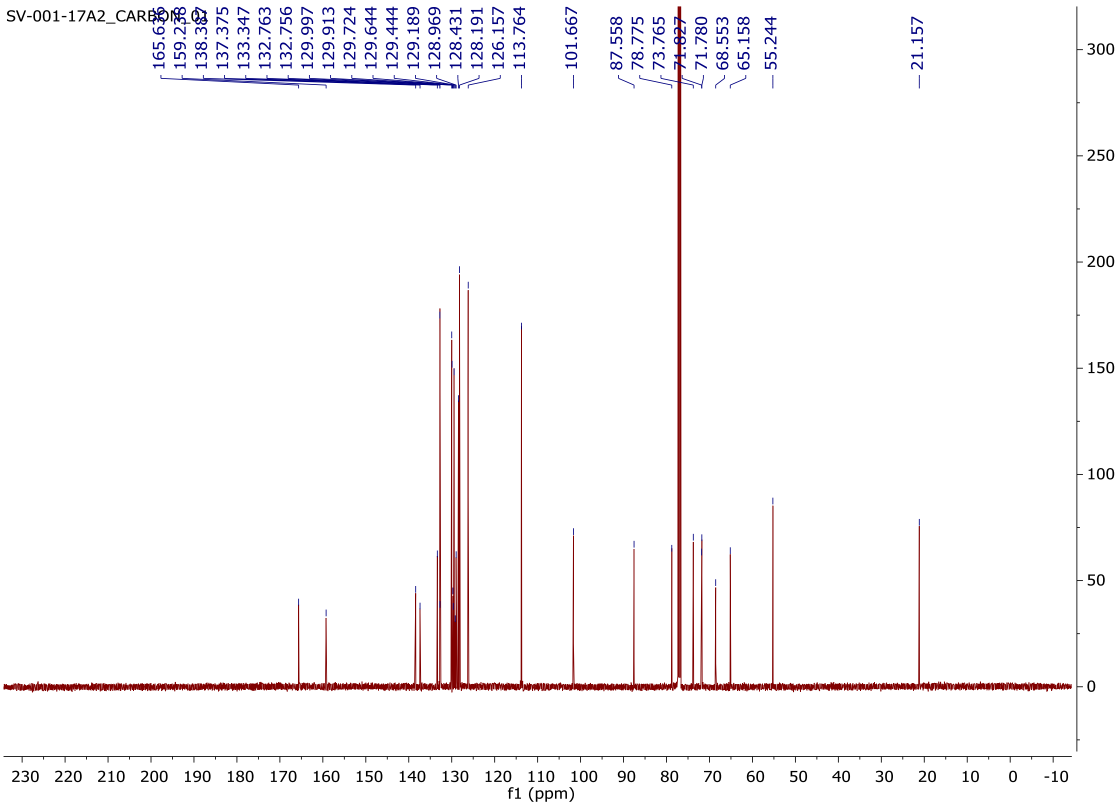

^{13}C NMR spectrum of compound **7**

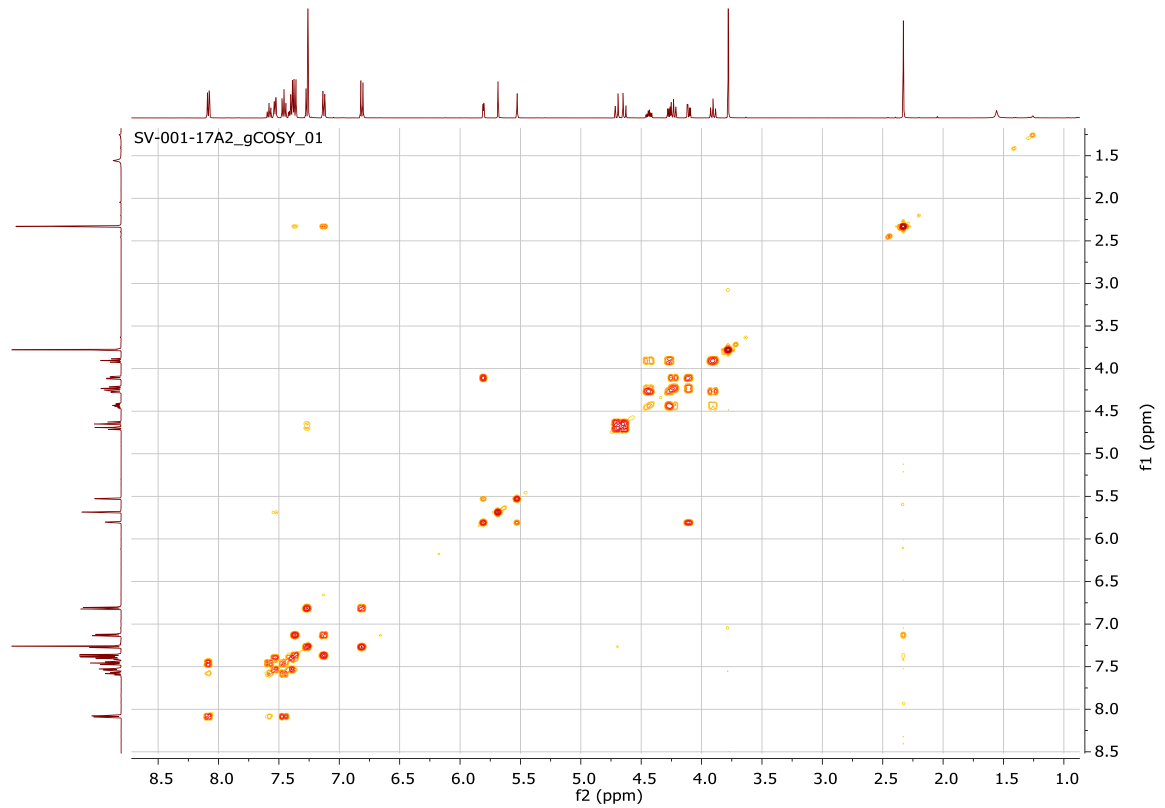

gCOSY NMR spectrum of compound **7**

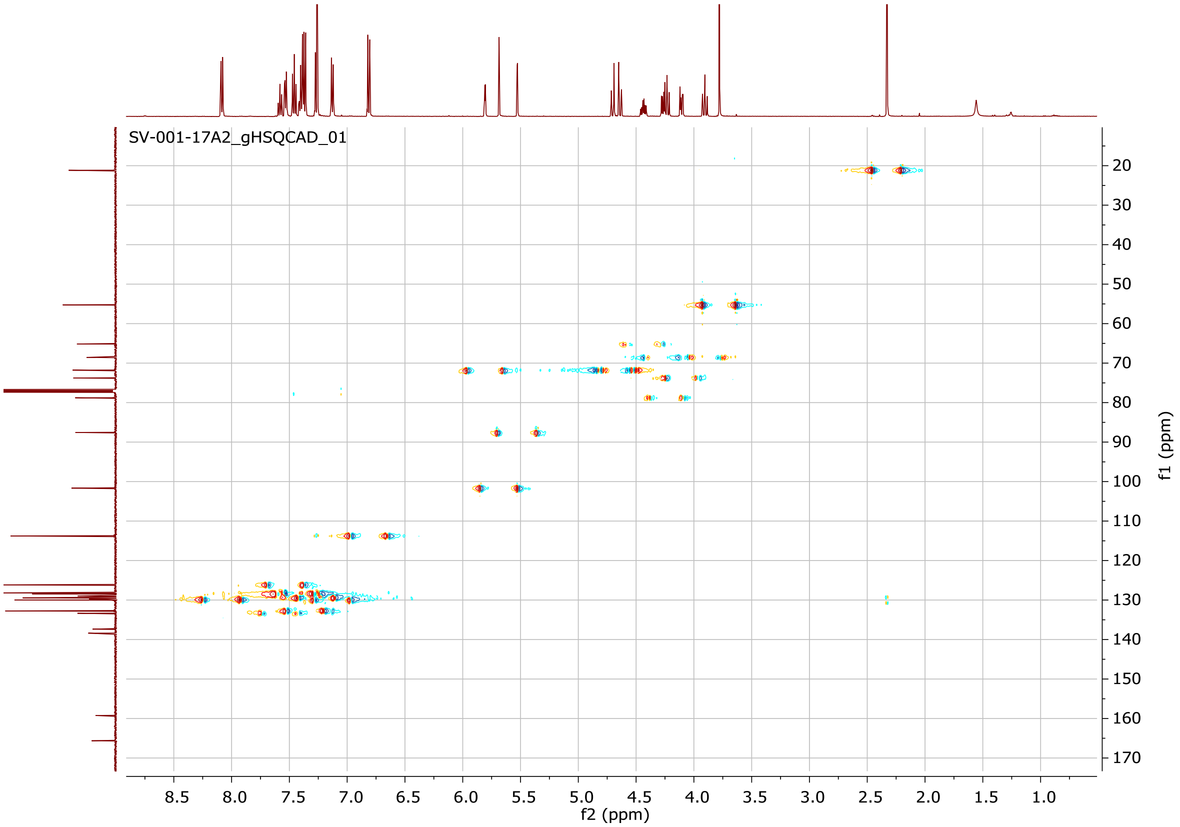

^{1}H-Coupled gHSQC NMR spectrum of compound **7**

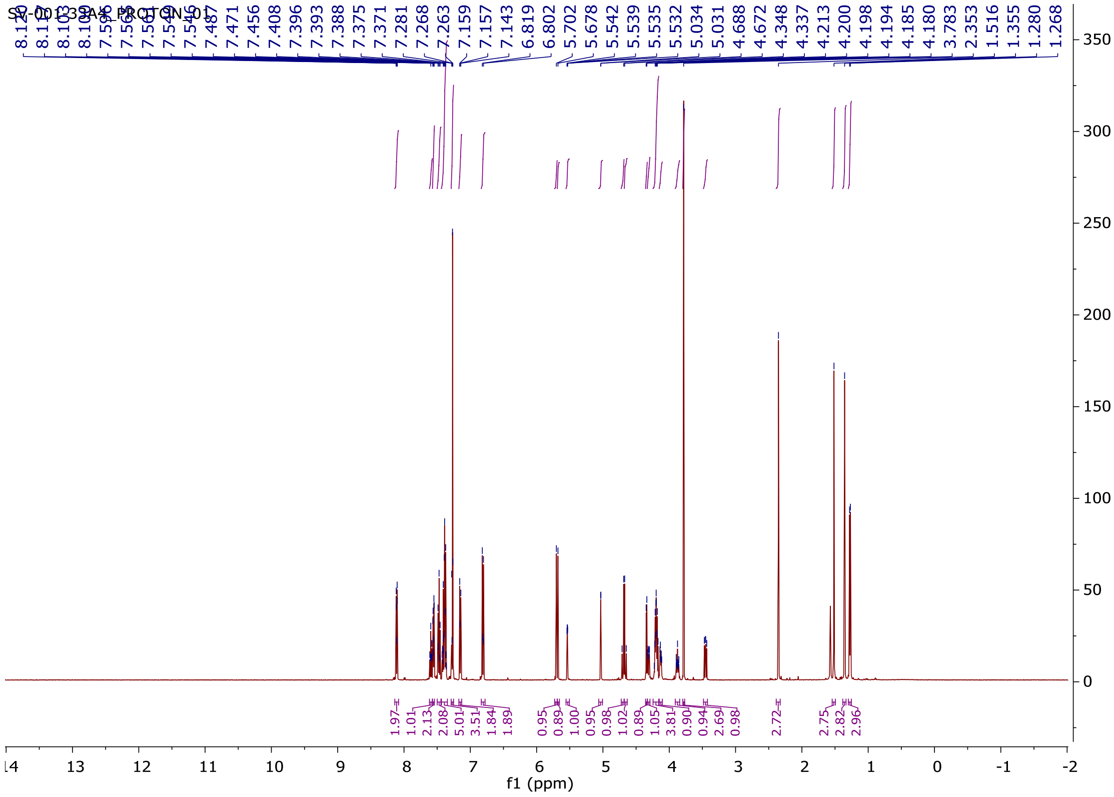

^{1}H NMR spectrum of compound **11**

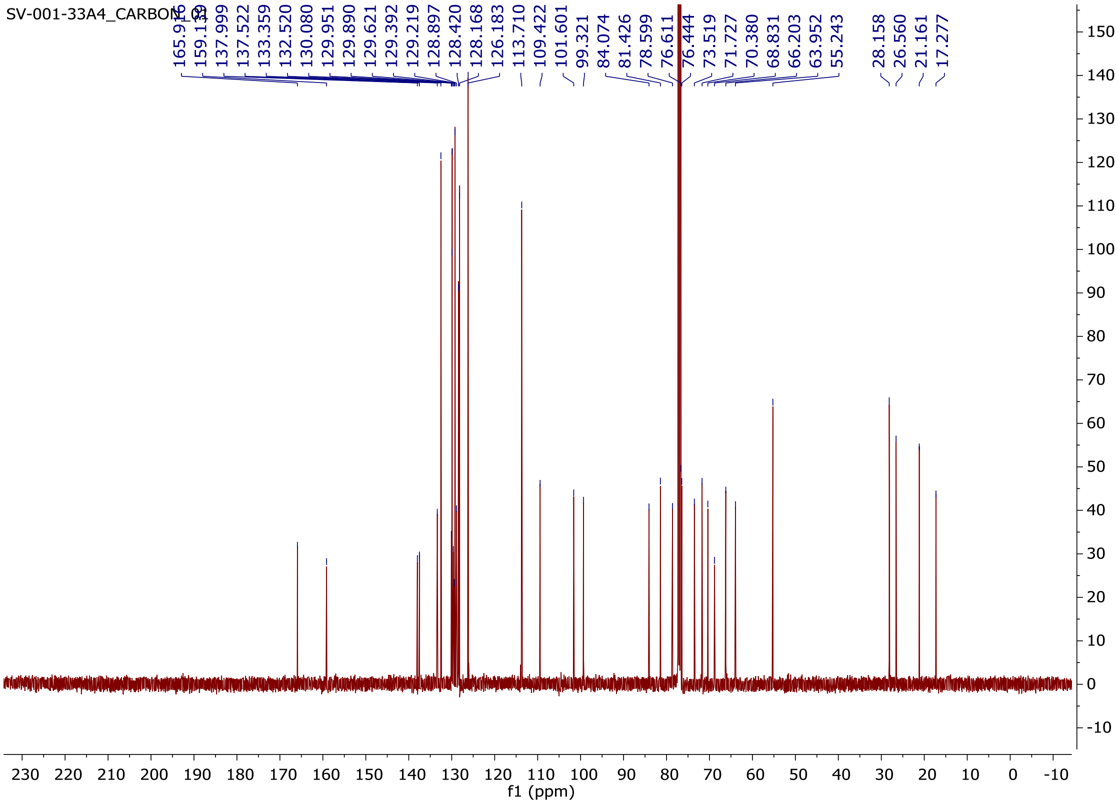

^{13}C NMR spectrum of compound **11**

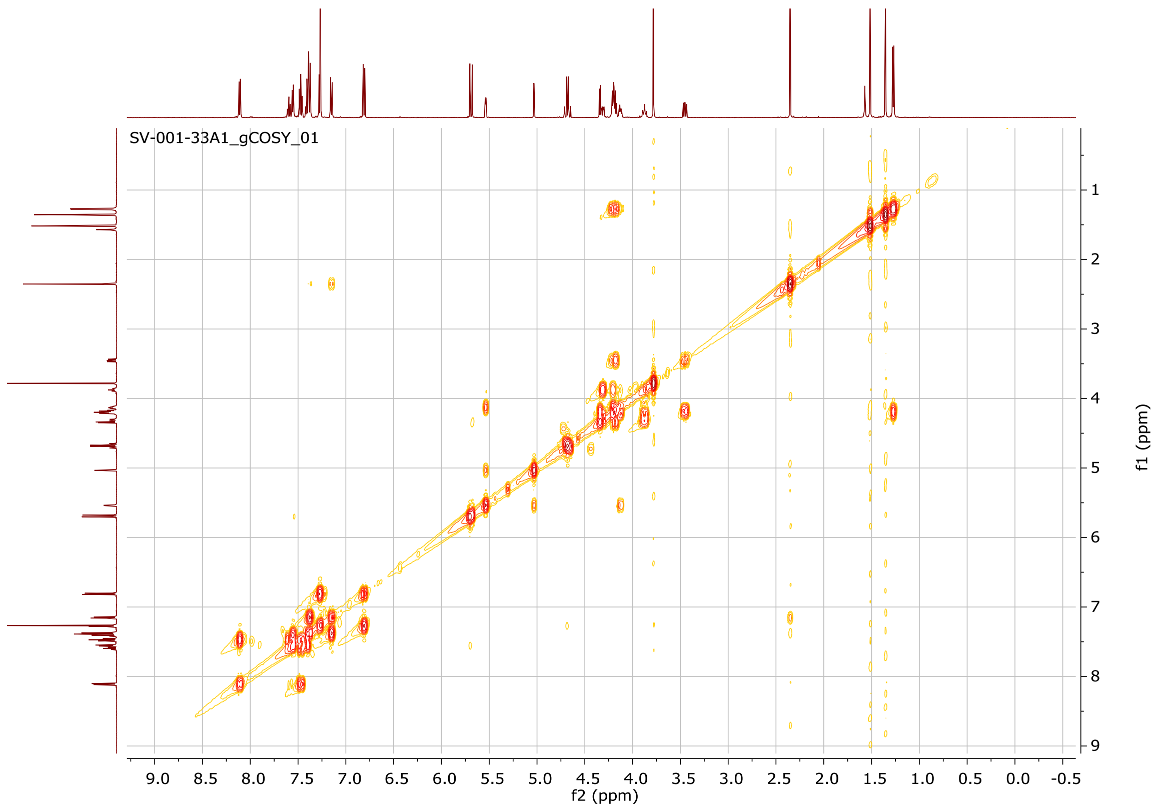

gCOSY NMR spectrum of compound **11**

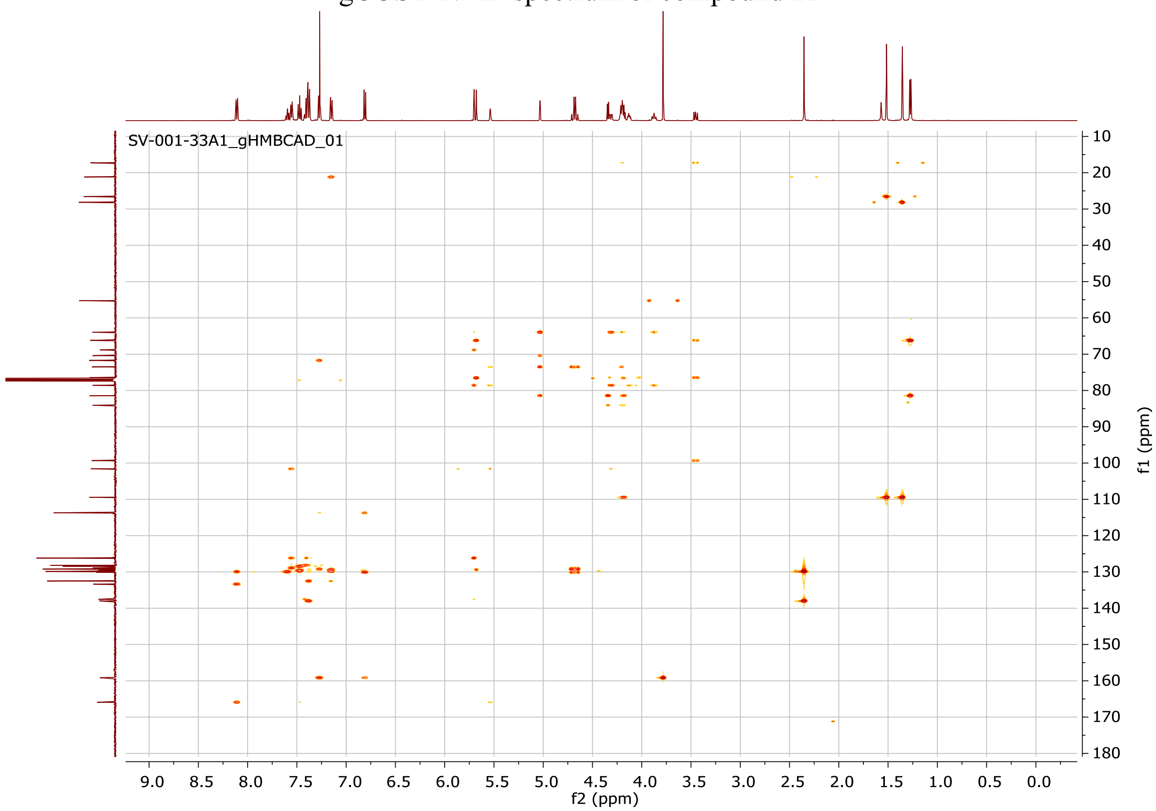

gHMBC NMR spectrum of compound **11**

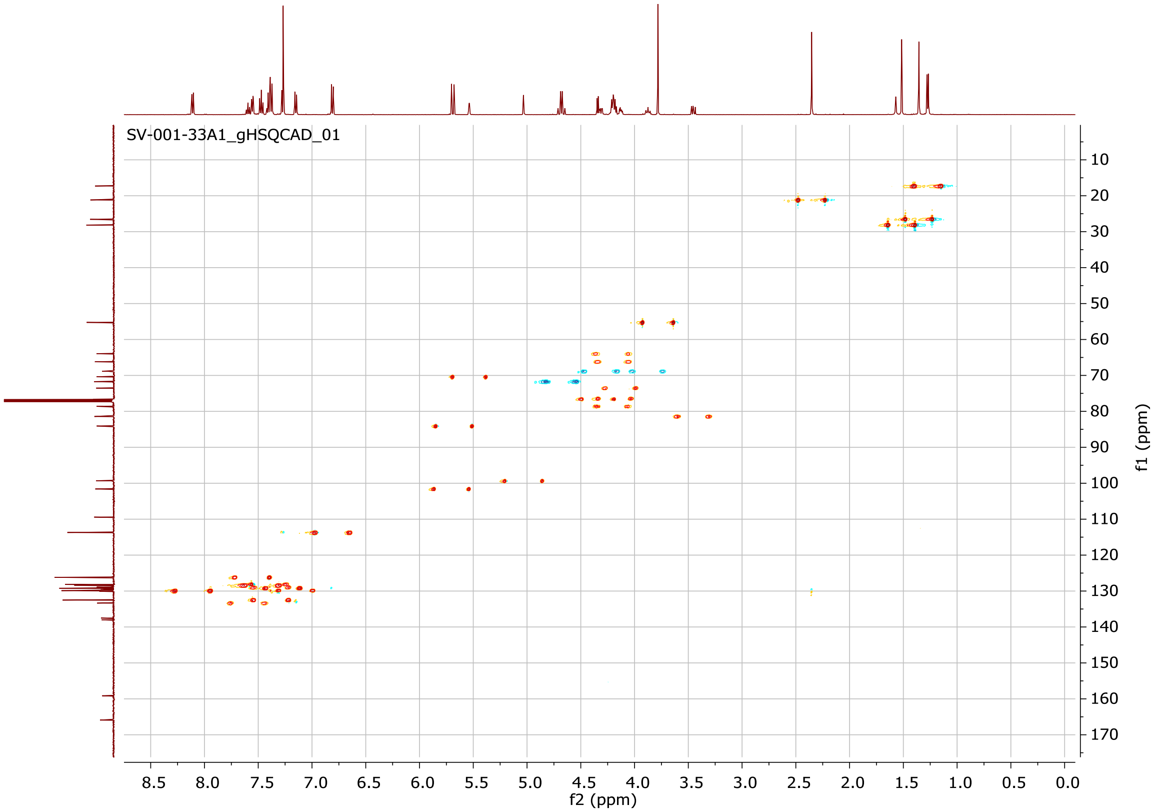

^{1}H-Coupled gHSQC NMR spectrum of compound **11**

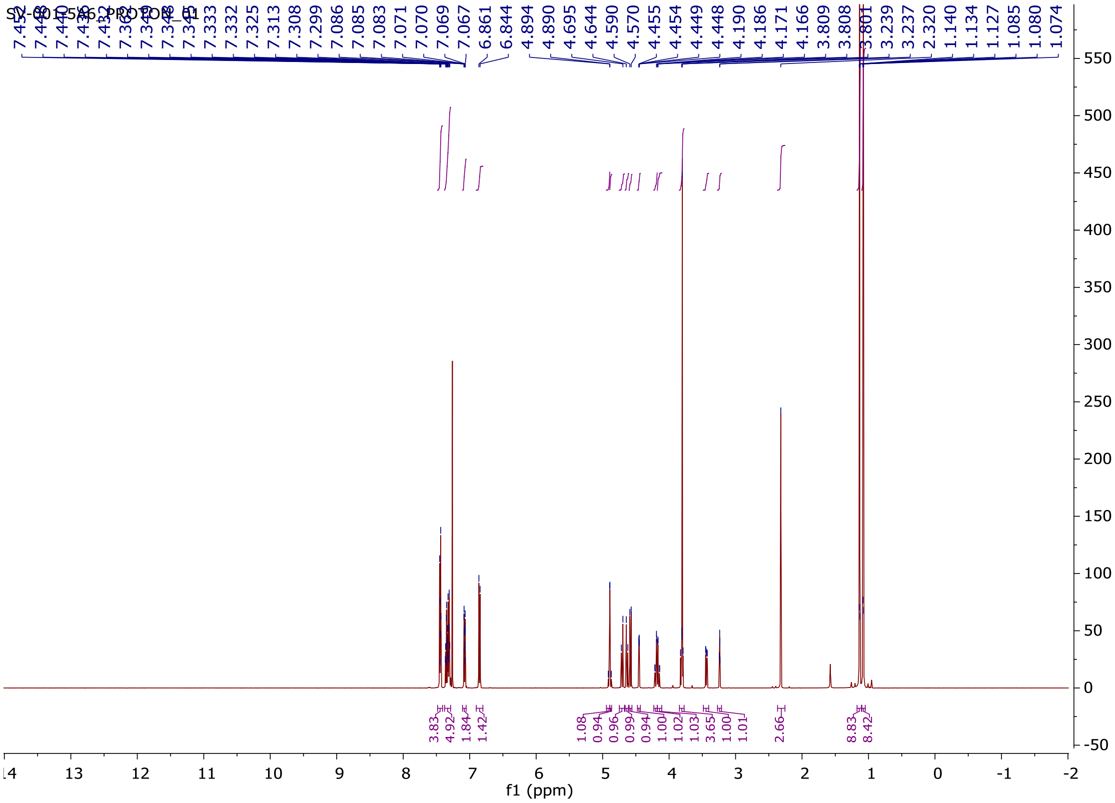

^{1}H NMR spectrum of compound **13**

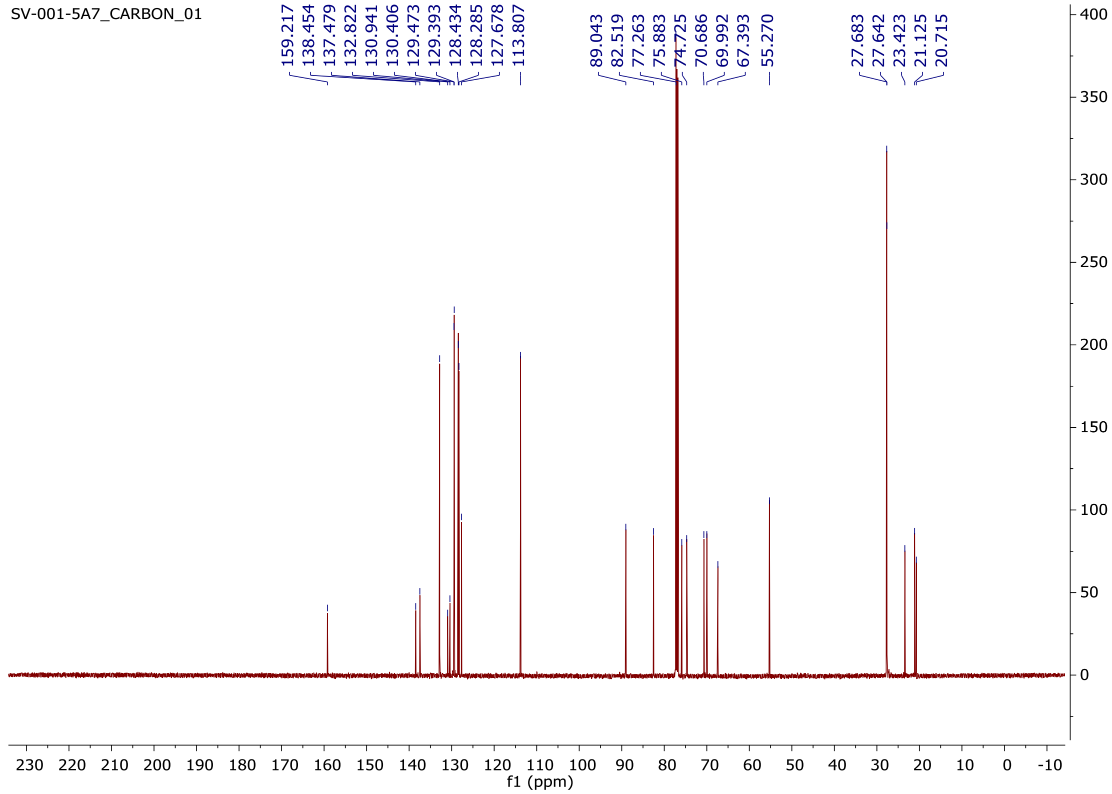

^{13}C NMR spectrum of compound **13**

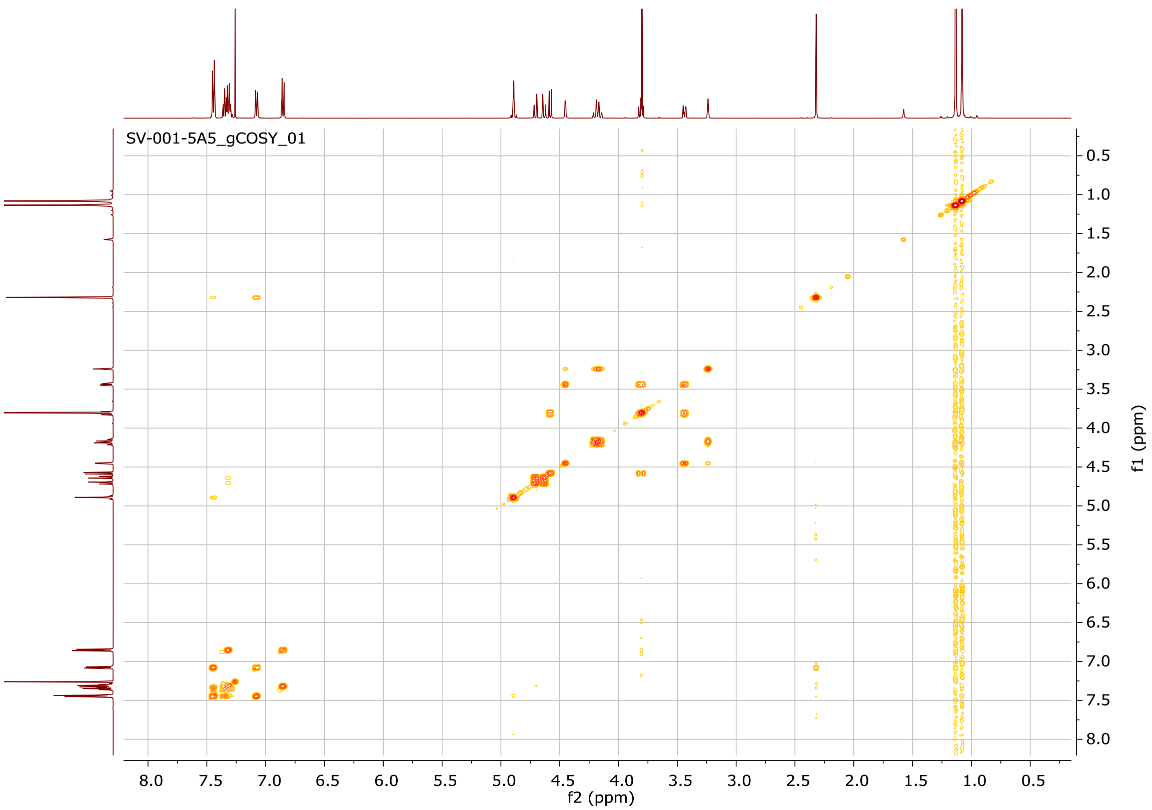

gCOSY NMR spectrum of compound **13**

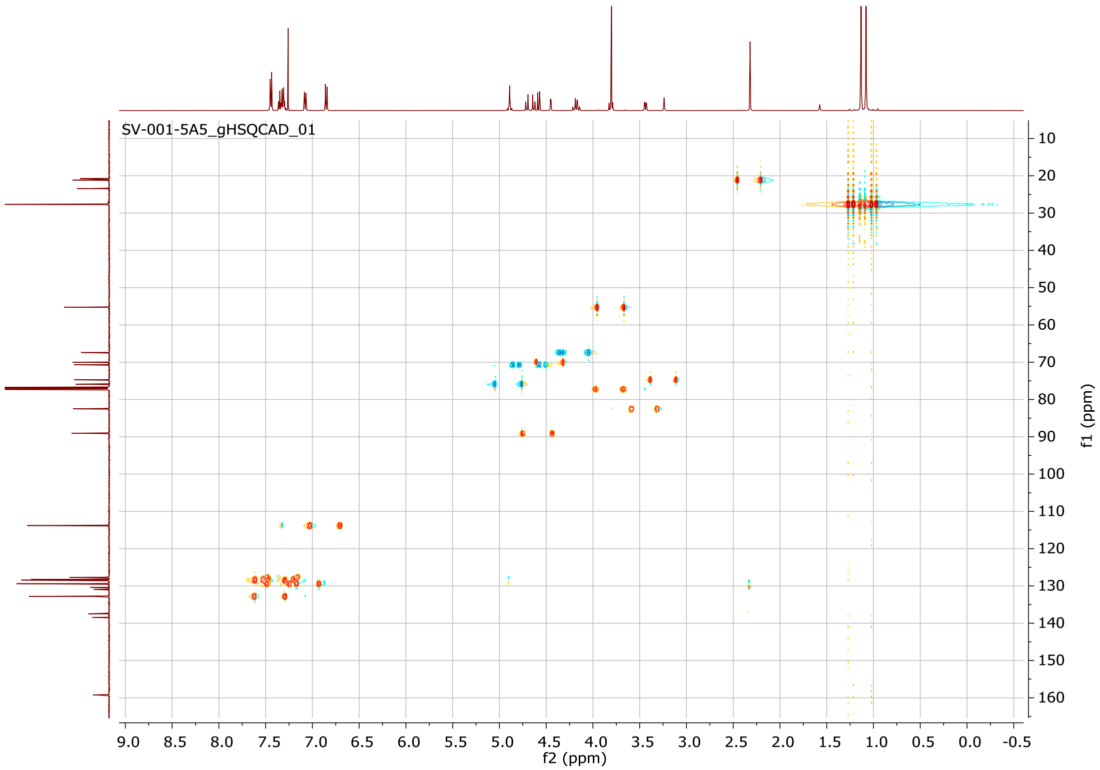

^{1}H-Coupled gHSQC NMR spectrum of compound **13**

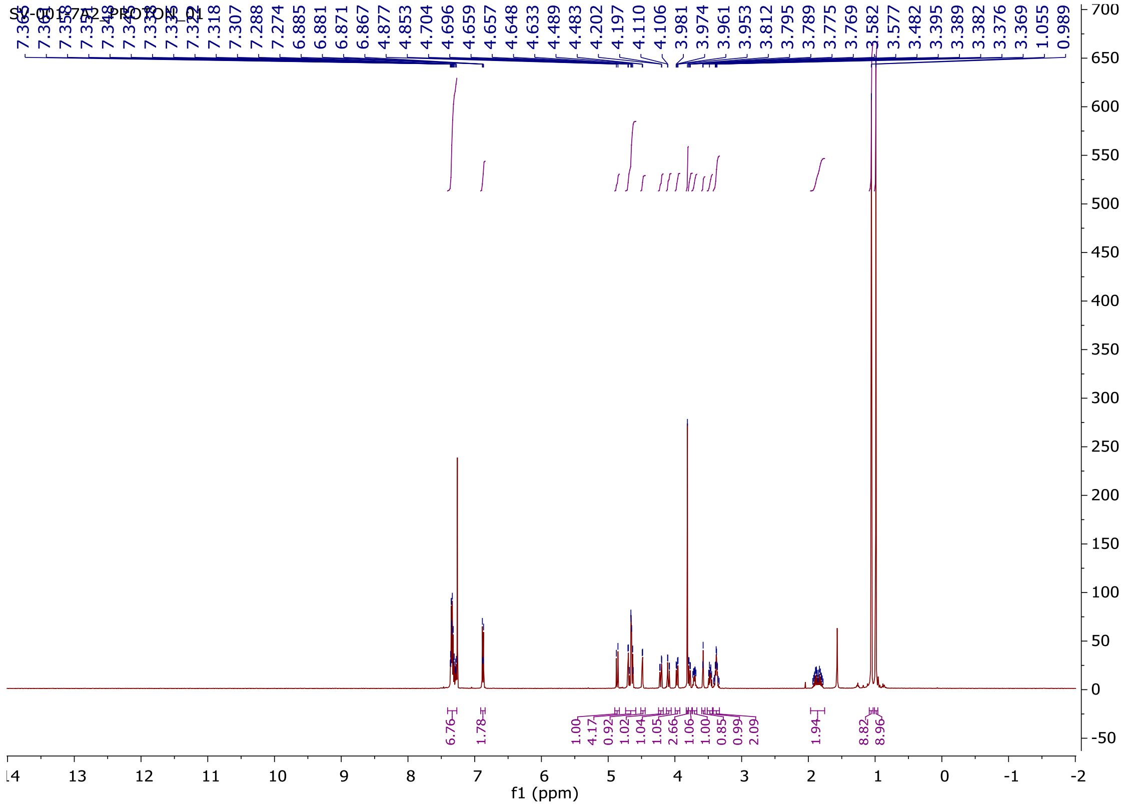

^{1}H NMR spectrum of compound **14**

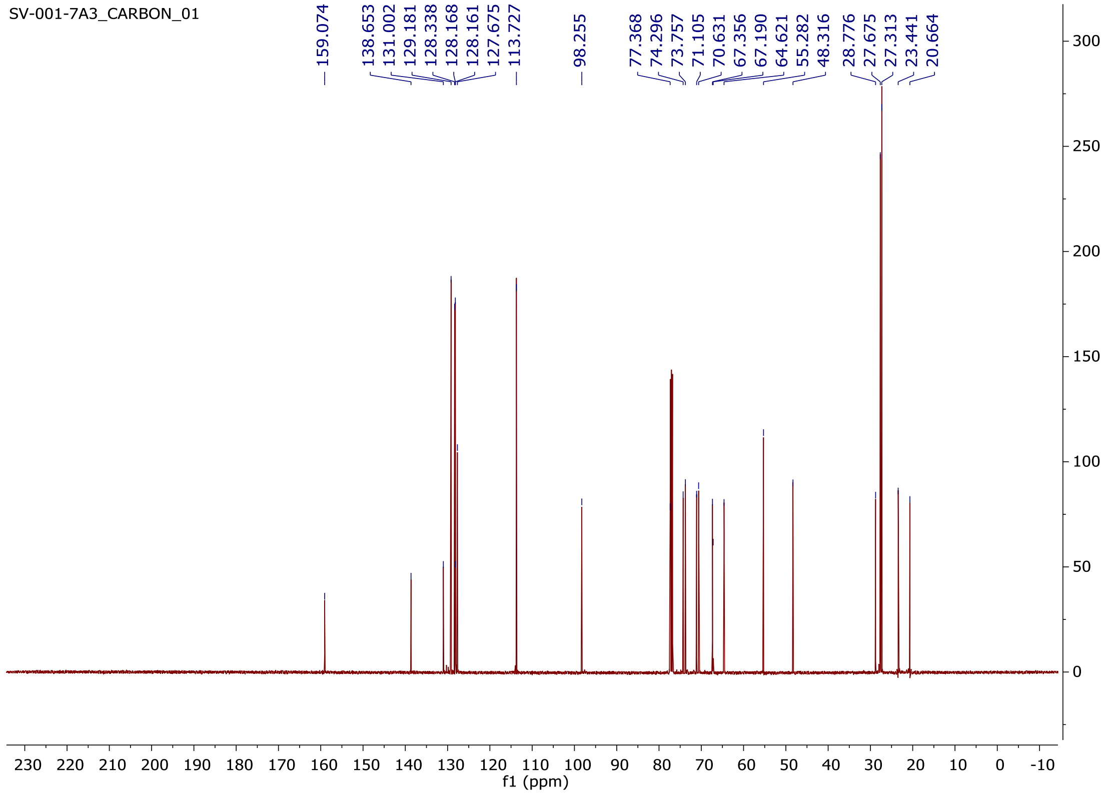

^{13}C NMR spectrum of compound **14**

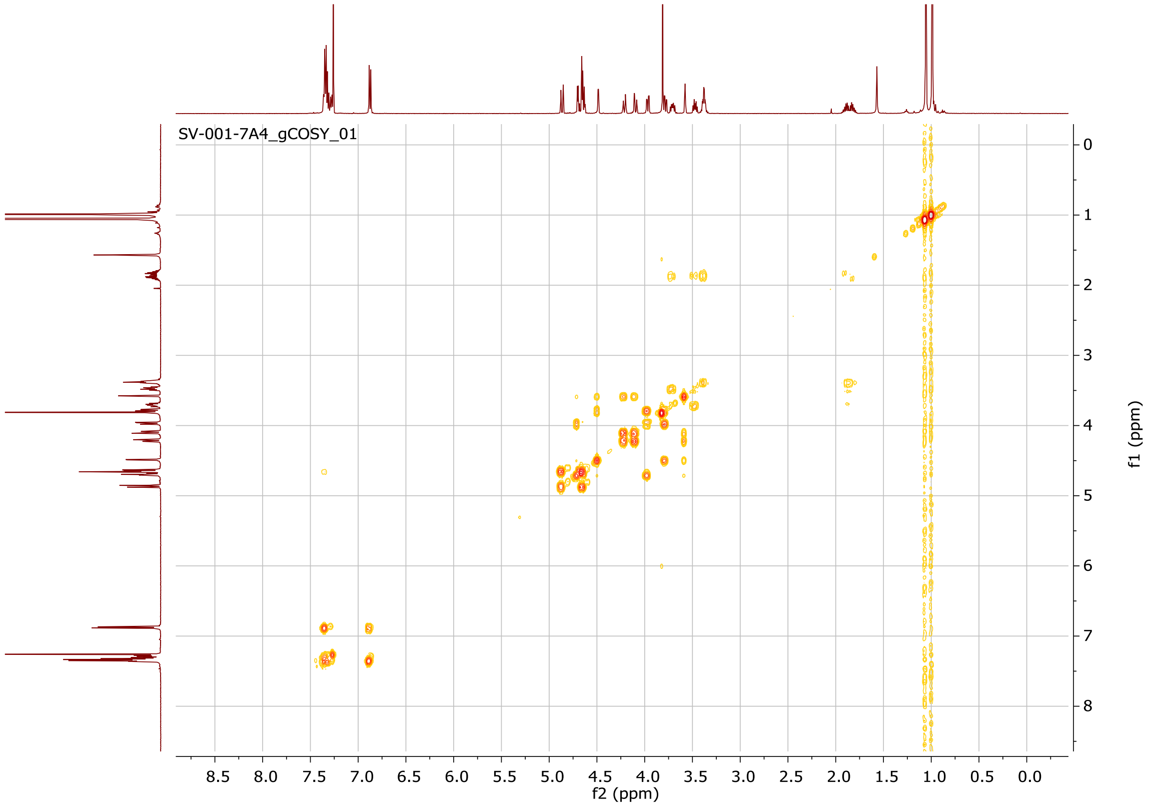

gCOSY NMR spectrum of compound **14**

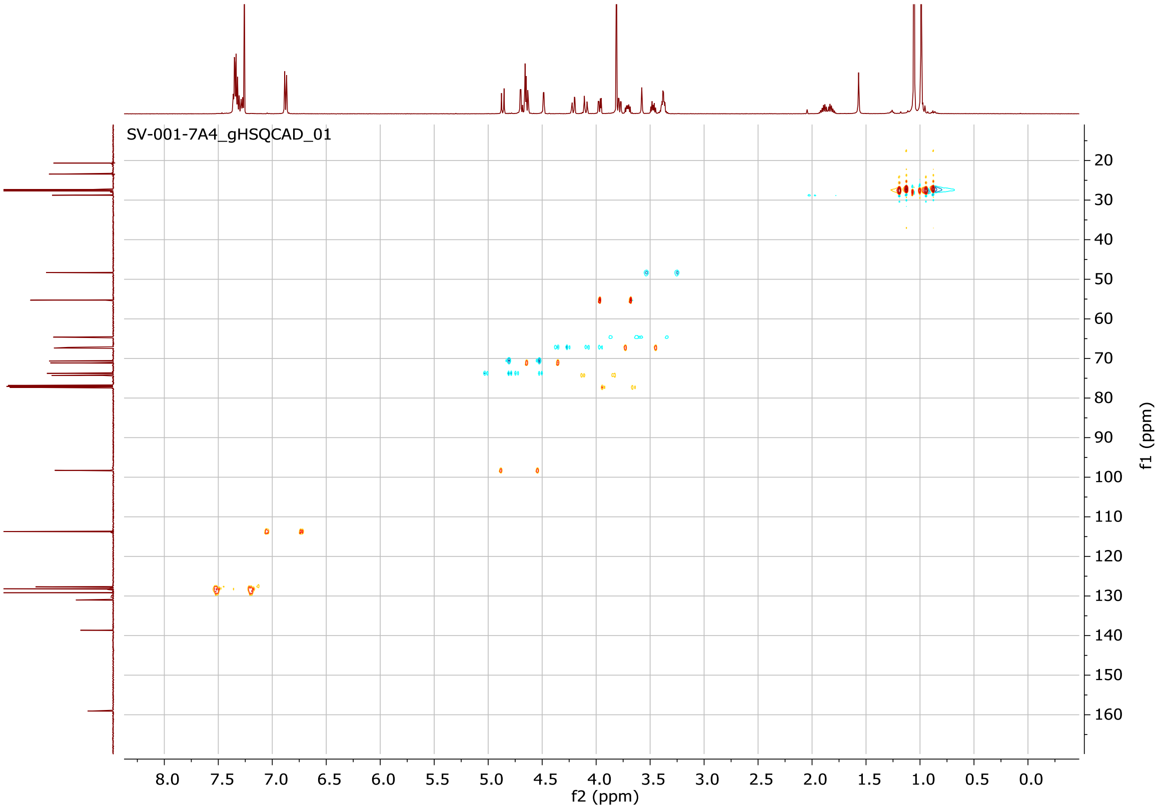

^{1}H-Coupled gHSQC NMR spectrum of compound **14**

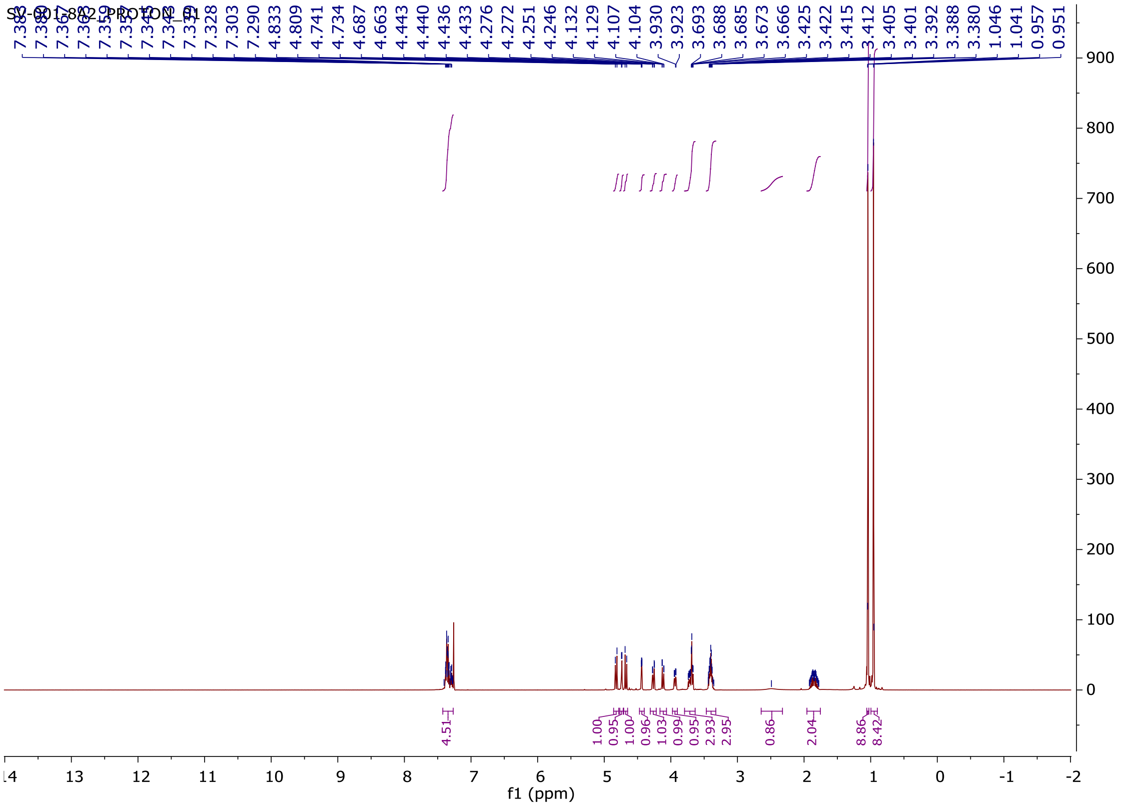

^{1}H NMR spectrum of compound **15**

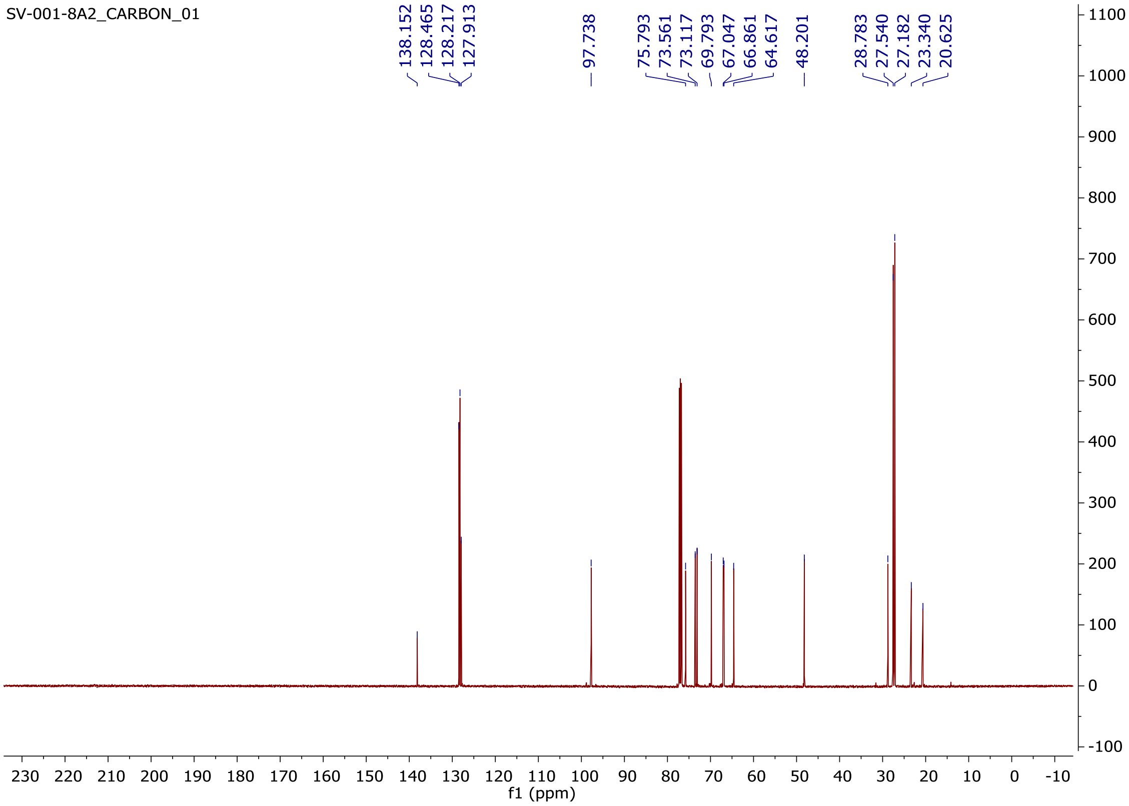

^{13}C NMR spectrum of compound **15**

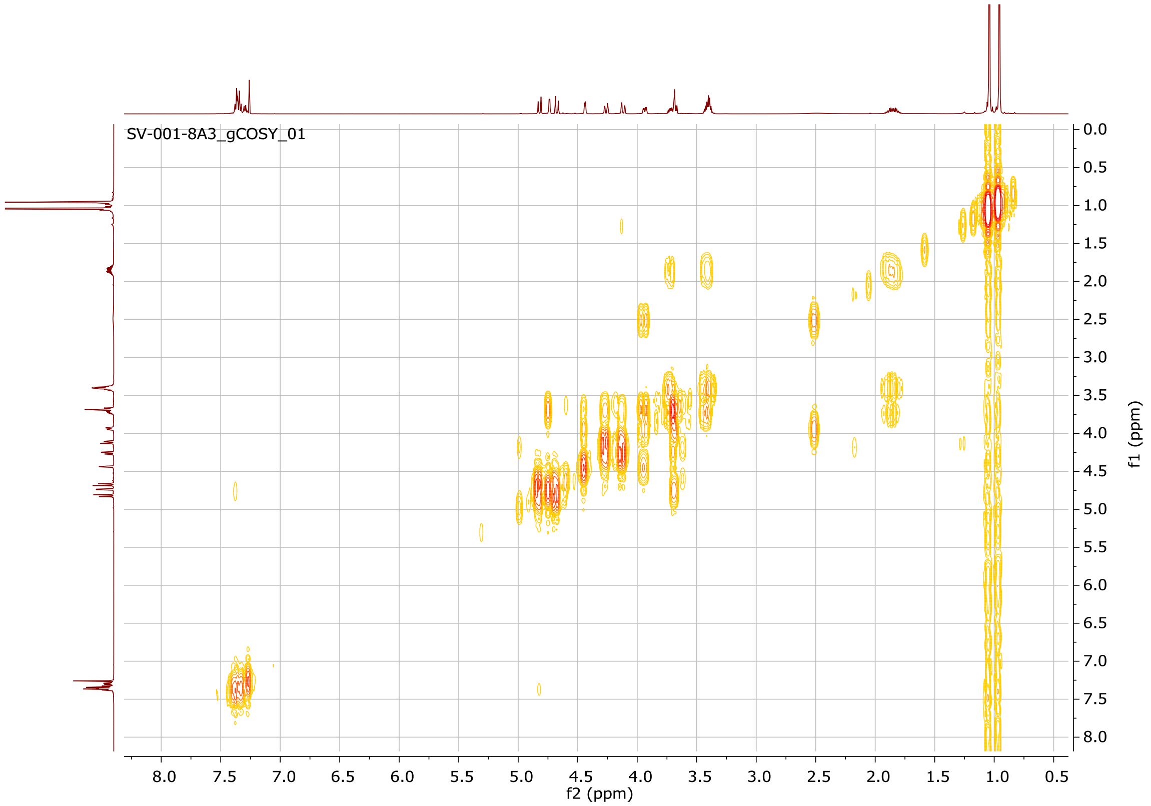

gCOSY NMR spectrum of compound **15**

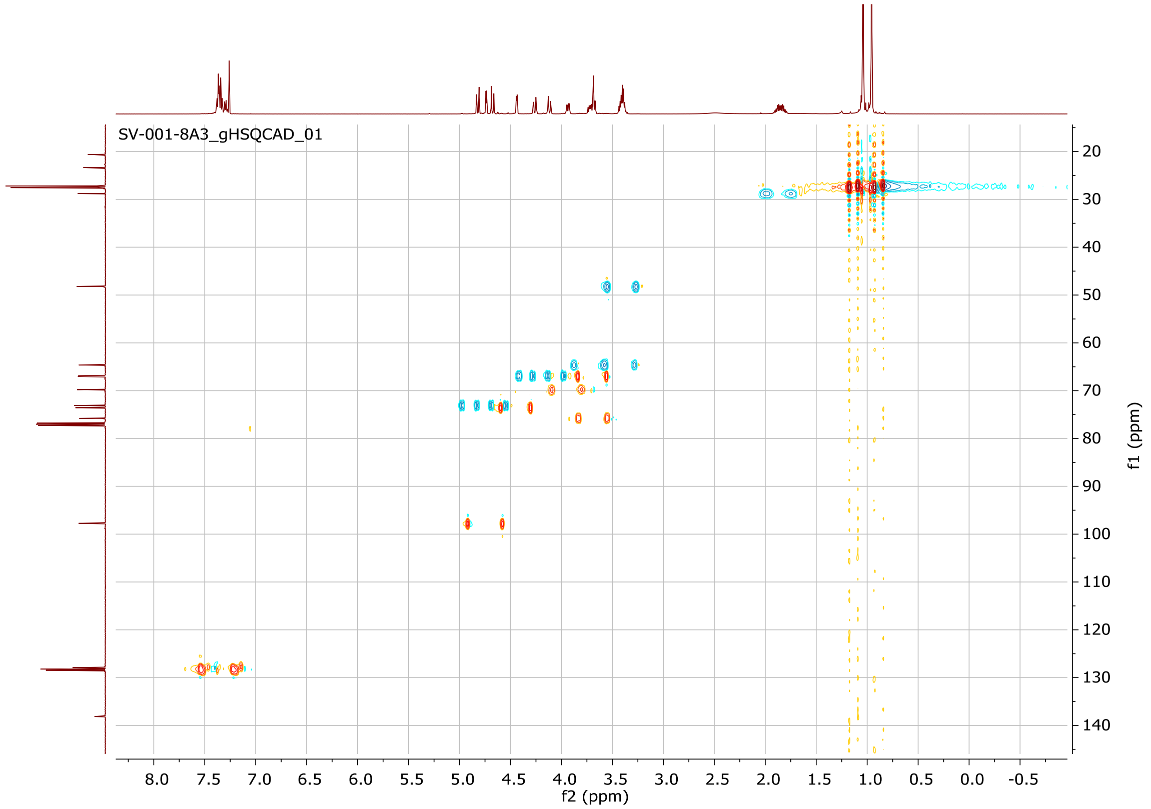

^{1}H-Coupled gHSQC NMR spectrum of compound **15**

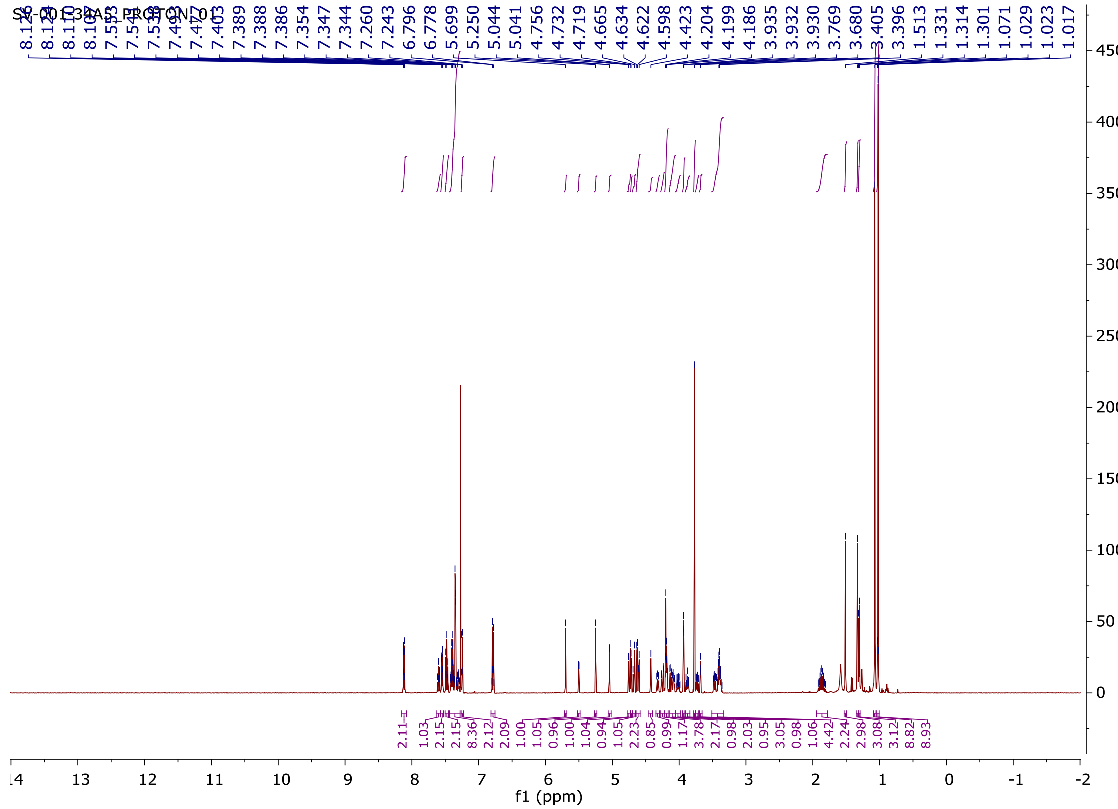

^{1}H NMR spectrum of compound **16**

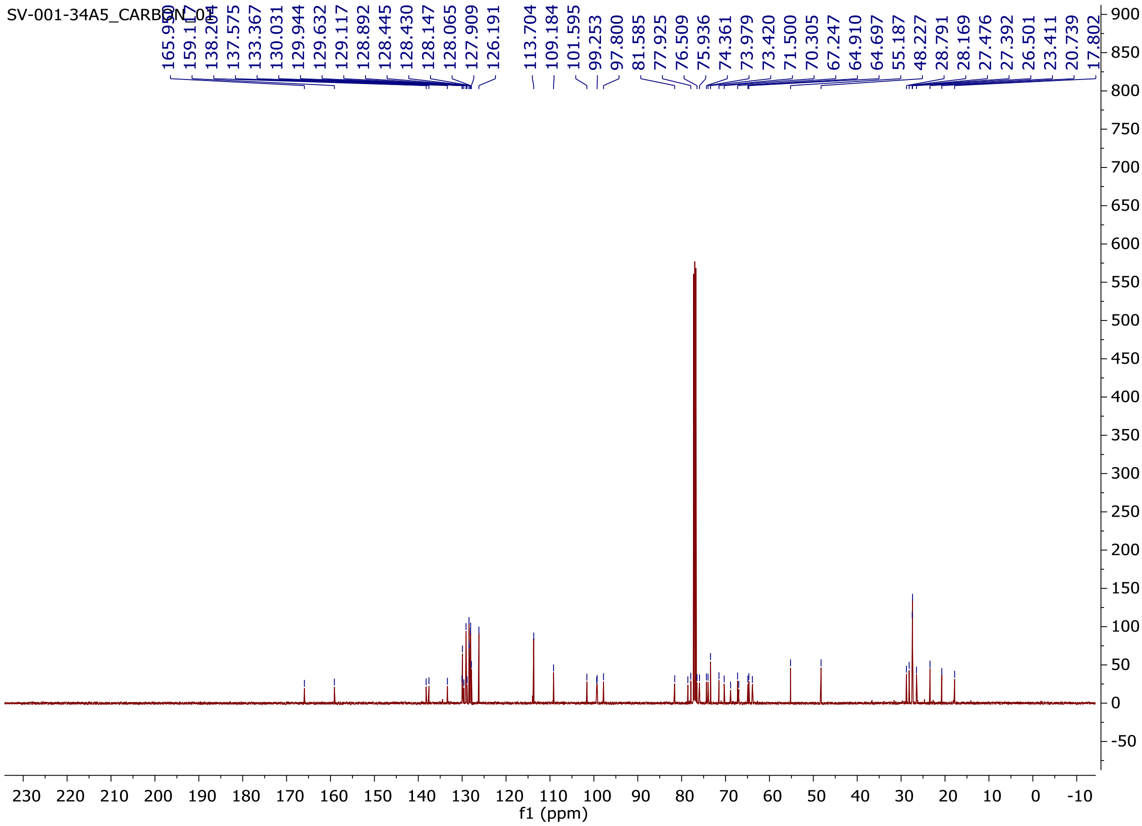

^{13}C NMR spectrum of compound **16**

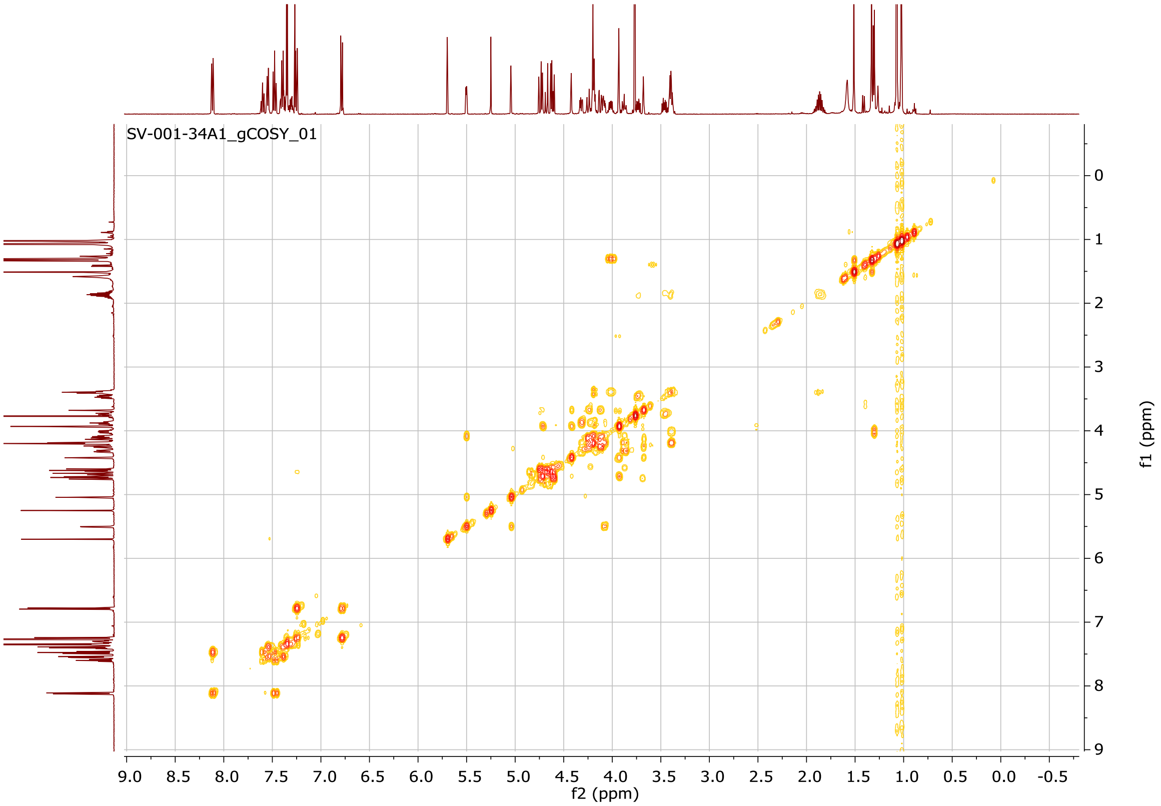

gCOSY NMR spectrum of compound **16**

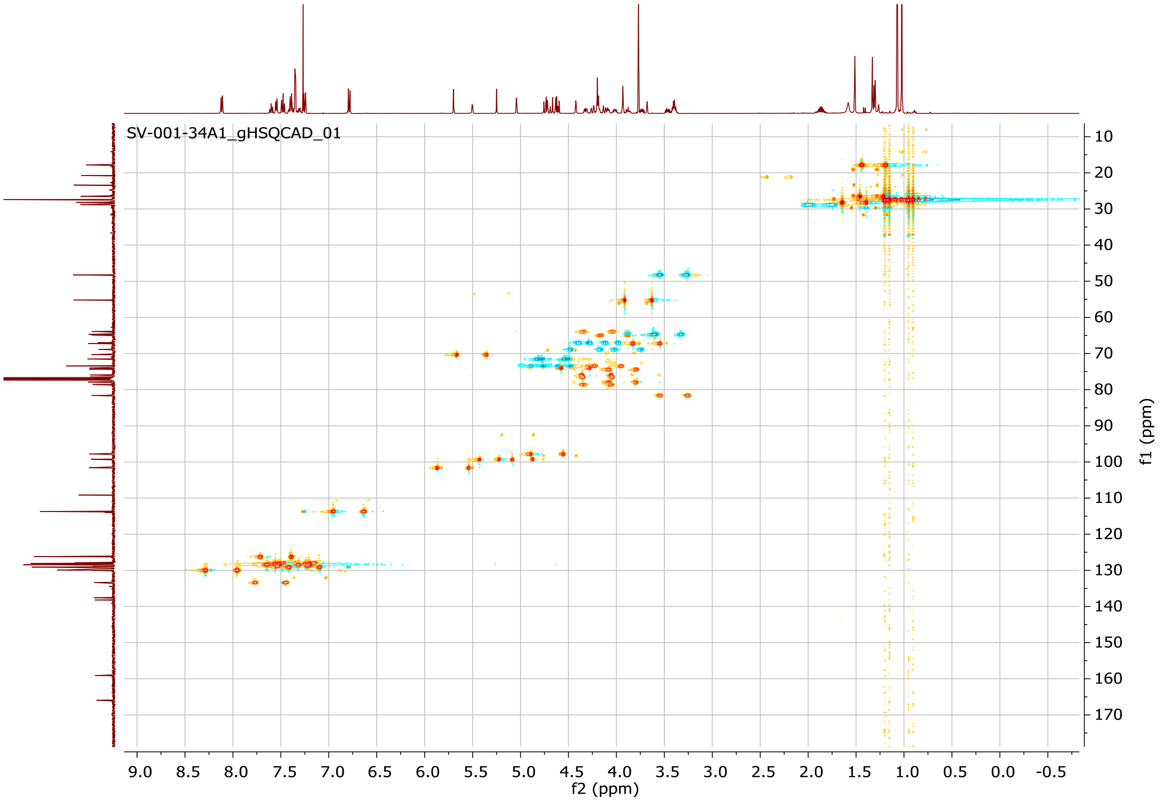

^{1}H-Coupled gHSQC NMR spectrum of compound **16**

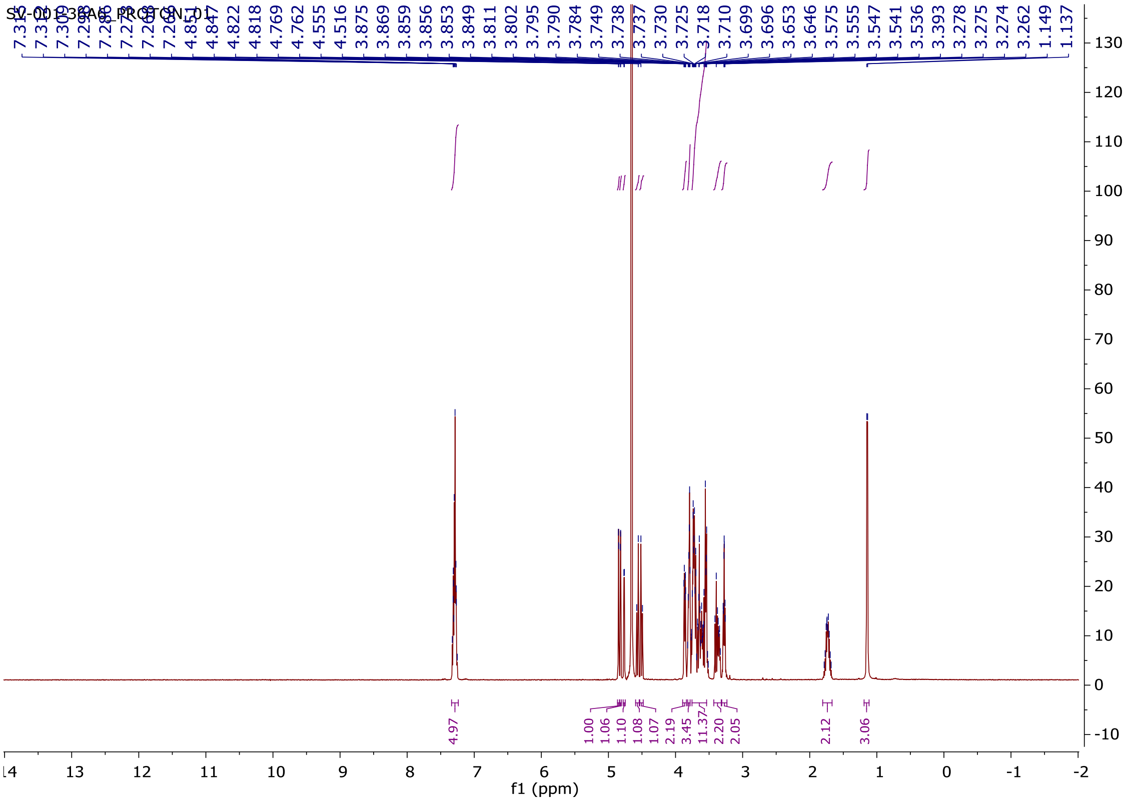

^{1}H NMR spectrum of compound **17**

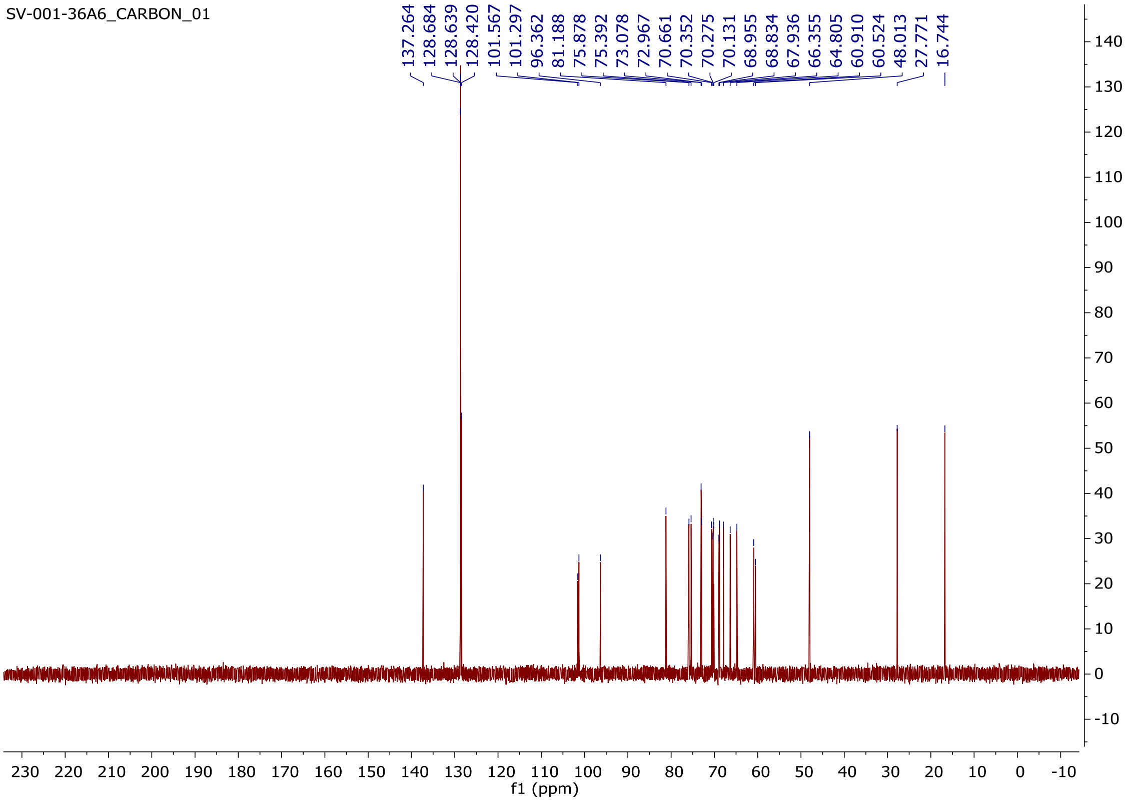

^{13}C NMR spectrum of compound **17**

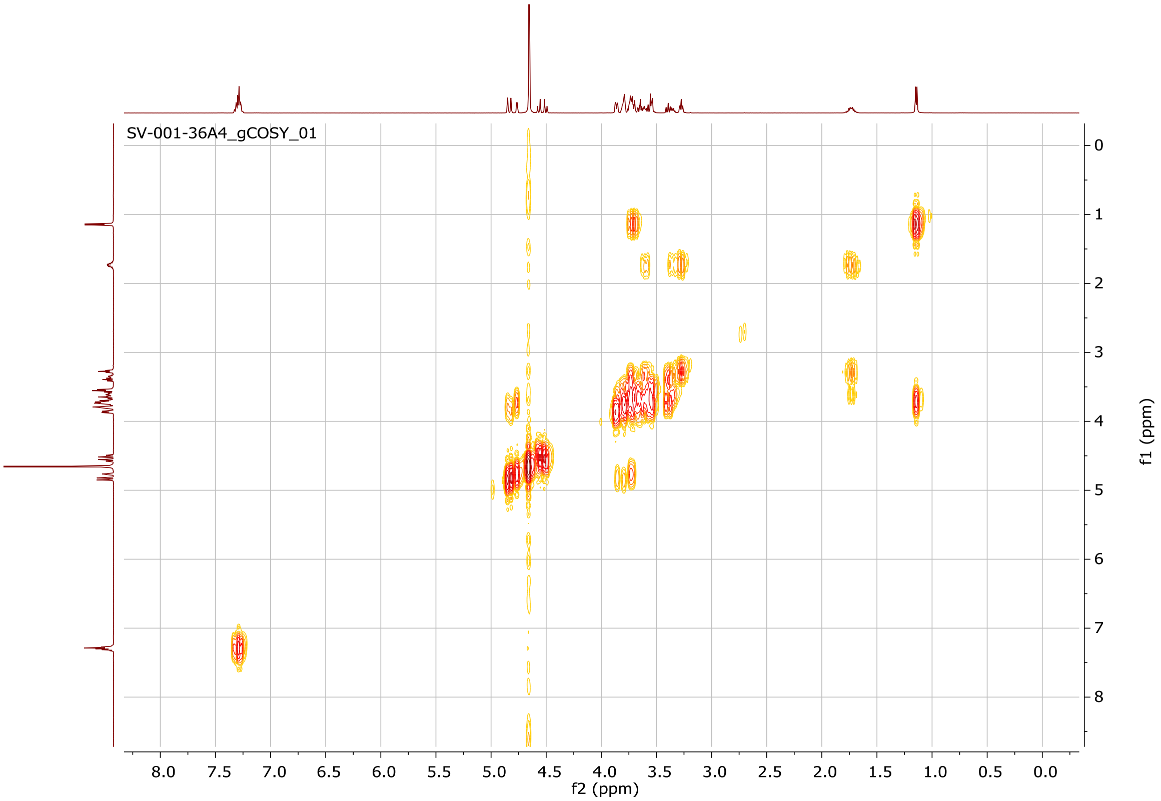

gCOSY NMR spectrum of compound **17**

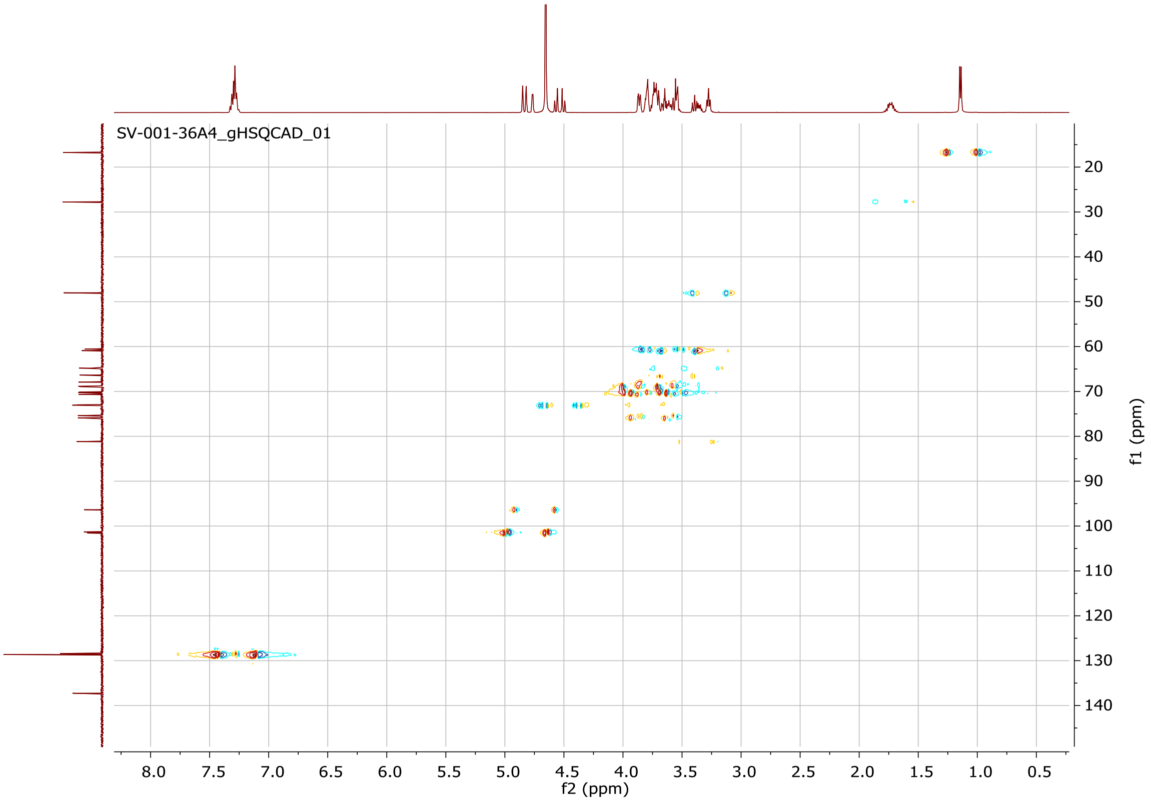

^{1}H-Coupled gHSQC NMR spectrum of compound **17**

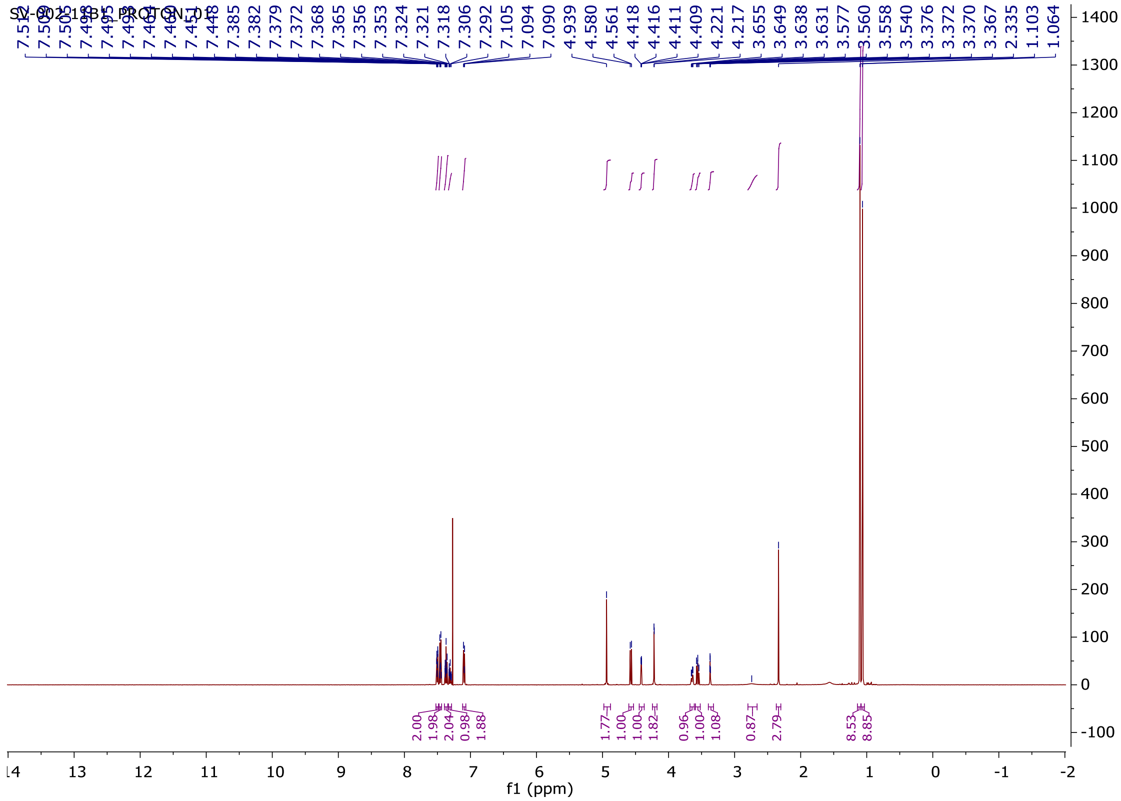

^{1}H NMR spectrum of compound **18**

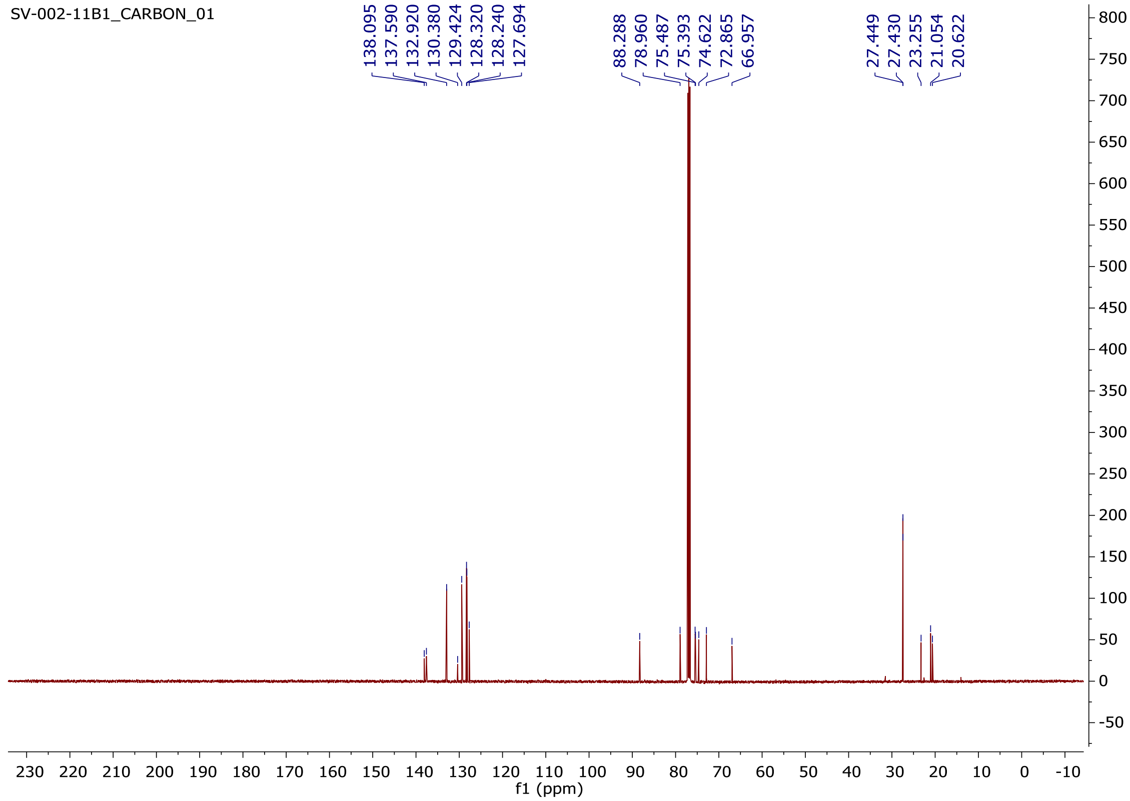

^{13}C NMR spectrum of compound **18**

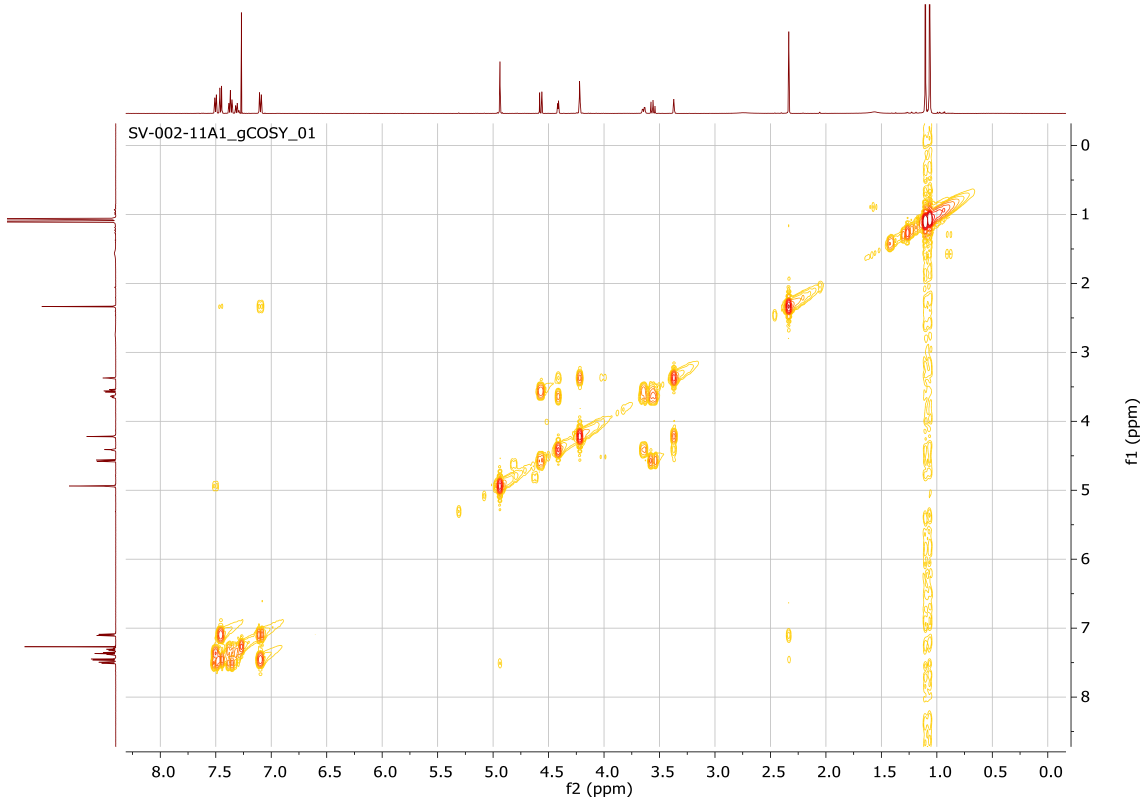

gCOSY NMR spectrum of compound **18**

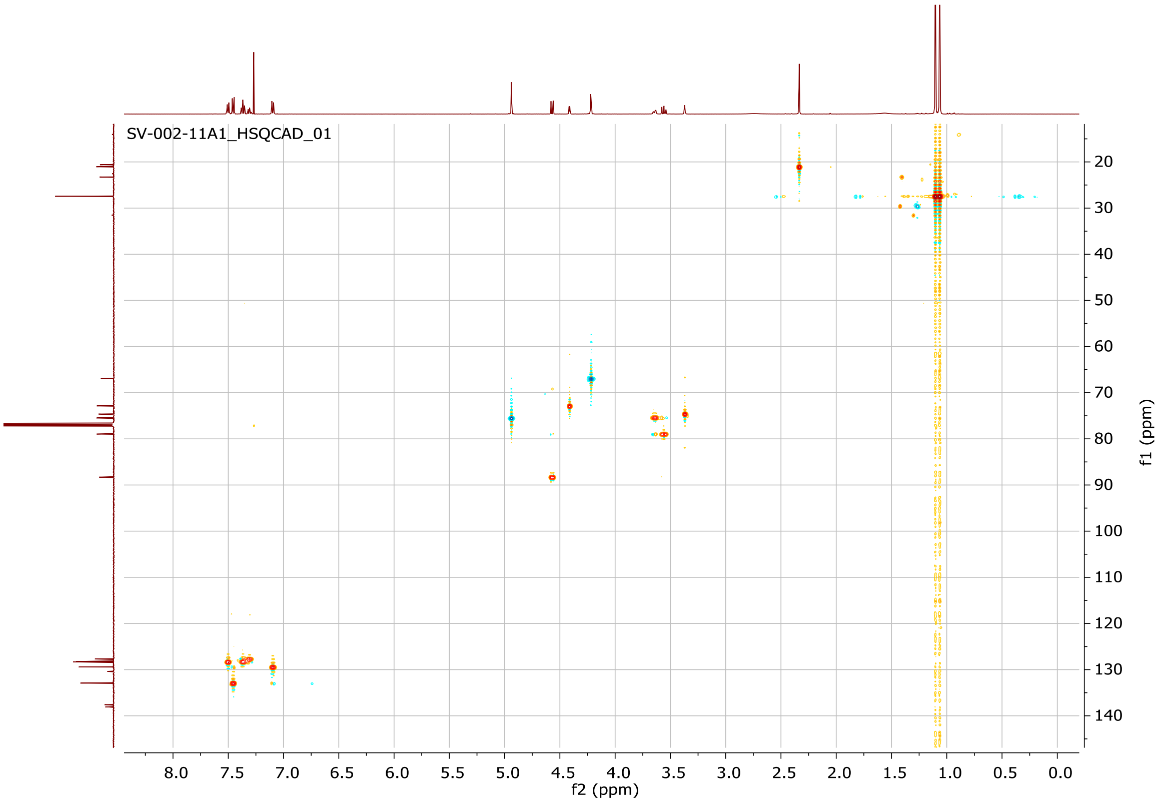

gHSQC NMR spectrum of compound **18**

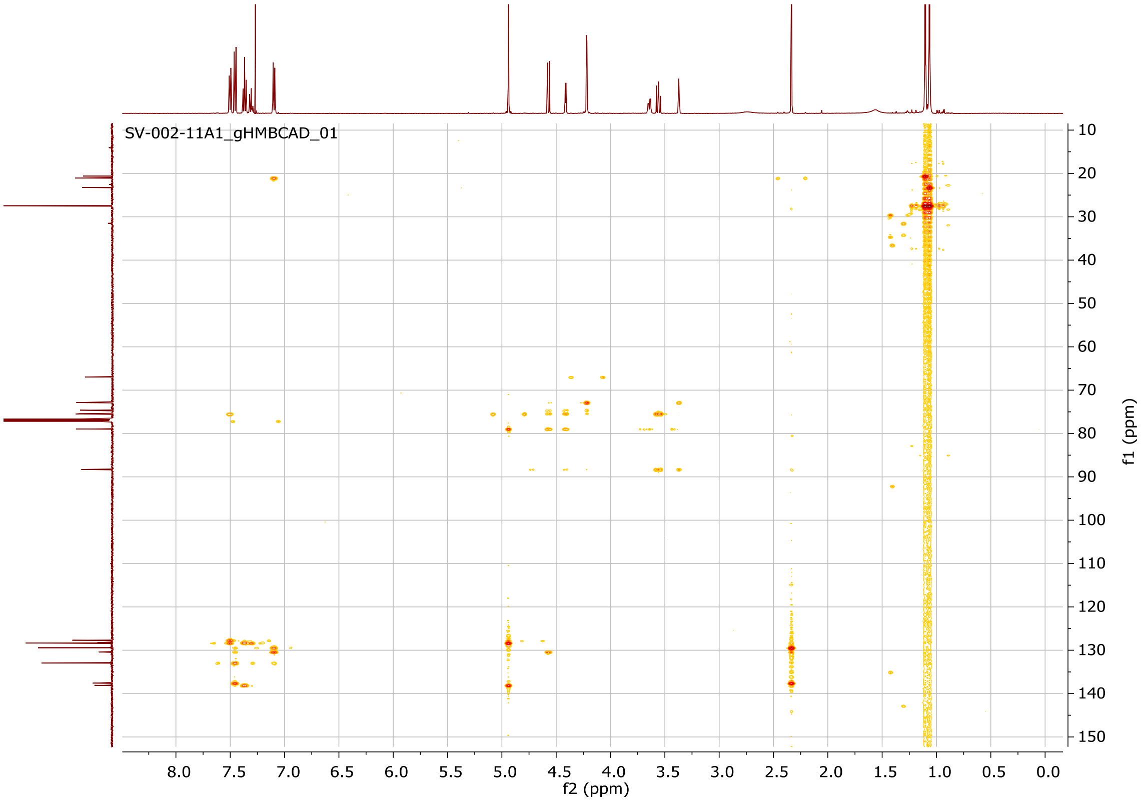

gHMBC NMR spectrum of compound **18**

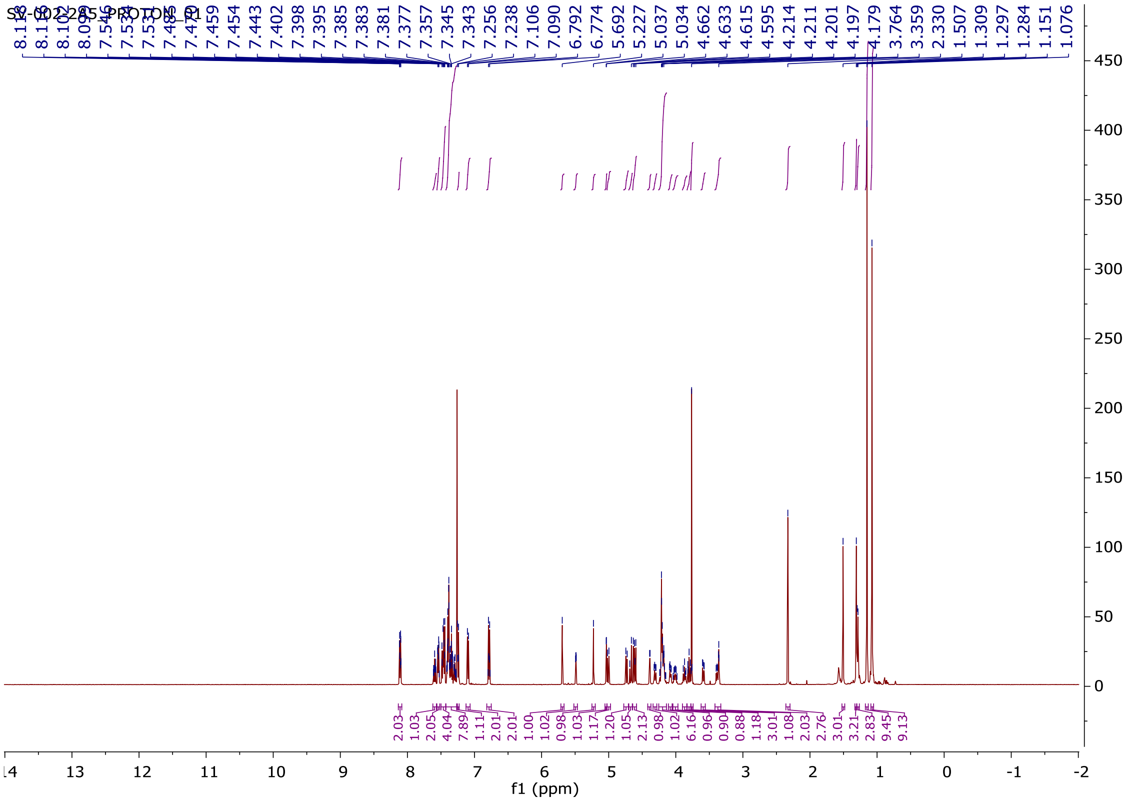

^{1}H NMR spectrum of compound **19**

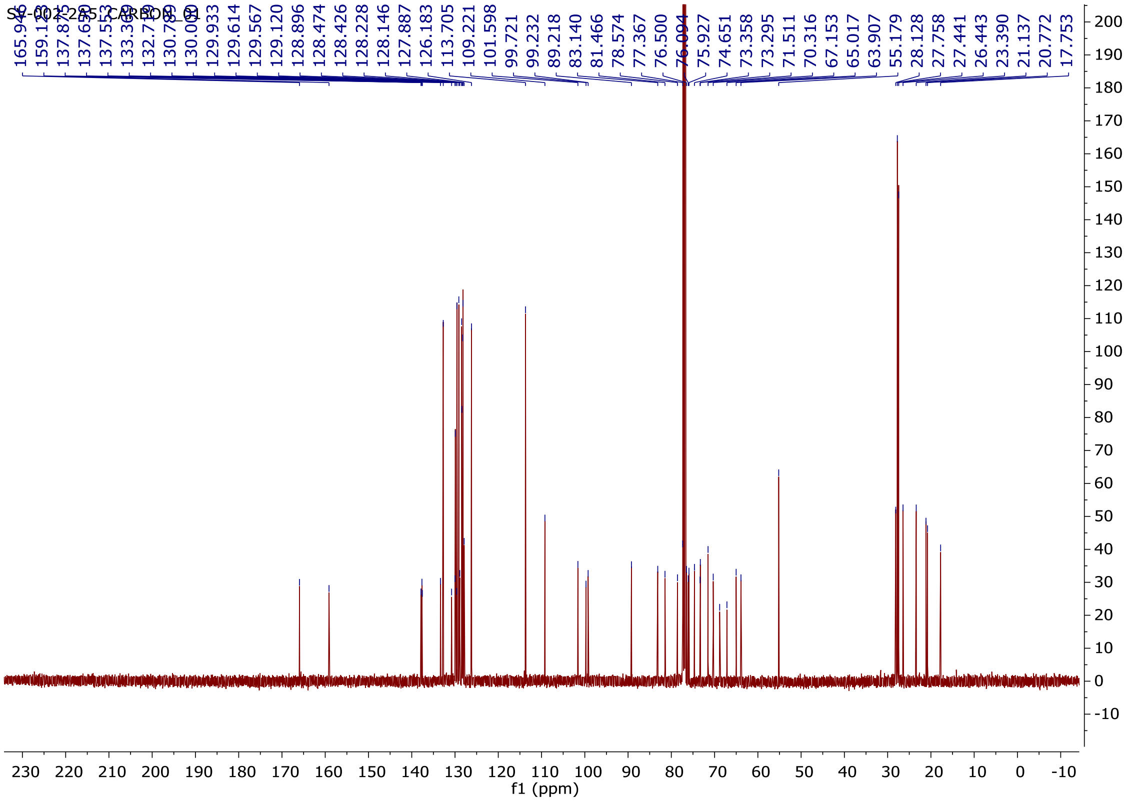

^{13}C NMR spectrum of compound **19**

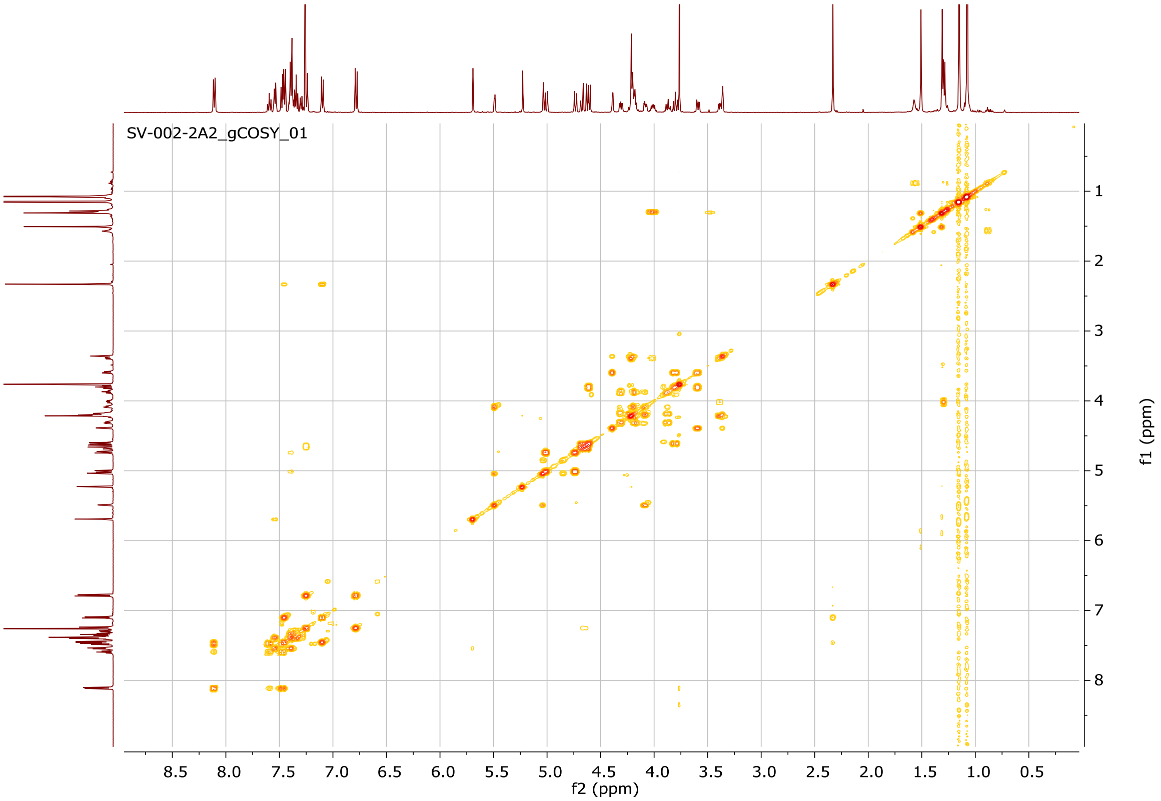

gCOSY NMR spectrum of compound **19**

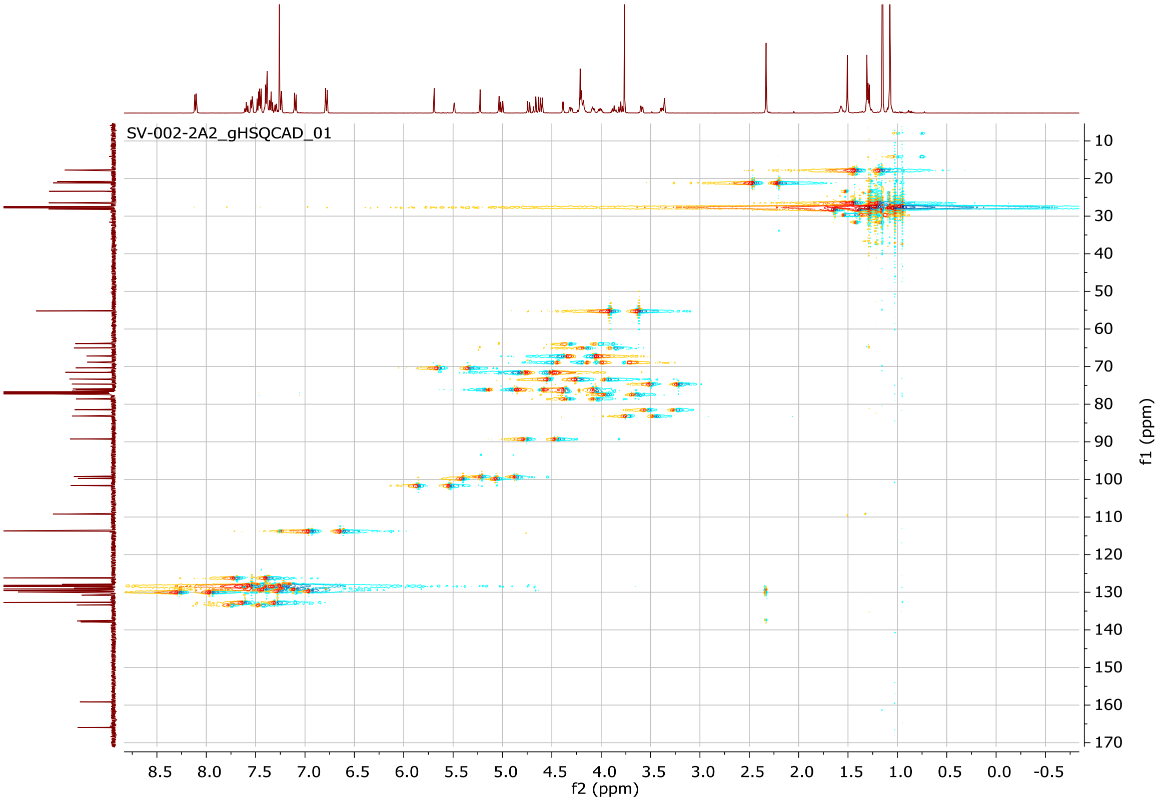

^{1}H-Coupled gHSQC NMR spectrum of compound **19**

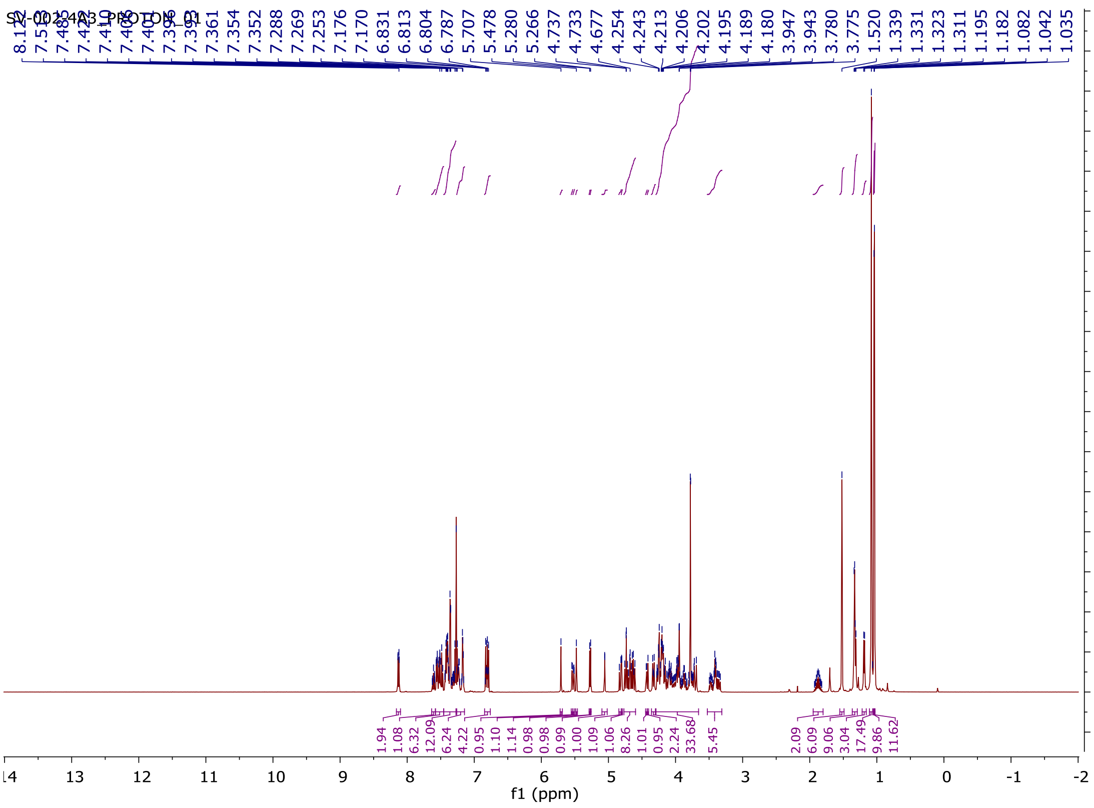

^{1}H NMR spectrum of compound **20**

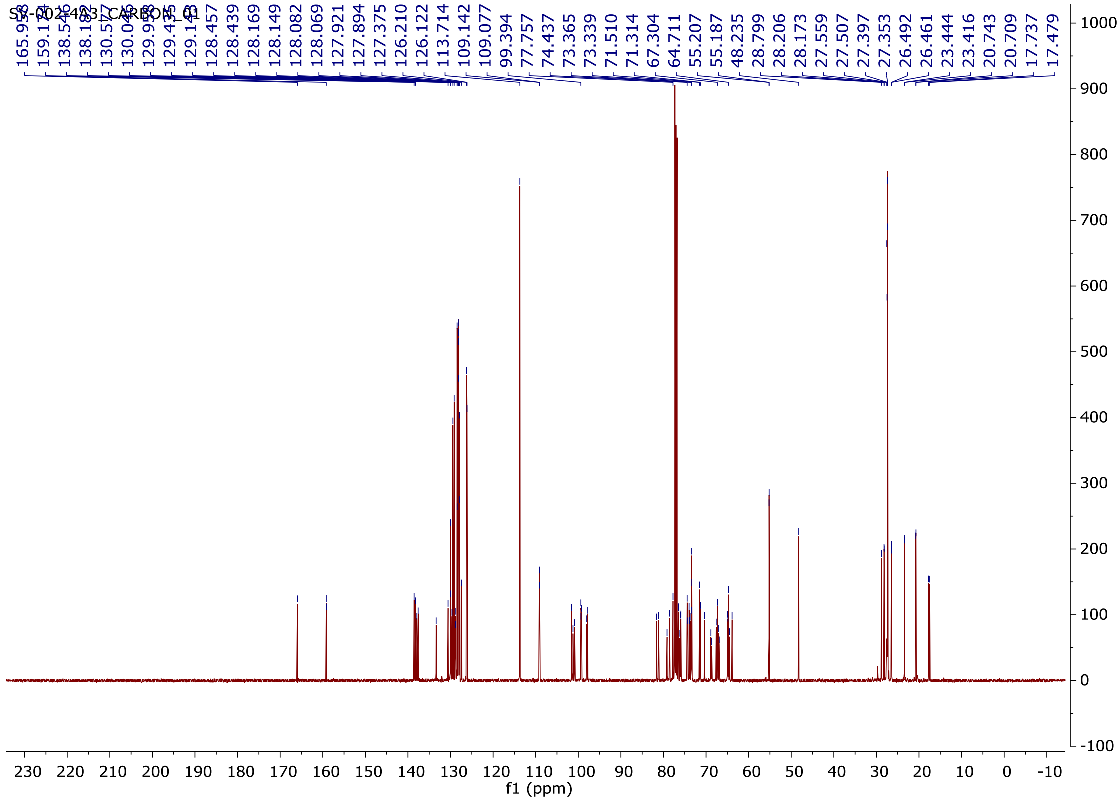

^{13}C NMR spectrum of compound **20**

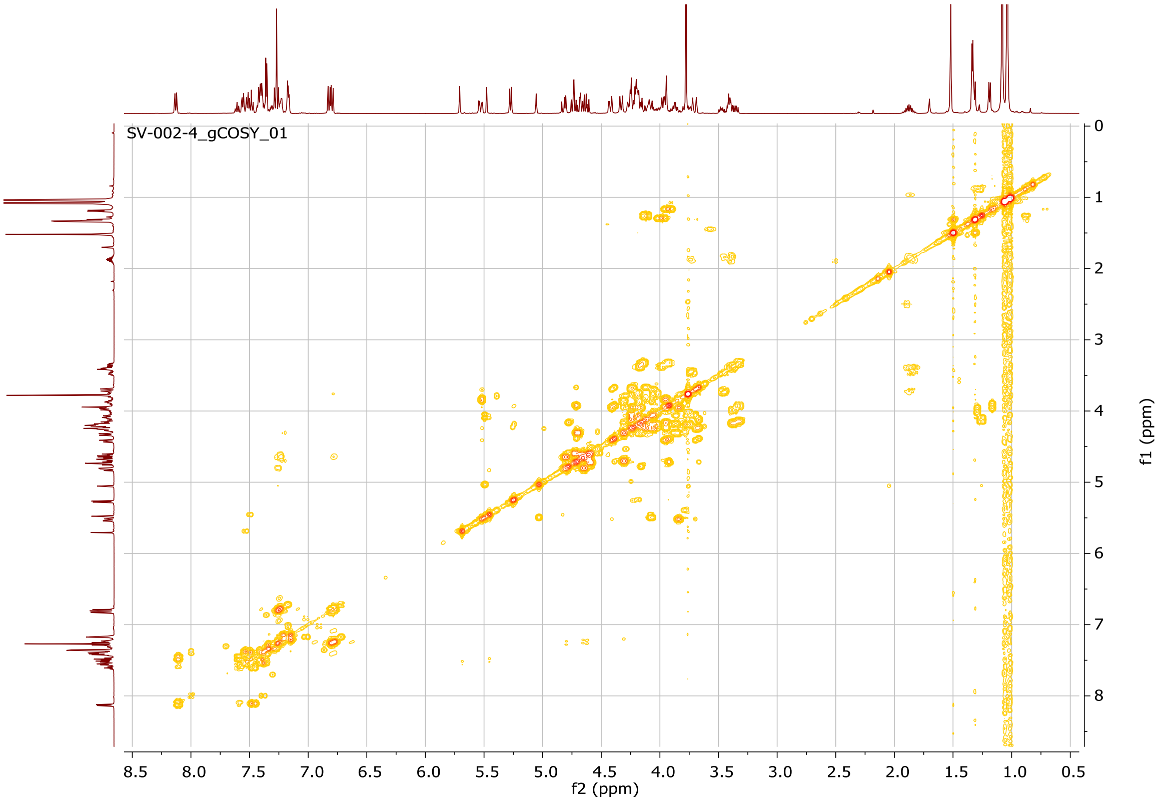

gCOSY NMR spectrum of compound **20**

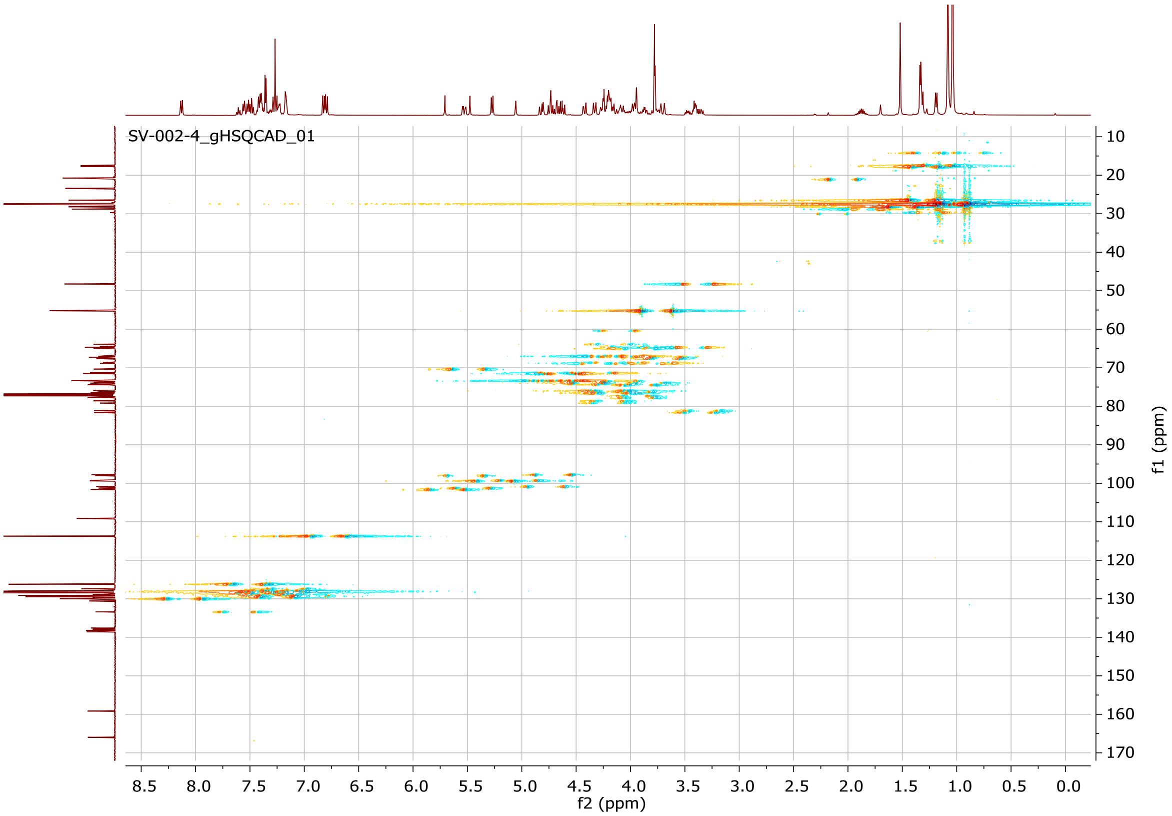

^{1}H-Coupled gHSQC NMR spectrum of compound **20**

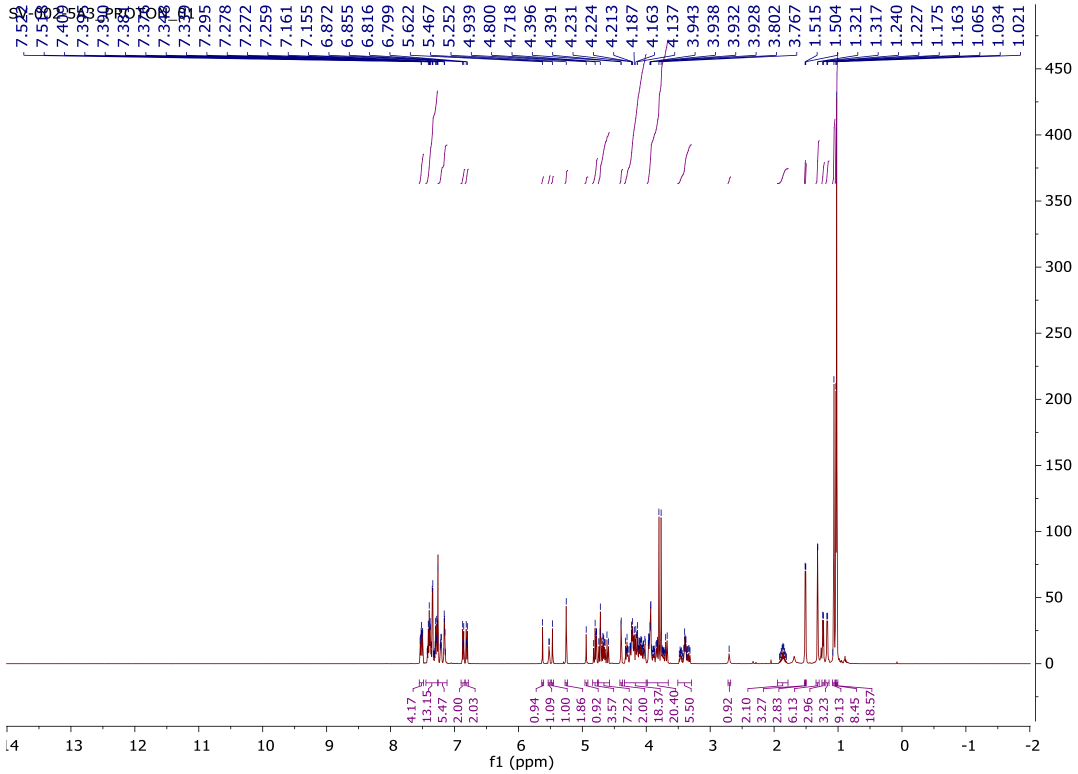

^{1}H NMR spectrum of compound **21**

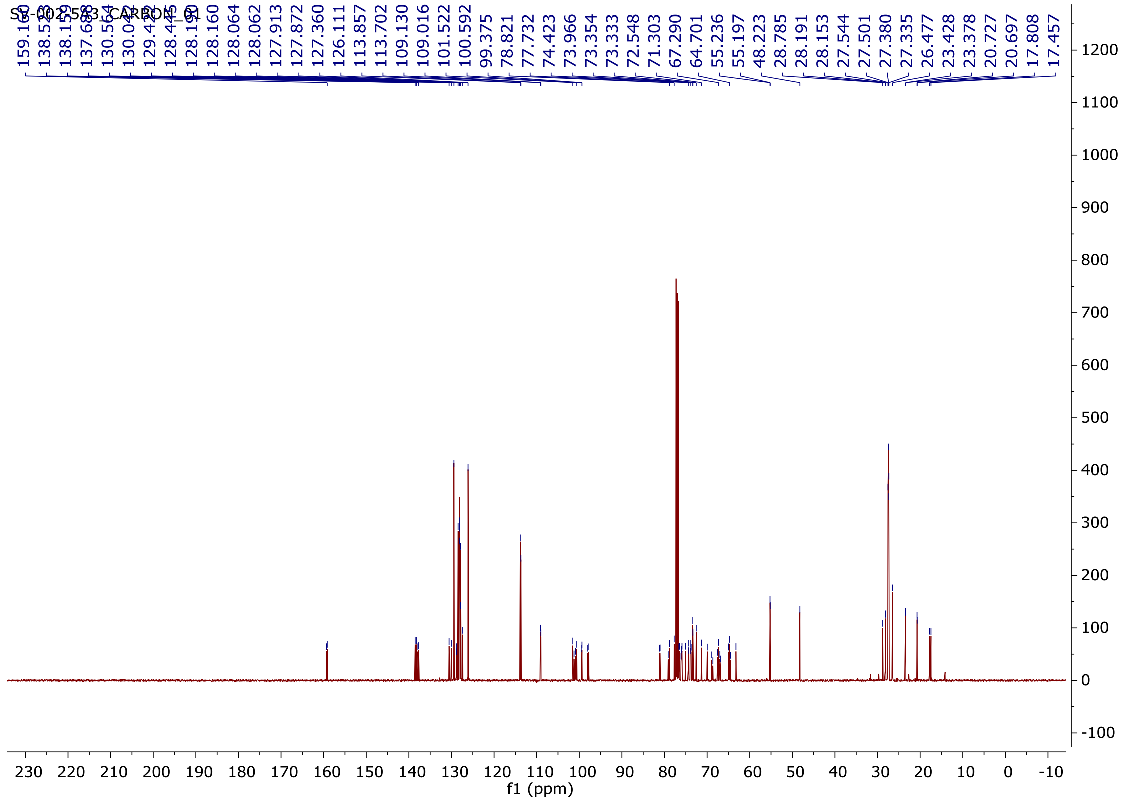

^{13}C NMR spectrum of compound **21**

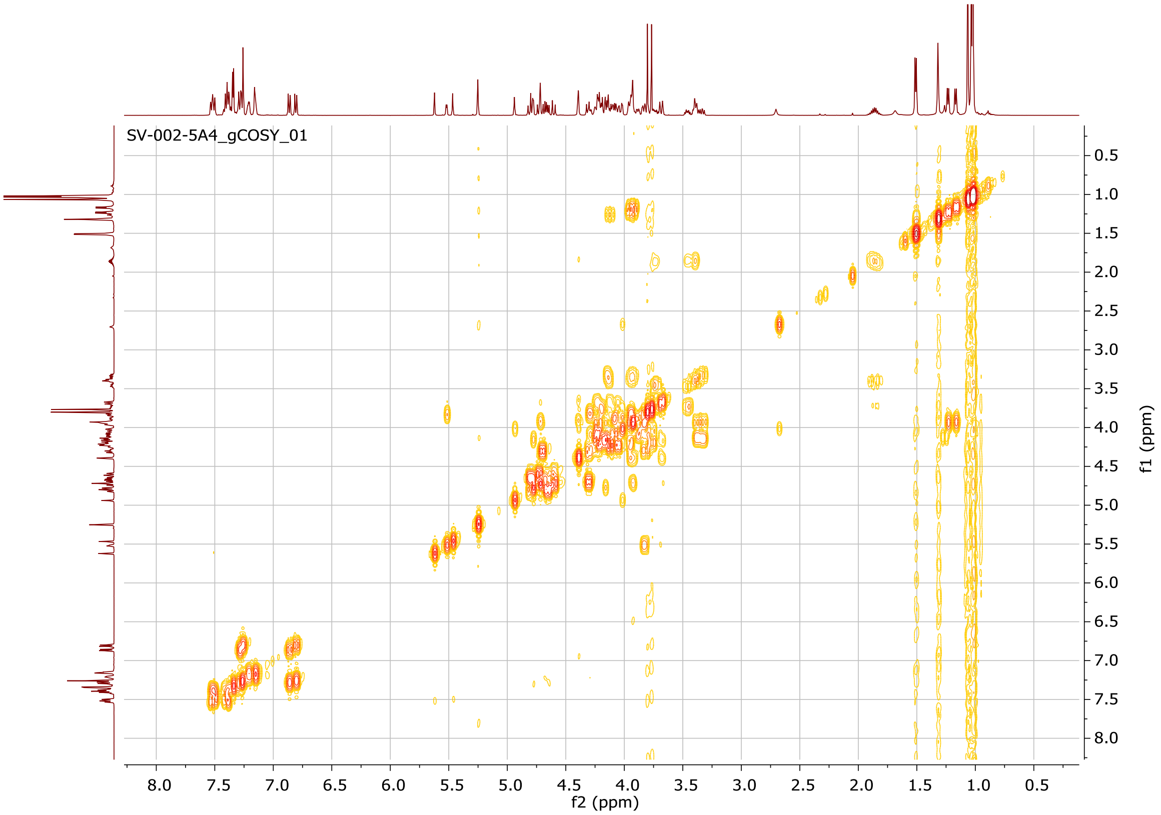

gCOSY NMR spectrum of compound **21**

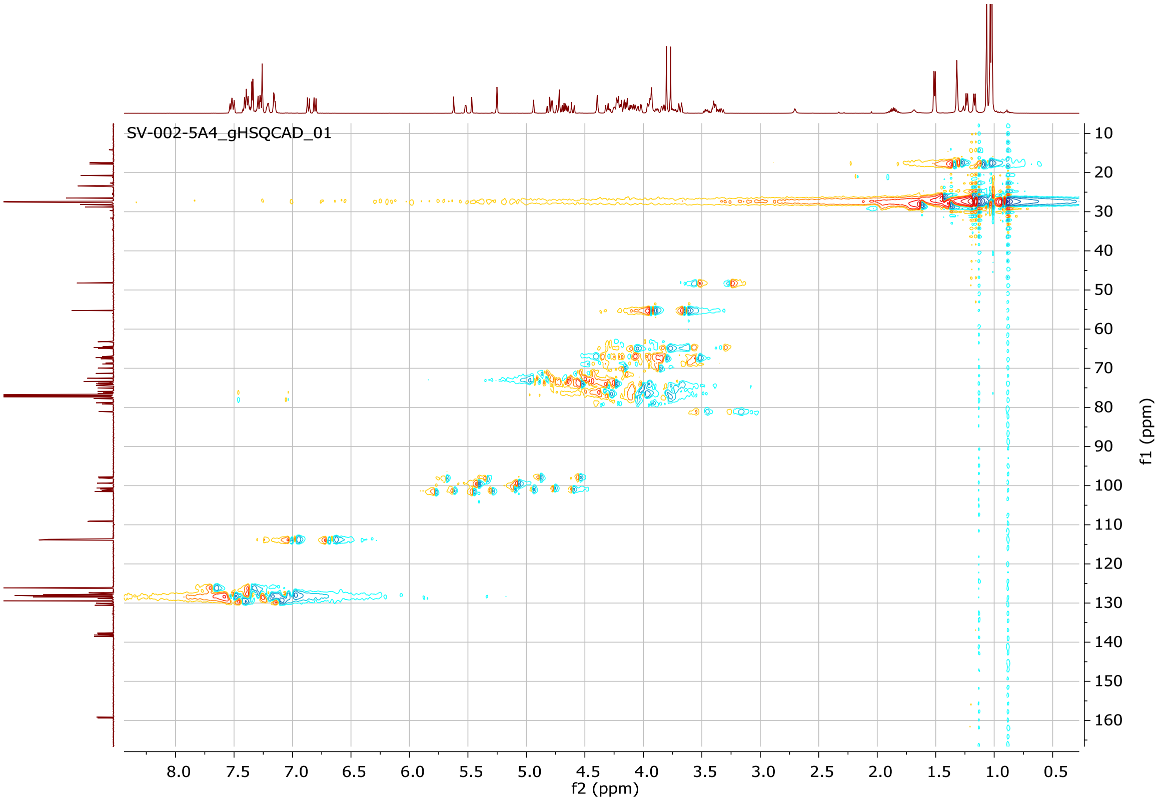

^{1}H-Coupled gHSQC NMR spectrum of compound **21**

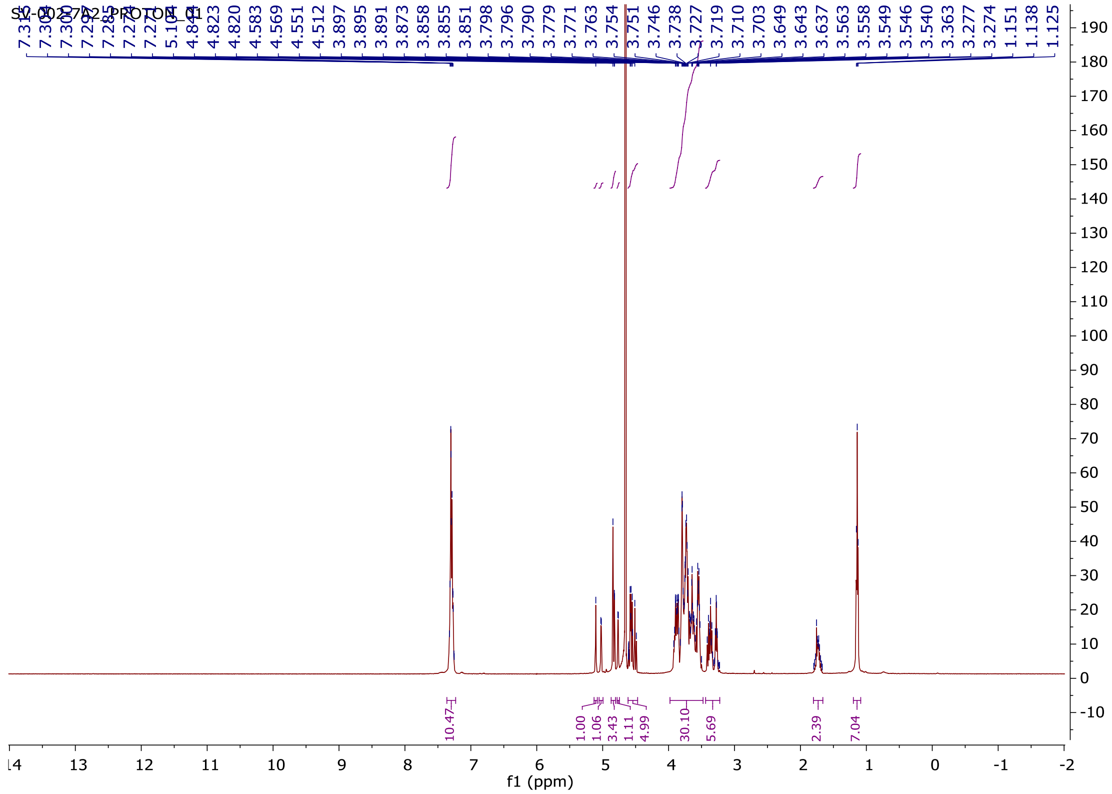

^{1}H NMR spectrum of compound **22**

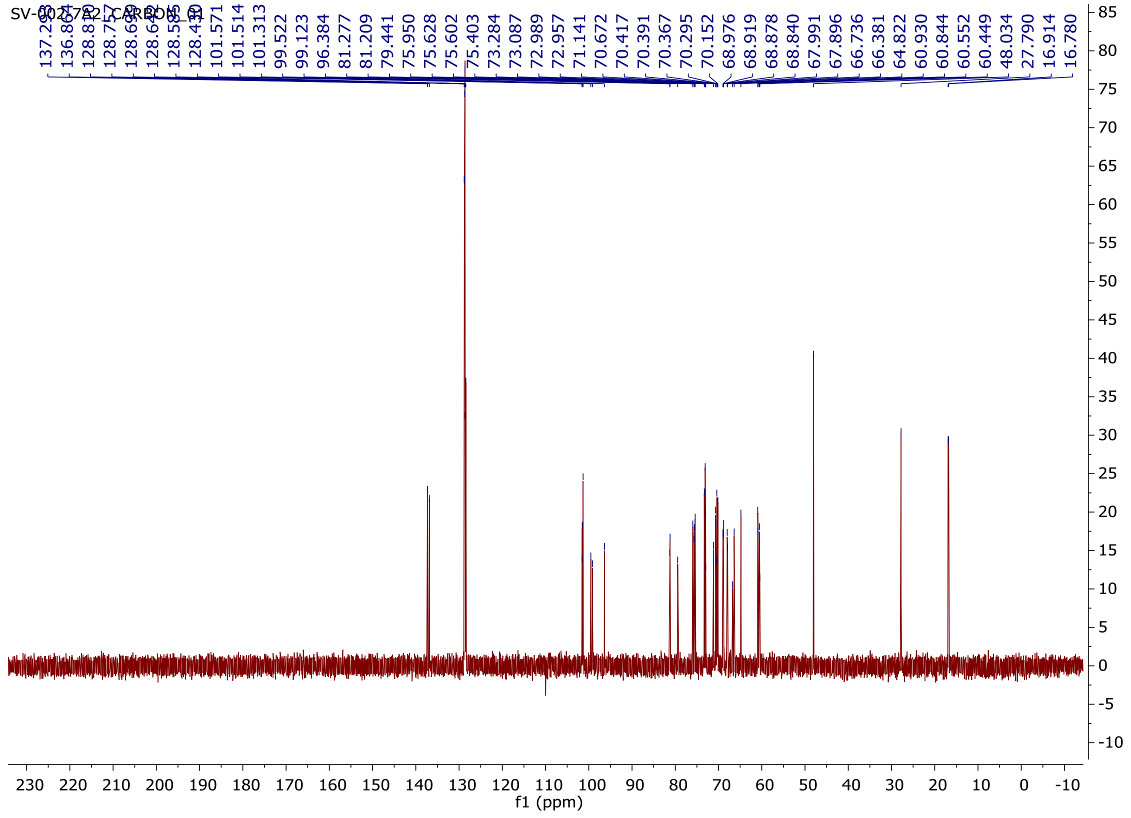

^{13}C NMR spectrum of compound **22**

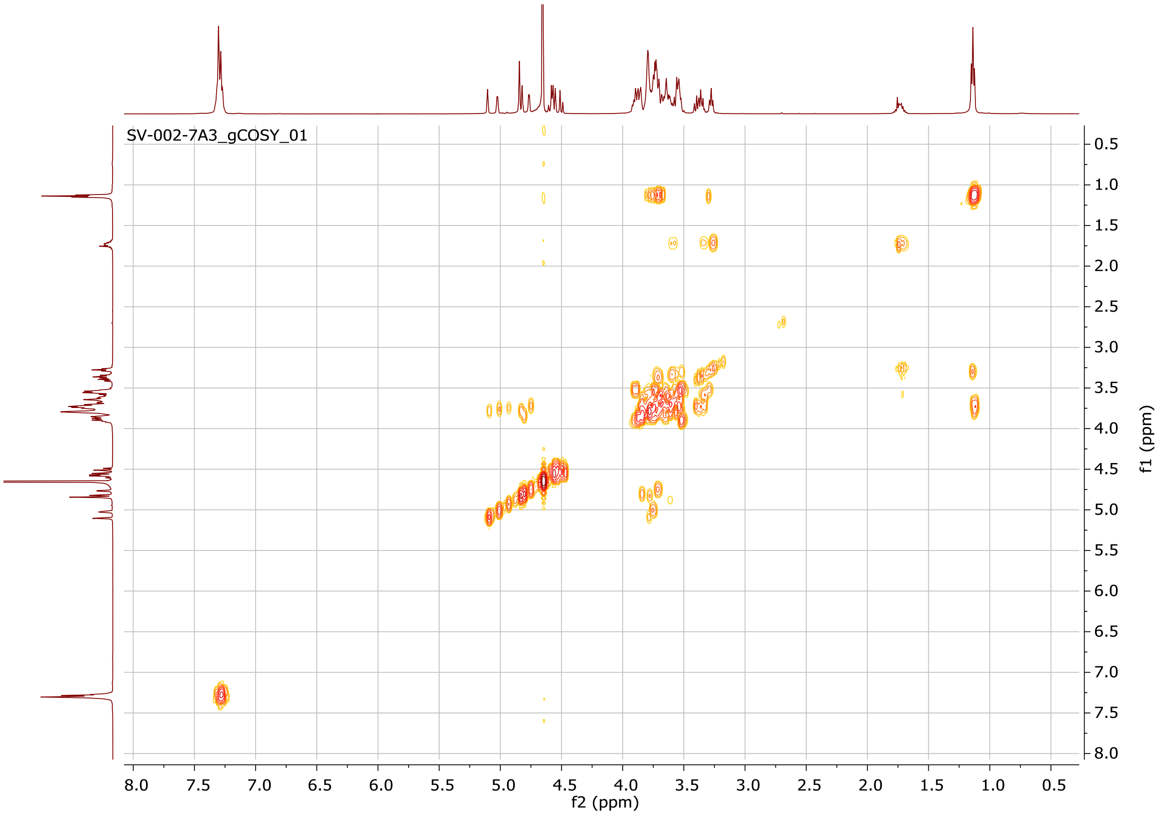

gCOSY NMR spectrum of compound **22**

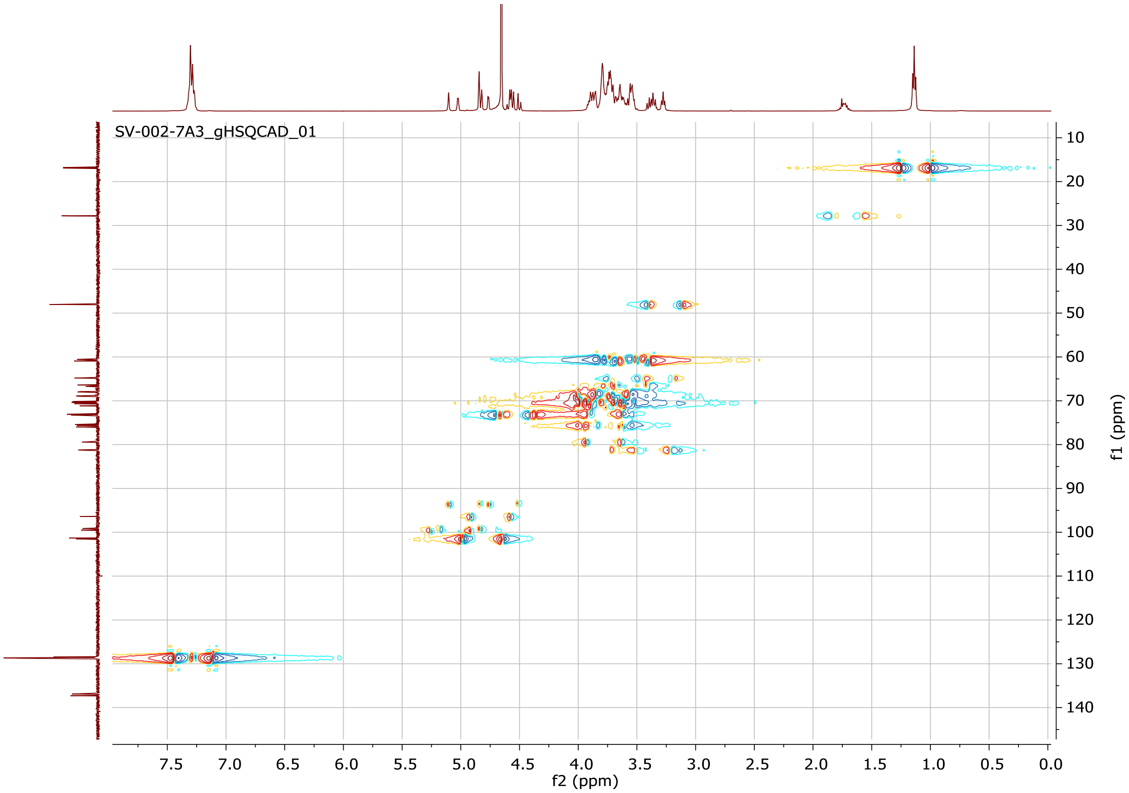

^{1}H-Coupled gHSQC NMR spectrum of compound **22**

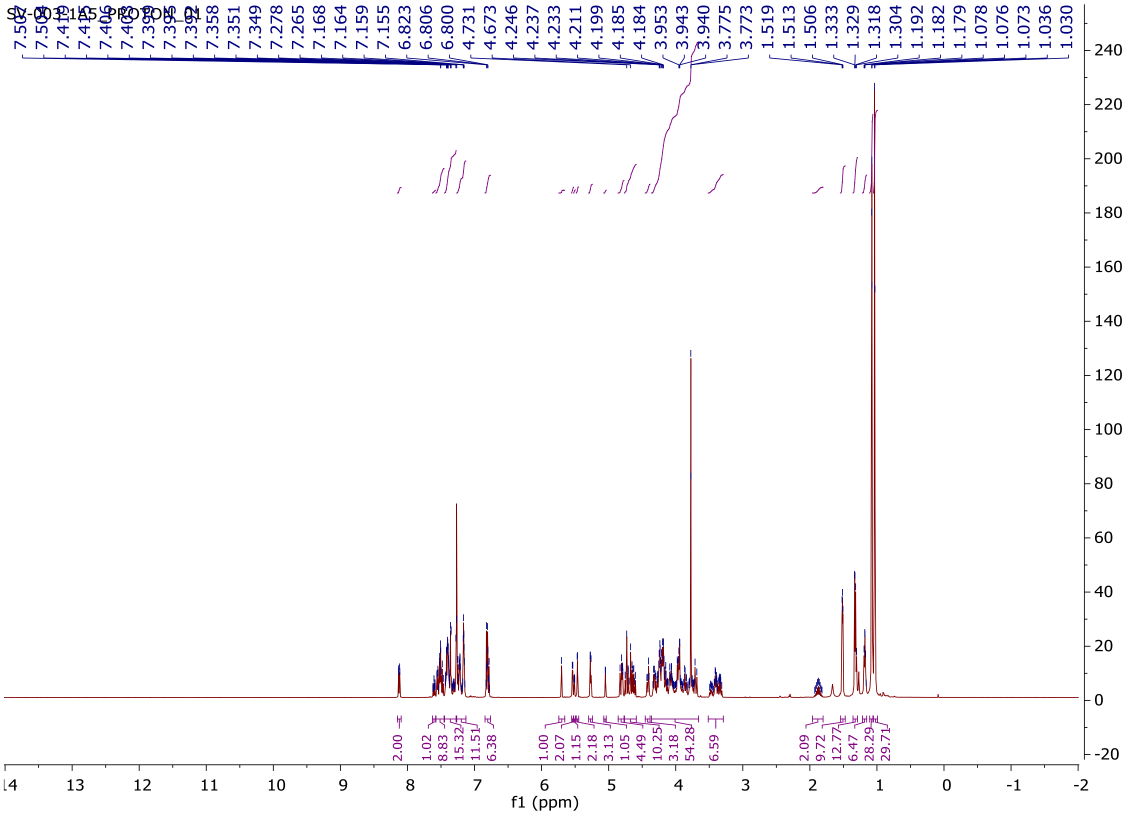

^{1}H NMR spectrum of compound **23**

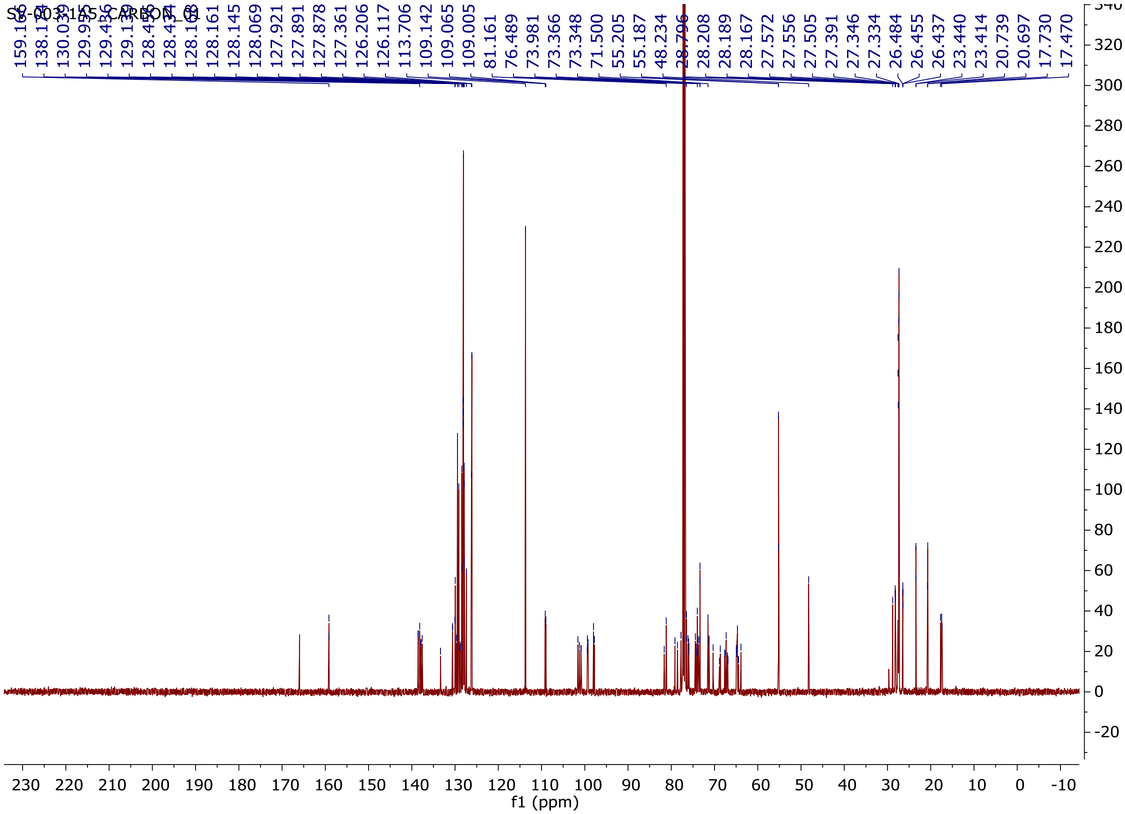

^{13}C NMR spectrum of compound **23**

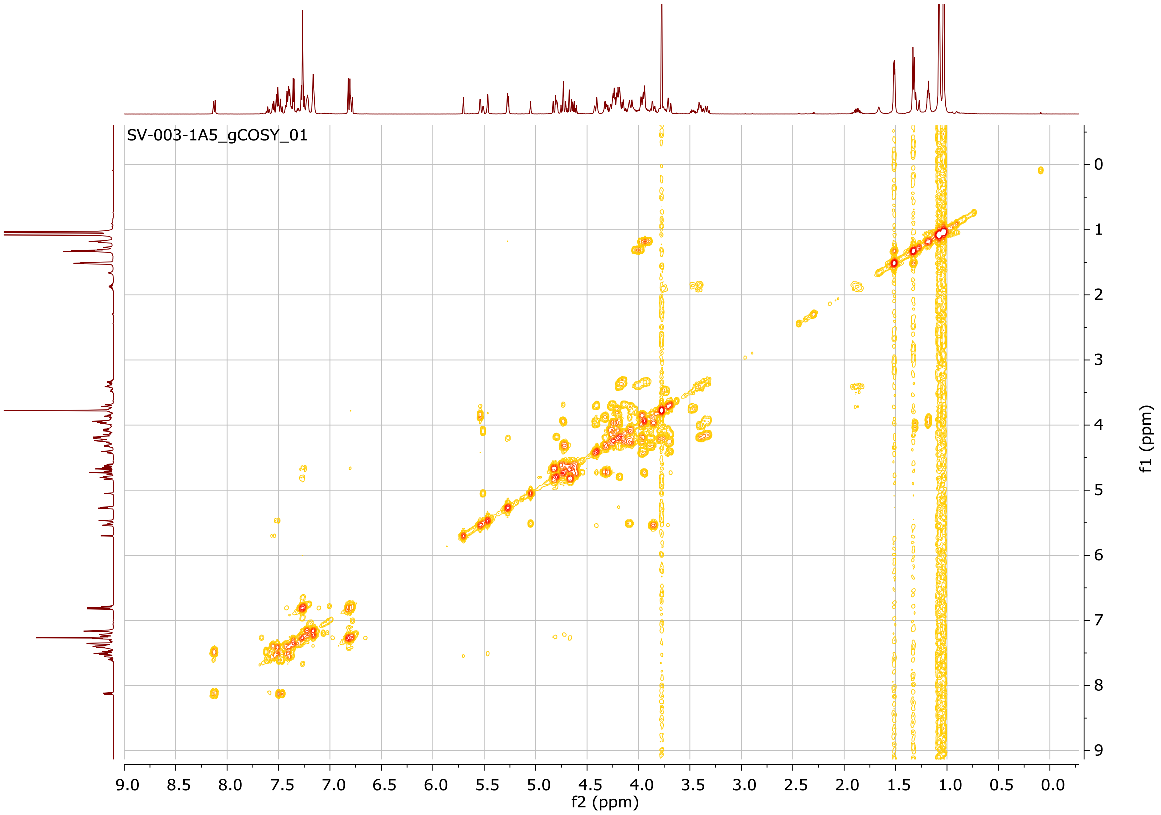

gCOSY NMR spectrum of compound **23**

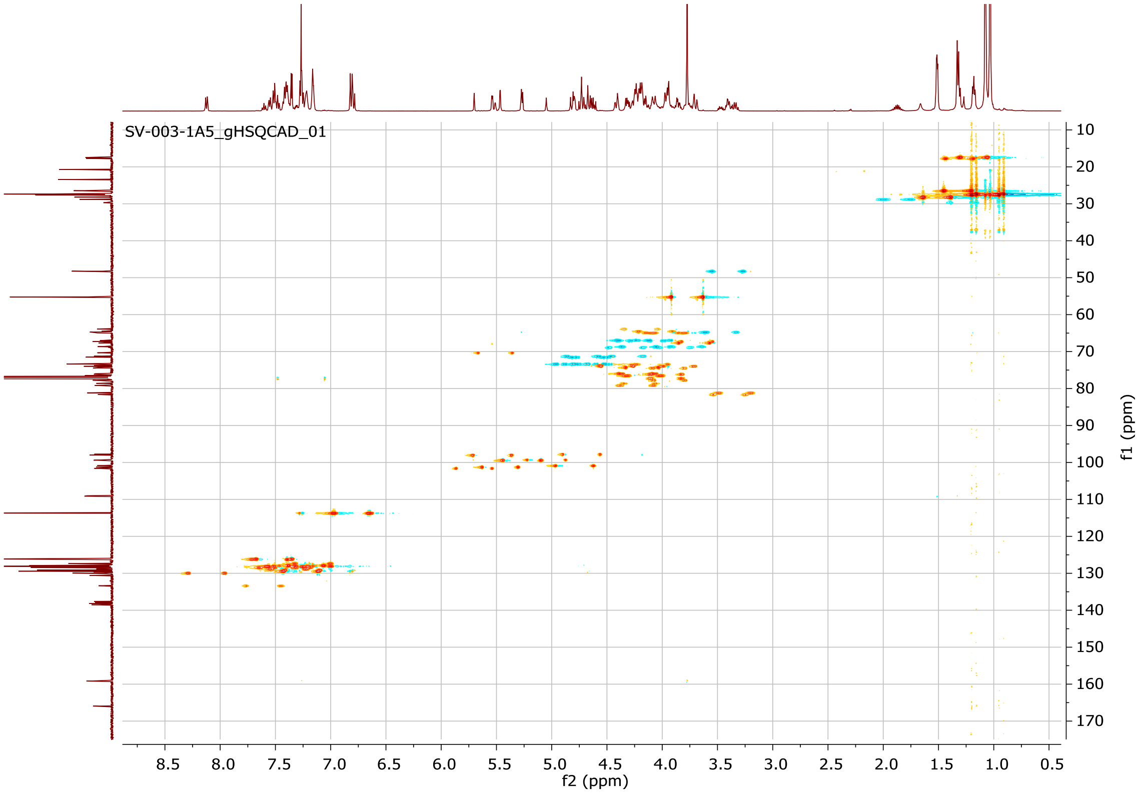

^{1}H-Coupled gHSQC NMR spectrum of compound **23**

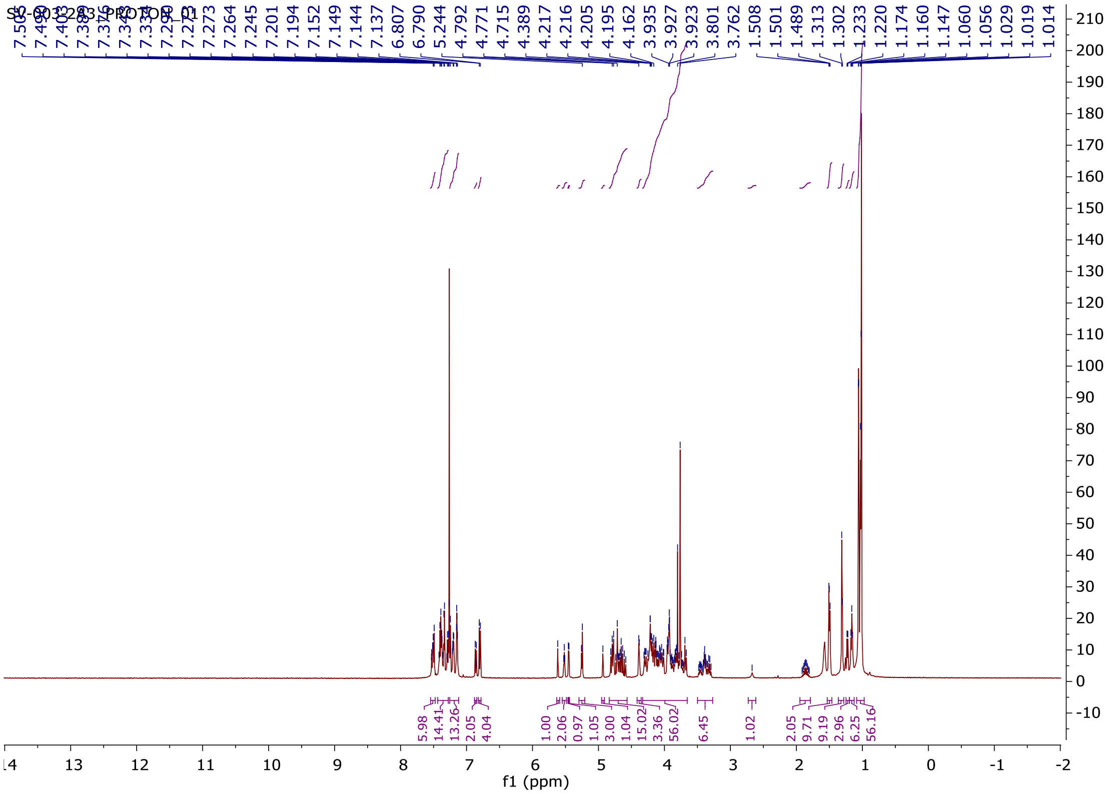

^{1}H NMR spectrum of compound **24**

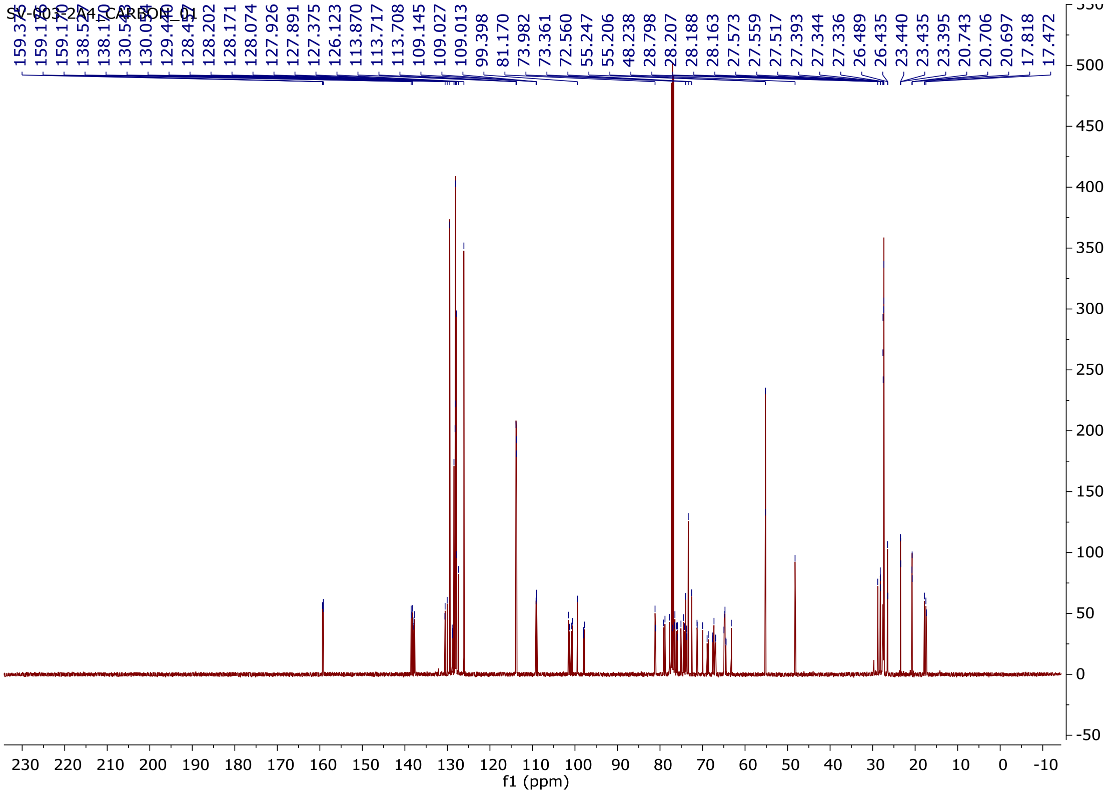

^{13}C NMR spectrum of compound **24**

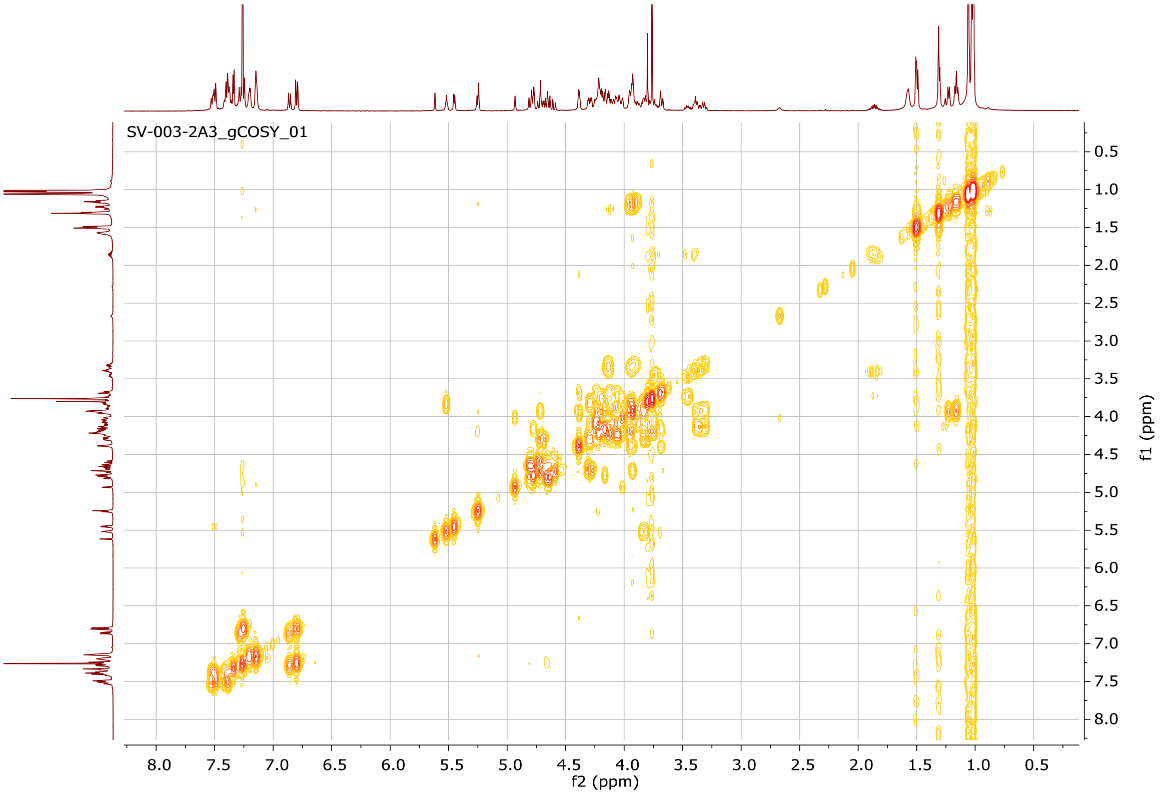

gCOSY NMR spectrum of compound **24**

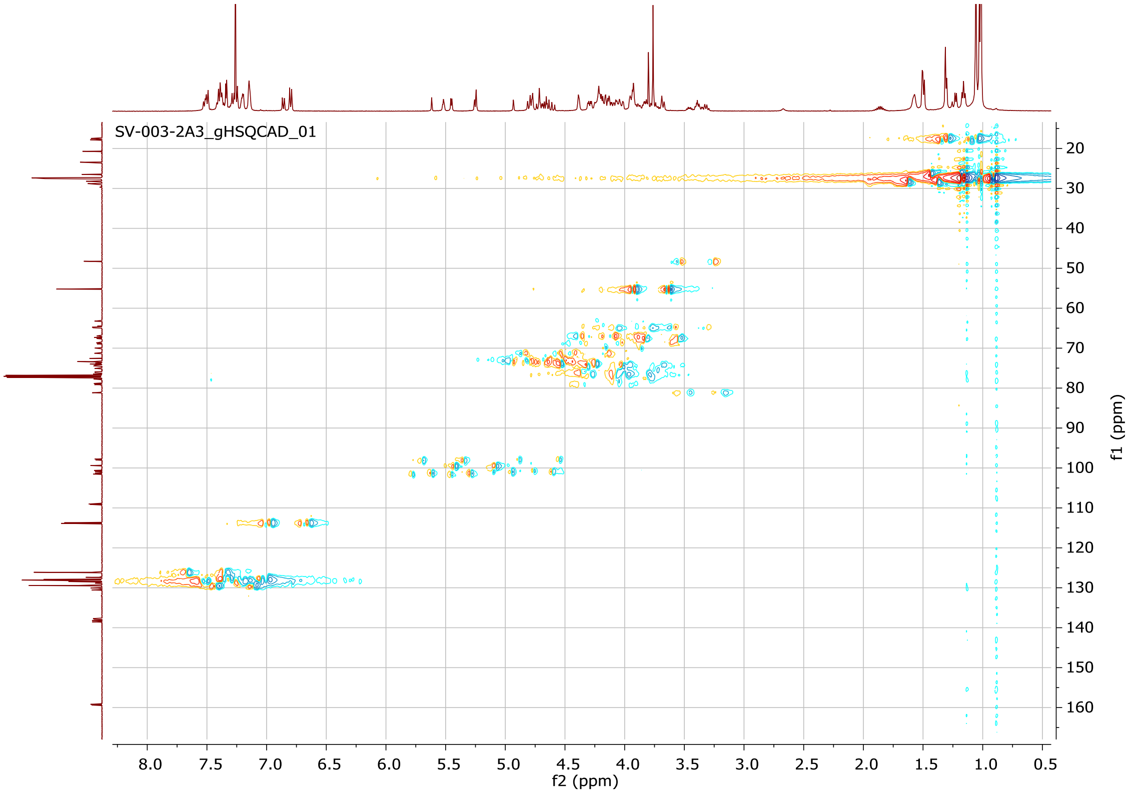

^{1}H-Coupled gHSQC NMR spectrum of compound **24**

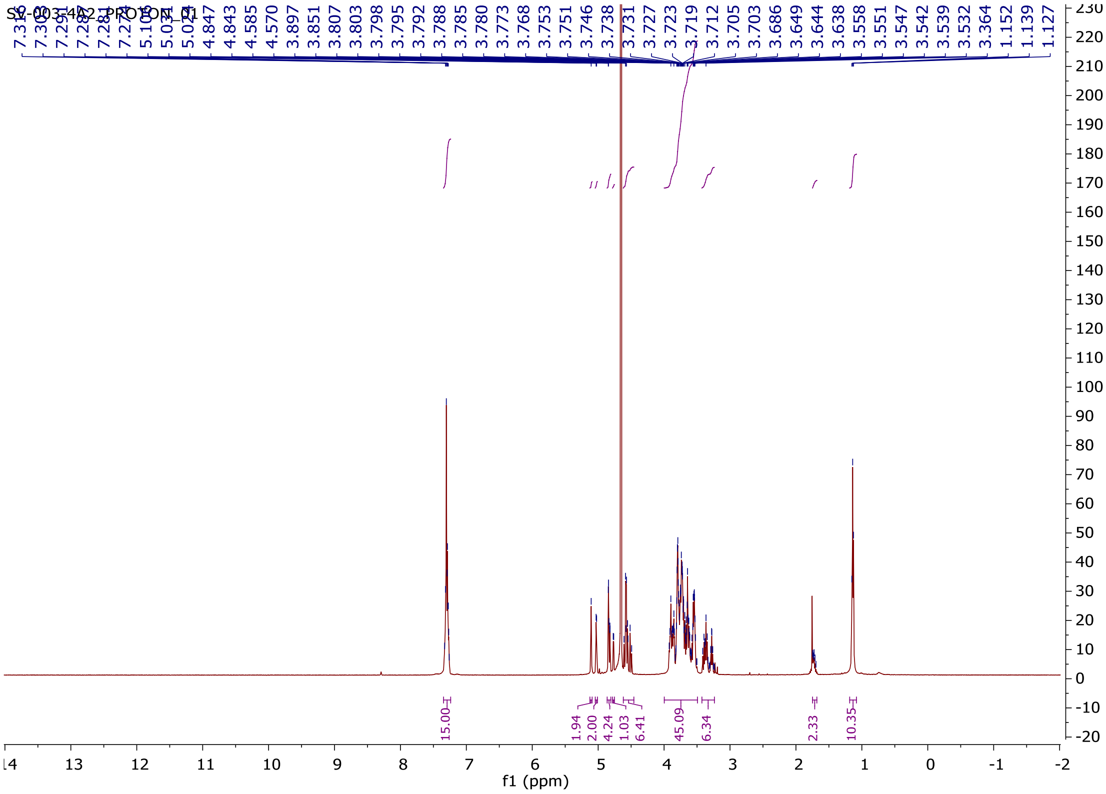

^{1}H NMR spectrum of compound **25**

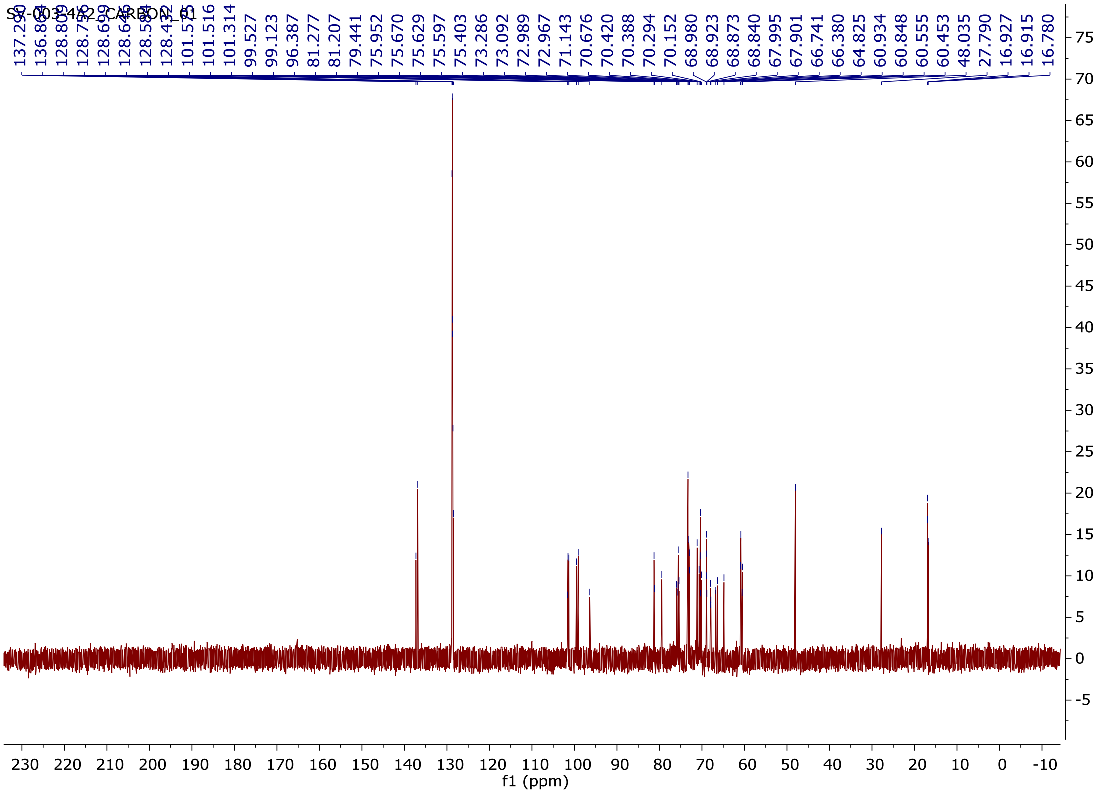

^{13}C NMR spectrum of compound **25**

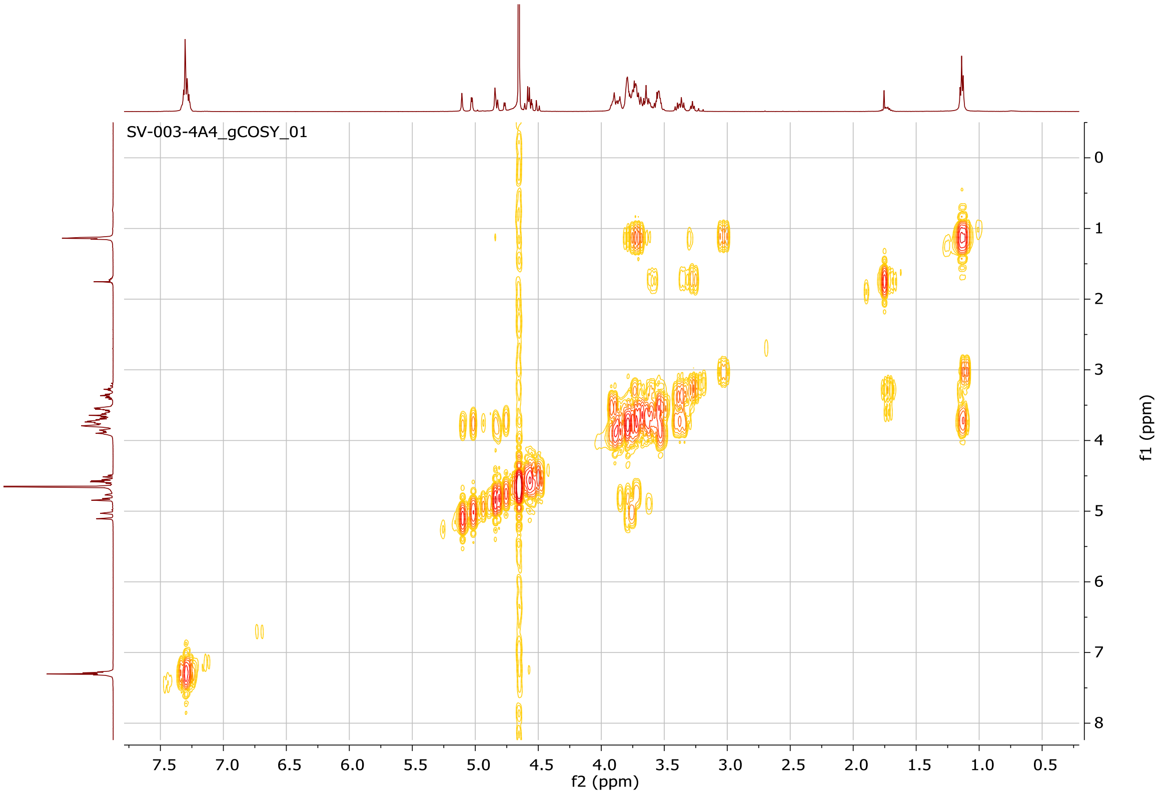

gCOSY NMR spectrum of compound **25**

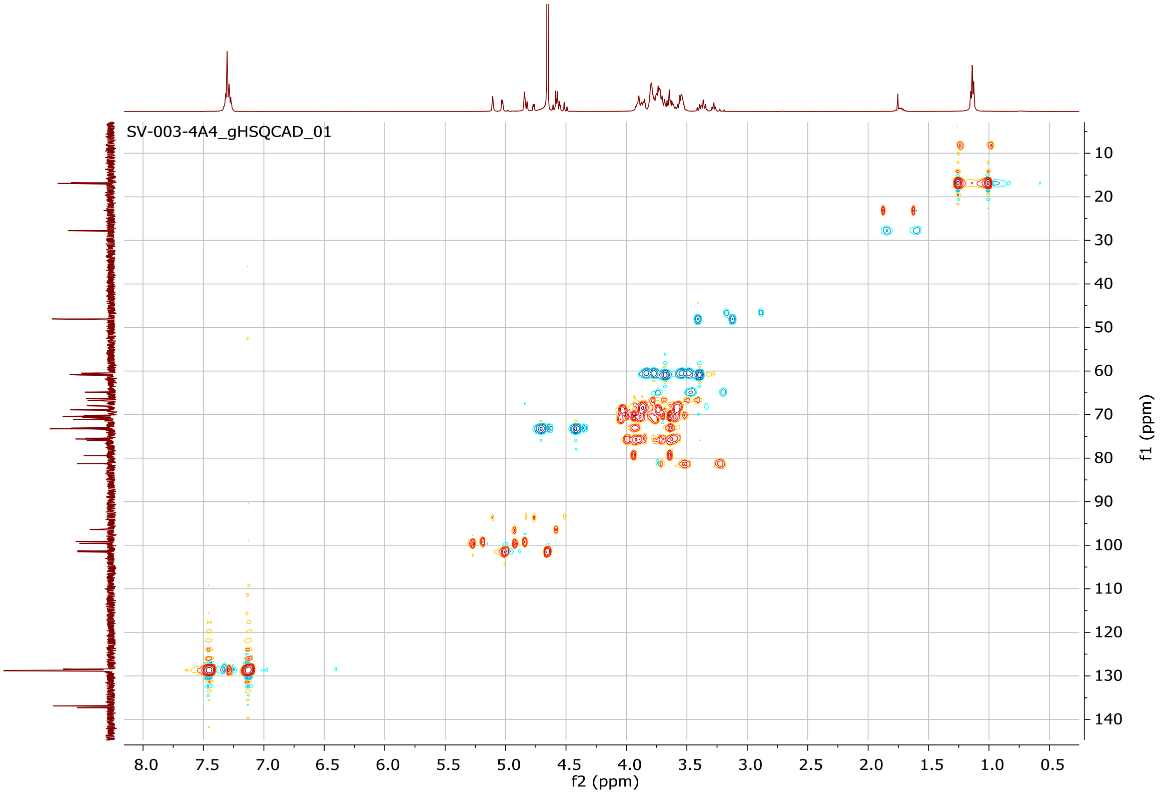

^{1}H-Coupled gHSQC NMR spectrum of compound **25**

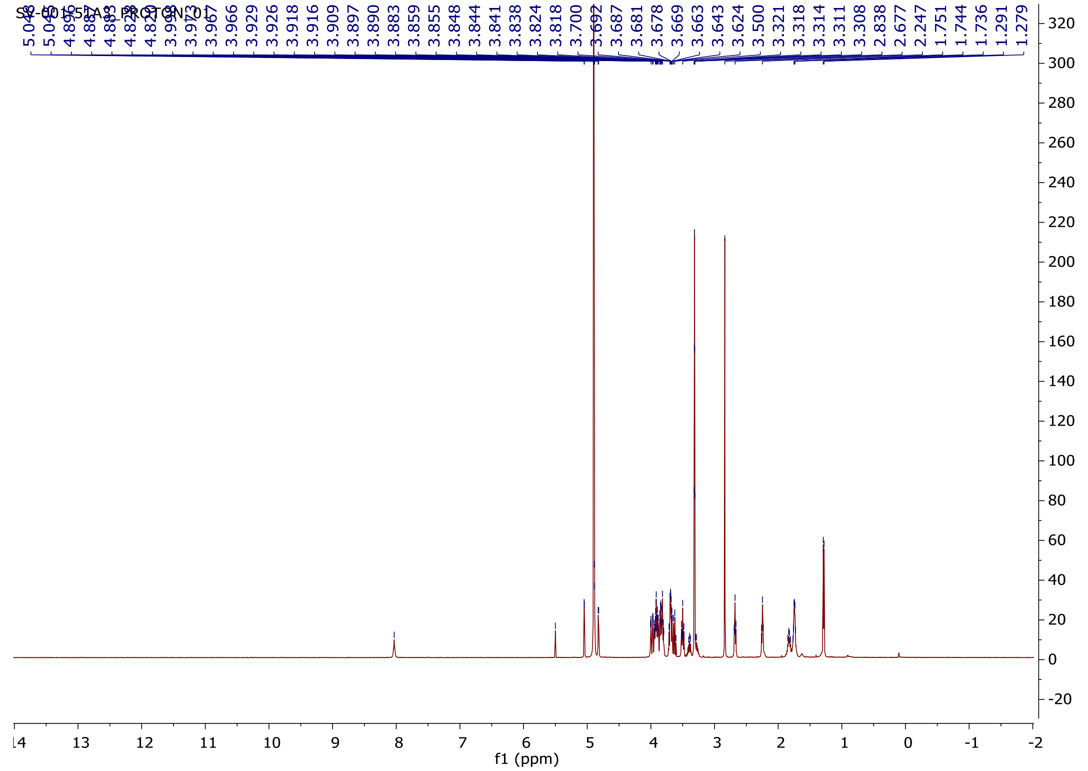

^{1}H NMR spectrum of compound **1'**

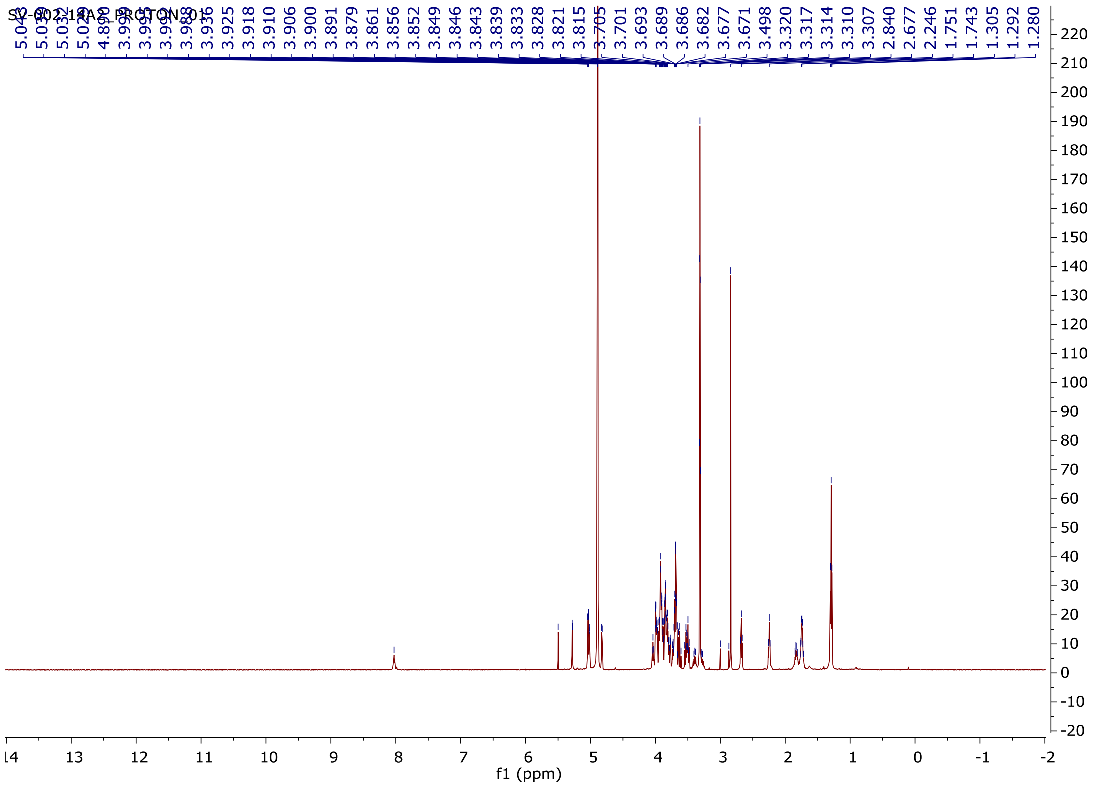

^{1}H NMR spectrum of compound **2'**

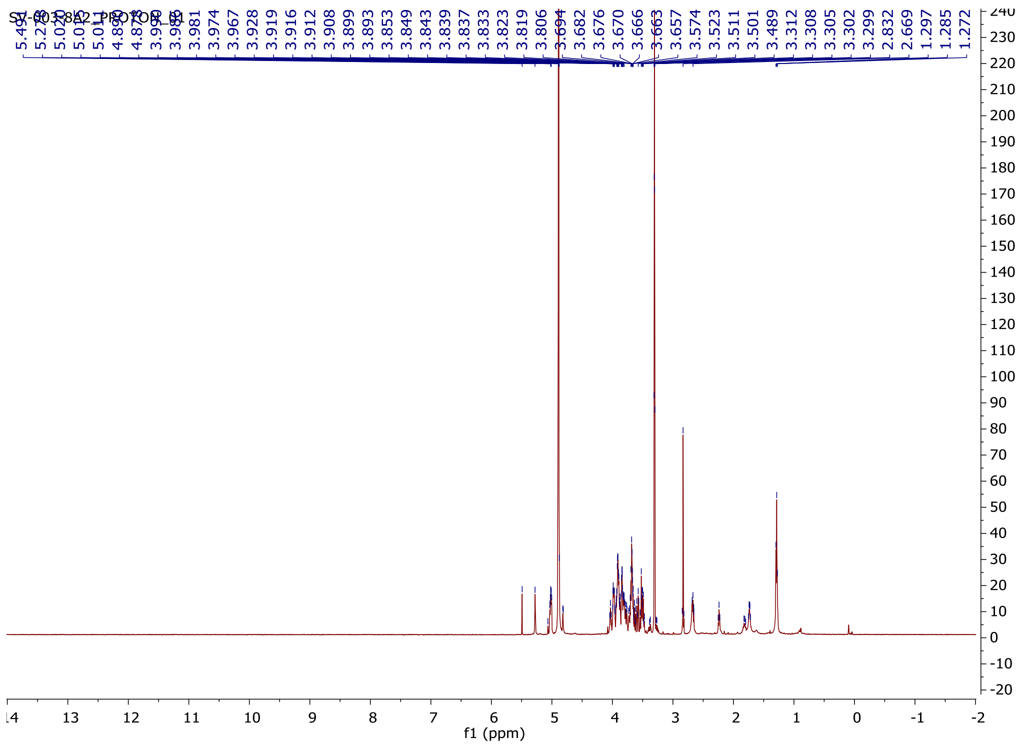

^{1}H NMR spectrum of compound **3'**

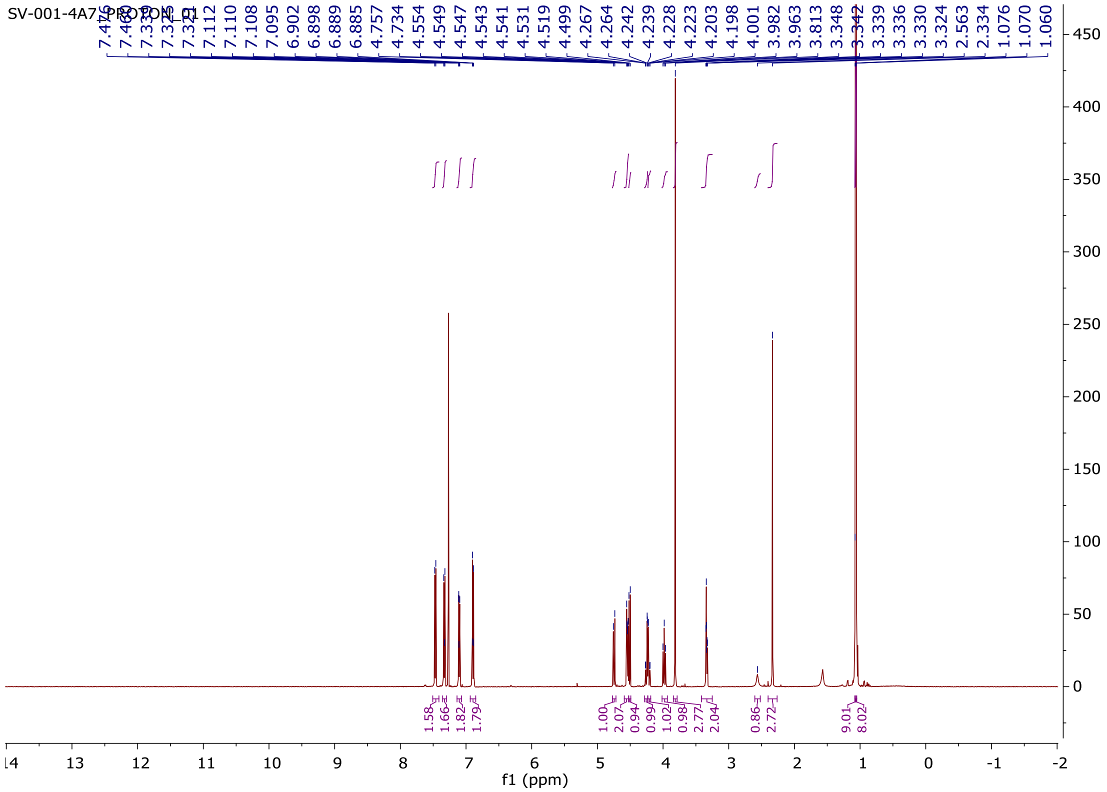

^{1}H NMR spectrum of compound **S1**

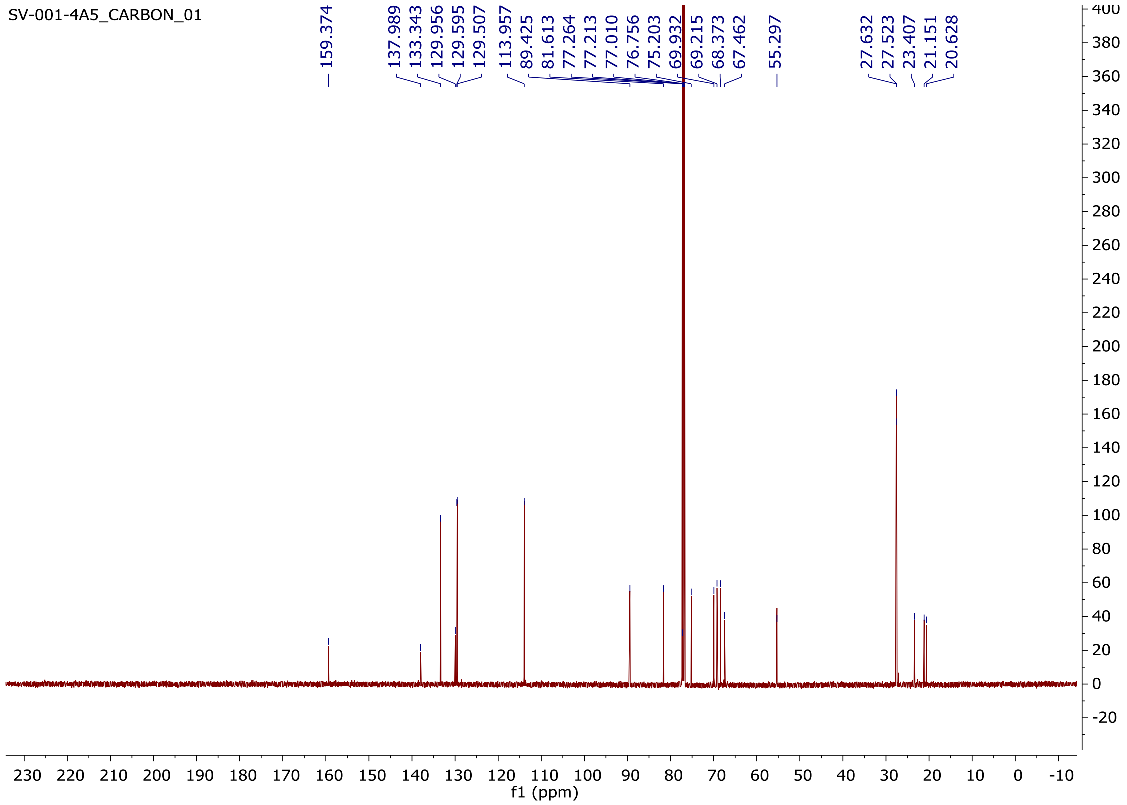

^{13}C NMR spectrum of compound **S1**

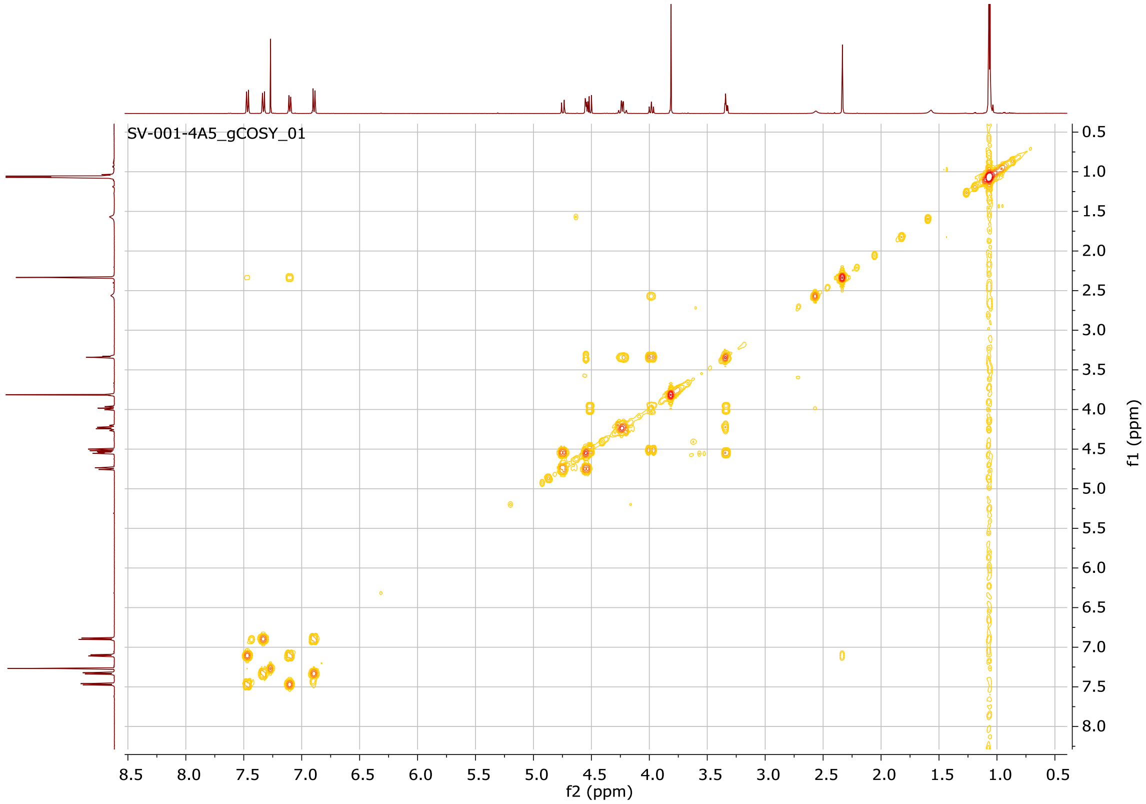

gCOSY NMR spectrum of compound **S1**

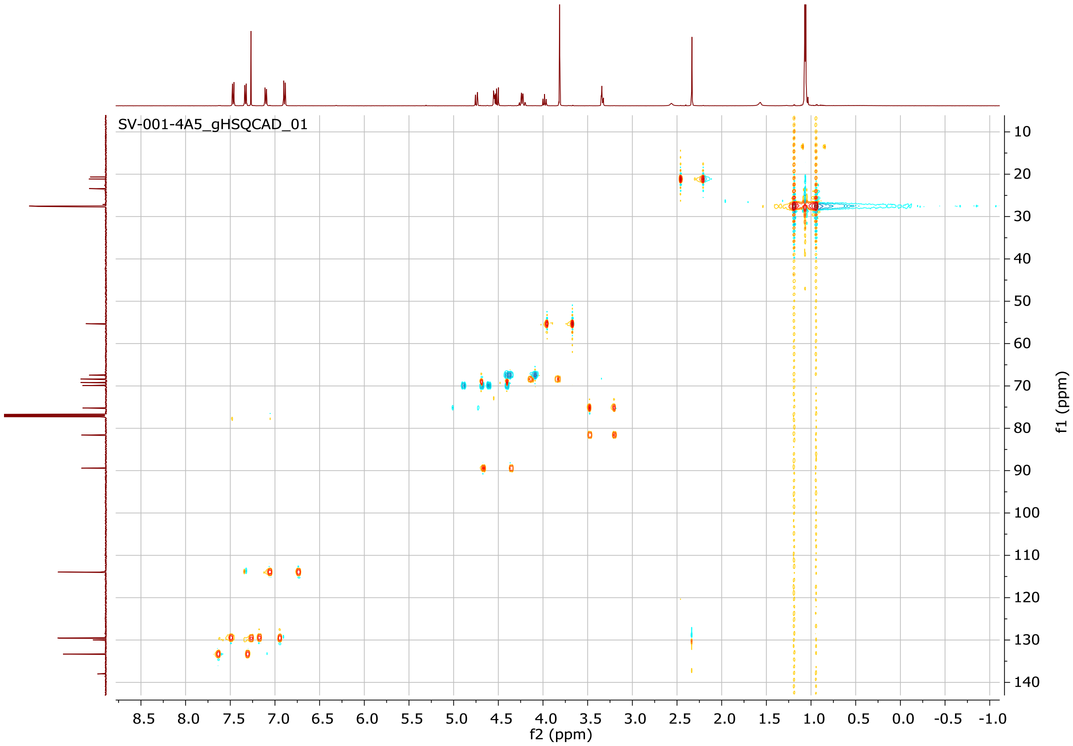

^{1}H-Coupled gHSQC NMR spectrum of compound **S1**

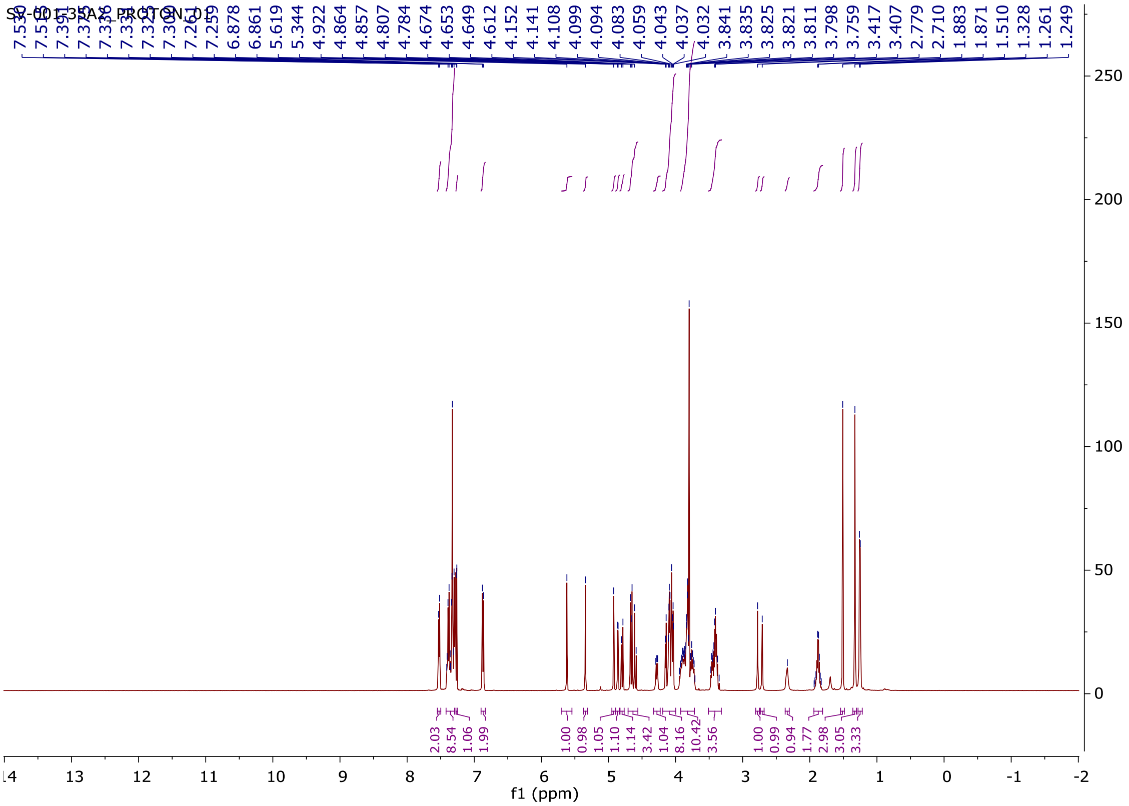

^{1}H NMR spectrum of compound **S2**

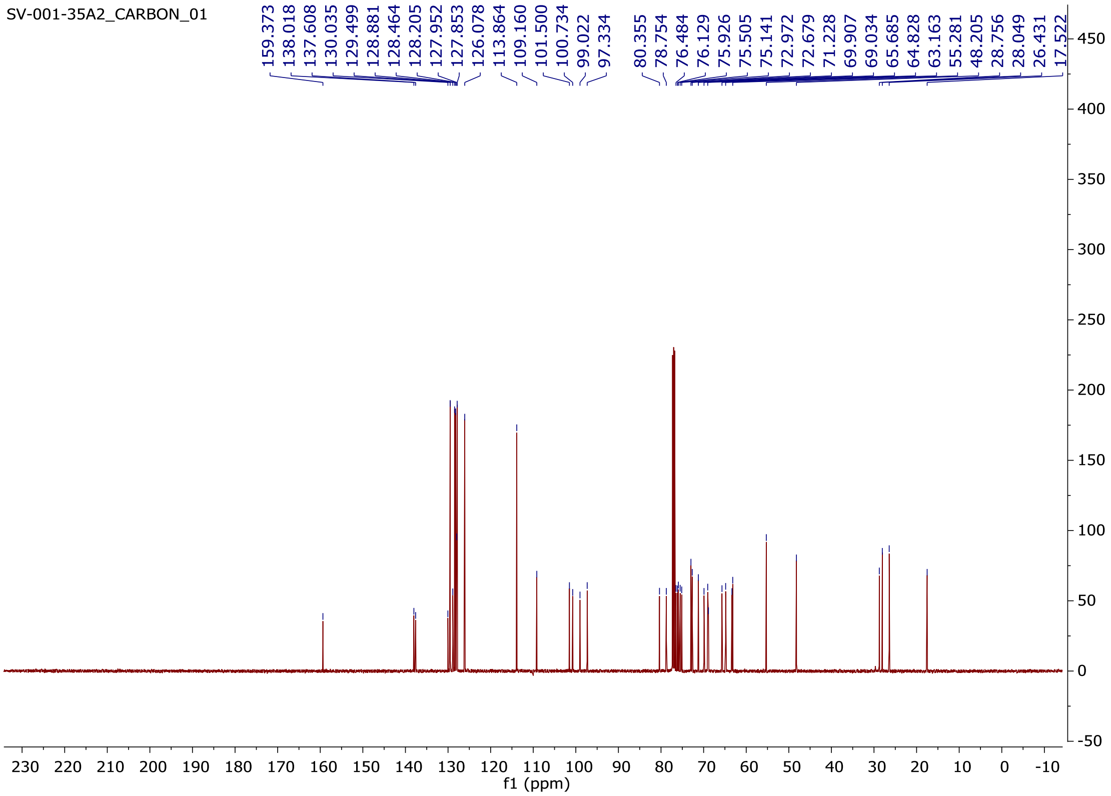

^{13}C NMR spectrum of compound **S2**

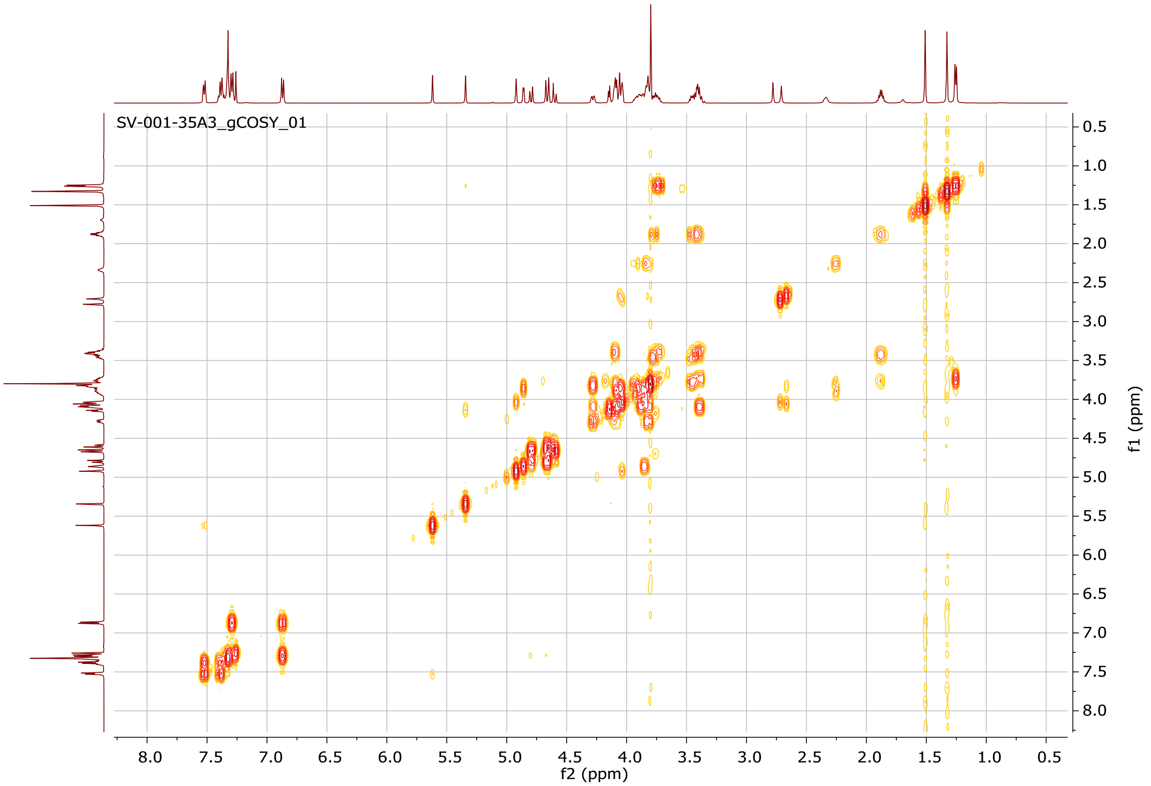

gCOSY spectrum of compound **S2**

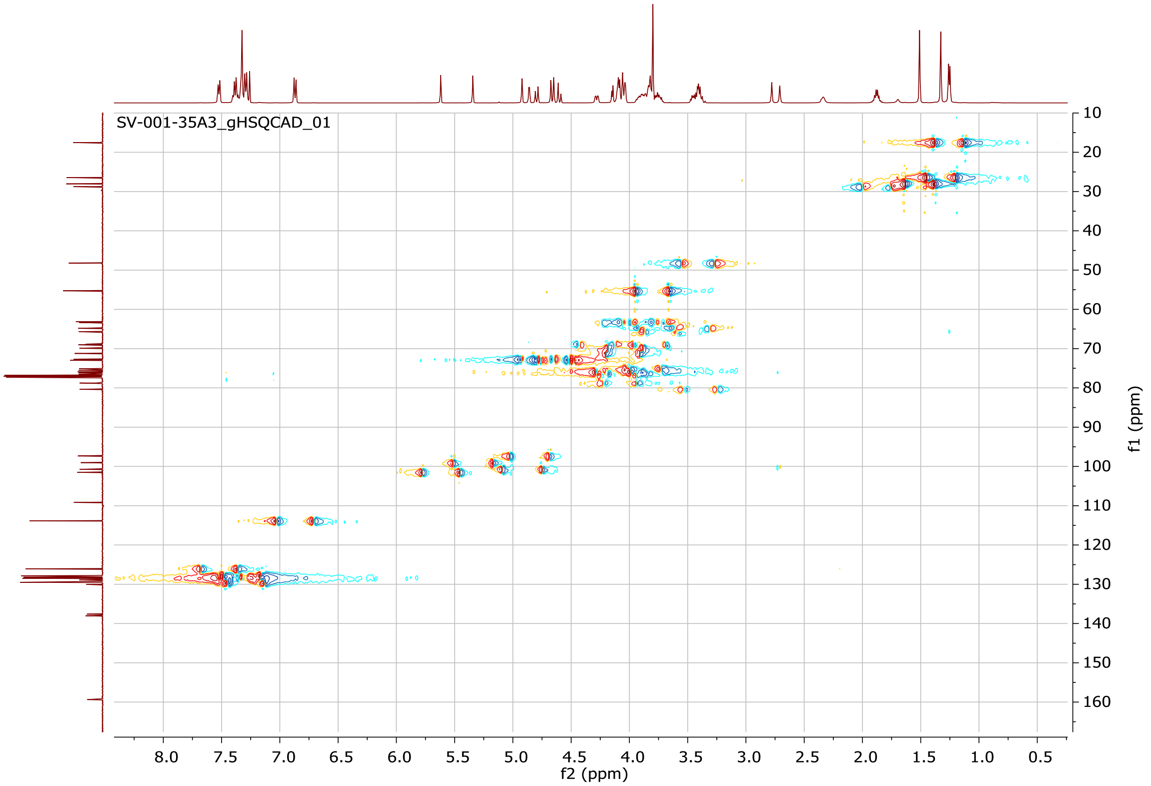

^{1}H-Coupled NMR spectrum of compound **S2**

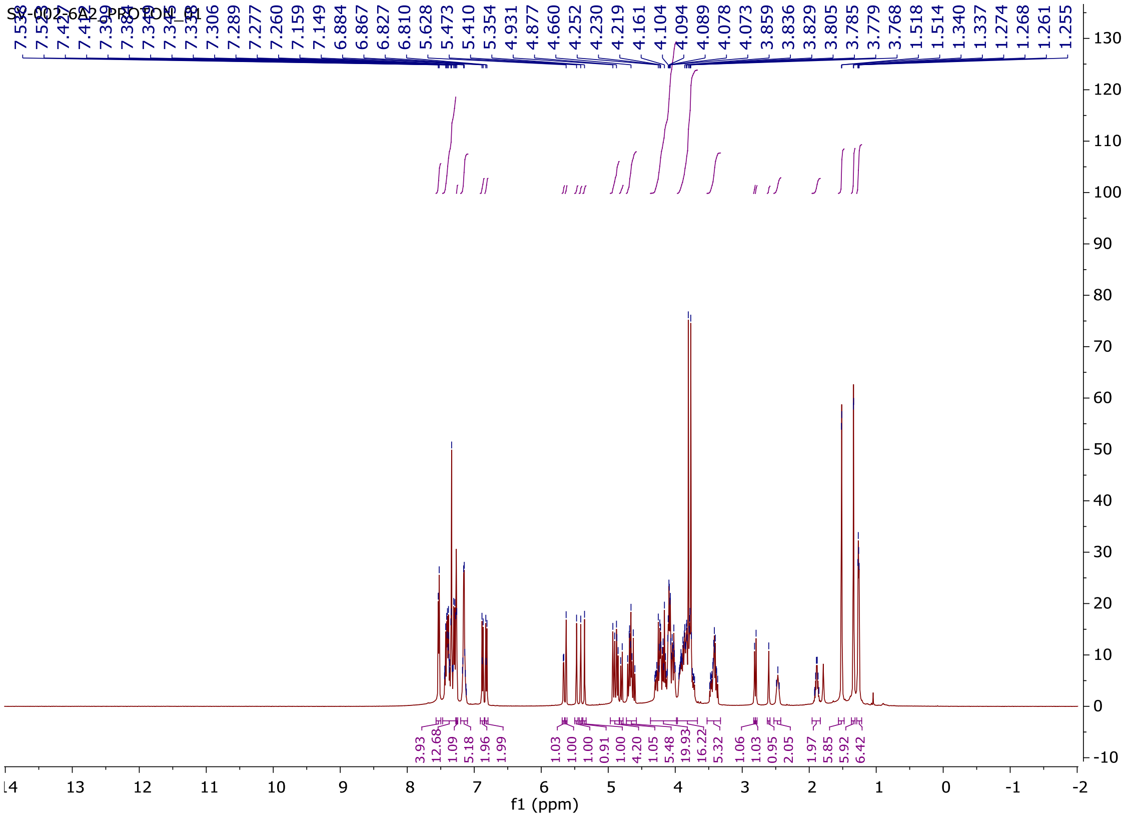

^{1}H NMR spectrum of compound **S3**

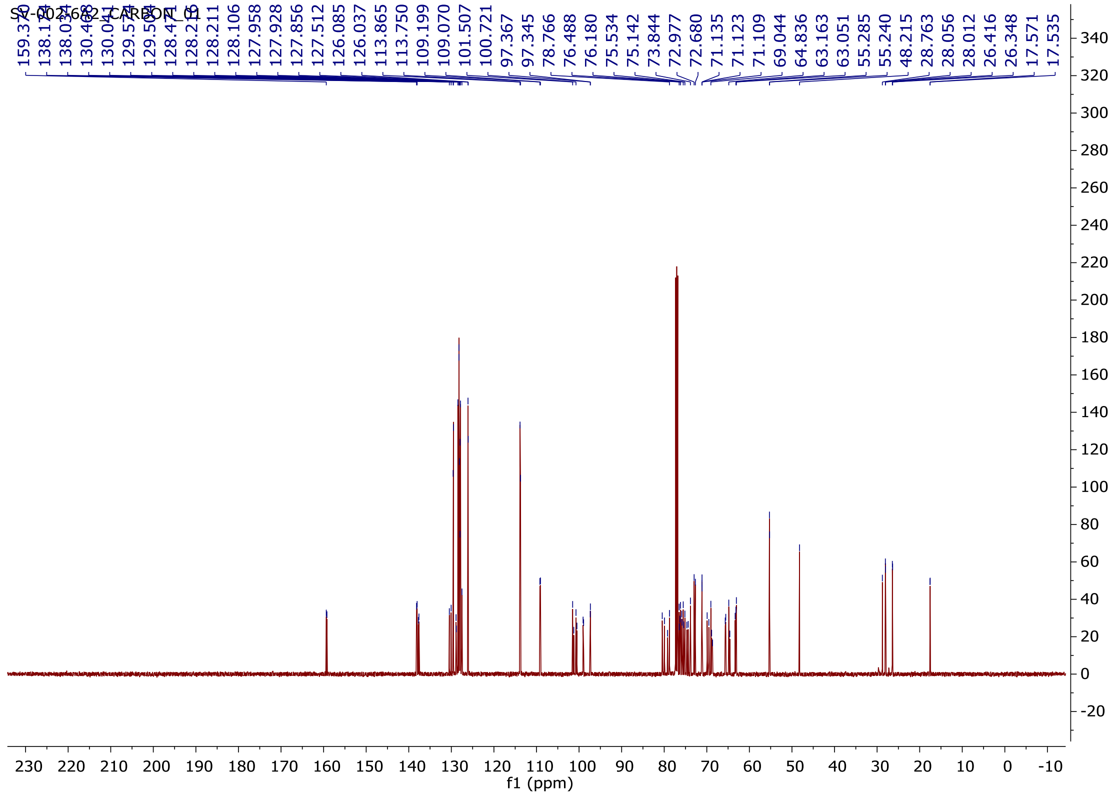

^{13}C NMR spectrum of compound **S3**

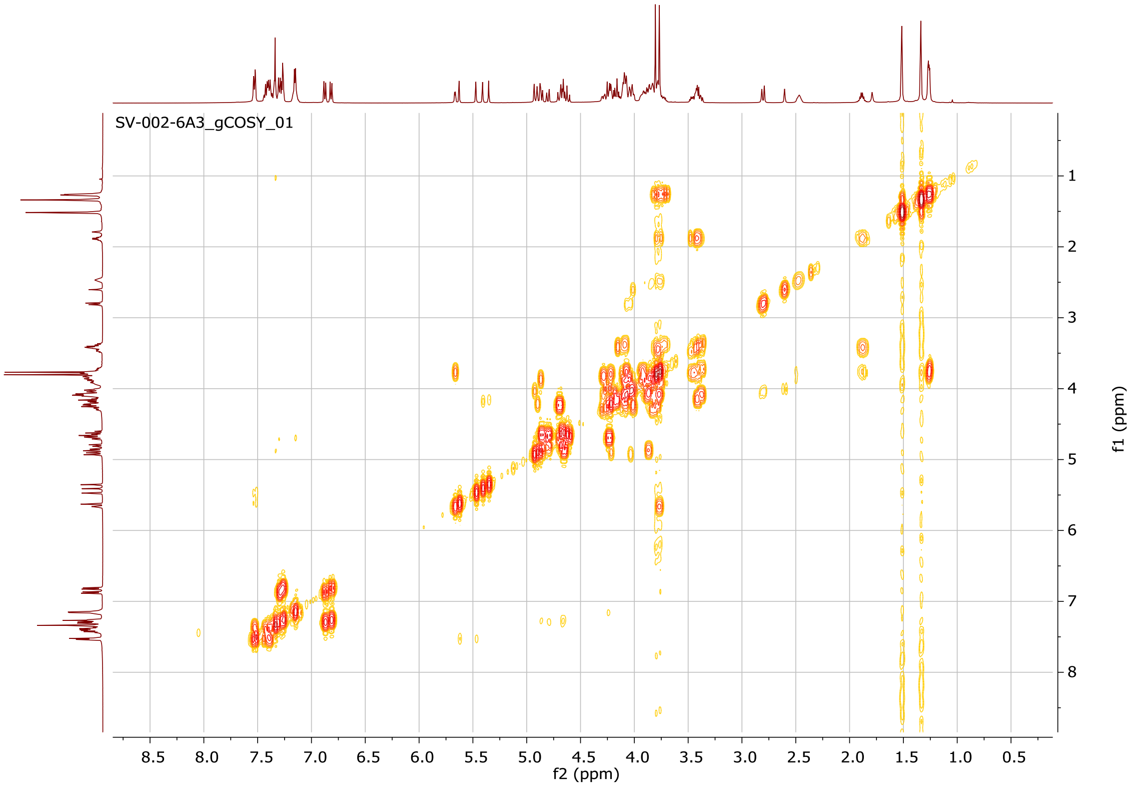

gCOSY NMR spectrum of compound **S3**

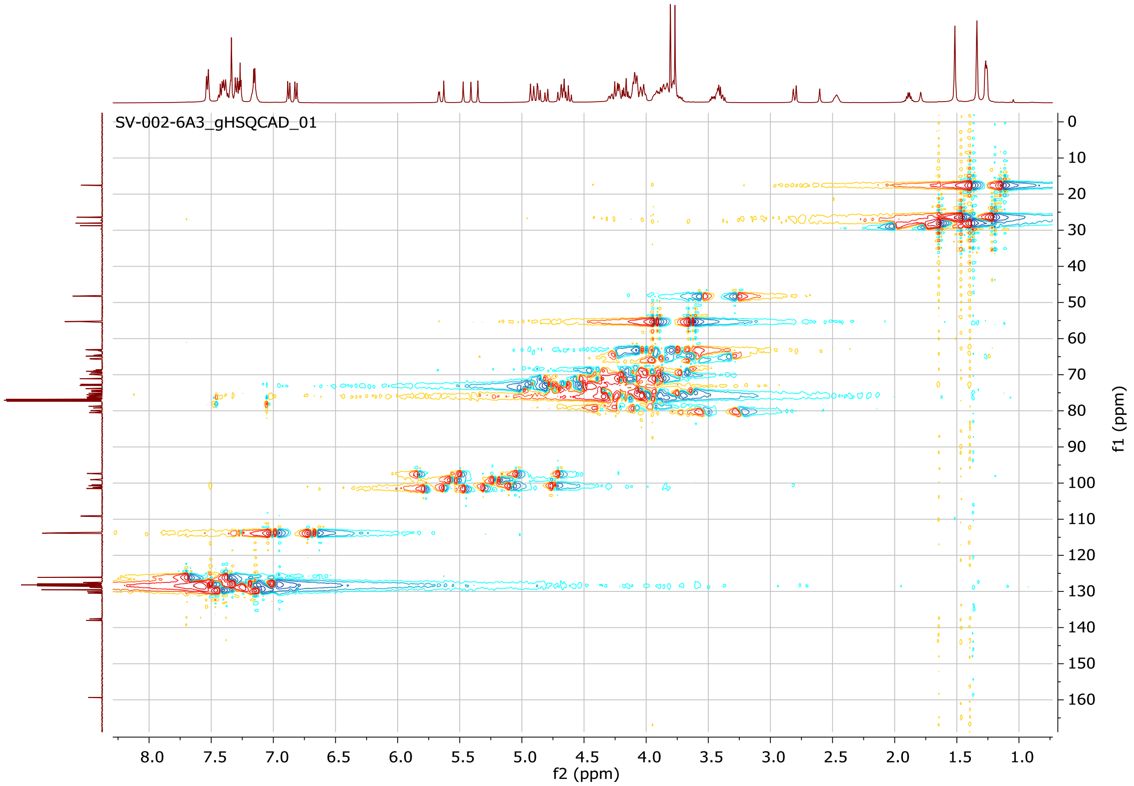

^{1}H-Coupled gHSQC NMR spectrum of compound **S3**

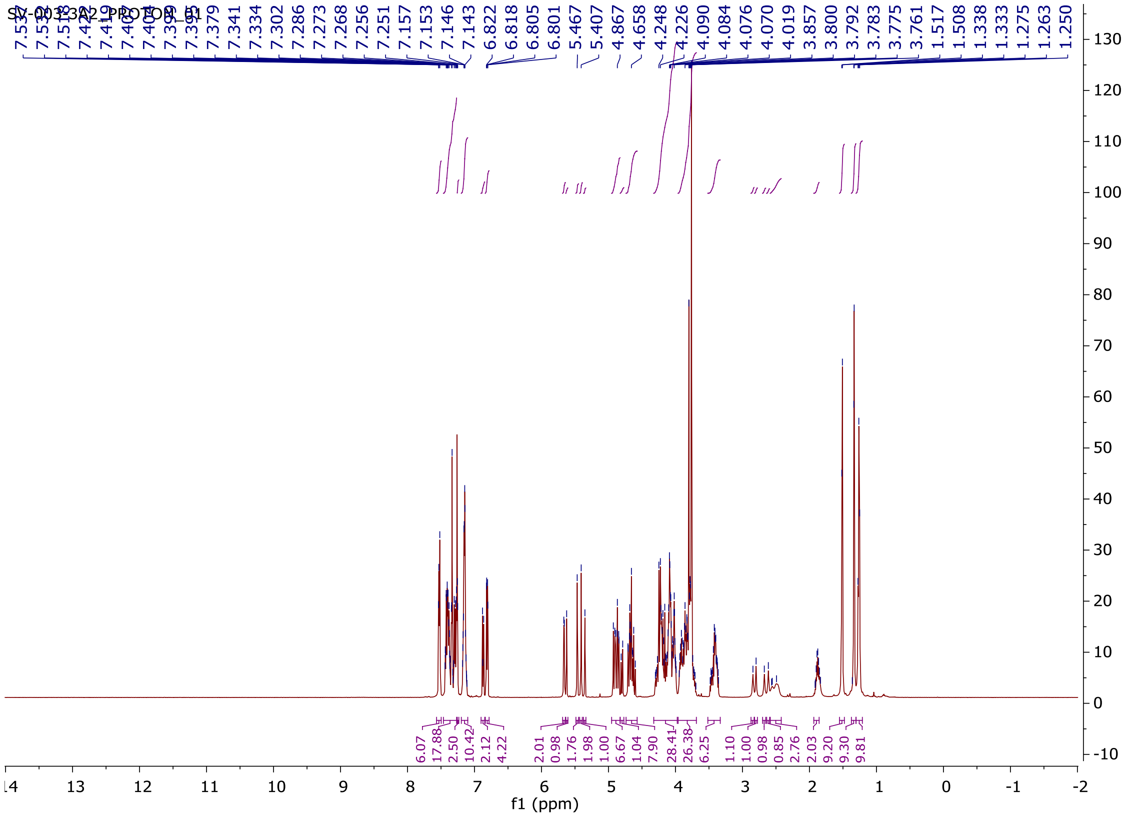

^{1}H NMR spectrum of compound **S4**

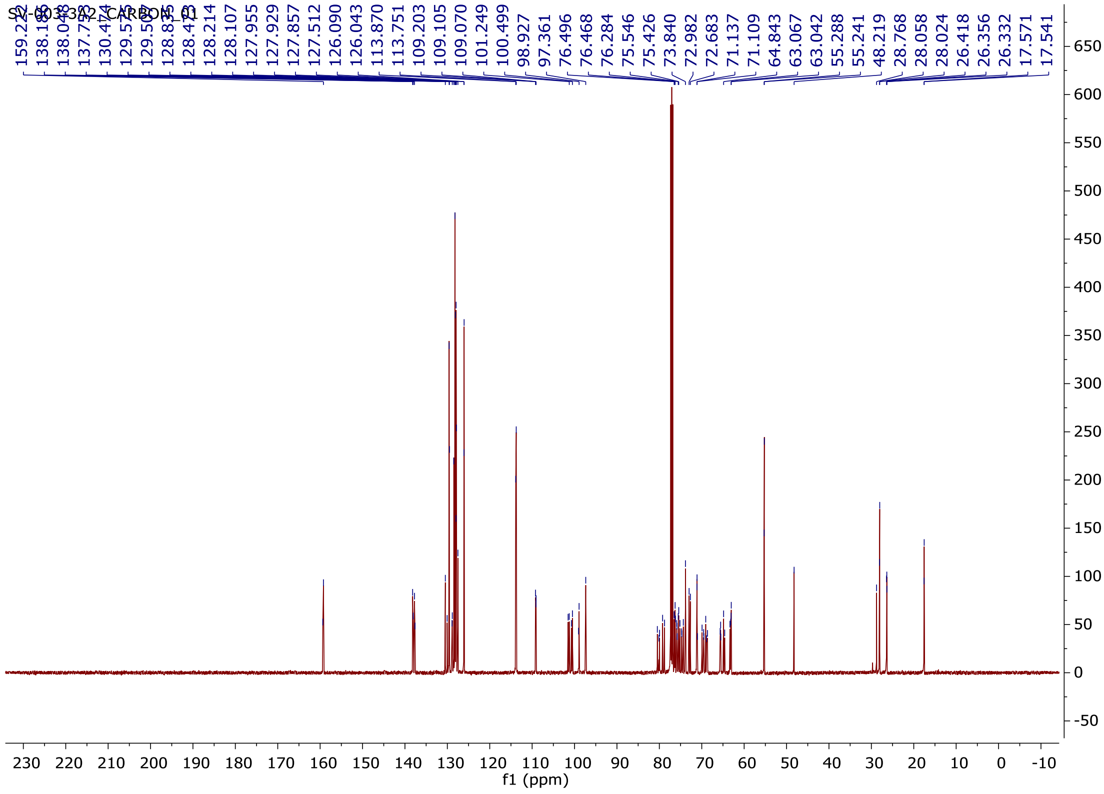

^{13}C NMR spectrum of compound **S4**

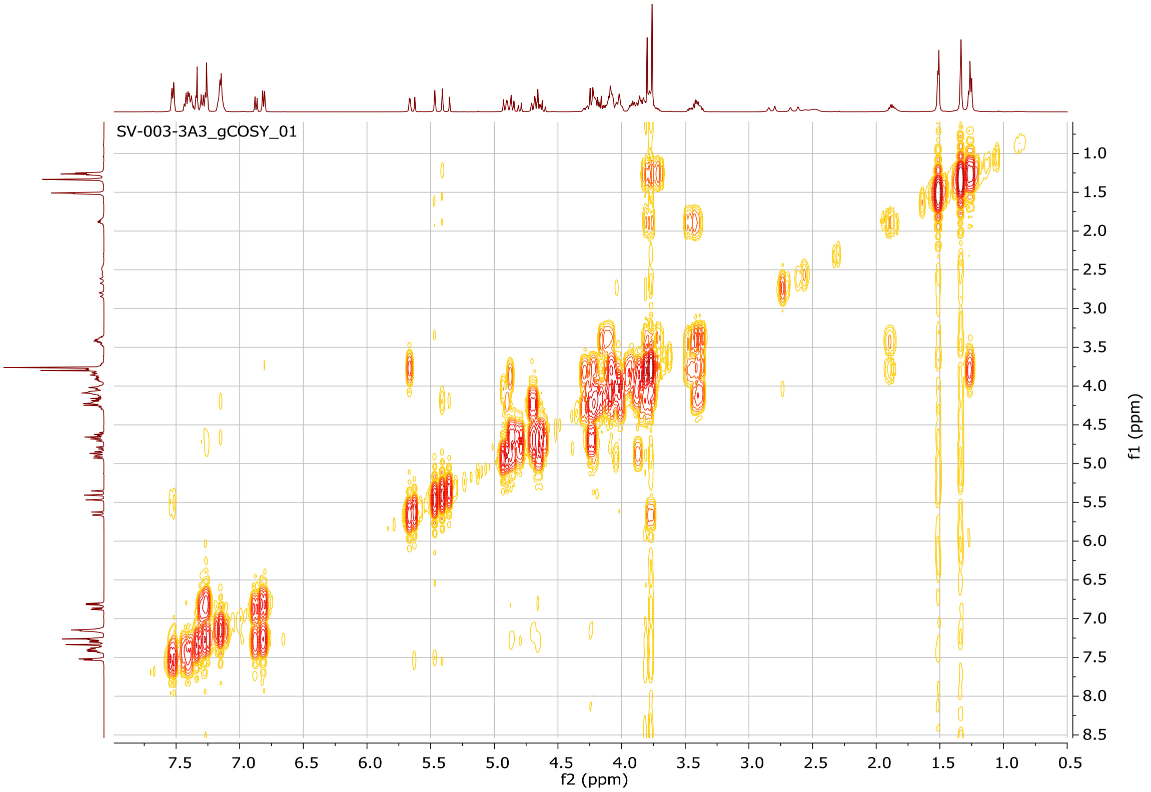

gCOSY NMR spectrum of compound **S4**

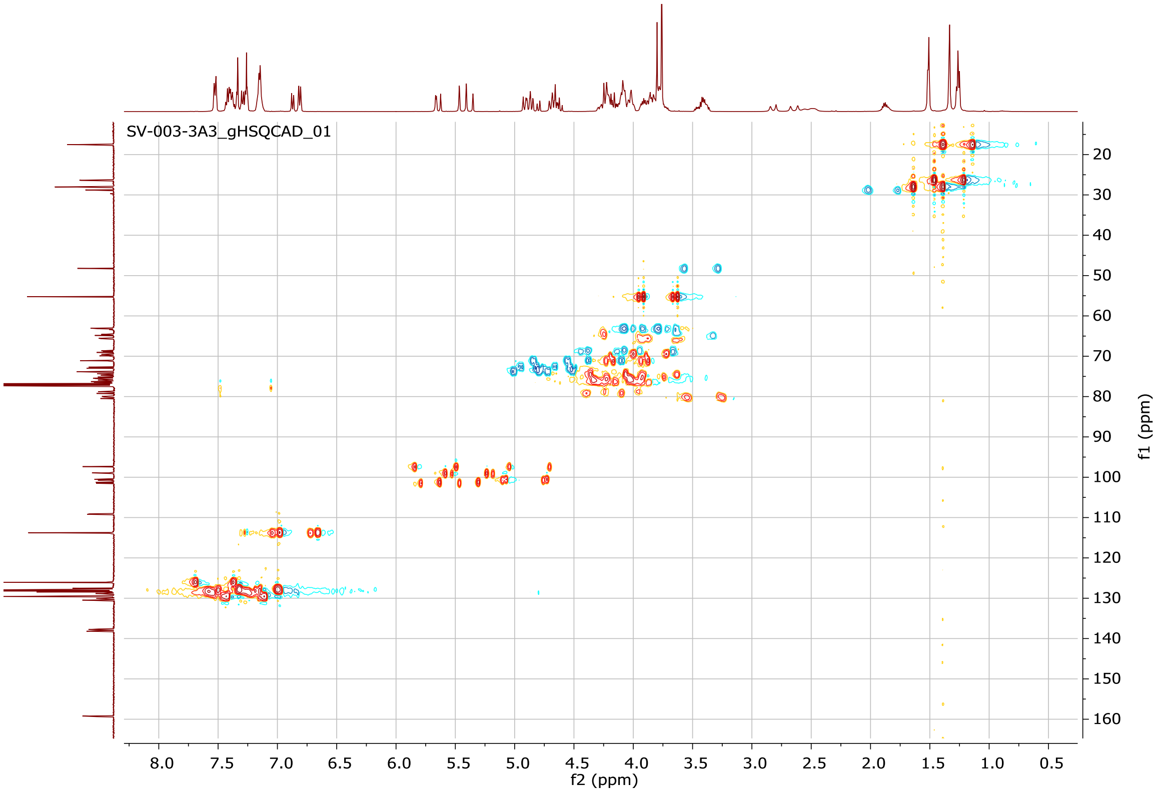

^{1}H-Coupled gHSQC NMR spectrum of compound **S4**
